# Application of unsupervised and supervised machine learning approaches to blood-based biomarkers of Alzheimer’s disease in a memory clinic cohort

**DOI:** 10.3389/fnagi.2026.1922577

**Published:** 2026-09-25

**Authors:** Edoardo Guido Torrigiani, Davide Cianca, Lorenzo Gaetani, Giovanna Nardi, Federico Paolini Paoletti, Davide Chiasserini, Lucilla Parnetti, Luca Gammaitoni, Igor Neri, Giovanni Bellomo

**Affiliations:** 1 Section of Neurology, Department of Medicine and Surgery, University of Perugia, Perugia, Italy; 2 Department of Physics and Geology, University of Perugia, Perugia, Italy; 3 Section of Physiology and Biochemistry, Department of Medicine and Surgery, University of Perugia, Perugia, Italy; 4 Department of Life Science, Health and Health Professions, Link Campus University, Rome, Italy

**Keywords:** Alzheimer’s disease, blood-based biomarkers, cutoff definition, machine learning, plasma biomarkers, supervised classification, unsupervised clustering

## Abstract

**Introduction:**

Blood-based biomarkers (BBMs) are promising tools to facilitate biological diagnosis of Alzheimer’s Disease (AD). Establishing reliable cutoffs for BBMs represents a major issue for real-world implementation and the investigation of combined BBM approaches is limited. Our aim is to test different machine learning (ML) models for BBMs interpretation with respect to established gold-standard *in vivo* biomarkers, i.e., cerebrospinal fluid (CSF) biomarkers, to enhance their standalone and combined diagnostic value for AD.

**Methods:**

We selected 434 patients who referred to Perugia University Hospital for perceived cognitive decline with available CSF A β 42/40, pTau181 and tTau and plasma Aβ42, Aβ40, pTau217, pTau181 and NfL. Multiple unsupervised clustering algorithms for cutoff determination and supervised classification models were compared for the prediction of AD defined by a CSF A^+^/T ^+^ profile.

**Results:**

Different unsupervised clustering models obtained cutoffs with high performances in predicting an AD CSF profile (accuracy > 0.85). Each combination of cutoffs was tested, the best ones achieving A^+^/T^+^ CSF profile prevalences > 0.9 in BBM-positive subgroups. The best supervised ML models achieved accuracies up to 0.88 in 10-fold cross-validation.

**Discussion:**

Our results prove that clustering approaches can help in identifying reliable BBMs cutoffs for AD diagnosis even in the absence of CSF gold standard reference and that ML approaches combining multiple BBMs can strongly enhance their diagnostic accuracy.

## 1 Introduction

Since brain-derived biomarkers reflecting Alzheimer’s Disease (AD) neuropathologic changes (NPC) were identified in the bloodstream (Arslan et al., 2024; Janelidze et al., 2020, 2021), there has been a growing interest for their reliable measurement and implementation as blood-based biomarkers (BBMs) hold promise for extending and accelerating the biological diagnosis of AD to larger cohorts, with impactful clinical outcomes, especially in the forthcoming era of disease modifying therapies (Cummings J. et al., 2024). As ultrasensitive techniques made it possible to reliably measure AD BBMs (Resmi et al., 2024), the development of fully automated assays (Bellomo et al., 2024; Pilotto et al., 2025; Musso et al., 2025) have provided a cost-effective, scalable instrument for reliable quantification of AD BBMs in clinical settings.

In this sense, BBMs are currently regarded as the most scalable candidate markers to complement or even replace cerebrospinal fluid (Palmqvist et al., 2025b) (CSF) and nuclear medicine biomarkers in the diagnosis of AD.

Of all BBMs, research has directed its attention to phosphorylated Tau isoforms (pTaus) (Wojdala et al., 2023, 2024; Anastasi et al., 2025) as the most promising standalone BBMs, and in particular to pTau phosphorylated at threonine 217 (pTau217), and its ratios to plasma β -amyloid fragment 1–42 (A β 42) and β -amyloid fragment 1–40 (A β 40) (Palmqvist et al., 2024), which seem to be the least affected by systemic confounding factors and the best candidates regarding the concordance with CSF and nuclear medicine AD biomarkers (Arslan et al., 2024; Bellomo et al., 2024; Gaetani et al., 2026).

Current approaches for BBMs implementation are clinically driven and focused on standardizing analytical procedures and developing interpretative frameworks, such as three-tiered cutoff systems based on gold-standard biomarkers of AD (CSF and nuclear medicine biomarkers) with two reference thresholds that, respectively, maximize sensitivity and specificity (Schindler et al., 2024; Brum et al., 2023). Such approaches are aimed at mitigating the impact of the overlap between AD cases and controls, defined either through gold standard CSF or amyloid PET biomarkers, in intermediate BBMs concentrations. In these cases, the presence of systemic confounding factors, like kidney disease, can affect BBMs diagnostic accuracies (Olvera-Rojas et al., 2024).

Other approaches are focused on the identification of different cutoffs for AD BBMs according to age, chronic health conditions (Zheng et al., 2024), the presence of other neurodegenerative copathologies (Weinshel et al., 2022) or the degree of cognitive impairment (Elahi et al., 2020). Although tailored cutoffs may improve BBMs diagnostic accuracy in selected populations, they could also reduce interpretability and operational simplicity, potentially limiting the scalability and widespread clinical implementation of these biomarkers.

Currently, the identification of BBMs cutoff values relies on the availability of gold standard biomarkers to serve as a reference. Clinical cohorts with available gold-standard AD biomarkers (CSF or amyloid PET) can be used to comparatively test unsupervised machine learning (ML) approaches for the identification of BBMs cutoff values and supervised ML approaches to enhance diagnostic accuracy of BBMs in AD diagnosis.

In fact, ML algorithms have recently gained interest for biomedical applications. Unsupervised clustering algorithms have already been applied in the definition of cutoffs for AD CSF biomarkers in the absence of neuropathologic reference (Bellomo et al., 2021) and more complex supervised classification algorithms, such as support vector machines (Maltarollo et al., 2019) (SVM), gradient boosting methods (Li et al., 2024), and neural networks (Rumelhart et al., 1986; Ching et al., 2018) have been applied to refine diagnostic workups for complex cases.

The rationale for applying unsupervised clustering was to explore whether biologically meaningful BBM cutoffs could be derived independently of CSF or PET reference biomarkers, thereby providing a potentially scalable framework for biomarker implementation in settings where gold-standard biomarkers are unavailable.

In this study, we aimed to assess the performances of multiple unsupervised clustering algorithms for the definition of reliable cutoffs for AD BBMs in a real-world cohort and to test the performance of each cutoff alone and combined for the identification of AD, i.e., a CSF A^+^/T^+^ profile, according to the 2018 NIA-AA A/T/(N) framework (Jack et al., 2018), which has been proven to reliably reflect the simultaneous presence of cerebral amyloidosis and tauopathy, the core neuropathological features of AD (Wang et al., 2023). We also aimed to test the ability of multiple supervised ML algorithms to identify patients with underlying AD pathology (as defined by a CSF A^+^/T^+^ profile) through a large panel of BBMs.

## 2 Materials and methods

### 2.1 Study population

We retrospectively selected 434 patients who referred to Neurology Section of Perugia University Hospital from 2013 to 2025 (Table 1) for perceived cognitive decline. For each of them, diagnostic workup consisted of clinical and neuropsychological evaluation, at least comprehensive of MiniMental State Examination (MMSE) (Folstein et al., 1975) and Clinical Dementia Rating Scale (CDR) (Hughes et al., 1982) and CSF and plasma collection. Diagnoses were reached evaluating clinico-biological profile of each patient according to current diagnostic criteria (Jack et al., 2024; Postuma et al., 2015; Rascovsky et al., 2011; Gorno-Tempini et al., 2011; McKeith et al., 2017).

**Table 1:** Demographic and biomarker data for each diagnostic group.

Variable	preAD	AD-MCI	FTD	PD-CN	PDD/DLB	CTRL-CN	CTRL-MCI	ADdem	PD-MCI
*n*	24	122	17	35	11	35	63	88	39
Age	72.33 ± 5.13	72.89 ± 5.97	73.24 ± 5.03	65.94 ± 6.28	72.55 ± 10.49	66.77 ± 8.59	71.62 ± 6.20	73.22 ± 6.72	68.74 ± 6.59
Education	10.38 ± 4.48	10.13 ± 4.59	10.59 ± 3.99	11.66 ± 4.14	9.55 ± 4.66	12.71 ± 4.76	8.86 ± 4.28	8.78 ± 4.35	9.64 ± 3.46
Sex (F/M percentage)	58.3/41.7	67.4/32.6	50/50	44.4/55.6	10/90	43.6/56	43.1/56.9	71.5/28.5	27.9/72.1
MMSE	28.08 ± 1.56	23.96 ± 3.11	22.12 ± 4.00	28.37 ± 1.40	20.36 ± 5.89	27.37 ± 5.05	25.59 ± 2.49	14.98 ± 6.23	25.92 ± 2.04
CDR	0.00 ± 0.00	0.50 ± 0.00	0.76 ± 0.47	0.01 ± 0.08	1.18 ± 0.56	0.01 ± 0.08	0.48 ± 0.09	1.28 ± 0.57	0.47 ± 0.11
CSF A*β*40	14098.79 ± 3370.88	12632.18 ± 4270.08	10409.76 ± 3879.50	10576.49 ± 3288.67	10118.64 ± 3362.65	12130.25 ± 4804.25	10852.71 ± 4623.04	12229.07 ± 3620.45	10139.64 ± 3603.24
CSF A*β*42	742.79 ± 175.06	594.17 ± 209.66	874.35 ± 422.71	1081.61 ± 300.12	574.18 ± 247.90	1315.59 ± 668.93	1054.46 ± 511.13	574.31 ± 180.87	983.28 ± 426.12
CSF A*β*42/40	0.05 ± 0.01	0.05 ± 0.01	0.08 ± 0.02	0.11 ± 0.02	0.06 ± 0.02	0.11 ± 0.03	0.10 ± 0.02	0.05 ± 0.01	0.10 ± 0.03
CSF pTau181	74.39 ± 20.70	111.33 ± 61.16	42.24 ± 17.70	38.28 ± 16.64	63.87 ± 36.47	45.47 ± 20.88	41.60 ± 14.30	123.58 ± 52.80	41.30 ± 18.68
CSF NfL	3323.45 ± 1508.74	2319.73 ± 1618.01	2790.37 ± 563.19	1292.67 ± 587.49	3047.98 ± nan	1357.04 ± 716.10	1824.73 ± 1784.93	2246.81 ± 1050.51	4172.49 ± nan
CSF tTau	515.58 ± 164.45	750.06 ± 361.03	348.59 ± 161.38	283.95 ± 202.11	426.36 ± 227.12	323.73 ± 161.59	313.91 ± 165.46	918.11 ± 435.61	286.75 ± 152.26
Plasma A*β*42/40	0.08 ± 0.01	0.08 ± 0.01	0.08 ± 0.01	0.09 ± 0.03	0.08 ± 0.01	0.09 ± 0.02	0.08 ± 0.02	0.08 ± 0.01	0.09 ± 0.02
Plasma pTau181	1.82 ± 0.68	2.80 ± 1.43	2.06 ± 0.73	1.09 ± 0.54	2.45 ± 1.04	1.67 ± 1.49	1.73 ± 1.00	3.32 ± 1.03	1.33 ± 0.63
Plasma pTau217	0.26 ± 0.16	0.62 ± 0.35	0.25 ± 0.23	0.07 ± 0.04	0.43 ± 0.35	0.12 ± 0.14	0.16 ± 0.19	0.90 ± 0.51	0.12 ± 0.09
Plasma NfL	19.10 ± 13.52	24.26 ± 11.18	35.83 ± 18.93	15.39 ± 5.99	29.30 ± 13.47	19.81 ± 25.47	22.93 ± 18.54	30.95 ± 15.78	19.50 ± 13.18
Plasma pTau217/A*β*40	0.08 ± 0.04	0.21 ± 0.12	0.07 ± 0.06	0.03 ± 0.01	0.14 ± 0.10	0.04 ± 0.05	0.05 ± 0.06	0.29 ± 0.17	0.04 ± 0.03
Plasma pTau217/A*β*42	1.08 ± 0.59	2.75 ± 1.62	0.98 ± 0.85	0.38 ± 0.60	1.93 ± 1.51	0.63 ± 0.89	0.66 ± 0.86	3.85 ± 2.12	0.50 ± 0.37
Plasma pTau181/A*β*40	0.59 ± 0.16	0.92 ± 0.47	0.61 ± 0.20	0.38 ± 0.16	0.89 ± 0.33	0.57 ± 0.44	0.53 ± 0.32	1.07 ± 0.32	0.49 ± 0.26

preAD: preclinical Alzheimer’s disease; AD-MCI: Mild Cognitive Impairment due to Alzheimer’s disease; ADdem: Alzheimer’s disease dementia; FTD: fronto-temporal dementia; PD-CN: cognitively unimpaired Parkinson’s disease; PD-MCI: Parkinson’s disease with Mild Cognitive Impairment; PDD/DLB: Parkinson’s disease dementia/dementia with Lewy bodies; CTRL-CN: cognitively unimpaired controls; CTRL-MCI: non-neurodegenerative Mild Cognitive Impairment; MMSE: MiniMental state examination; n: number of patients per subgroup; Age: age at lumbar puncture and blood sampling; Education: years of formal education. All biomarker values are expressed as pg/mL, while ratios are expressed as absolute numbers. Plasma pTau/A*β* ratios are shown as multiplied by 100 for better visualization; original values were used for all analyses.

Namely, diagnostic groups included: preclinical AD (preAD), mild cognitive impairment due to AD (AD-MCI), dementia due to AD (ADDem), Parkinson’s Disease (PD), both cognitively unimpaired (PD-CN) and with mild cognitive impairment (PDMCI), PD dementia and Dementia with Lewy Bodies (PDD/DLB), Fronto-Temporal Dementia (FTD) and control subjects, both cognitively unimpaired and with MCI (CTRL-CN, CTRL-MCI). Control subjects were selected among those subjects who underwent lumbar puncture for CSF analysis within the diagnostic work-up for self-perceived cognitive decline or other, non neurodegenerative neurological diseases (headache, mono−/poli-neuropathy, psychiatric disturbances). Their CSF profile was negative for AD (A^−^/T^−^). According to the neuropsychological evaluation, these subjects were either cognitively unimpaired (CTRL-CN) or had MCI (CTRL-MCI). Only CTRL-MCI subjects with stable cognitive performances at follow-up were included. In our cohort, 37 subjects had a known medical history of kidney disease, as defined by estimated glomerular filtration rate below 60 mL/min.

### 2.2 Biological measures

Lumbar puncture was performed according to international guidelines (Teunissen et al., 2009); 10–12 mL of CSF were collected in sterile polypropylene tubes (Sarstedt tubes, code: 62.610.210) and centrifuged for 10 min (2000 × g), at room temperature. 0.5 mL aliquots were frozen at $-80^{\circ }$ C in polypropylene tubes (Sarstedt tubes, code: 72.730.007). CSF was analyzed with a fully automated chemiluminescent platform (Lumipulse G600-II-Fujirebio Inc) for β -amyloid fragment 1–42 (A β 42), β -amyloid fragment 1–40 (A β 40), t-Tau and p-Tau (Thr181) levels, according to the instructions from the manufacturer. All CSF samples were analyzed directly in their 0.5 mL storage tubes.

Blood samples were collected at the same date of lumbar puncture from all participants in dipotassium EDTA anticoagulant tubes through venipuncture. Samples were centrifuged for 10 min at 1800–2000 × *g* at room temperature, 0.5 mL plasma aliquots were stored in polypropylene tubes (Sarstedt tubes) at $-80^{\circ }$ C.

For biomarker measurement, plasma tubes were preliminarily thawed at room temperature (15°C to 25°C) for 30 min, vortexed for 10 s, and centrifuged for 5 min at 2000 × g. Plasma A β 40, A β 42, pTau181, pTau217, Neurofilament Light Chain (NfL) measurements were performed on the Lumipulse G System (Lumipulse G1200) according to the instructions provided by the manufacturer.

All measures were performed at the Laboratory of Clinical Neurochemistry, Section of Neurology, Department of Medicine and Surgery, University of Perugia, Perugia (IT).

Protein ratios were calculated between plasma pTau217 and A β 42, plasma pTau217 and A β 40, plasma pTau181 and A β 40 as plasma pTau217/A β 42 has shown to outperform plasma pTau181/A β 42 ratio in detecting A^+^ status (Lehmann et al., 2025). Furthermore, BBMs ratios with A β 40 are used to normalize brain-derived biomarkers concentrations for systemic variability (Thijssen et al., 2021) and recent evidence has confirmed A β 40’s role as a normalizator for several CSF and plasma biomarkers with respect to tau and amyloid PET (Karlsson, 2026). A supplementary analysis including the plasma pTau181/A β 42 ratio as an eighth marker (*n* = 255 combinations) confirmed that its addition did not substantially change the diagnostic performance of biomarker combinations (Supplementary material 2). CSF profiling was based on the A/T/(N) framework (Jack et al., 2018) according to previously published and locally validated Lumipulse cutoffs: Aβ42/Aβ40 < 0.072, pTau181 > 50 pg./mL and tTau > 392 pg./mL (Bellomo et al., 2021).

### 2.3 Statistical analysis

For cutoff derivation, we excluded subjects presenting outlier values in any of the seven plasma biomarkers and biomarker ratios. Outliers were defined using the conventional 1.5 × interquartile range rule applied independently to each biomarker, and exclusion was performed cumulatively across markers such that a subject was removed if identified as an outlier for one or more measures. Once cutoffs had been established, the excluded subjects were reinstated and included in the subsequent diagnostic accuracy analyses. All accuracy analyses were performed with CSF A^+^/T^+^ status as a reference.

#### 2.3.1 Unsupervised clustering for cutoff definition

Seven unsupervised clustering algorithms were applied: Gaussian Mixture Model (GMM) (Dempster et al., 1977), K-Means (Lloyd, 1982), MiniBatch K-Means (Sculley, 2010), Agglomerative Clustering with Ward linkage (Ward, 1963), Agglomerative Clustering with Complete linkage (Defays, 1977), Spectral Clustering (Ng et al., 2002), and Birch (Zhang et al., 1996). All of these methods used as input variables plasma A β 42/40, plasma pTau217, plasma pTau181, plasma NfL, plasma pTau217/A β 40, plasma pTau217/A β 42 and plasma pTau181/A β 40, standardized via z-score normalization. All algorithms were forced to partition the dataset into two clusters, following the assumption that one of the two clusters would result enriched in AD patients (AD-like cluster) and the other in non-AD patients.

The AD-like cluster was identified as the one with higher mean plasma pTau217 values (Palmqvist et al., 2024). Clustering performance was evaluated through Silhouette Score (Rousseeuw, 1987), which measures the similarity of each data point to its own cluster relative to other clusters (ranging from −1 to +1 , with higher values indicating better-defined clusters), and Calinski-Harabasz Index (Calinski and Harabasz, 1974), defined as the ratio of between-cluster to within-cluster dispersion (higher values indicating more distinct clusters).

Three cutoff values were determined for each BBM from the Gaussian distributions fitted to each cluster: one cutoff at the intersection point of the two fitted Gaussian curves, representing the optimal discrimination boundary between clusters, one cutoff maximizing sensitivity ( ∼ 95%) and one cutoff maximizing specificity ( ∼ 95%).

#### 2.3.2 Cutoff combination testing

The prevalence of CSF AD profile was then measured in each subgroup defined by all possible combinations of plasma biomarker intersectional cutoff values (i.e., the values at the intersection of the two fitted Gaussian curves, as described above), ranging from single biomarkers to combinations including all seven biomarkers ( n=127 combinations). A patient was considered positive for a given combination when all biomarkers in the combination showed values beyond their respective intersectional cutoffs. The prevalence of a non-AD CSF profile was also evaluated in the concordantly negative subgroup, defined as patients whose values fell below (or above, for A β 42/40) all cutoffs in the combination. Patients with discordant biomarker results were not assigned to either subgroup.

#### 2.3.3 Supervised classification

Eleven supervised classification algorithms were applied for their ability to distinguish between CSF-defined AD (i.e., A^+^/T^+^) and non-AD profiles using the same set of plasma biomarkers: Support Vector Machine with RBF kernel (SVM-RBF) (Maltarollo et al., 2019), Support Vector Machine with linear kernel (SVM-Linear), Logistic Regression (LR) (Cox, 1958), Random Forest (RF) (Breiman, 2001), Gradient Boosting (Li et al., 2024), AdaBoost (Freund and Schapire, 1997), Extra Trees (Geurts et al., 2006), K-Nearest Neighbors (KNN) (Cover and Hart, 1967), Gaussian Naive Bayes (Zhang, 2004), Decision Tree (Breiman et al., 1984), and Multilayer Perceptron (MLP) (Rumelhart et al., 1986).

Each model’s performance was assessed using stratified 10-fold cross-validation. To prevent data leakage, feature standardization was done within each fold using a pipeline architecture. Performance was assessed through accuracy, precision, recall, and F1-score.

#### 2.3.4 Software and libraries

All analyses were implemented in Python 3. Data manipulation was performed with pandas (The pandas development team, 2024) and NumPy (Harris et al., 2020). Clustering algorithms, supervised classifiers, cross-validation, feature scaling and evaluation metrics were provided by scikit-learn (Pedregosa et al., 2011). Gaussian distribution fitting and intersection-point computation relied on SciPy (Virtanen et al., 2020). Figures were generated with Matplotlib (Hunter, 2007).

## 3 Results

Demographic variables together with CSF and plasma biomarkers values among the different diagnostic groups are summarized in Table 1.

### 3.1 Clustering algorithm comparison

After removal of outlier values (1.5 × IQR), 379 patients were considered for clustering analysis. All seven clustering algorithms were applied to partition the dataset into two clusters (Table 2). Cluster sizes varied across algorithms: Agglomerative Clustering with Ward linkage produced the most balanced partition (189 AD-like, 190 non-AD-like), while Birch obtained the most unbalanced split (119 AD-like, 260 non-AD-like). GMM produced a moderately balanced partition (211 AD-like, 168 non-AD-like), while K-Means, MiniBatch K-Means, Agglomerative (Complete) and Spectral produced intermediate partitions with the AD-like cluster comprising 131 to 155 patients.

**Table 2:** Clustering algorithm comparison.

Algorithm	AD cluster	Non-AD cluster	Silhouette	Calinski-Harabasz
GMM	211	168	0.3404	272.4
K-Means	152	227	0.4336	395.19
MiniBatch K-Means	155	224	0.4323	394.21
Agglomerative (Ward)	189	190	0.3734	311.22
Agglomerative (Complete)	153	226	0.4233	379.83
Spectral	131	248	0.4351	385.07
Birch	119	260	0.4198	340.75

All algorithms were forced to partition the dataset into two clusters.

Clustering quality metrics showed that Spectral Clustering had the highest Silhouette Score (0.4351), followed closely by K-Means (0.4336) and MiniBatch K-Means (0.4323), while K-Means achieved the highest Calinski-Harabasz Index (395.19). GMM showed the lowest Silhouette Score (0.3404) and Calinski-Harabasz Index (272.40).

Clustering classification results with respect to CSF A β 42/40 and pTau181 distributions and cutoffs are shown in Figure 1.

**Figure 1:**
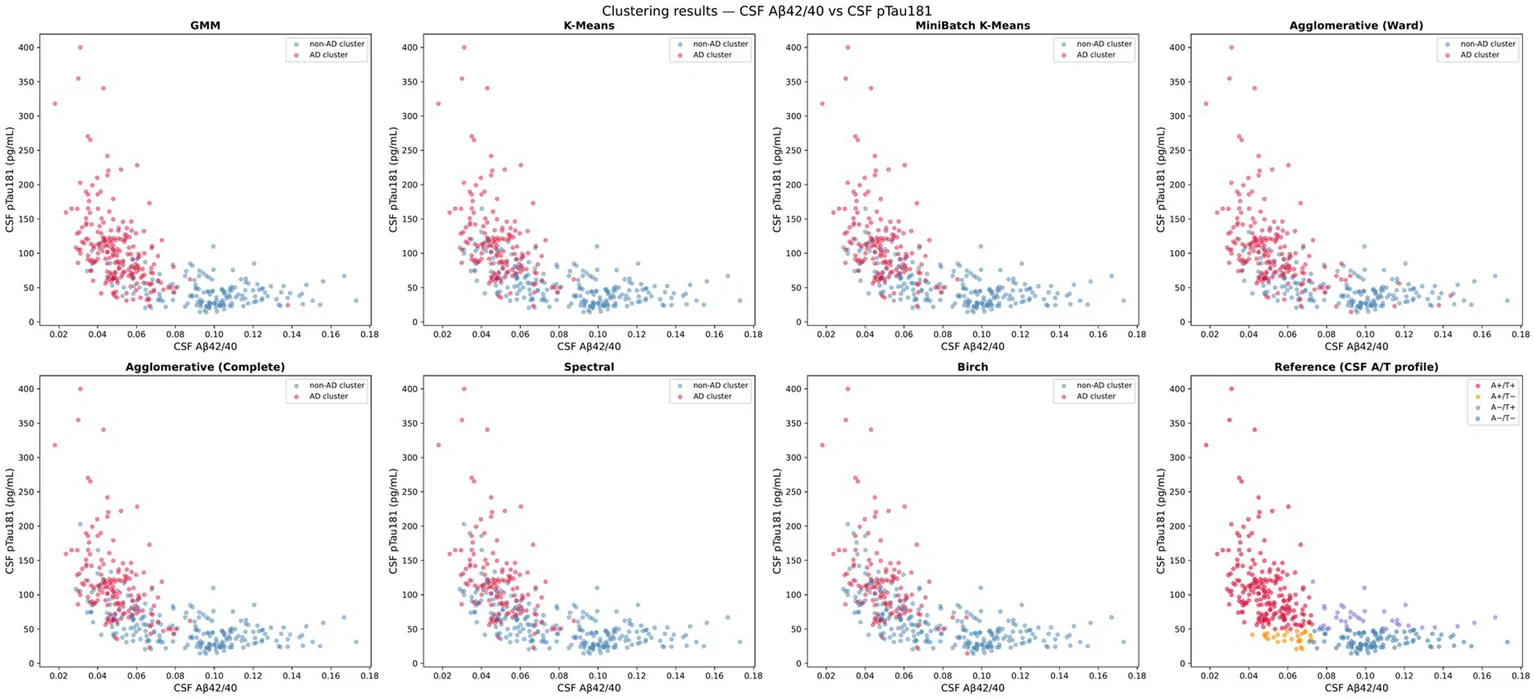
Clustering results projected onto CSF Aβ42/40 vs pTau181 axes. Each panel shows the two. clusters identified by a different algorithm, with the reference CSF A/T profile shown in the bottom-right panel.

### 3.2 Cutoff definition

Clustering-based analyses led to the definition of three cutoffs for each of the seven plasma biomarkers, for each clustering algorithm (Table 3). The clustering analyses included 379 patients for all examined BBMs and ratios while for performance testing patients with outlier values were again included (i.e., whole cohort of 434 patients).

**Table 3:** Clustering-based cutoff values for each plasma biomarker across all seven algorithms.

Biomarker	Cutoff	GMM	K-Means MB	K-Means	Ward	Complete	Spectral	Birch
A*β*42/40	Best	0.0822	0.0817	0.0819	0.0829	0.0819	0.0783	0.0785
	95% Sens	0.0893	0.0885	0.0887	0.0911	0.0883	0.089	0.0907
	95% Spec	0.067	0.0642	0.0642	0.064	0.0642	0.0627	0.0626
pTau217	Best	0.2271	0.3958	0.384	0.3171	0.3857	0.4549	0.4913
	95% Sens	0.1511	0.3482	0.3423	0.1757	0.3561	0.4481	0.4145
	95% Spec	0.19	0.3612	0.3461	0.2875	0.3447	0.4163	0.4889
pTau181	Best	2.0816	2.293	2.2789	2.0981	2.2953	2.4008	2.4605
	95% Sens	1.3395	1.9423	1.9196	1.6271	1.8228	2.0741	2.1466
	95% Spec	2.303	2.474	2.4594	2.1929	2.5546	2.6293	2.6936
NfL	Best	19.0446	20.7553	20.7988	20.291	19.9336	21.0317	21.5913
	95% Sens	11.311	14.1523	14.1209	12.6225	13.4115	14.5443	14.752
	95% Spec	34.179	33.5962	33.4104	32.8983	34.5967	34.0814	33.9628
pTau217/A*β*40	Best	0.0007	0.0013	0.0013	0.0011	0.0013	0.0015	0.0016
	95% Sens	0.0005	0.0011	0.0011	0.0006	0.0012	0.0014	0.0013
	95% Spec	0.0006	0.0012	0.0012	0.001	0.0011	0.0014	0.0017
pTau217/A*β*42	Best	0.009	0.017	0.0166	0.0141	0.0164	0.0198	0.0217
	95% Sens	0.0058	0.0141	0.0137	0.0064	0.0148	0.0173	0.0159
	95% Spec	0.0074	0.0155	0.0151	0.013	0.0145	0.0187	0.0226
pTau181/A*β*40	Best	0.0069	0.0076	0.0076	0.0068	0.0076	0.0079	0.0081
	95% Sens	0.0045	0.0064	0.0063	0.0053	0.0061	0.0067	0.007
	95% Spec	0.0082	0.0085	0.0085	0.0069	0.0086	0.009	0.0087

Best: intersection of fitted Gaussian curves; 95% Sens: maximizing sensitivity at 95%; 95% Spec: maximizing specificity at 95%.

The diagnostic accuracy of each single-biomarker cutoff was evaluated across 10 non-overlapping stratified subsets of the study population, generated to preserve the distribution of diagnostic groups within each subset. Accuracy metrics were calculated separately in each subset and subsequently summarized to assess the robustness and stability of cutoff performance across different population samples and reported as mean ± SD (Table 4). Accuracy was defined as the proportion of correctly classified patients (true positives + true negatives) with respect to the CSF A^+^/T^+^ reference profile. The highest single-biomarker accuracies were achieved by GMM-derived cutoffs for pTau217/A β 40 (0.873 ± 0.046), pTau217 (0.862 ± 0.057) and pTau217/A β 42 (0.860 ± 0.055). For K-Means, MiniBatch K-Means and Agglomerative Complete, pTau217/A β 40 yielded the best accuracy (0.798 ± 0.070). For Ward, pTau217 achieved the highest accuracy (0.823 ± 0.069), followed by pTau217/A β 40 (0.809 ± 0.070). For Spectral, pTau217 and pTau217/A β 40 were tied (0.775 ± 0.085 and 0.775 ± 0.069, respectively), while Birch achieved 0.765 ± 0.065 with pTau217/A β 40.

**Table 4:** Diagnostic accuracy (mean ± SD) of each single-biomarker clustering-based best cutoff across all seven algorithms, computed on 10 non-overlapping stratified subsets of the cohort.

Biomarker	GMM	K-Means MB	K-Means	Ward	Complete	Spectral	Birch
A*β*42/40	0.693 ± 0.047	0.694 ± 0.053	0.694 ± 0.048	0.691 ± 0.048	0.694 ± 0.048	0.673 ± 0.069	0.668 ± 0.069
pTau217	0.862 ± 0.057	0.791 ± 0.077	0.796 ± 0.078	0.823 ± 0.069	0.796 ± 0.078	0.775 ± 0.085	0.756 ± 0.080
pTau181	0.791 ± 0.045	0.761 ± 0.052	0.770 ± 0.043	0.791 ± 0.043	0.761 ± 0.052	0.745 ± 0.068	0.745 ± 0.071
NfL	0.645 ± 0.044	0.636 ± 0.054	0.634 ± 0.056	0.639 ± 0.054	0.641 ± 0.050	0.643 ± 0.060	0.636 ± 0.059
pTau217/A*β*40	0.873 ± 0.046	0.798 ± 0.070	0.798 ± 0.070	0.809 ± 0.070	0.798 ± 0.070	0.775 ± 0.069	0.765 ± 0.065
pTau217/A*β*42	0.860 ± 0.055	0.786 ± 0.069	0.789 ± 0.071	0.800 ± 0.069	0.791 ± 0.073	0.768 ± 0.073	0.756 ± 0.063
pTau181/A*β*40	0.795 ± 0.048	0.756 ± 0.051	0.756 ± 0.051	0.798 ± 0.045	0.756 ± 0.051	0.738 ± 0.058	0.733 ± 0.044

Accuracy is defined as (true positives + true negatives) / total, where the reference standard is the CSF A^+^/T^+^ profile.

### 3.3 Cutoff combinations

The prevalence of an A^+^/T^+^ CSF profile in subgroups defined by each possible combination (from 1 to 7, n=127 per algorithm) of clustering-based best cutoffs in the whole dataset, including outlier values, is shown in Supplementary Tables, ranked by prevalence of AD CSF profile. Also, we evaluated the prevalence of an A^+^/T^+^ CSF profile in subgroups of patients defined by BBMs cutoffs positivity for each combination and the prevalence of non A^+^/T^+^ CSF profile in subgroups of patients defined by BBMs cutoffs negativity for each combination (Table 5). Also in this analysis, for performance testing patients with outlier values were included.

**Table 5:** Top cutoff combinations for each clustering algorithm.

Algorithm	Combination	A+/T+ in BBM+	n BBM+	non-A+/T+ in BBM-	n BBM-
GMM	pTau181 + pTau217/A*β*40	0.923	194	0.873	166
	pTau181 + pTau217/A*β*40 + pTau181/A*β*40	0.923	182	0.869	160
	pTau217 + pTau181 + pTau217/A*β*40 + pTau181/A*β*40	0.923	181	0.874	159
	A*β*42/40 + pTau181 + pTau217/A*β*40	0.919	161	0.949	99
	A*β*42/40 + pTau181 + pTau217/A*β*40 + pTau217/A*β*42	0.919	161	0.949	99
	A*β*42/40 + pTau217 + pTau181 + pTau217/A*β*40	0.919	161	0.949	99
K-Means	pTau181 + NfL + pTau217/A*β*40 + pTau217/A*β*42 + pTau181/A*β*40	0.946	111	0.762	143
	pTau181 + NfL + pTau217/A*β*42 + pTau181/A*β*40	0.946	111	0.753	146
	pTau217 + pTau181 + NfL + pTau217/A*β*40 + pTau181/A*β*40	0.946	112	0.767	146
	A*β*42/40 + pTau217 + pTau181 + NfL + pTau217/A*β*40 + pTau181/A*β*40	0.937	95	0.904	83
	A*β*42/40 + pTau217 + pTau181 + NfL + pTau217/A*β*40 + pTau217/A*β*42 + pTau181/A*β*40	0.937	95	0.904	83
	A*β*42/40 + pTau181 + NfL + pTau217/A*β*40 + pTau217/A*β*42 + pTau181/A*β*40	0.937	95	0.904	83
MB K-Means	pTau181 + NfL + pTau217/A*β*40 + pTau217/A*β*42 +pTau181/A*β*40	0.946	112	0.773	141
	pTau181 + NfL + pTau217/A*β*42 + pTau181/A*β*40	0.946	112	0.764	144
	pTau217 + pTau181 + NfL + pTau217/A*β*40 + pTau181/A*β*40	0.946	112	0.778	144
	A*β*42/40 + pTau217 + pTau181 + NfL + pTau217/A*β*40 + pTau181/A*β*40	0.936	94	0.902	82
	A*β*42/40 + pTau217 + pTau181 + NfL + pTau217/A*β*40 + pTau217/A*β*42 + pTau181/A*β*40	0.936	94	0.902	82
	A*β*42/40 + pTau181 + NfL + pTau217/A*β*40 + pTau217/A*β*42 + pTau181/A*β*40	0.936	94	0.902	82
Ward	pTau217 + pTau181 + NfL + pTau217/A*β*40 + pTau181/A*β*40	0.939	131	0.812	128
	pTau217 + pTau181 + NfL + pTau217/A*β*42	0.939	131	0.801	136
	pTau217 + pTau181 + NfL + pTau217/A*β*40	0.939	131	0.799	139
	A*β*42/40 + pTau217 + NfL + pTau181/A*β*40	0.93	115	0.922	77
	A*β*42/40 + pTau217 + NfL + pTau217/A*β*42 + pTau181/A*β*40	0.929	113	0.922	77
	A*β*42/40 + pTau217 + pTau181 + NfL + pTau181/A*β*40	0.938	113	0.921	76
Complete	pTau181 + NfL + pTau217/A*β*40 + pTau217/A*β*42 + pTau181/A*β*40	0.942	120	0.754	138
	pTau181 + NfL + pTau217/A*β*42 + pTau181/A*β*40	0.942	120	0.75	140
	pTau217 + pTau181 + NfL + pTau217/A*β*40 + pTau181/A*β*40	0.942	120	0.759	141
	A*β*42/40 + pTau217 + pTau181 + NfL + pTau217/A*β*42 + pTau181/A*β*40	0.931	102	0.899	79
	A*β*42/40 + pTau217 + pTau181 + NfL + pTau217/A*β*40 + pTau181/A*β*40	0.931	102	0.897	78
	A*β*42/40 + pTau181 + NfL + pTau217/A*β*40 + pTau181/A*β*40	0.931	102	0.897	78
Spectral	pTau217 + pTau181 + NfL + pTau217/A*β*40 + pTau181/A*β*40	0.952	104	0.742	159
	pTau181 + NfL + pTau217/A*β*42 + pTau181/A*β*40	0.952	104	0.736	159
	pTau181 + NfL + pTau217/A*β*40 + pTau181/A*β*40	0.952	104	0.744	160
	A*β*42/40 + pTau181 + NfL + pTau217/A*β*40 + pTau217/A*β*42 + pTau181/A*β*40	0.932	74	0.85	107
	A*β*42/40 + pTau181 + NfL + pTau217/A*β*40 + pTau181/A*β*40	0.932	74	0.85	107
	A*β*42/40 + pTau217 + pTau181 + NfL + pTau217/A*β*40 + pTau217/A*β*42 + pTau181/A*β*40	0.932	74	0.849	106
Birch	pTau181 + pTau217/A*β*42 + pTau181/A*β*40	0.949	118	0.725	229
	pTau181 + pTau217/A*β*40 + pTau217/A*β*42 + pTau181/A*β*40	0.949	117	0.731	227
	pTau217 + pTau181 + pTau217/A*β*42 + pTau181/A*β*40	0.948	115	0.73	226
	A*β*42/40 + NfL + pTau217/A*β*40 + pTau217/A*β*42 + pTau181/A*β*40	0.928	69	0.838	111
	A*β*42/40 + pTau181 + NfL + pTau217/A*β*40 + pTau217/A*β*42 + pTau181/A*β*40	0.925	67	0.838	111
	A*β*42/40 + NfL + pTau217/A*β*40 + pTau181/A*β*40	0.928	69	0.838	111

For each algorithm, the three combinations with highest prevalence of A^+^/T^+^ CSF profile in BBM+ subgroups and the three with highest prevalence of non-A^+^/T^+^ CSF profile in BBM- subgroups are shown. n BBM+ and n BBM- indicate the number of patients in each subgroup.

Combining different BBMs cutoffs significantly improved the reliability of plasma biomarkers as diagnostic tools for AD.

Across all calculation methods, the best cutoff combinations for AD diagnosis achieved AD CSF prevalences ranging from 0.923 (GMM: pTau181 + pTau217/A β 40) to 0.952 (Spectral: pTau217 + pTau181 + NfL + pTau217/A β 40 + pTau181/A β 40). K-Means and MiniBatch K-Means achieved comparable top performances (AD prevalence 0.946) with combinations including pTau181, NfL, pTau217/A β 40 and pTau181/A β 40. Birch reached 0.949 with pTau181 + pTau217/A β 42 + pTau181/A β 40.

The best standalone clustering-based cutoff for AD diagnosis was the one obtained for pTau217/A β 40, which achieved AD prevalences of 0.912 for K-Means, MiniBatch K-Means and Agglomerative Complete, 0.902 for Ward and 0.925 for Birch. Spectral clustering achieved the highest standalone performance with pTau217 (AD prevalence 0.927). For GMM, the best standalone cutoff was also the one obtained for pTau217/A β 40 with an AD prevalence of 0.877.

The best combination of two clustering-based cutoffs for AD diagnosis was pTau181 + pTau217/A β 40 for GMM, K-Means, MiniBatch K-Means and Ward (AD prevalence 0.923–0.935), while for Spectral clustering it was pTau217 + pTau217/A β 40 (AD prevalence 0.936) and for Agglomerative Complete and Birch it was pTau181 + pTau217/A β 42 (AD prevalence 0.934 and 0.945, respectively).

Regarding the ability to rule out a CSF AD profile, the best cutoffs for standalone markers or ratios were those obtained for pTau217/A β 40 and pTau217, which achieved not-AD CSF prevalences ranging from 0.672 to 0.869 depending on the algorithm. GMM achieved the highest not-AD prevalence (0.869 with pTau217/A β 40), while other algorithms ranged from 0.672 (Birch) to 0.755 (Ward).

The best combinations of cutoffs to rule out an AD CSF profile are also summarized in Table 5. Of interest, the best algorithm to define cutoffs and combinations for AD CSF exclusion was GMM, with a non-AD CSF prevalence in the best combination of 0.949, while the worst algorithm resulted to be Birch with the best combination of cutoffs defined by it achieving a prevalence of non-AD CSF of 0.838.

Although Spectral clustering achieved the highest A^+^/T^+^ prevalence in the BBM + subgroup for a single marker (0.927 with pTau217), its diagnostic accuracy was lower than GMM (0.775 ± 0.085 vs. 0.862 ± 0.057), reflecting a more restrictive cutoff. GMM-defined cutoffs achieved the best balance between identification and exclusion of an A^+^/T^+^ CSF profile, with pTau217/A β 40 reaching the highest single-marker diagnostic accuracy (0.873 ± 0.046), an A^+^/T^+^ prevalence of 0.877 in the BBM + subgroup and a non-A^+^/T^+^ prevalence of 0.869 in the BBM − subgroup.

### 3.4 Supervised classification

Eleven supervised classification models were evaluated through stratified 10-fold cross-validation with feature standardization performed within each fold (Table 6).

**Table 6:** Supervised classification results. Performance assessed through stratified 10-fold cross-validation with pipeline-based feature standardization.

Algorithm	Accuracy	Precision	Recall	F1
SVM (RBF)	0.8575 ± 0.0619	0.8608 ± 0.0617	0.8582 ± 0.0601	0.8564 ± 0.0619
SVM (Linear)	0.8598 ± 0.0654	0.8656 ± 0.0615	0.8626 ± 0.0618	0.8590 ± 0.0654
Logistic Regression	0.8527 ± 0.0510	0.8582 ± 0.0496	0.8549 ± 0.0473	0.8518 ± 0.0506
Random Forest	0.8804 ± 0.0343	0.8892 ± 0.0394	0.8757 ± 0.0327	0.8780 ± 0.0342
Gradient Boosting	0.8619 ± 0.0454	0.8709 ± 0.0488	0.8579 ± 0.0443	0.8592 ± 0.0454
AdaBoost	0.8779 ± 0.0355	0.8869 ± 0.0357	0.8721 ± 0.0372	0.8748 ± 0.0366
Extra Trees	0.8710 ± 0.0438	0.8813 ± 0.0485	0.8672 ± 0.0416	0.8686 ± 0.0439
K-NN	0.8642 ± 0.0626	0.8726 ± 0.0610	0.8606 ± 0.0634	0.8612 ± 0.0643
Naive Bayes	0.7931 ± 0.0608	0.8122 ± 0.0463	0.8024 ± 0.0546	0.7915 ± 0.0620
Decision Tree	0.8159 ± 0.0563	0.8184 ± 0.0553	0.8157 ± 0.0556	0.8141 ± 0.0563
MLP	0.8782 ± 0.0581	0.8842 ± 0.0603	0.8758 ± 0.0551	0.8767 ± 0.0578

F1 = F1-score (harmonic mean of precision and recall).

Random Forest, AdaBoost and Multilayer Perceptron achieved the highest accuracies ( ∼ 0.88).

Extra Trees, K-Nearest Neighbors, Gradient Boosting, SVM models with RBF and linear kernels, and Logistic Regression all achieved 10-fold cv. accuracies ranging from 0.85 to 0.87. Precision–recall curves for all classifiers are shown in Figure 2. Results of SVM with RBF kernel are graphically shown in Figure 3.

**Figure 2:**
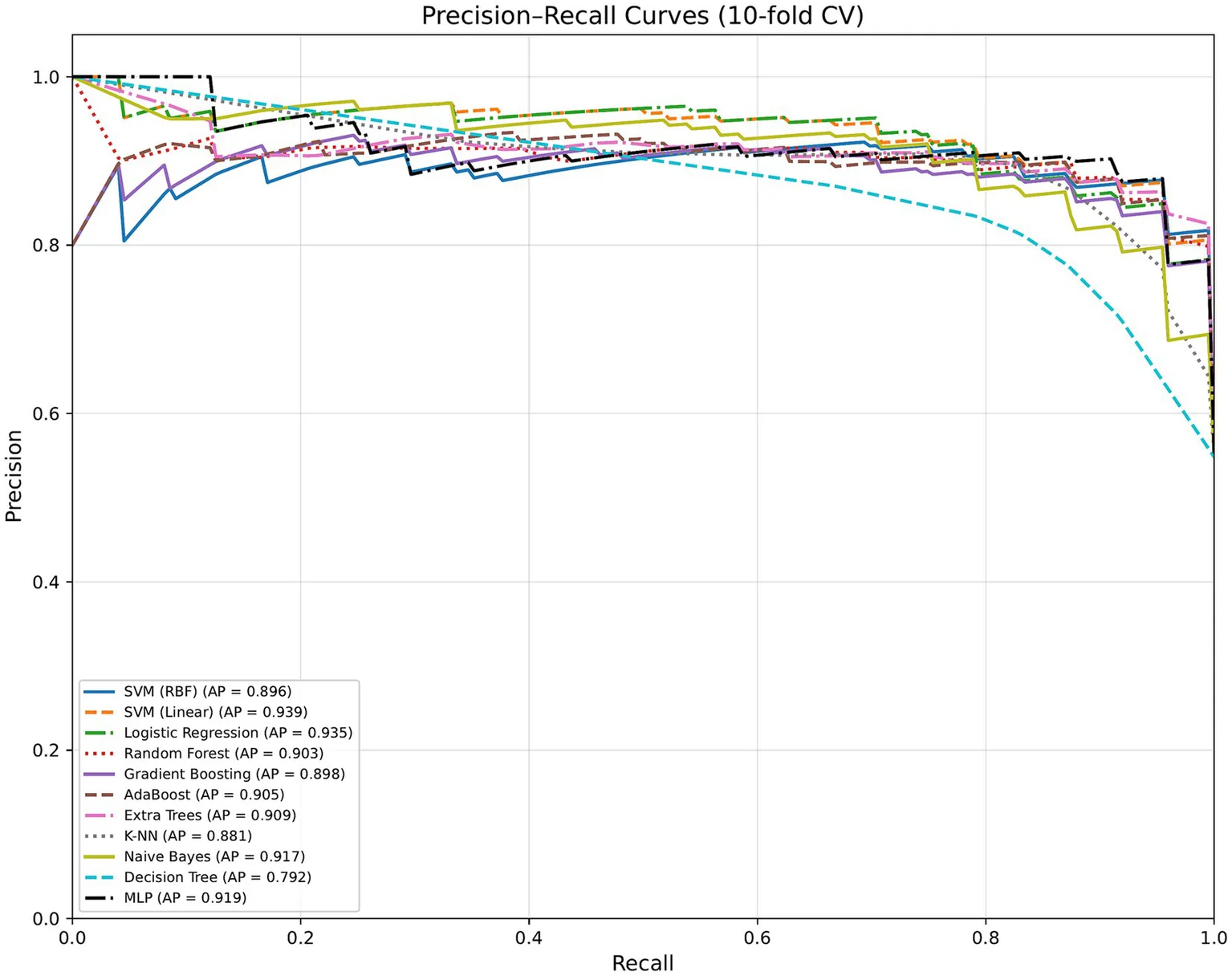
Mean precision–recall curves from stratified 10-fold cross-validation for all eleven supervised classification algorithms. AP = Average Precision (area under the precision–recall curve).

**Figure 3:**
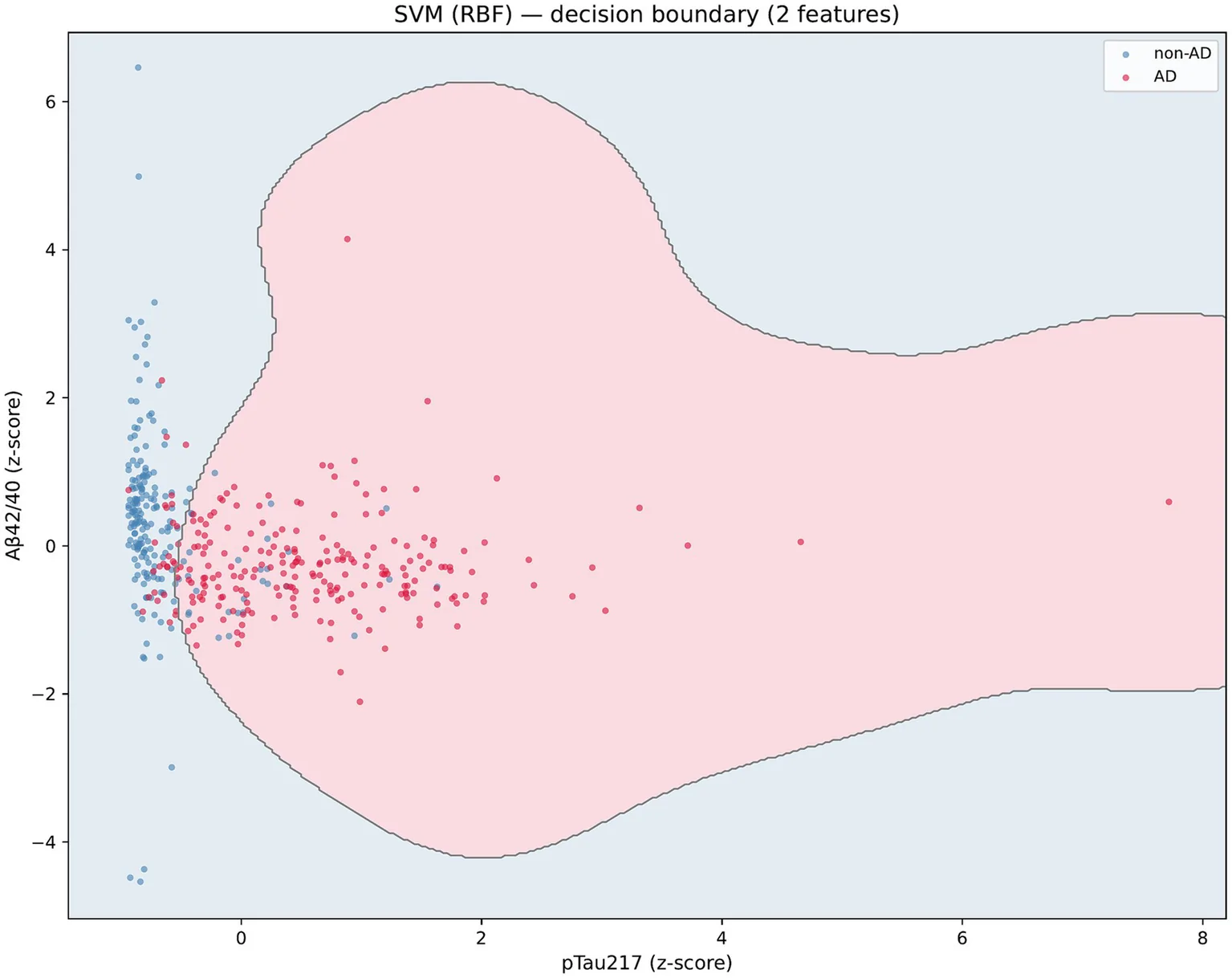
SVM (RBF kernel) decision boundary using two standardized plasma features (pTau217 and Aβ42/40).

Decision Tree and Gaussian Naive Bayes showed the lowest performances, likely due to overfitting (Decision Tree) and the strong feature independence assumption (Naive Bayes). Results of Decision Tree are shown in Figure 4.

**Figure 4:**
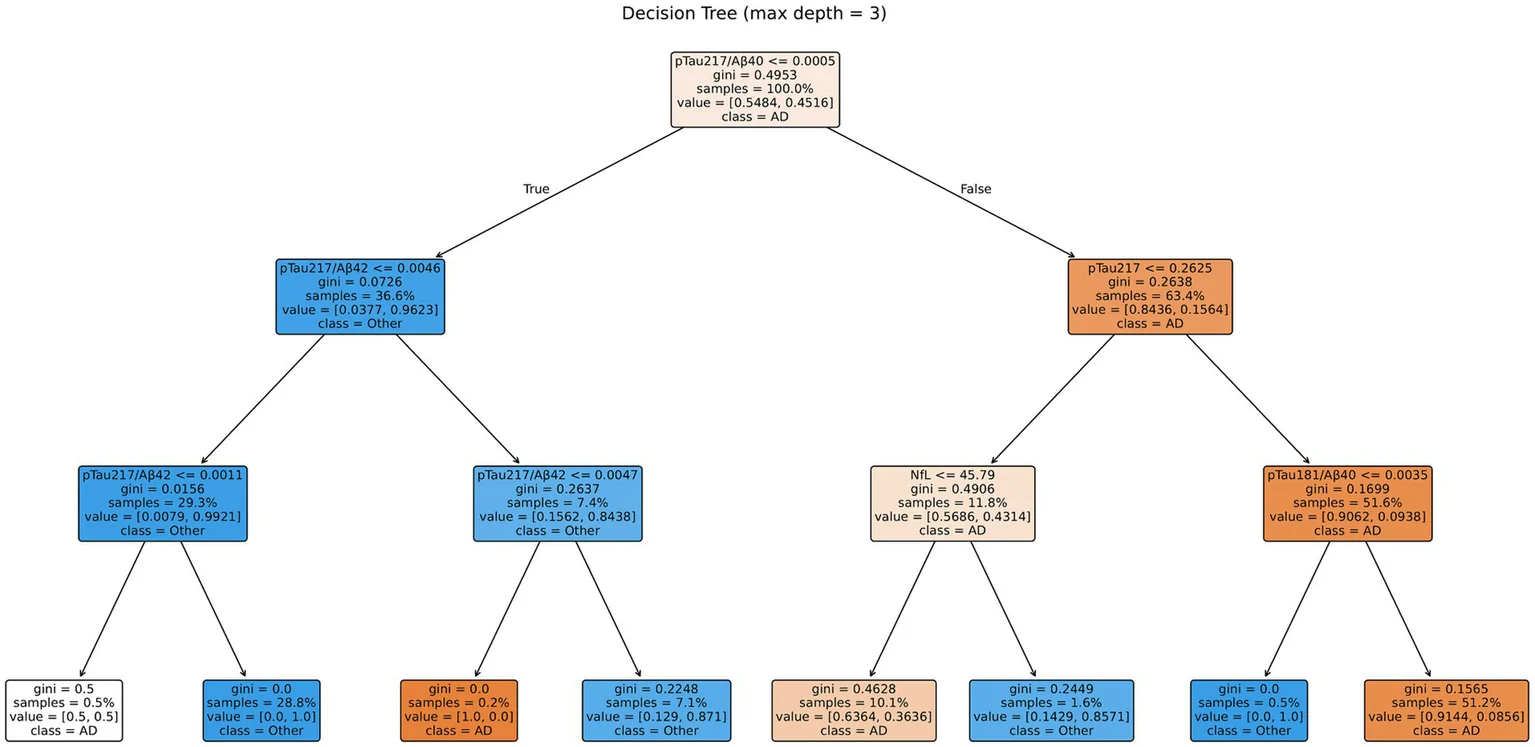
Decision Tree (max depth = 3) trained on plasma biomarker features for AD vs non-AD classification.

Of note, while cutoff-based AD prevalence (positive predictive value) reached values above 0.9 for the best combinations, supervised models achieved overall classification accuracies up to 0.88, representing a complementary metric that accounts for both true positives and true negatives. The ensemble tree-based methods (Random Forest, AdaBoost) also showed lower variance across folds, indicating more robust generalization.

## 4 Discussion and conclusion

Our study shows that in a real-world cohort, including patients with discordant A/T CSF profiles (i.e., A^+^/T^−^ and A^−^/T^+^), patients with AD in all its clinical stages as well as patients with other neurodegenerative diseases and controls, ML approaches can help identifying cutoffs for BBMs without CSF gold standard reference and enhance diagnostic accuracy of BBM combinations to rule in or rule out AD.

By evaluating multiple unsupervised approaches, we could establish a pipeline for generating reliable cutoffs for AD BBMs in the absence of CSF as a reference, demonstrating a role for such techniques in the field of biomarker validation and clinical implementation. By comparing seven different clustering algorithms, we demonstrated that the choice of algorithm impacts cutoff values and cluster structure. Despite this, the resulting cutoffs, while varying in absolute values, maintained broadly consistent rankings of diagnostic performance for each BBM across algorithms. Thus, our results prove that integrating information from different biomarkers via unsupervised clustering allows generating cutoffs with strong predictive value for an AD CSF profile without requiring CSF data for their definition. Since this study was exploratory in nature, we have chosen to define AD exclusively on the basis of CSF-confirmed A+/T+ status, as this CSF profile shows the highest concordance with Alzheimer’s disease neuropathology at *post mortem* examination (Wang et al., 2023).

In our cohort, the exploratory analysis on a combination approach for multiple BBMs cutoffs generated via unsupervised clustering, led to the identification of an underlying AD CSF profile with a concordance greater than 0.9, and the combination of multiple biomarkers’ best cutoffs consistently outperformed the use of a single biomarker’s best cutoff (e.g., AD prevalence from 0.877 for pTau217/A β 40 alone to 0.923–0.952 for multi-marker combinations across algorithms).

Ruling out an AD CSF profile proved to be more challenging. The best single-marker rule-out performance was achieved with GMM-derived pTau217/A β 40 cutoff (non-AD CSF prevalence 0.869), while other unsupervised algorithms-derived cutoffs ranged from 0.672 to 0.755. Also in ruling out an AD CSF profile, combinations of BBMs outperformed a single biomarker approach, with prevalences higher than 0.9 (0.949 for multiple GMM-defined cutoffs’ combinations). Among BBMs combinations achieving the best results, both in confirming and ruling out AD, there are many including 4 or fewer plasma biomarkers. These findings can be of great interest as our results could help in the definition of specific and cost-effective AD BBM panels for different settings according to the issue at stake (i.e., primary care, secondary care or tertiary care) (Schindler et al., 2024). Nonetheless, our combination analysis represents an exploratory result rather than an established outcome of this work. To prove the reliability of such a combined approach for AD BBMs, independent validation analyses on larger/diverse cohorts are needed.

As expected, in our cohort plasma pTau217 and its ratios with A β isoforms yielded the highest diagnostic accuracy for AD detection, consistently with a growing body of evidence identifying plasma pTau217 as one of the most accurate blood-based biomarkers for detecting AD pathology and predicting amyloid and tau positivity across different clinical settings (Palmqvist et al., 2025a).

Of all tested unsupervised approaches, GMM clustering-defined cutoffs showed a slightly better performance with the most balanced accuracy in predicting and excluding an A^+^/T^+^ CSF profile. A possible explanation for this result is that GMM clustering models data according to probabilistic distributions rather than geometrical distance, accommodating non-spherical cluster structures and probabilistic overlap, which are common features of heterogeneous, real-world biological data. This interpretation is in line with previous methodological studies suggesting that probabilistic clustering methods may better capture the complexity of biological datasets compared with distance-based approaches when biomarkers exhibit overlapping distributions (Fraley and Raftery, 2002; Scrucca et al., 2016).

Taken together, these findings can be of help in defining meaningful cutoffs for AD BBMs in settings where CSF or nuclear medicine biomarkers are not largely available. This could facilitate the broader implementation of BBMs as rule-in/rule-out tools to guide patient selection before confirmatory testing. However, our results were obtained in a selected population from a real-world tertiary care setting, and their generalizability to less specialized clinical environments will require further validation.

The comparison of 11 supervised classification algorithms, evaluated through rigorous stratified 10-fold cross-validation with proper data leakage prevention, demonstrated that supervised machine learning models can reliably identify an AD CSF profile from plasma biomarkers. Random Forest achieved the best accuracy (0.88), followed by AdaBoost and Multilayer Perceptron ( ∼ 0.88), while SVM, Logistic Regression, Extra Trees and other models achieved accuracies around 0.85–0.87. These results are consistent with previous machine-learning studies in AD showing that ensemble algorithms and nonlinear models often achieve the highest diagnostic performance when integrating plasma, imaging or CSF biomarkers (Song et al., 2021; Chang et al., 2021).

Of note, while supervised models optimize overall classification accuracy, cutoff-based BBMs combinations optimize the detection of an underlying AD CSF profile within defined subgroups (positive predictive value). Supervised models were not used for cutoff derivation because their training relied on CSF labels as the reference standard. Instead, cutoffs were derived through unsupervised clustering to evaluate whether reliable thresholds could be identified without gold-standard labelling. Supervised classification models were used solely to determine the extent to which combining multiple blood-based biomarkers improved the detection of a CSF AD profile. Furthermore, the evaluated classifiers spanned a spectrum from readily interpretable models (logistic regression, SVM with linear kernel, decision trees) to models with progressively lower intrinsic interpretability, such as random forest, gradient boosting, and multilayer perceptron. Accordingly, model comparison focused on predictive performance rather than variable importance (Strobl et al., 2008), as no single importance metric could be applied consistently across all classifiers (Hooker et al., 2021). Both unsupervised and supervised approaches confirm that plasma biomarkers carry sufficient information to reliably distinguish AD from non-AD CSF profiles, whether through explicit cutoff rules or through learned multivariate decision boundaries.

AD research has already proved to be a precursor in many developments regarding biology-driven approaches to diagnosis and treatment in neurodegenerative diseases. As we are moving towards an era in which disease-modifying therapies for AD will be largely available (Cummings J. L. et al., 2024), an early biological diagnosis will be more and more an issue in clinical practice. Our results show that ML approaches can enhance the possibility of achieving a reasonable degree of certainty using an assessment as minimally invasive as blood sampling. Beyond these findings, the present study is, to our knowledge, the first to comprehensively evaluate and benchmark multiple unsupervised clustering algorithms together with supervised classification models, establishing a reference for future methodological developments in this area.

In conclusion, as our understanding of neurodegenerative diseases’ biology deepens (Gaetani et al., 2025; Gaetani et al., 2020) and biologically driven disease-modifying therapies become available (Cummings J. L. et al., 2024), comprehensive, accessible, and broad molecular profiling will be critical to improve patient stratification and predict treatment response. Our results support the implementation of ML approaches to address these challenges by providing a practical, reproducible framework to quantify the contribution of ML in confirming and enhancing the diagnostic value of candidate biomarkers in neurodegenerative diseases.

## Data Availability

The raw data supporting the conclusions of this article will be made available by the authors, without undue reservation.
