# Supplementary material for: Application of unsupervised and supervised machine learning approaches to blood-based biomarkers of Alzheimer’s disease in a memory clinic cohort
Source: Front Aging Neurosci. 2026 Sep 25;18:1922577. doi: 10.3389/fnagi.2026.1922577 (PMC13649989; doi:10.3389/fnagi.2026.1922577)
Supplement: Supplementary file 1 [file Data_Sheet_1.docx]

1

**Supplementary material 1**

The following tables report all 127 cutoff combinations for each clustering algorithm, ranked by prevalence of A^+^/T^+^ CSF profile in BBM-positive subgroups.

Supplementary table 1 Cutoff combinations — GMM (all 127 combinations). Prevalence of A^+^/T^+^ CSF profile in BBM-positive subgroups and prevalence of non-A^+^/T^+^ CSF profile in BBM-negative subgroups.

| Combination | A+/T+ in BBM+ | n BBM+ | non-A+/T+ in BBM- | n BBM- |
| --- | --- | --- | --- | --- |
| pTau181 + pTau217/A*β*40 | 0.923 | 194 | 0.873 | 166 |
| pTau181 + pTau217/A*β*40 + pTau181/A*β*40 | 0.923 | 182 | 0.869 | 160 |
| pTau217 + pTau181 + pTau217/A*β*40 + pTau181/A*β*40 | 0.923 | 181 | 0.874 | 159 |
| pTau181 + pTau217/A*β*40 + pTau217/A*β*42 + pTau181/A*β*40 | 0.923 | 181 | 0.872 | 156 |
| pTau217 + pTau181 + pTau217/A*β*40 + pTau217/A*β*42 + pTau181/A*β*40 | 0.923 | 181 | 0.877 | 155 |
| pTau217 + pTau181 + pTau217/A*β*42 + pTau181/A*β*40 | 0.923 | 181 | 0.877 | 155 |
| pTau217 + pTau181 + pTau217/A*β*40 | 0.922 | 193 | 0.879 | 165 |
| pTau217 + pTau181 + pTau217/A*β*42 | 0.922 | 192 | 0.877 | 162 |
| pTau181 + pTau217/A*β*40 + pTau217/A*β*42 | 0.922 | 192 | 0.876 | 161 |
| pTau217 + pTau181 + pTau217/A*β*40 + pTau217/A*β*42 | 0.922 | 192 | 0.881 | 160 |
| A*β*42/40 + pTau181 + pTau217/A*β*40 + pTau181/A*β*40 | 0.921 | 152 | 0.948 | 97 |
| pTau217/A*β*40 + pTau217/A*β*42 + pTau181/A*β*40 | 0.921 | 191 | 0.866 | 172 |
| pTau217 + pTau217/A*β*40 + pTau217/A*β*42 + pTau181/A*β*40 | 0.921 | 190 | 0.871 | 170 |
| pTau217 + pTau217/A*β*42 + pTau181/A*β*40 | 0.921 | 190 | 0.871 | 170 |
| pTau217 + pTau217/A*β*40 + pTau181/A*β*40 | 0.921 | 190 | 0.868 | 174 |
| A*β*42/40 + pTau217 + pTau181 + pTau181/A*β*40 | 0.921 | 152 | 0.939 | 98 |
| A*β*42/40 + pTau217 + pTau181 + pTau217/A*β*40 + pTau217/A*β*42 +  pTau181/A*β*40 | 0.921 | 152 | 0.948 | 97 |
| A*β*42/40 + pTau217 + pTau181 + pTau217/A*β*42 + pTau181/A*β*40 | 0.921 | 152 | 0.948 | 97 |
| A*β*42/40 + pTau217 + pTau181 + pTau217/A*β*40 + pTau181/A*β*40 | 0.921 | 152 | 0.948 | 97 |
| A*β*42/40 + pTau181 + pTau217/A*β*40 + pTau217/A*β*42 + pTau181/A*β*40 | 0.921 | 152 | 0.948 | 97 |
| A*β*42/40 + pTau217 + pTau181 + pTau217/A*β*42 | 0.919 | 161 | 0.941 | 101 |
| pTau181 + NfL + pTau217/A*β*40 + pTau181/A*β*40 | 0.919 | 149 | 0.864 | 110 |
| A*β*42/40 + pTau181 + pTau217/A*β*40 + pTau217/A*β*42 | 0.919 | 161 | 0.949 | 99 |

| Combination | A+/T+ in BBM+ | n BBM+ | non-A+/T+ in BBM- | n BBM- |
| --- | --- | --- | --- | --- |
| A*β*42/40 + pTau217 + pTau181 + pTau217/A*β*40 | 0.919 | 161 | 0.949 | 99 |
| A*β*42/40 + pTau181 + pTau217/A*β*40 | 0.919 | 161 | 0.949 | 99 |
| A*β*42/40 + pTau217 + pTau181 | 0.919 | 161 | 0.931 | 102 |
| pTau181 + NfL + pTau217/A*β*40 + pTau217/A*β*42 + pTau181/A*β*40 | 0.919 | 149 | 0.861 | 108 |
| pTau217 + pTau181 + NfL + pTau217/A*β*42 + pTau181/A*β*40 | 0.919 | 149 | 0.861 | 108 |
| A*β*42/40 + pTau217 + pTau181 + pTau217/A*β*40 + pTau217/A*β*42 | 0.919 | 161 | 0.949 | 99 |
| pTau217 + pTau181 + NfL + pTau217/A*β*40 + pTau217/A*β*42 + pTau181/A*β*40 | 0.919 | 149 | 0.861 | 108 |
| A*β*42/40 + pTau217 + pTau181 + NfL + pTau217/A*β*40 + pTau217/A*β*42 +  pTau181/A*β*40 | 0.919 | 124 | 0.943 | 70 |
| pTau217 + pTau181 + NfL + pTau217/A*β*40 + pTau181/A*β*40 | 0.919 | 149 | 0.864 | 110 |
| A*β*42/40 + pTau217 + pTau181 + NfL + pTau217/A*β*40 + pTau181/A*β*40 | 0.919 | 124 | 0.943 | 70 |
| A*β*42/40 + pTau217 + pTau181 + NfL + pTau217/A*β*42 + pTau181/A*β*40 | 0.919 | 124 | 0.943 | 70 |
| A*β*42/40 + pTau217 + pTau181 + NfL + pTau181/A*β*40 | 0.919 | 124 | 0.93 | 71 |
| A*β*42/40 + pTau181 + NfL + pTau217/A*β*40 + pTau181/A*β*40 | 0.919 | 124 | 0.943 | 70 |
| A*β*42/40 + pTau181 + NfL + pTau217/A*β*40 + pTau217/A*β*42 + pTau181/A*β*40 | 0.919 | 124 | 0.943 | 70 |
| A*β*42/40 + pTau217/A*β*40 + pTau217/A*β*42 + pTau181/A*β*40 | 0.918 | 159 | 0.942 | 104 |
| A*β*42/40 + pTau217 + pTau217/A*β*42 + pTau181/A*β*40 | 0.918 | 158 | 0.942 | 103 |
| pTau217 + pTau181 + NfL + pTau217/A*β*40 | 0.918 | 158 | 0.87 | 115 |
| A*β*42/40 + pTau217 + pTau217/A*β*40 + pTau217/A*β*42 + pTau181/A*β*40 | 0.918 | 158 | 0.942 | 103 |
| A*β*42/40 + pTau217 + pTau217/A*β*40 + pTau181/A*β*40 | 0.918 | 158 | 0.942 | 103 |
| pTau217/A*β*40 + pTau181/A*β*40 | 0.918 | 194 | 0.864 | 176 |
| A*β*42/40 + pTau217 + pTau181/A*β*40 | 0.918 | 158 | 0.933 | 104 |
| pTau181 + pTau217/A*β*42 + pTau181/A*β*40 | 0.918 | 182 | 0.867 | 158 |
| pTau181 + NfL + pTau217/A*β*40 | 0.918 | 158 | 0.87 | 115 |
| pTau217 + pTau181 + pTau181/A*β*40 | 0.918 | 182 | 0.859 | 163 |

| Combination | A+/T+ in BBM+ | n BBM+ | non-A+/T+ in BBM- | n BBM- |
| --- | --- | --- | --- | --- |
| A*β*42/40 + pTau217/A*β*40 + pTau181/A*β*40 | 0.918 | 159 | 0.942 | 104 |
| pTau181 + pTau217/A*β*42 | 0.917 | 193 | 0.867 | 165 |
| pTau217 + pTau181 + NfL + pTau217/A*β*42 | 0.917 | 157 | 0.86 | 114 |
| pTau181 + NfL + pTau217/A*β*40 + pTau217/A*β*42 | 0.917 | 157 | 0.866 | 112 |
| pTau217 + pTau181 + NfL + pTau217/A*β*40 + pTau217/A*β*42 | 0.917 | 157 | 0.866 | 112 |
| A*β*42/40 + pTau181 + pTau181/A*β*40 | 0.916 | 154 | 0.865 | 111 |
| pTau217 + pTau181/A*β*40 | 0.916 | 191 | 0.854 | 178 |
| A*β*42/40 + pTau217 + pTau181 + NfL + pTau217/A*β*40 | 0.915 | 130 | 0.944 | 72 |
| A*β*42/40 + pTau217 + pTau181 + NfL | 0.915 | 130 | 0.92 | 75 |
| A*β*42/40 + pTau181 + NfL + pTau217/A*β*40 + pTau217/A*β*42 | 0.915 | 130 | 0.944 | 72 |
| A*β*42/40 + pTau217 + pTau181 + NfL + pTau217/A*β*42 | 0.915 | 130 | 0.932 | 74 |
| A*β*42/40 + pTau217 + pTau181 + NfL + pTau217/A*β*40 + pTau217/A*β*42 | 0.915 | 130 | 0.944 | 72 |
| A*β*42/40 + pTau181 + NfL + pTau217/A*β*40 | 0.915 | 130 | 0.944 | 72 |
| A*β*42/40 + pTau181 + pTau217/A*β*42 + pTau181/A*β*40 | 0.915 | 153 | 0.939 | 99 |
| A*β*42/40 + pTau181 + pTau217/A*β*42 | 0.914 | 162 | 0.932 | 103 |
| NfL + pTau217/A*β*40 + pTau181/A*β*40 | 0.914 | 152 | 0.857 | 112 |
| pTau217 + NfL + pTau217/A*β*40 + pTau181/A*β*40 | 0.914 | 152 | 0.857 | 112 |
| NfL + pTau217/A*β*40 + pTau217/A*β*42 + pTau181/A*β*40 | 0.914 | 152 | 0.855 | 110 |
| pTau217 + NfL + pTau217/A*β*40 + pTau217/A*β*42 + pTau181/A*β*40 | 0.914 | 152 | 0.855 | 110 |
| pTau217 + NfL + pTau217/A*β*42 + pTau181/A*β*40 | 0.914 | 152 | 0.855 | 110 |
| A*β*42/40 + NfL + pTau217/A*β*40 + pTau181/A*β*40 | 0.913 | 126 | 0.944 | 71 |
| pTau181 + NfL + pTau217/A*β*42 + pTau181/A*β*40 | 0.913 | 150 | 0.862 | 109 |
| A*β*42/40 + pTau217 + NfL + pTau181/A*β*40 | 0.913 | 126 | 0.931 | 72 |
| pTau217 + pTau181 + NfL + pTau181/A*β*40 | 0.913 | 150 | 0.842 | 114 |
| A*β*42/40 + NfL + pTau217/A*β*40 + pTau217/A*β*42 + pTau181/A*β*40 | 0.913 | 126 | 0.944 | 71 |
| pTau217 + pTau181 | 0.913 | 195 | 0.855 | 172 |
| A*β*42/40 + pTau217 + NfL + pTau217/A*β*42 + pTau181/A*β*40 | 0.913 | 126 | 0.944 | 71 |
|  |  |  |  | |

| Combination | A+/T+ in BBM+ | n BBM+ | non-A+/T+ in BBM- | n BBM- |
| --- | --- | --- | --- | --- |
| A*β*42/40 + pTau217 + NfL + pTau217/A*β*40 + pTau181/A*β*40 | 0.913 | 126 | 0.944 | 71 |
| A*β*42/40 + pTau217 + NfL + pTau217/A*β*40 + pTau217/A*β*42 + pTau181/A*β*40 | 0.913 | 126 | 0.944 | 71 |
| pTau217/A*β*42 + pTau181/A*β*40 | 0.912 | 193 | 0.857 | 175 |
| A*β*42/40 + pTau181 + NfL + pTau217/A*β*42 + pTau181/A*β*40 | 0.912 | 125 | 0.944 | 71 |
| A*β*42/40 + pTau181 + NfL + pTau181/A*β*40 | 0.912 | 125 | 0.883 | 77 |
| pTau181 + NfL + pTau217/A*β*42 | 0.911 | 158 | 0.861 | 115 |
| A*β*42/40 + pTau181 + NfL + pTau217/A*β*42 | 0.908 | 131 | 0.933 | 75 |
| NfL + pTau217/A*β*42 + pTau181/A*β*40 | 0.908 | 153 | 0.856 | 111 |
| pTau217 + NfL + pTau181/A*β*40 | 0.908 | 153 | 0.836 | 116 |
| A*β*42/40 + pTau217/A*β*42 + pTau181/A*β*40 | 0.907 | 161 | 0.925 | 107 |
| A*β*42/40 + NfL + pTau217/A*β*42 + pTau181/A*β*40 | 0.906 | 127 | 0.944 | 72 |
| pTau217 + pTau181 + NfL | 0.906 | 160 | 0.836 | 122 |
| A*β*42/40 + NfL + pTau181/A*β*40 | 0.898 | 128 | 0.885 | 78 |
| pTau181 + NfL + pTau181/A*β*40 | 0.895 | 153 | 0.774 | 133 |
| A*β*42/40 + pTau217 + NfL | 0.893 | 140 | 0.914 | 81 |
| A*β*42/40 + pTau217 + NfL + pTau217/A*β*40 | 0.892 | 139 | 0.948 | 77 |
| A*β*42/40 + NfL + pTau217/A*β*40 + pTau217/A*β*42 | 0.892 | 139 | 0.948 | 77 |
| A*β*42/40 + pTau217 + NfL + pTau217/A*β*42 | 0.892 | 139 | 0.925 | 80 |
| A*β*42/40 + pTau217 + NfL + pTau217/A*β*40 + pTau217/A*β*42 | 0.892 | 139 | 0.948 | 77 |
| A*β*42/40 + NfL + pTau217/A*β*40 | 0.892 | 139 | 0.948 | 77 |
| pTau217 + NfL + pTau217/A*β*40 | 0.891 | 175 | 0.861 | 122 |
| A*β*42/40 + pTau181/A*β*40 | 0.891 | 165 | 0.843 | 121 |
| NfL + pTau217/A*β*40 | 0.891 | 175 | 0.861 | 122 |
| pTau217 + NfL + pTau217/A*β*42 | 0.89 | 173 | 0.844 | 122 |
| NfL + pTau217/A*β*40 + pTau217/A*β*42 | 0.89 | 173 | 0.857 | 119 |
| pTau217 + NfL + pTau217/A*β*40 + pTau217/A*β*42 | 0.89 | 173 | 0.857 | 119 |
| NfL + pTau181/A*β*40 | 0.885 | 157 | 0.754 | 138 |
| pTau181 + pTau181/A*β*40 | 0.885 | 191 | 0.772 | 197 |
| pTau217 + pTau217/A*β*42 | 0.884 | 232 | 0.865 | 185 |
| pTau217 + pTau217/A*β*40 + pTau217/A*β*42 | 0.884 | 232 | 0.874 | 182 |
| A*β*42/40 + pTau217 | 0.883 | 188 | 0.922 | 115 |
| pTau217 + NfL | 0.882 | 178 | 0.823 | 130 |
| A*β*42/40 + pTau217 + pTau217/A*β*42 | 0.882 | 187 | 0.93 | 114 |
| A*β*42/40 + pTau217 + pTau217/A*β*40 | 0.882 | 187 | 0.946 | 111 |

| Combination | A+/T+ in BBM+ | n BBM+ | non-A+/T+ in BBM- | n BBM- |
| --- | --- | --- | --- | --- |
| pTau217/A*β*40 + pTau217/A*β*42 | 0.882 | 237 | 0.87 | 185 |
| A*β*42/40 + pTau217 + pTau217/A*β*40 + pTau217/A*β*42 | 0.882 | 187 | 0.946 | 111 |
| NfL + pTau217/A*β*42 | 0.881 | 176 | 0.846 | 123 |
| pTau217 + pTau217/A*β*40 | 0.881 | 235 | 0.872 | 188 |
| A*β*42/40 + pTau217/A*β*40 | 0.88 | 191 | 0.947 | 113 |
| A*β*42/40 + NfL + pTau217/A*β*42 | 0.88 | 142 | 0.926 | 81 |
| A*β*42/40 + pTau217/A*β*40 + pTau217/A*β*42 | 0.88 | 191 | 0.947 | 113 |
| A*β*42/40 + pTau181 | 0.878 | 172 | 0.839 | 118 |
| pTau217/A*β*40 | 0.877 | 243 | 0.869 | 191 |
| pTau217 | 0.874 | 238 | 0.847 | 196 |
| pTau217/A*β*42 | 0.864 | 243 | 0.853 | 191 |
| A*β*42/40 + pTau181 + NfL | 0.863 | 139 | 0.855 | 83 |
| A*β*42/40 + pTau217/A*β*42 | 0.858 | 197 | 0.916 | 119 |
| pTau181/A*β*40 | 0.856 | 209 | 0.738 | 225 |
| pTau181 | 0.836 | 219 | 0.744 | 215 |
| pTau181 + NfL | 0.835 | 176 | 0.741 | 147 |
| A*β*42/40 + NfL | 0.756 | 172 | 0.817 | 93 |
| A*β*42/40 | 0.695 | 269 | 0.691 | 165 |
| NfL | 0.672 | 244 | 0.611 | 190 |

Supplementary table 2 Cutoff combinations — K-Means (all 127 combinations). Prevalence of A^+^/T^+^ CSF profile in BBM-positive subgroups and prevalence of non-A^+^/T^+^ CSF profile in BBM-negative subgroups.

| Combination | A+/T+ in BBM+ | n BBM+ | non-A+/T+ in BBM- | n BBM- |
| --- | --- | --- | --- | --- |
| pTau181 + NfL + pTau217/A*β*40 + pTau217/A*β*42 + pTau181/A*β*40 | 0.946 | 111 | 0.762 | 143 |
| pTau181 + NfL + pTau217/A*β*42 + pTau181/A*β*40 | 0.946 | 111 | 0.753 | 146 |
| pTau217 + pTau181 + NfL + pTau217/A*β*40 + pTau181/A*β*40 | 0.946 | 112 | 0.767 | 146 |
| pTau217 + pTau181 + NfL + pTau217/A*β*40 + pTau217/A*β*42 + pTau181/A*β*40 | 0.946 | 111 | 0.762 | 143 |
| pTau181 + NfL + pTau217/A*β*40 + pTau181/A*β*40 | 0.946 | 112 | 0.769 | 147 |
| pTau217 + pTau181 + NfL + pTau217/A*β*42 + pTau181/A*β*40 | 0.946 | 111 | 0.759 | 145 |
| pTau181 + NfL + pTau217/A*β*42 | 0.94 | 116 | 0.748 | 151 |
| pTau217 + pTau181 + NfL + pTau217/A*β*40 | 0.94 | 117 | 0.758 | 153 |
| pTau217 + pTau181 + NfL + pTau217/A*β*42 | 0.94 | 116 | 0.753 | 150 |

| pTau181 + NfL + pTau217/A*β*40 | 0.94 | 117 | 0.76 | 154 |
| --- | --- | --- | --- | --- |
| pTau217 + pTau181 + NfL + pTau217/A*β*40 + pTau217/A*β*42 | 0.94 | 116 | 0.757 | 148 |
| pTau181 + NfL + pTau217/A*β*40 + pTau217/A*β*42 | 0.94 | 116 | 0.757 | 148 |
| A*β*42/40 + pTau217 + pTau181 + NfL + pTau217/A*β*40 | 0.939 | 98 | 0.897 | 87 |
| A*β*42/40 + pTau217 + pTau181 + NfL + pTau217/A*β*42 | 0.939 | 98 | 0.888 | 89 |
| A*β*42/40 + pTau181 + NfL + pTau217/A*β*40 | 0.939 | 98 | 0.897 | 87 |
| A*β*42/40 + pTau217 + pTau181 + NfL + pTau217/A*β*40 + pTau217/A*β*42 | 0.939 | 98 | 0.897 | 87 |
| pTau217 + pTau181 + NfL + pTau181/A*β*40 | 0.939 | 115 | 0.753 | 150 |
| A*β*42/40 + pTau181 + NfL + pTau217/A*β*42 | 0.939 | 98 | 0.878 | 90 |
| A*β*42/40 + pTau181 + NfL + pTau217/A*β*40 + pTau217/A*β*42 | 0.939 | 98 | 0.897 | 87 |
| A*β*42/40 + pTau217 + pTau181 + NfL + pTau181/A*β*40 | 0.938 | 96 | 0.884 | 86 |
| A*β*42/40 + pTau181 + NfL + pTau217/A*β*40 + pTau217/A*β*42 + pTau181/A*β*40 | 0.937 | 95 | 0.904 | 83 |
| pTau181 + pTau217/A*β*40 + pTau181/A*β*40 | 0.937 | 143 | 0.762 | 210 |
| pTau181 + pTau217/A*β*42 + pTau181/A*β*40 | 0.937 | 143 | 0.751 | 209 |
| pTau217 + pTau181 + pTau217/A*β*40 + pTau181/A*β*40 | 0.937 | 142 | 0.761 | 209 |
| pTau181 + pTau217/A*β*40 + pTau217/A*β*42 + pTau181/A*β*40 | 0.937 | 142 | 0.757 | 206 |
| A*β*42/40 + pTau181 + NfL + pTau217/A*β*40 + pTau181/A*β*40 | 0.937 | 95 | 0.904 | 83 |
| A*β*42/40 + pTau217 + pTau181 + NfL + pTau217/A*β*40 + pTau181/A*β*40 | 0.937 | 95 | 0.904 | 83 |
| A*β*42/40 + pTau217 + pTau181 + NfL + pTau217/A*β*42 + pTau181/A*β*40 | 0.937 | 95 | 0.894 | 85 |
| A*β*42/40 + pTau181 + NfL + pTau217/A*β*42 + pTau181/A*β*40 | 0.937 | 95 | 0.884 | 86 |
| A*β*42/40 + pTau217 + pTau181 + NfL + pTau217/A*β*40 + pTau217/A*β*42 +  pTau181/A*β*40 | 0.937 | 95 | 0.904 | 83 |
| pTau217 + pTau181 + pTau217/A*β*42 + pTau181/A*β*40 | 0.937 | 142 | 0.755 | 208 |
| pTau217 + pTau181 + pTau217/A*β*40 + pTau217/A*β*42 + pTau181/A*β*40 | 0.936 | 141 | 0.757 | 206 |

| Combination | A+/T+ in BBM+ | n BBM+ | non-A+/T+ in BBM- | n BBM- |
| --- | --- | --- | --- | --- |
| pTau181 + pTau217/A*β*40 + pTau217/A*β*42 | 0.933 | 149 | 0.755 | 212 |
| pTau217 + pTau181 + pTau217/A*β*42 | 0.933 | 149 | 0.752 | 214 |
| pTau217 + NfL + pTau217/A*β*40 + pTau181/A*β*40 | 0.933 | 119 | 0.757 | 148 |
| NfL + pTau217/A*β*40 + pTau181/A*β*40 | 0.933 | 119 | 0.758 | 149 |
| pTau181 + pTau217/A*β*42 | 0.933 | 150 | 0.749 | 215 |
| pTau181 + pTau217/A*β*40 | 0.933 | 150 | 0.757 | 218 |
| pTau217 + pTau181 + pTau217/A*β*40 | 0.933 | 149 | 0.756 | 217 |
| pTau217 + pTau181 + pTau217/A*β*40 + pTau217/A*β*42 | 0.932 | 148 | 0.755 | 212 |
| pTau217 + NfL + pTau217/A*β*42 + pTau181/A*β*40 | 0.932 | 118 | 0.748 | 147 |
| pTau217 + NfL + pTau217/A*β*40 + pTau217/A*β*42 + pTau181/A*β*40 | 0.932 | 118 | 0.752 | 145 |
| pTau217 + pTau181 + pTau181/A*β*40 | 0.932 | 146 | 0.751 | 213 |
| NfL + pTau217/A*β*42 + pTau181/A*β*40 | 0.932 | 118 | 0.743 | 148 |
| NfL + pTau217/A*β*40 + pTau217/A*β*42 + pTau181/A*β*40 | 0.932 | 118 | 0.752 | 145 |
| A*β*42/40 + pTau217 + pTau181 + NfL | 0.93 | 100 | 0.878 | 90 |
| A*β*42/40 + pTau181 + pTau217/A*β*42 | 0.93 | 128 | 0.846 | 123 |
| A*β*42/40 + pTau217 + pTau181 + pTau217/A*β*42 | 0.929 | 127 | 0.852 | 122 |
| A*β*42/40 + pTau181 + pTau217/A*β*40 + pTau217/A*β*42 | 0.929 | 127 | 0.858 | 120 |
| A*β*42/40 + pTau217 + pTau181 + pTau217/A*β*40 + pTau217/A*β*42 | 0.929 | 126 | 0.858 | 120 |
| A*β*42/40 + pTau217 + pTau181 + pTau217/A*β*40 | 0.929 | 126 | 0.858 | 120 |
| A*β*42/40 + pTau181 + pTau217/A*β*40 | 0.929 | 127 | 0.858 | 120 |
| pTau217/A*β*40 + pTau181/A*β*40 | 0.929 | 154 | 0.741 | 224 |
| pTau217 + pTau217/A*β*42 + pTau181/A*β*40 | 0.928 | 152 | 0.736 | 220 |
| pTau217/A*β*40 + pTau217/A*β*42 + pTau181/A*β*40 | 0.928 | 153 | 0.736 | 220 |
| A*β*42/40 + pTau217 + pTau181 + pTau181/A*β*40 | 0.928 | 125 | 0.849 | 119 |
| A*β*42/40 + pTau181 + pTau217/A*β*42 + pTau181/A*β*40 | 0.928 | 125 | 0.849 | 119 |
| pTau217 + pTau217/A*β*40 + pTau181/A*β*40 | 0.928 | 152 | 0.742 | 221 |
| A*β*42/40 + pTau181 + pTau217/A*β*40 + pTau181/A*β*40 | 0.927 | 124 | 0.862 | 116 |
| A*β*42/40 + pTau217 + pTau181 + pTau217/A*β*40 + pTau181/A*β*40 | 0.927 | 123 | 0.862 | 116 |
|  |  |  |  | |

| Combination | A+/T+ in BBM+ | n BBM+ | non-A+/T+ in BBM- | n BBM- |
| --- | --- | --- | --- | --- |
| A*β*42/40 + pTau217 + pTau181 + pTau217/A*β*42 + pTau181/A*β*40 | 0.927 | 124 | 0.856 | 118 |
| A*β*42/40 + pTau181 + pTau217/A*β*40 + pTau217/A*β*42 + pTau181/A*β*40 | 0.927 | 124 | 0.862 | 116 |
| A*β*42/40 + pTau217 + pTau181 + pTau217/A*β*40 + pTau217/A*β*42 +  pTau181/A*β*40 | 0.927 | 123 | 0.862 | 116 |
| pTau217 + pTau217/A*β*40 + pTau217/A*β*42 + pTau181/A*β*40 | 0.927 | 151 | 0.739 | 218 |
| A*β*42/40 + pTau217 + NfL + pTau217/A*β*40 + pTau217/A*β*42 | 0.926 | 108 | 0.882 | 93 |
| pTau217 + NfL + pTau181/A*β*40 | 0.926 | 122 | 0.743 | 152 |
| A*β*42/40 + NfL + pTau217/A*β*40 | 0.926 | 108 | 0.882 | 93 |
| pTau217 + pTau181 + NfL | 0.926 | 122 | 0.741 | 158 |
| A*β*42/40 + NfL + pTau217/A*β*40 + pTau217/A*β*42 | 0.926 | 108 | 0.882 | 93 |
| A*β*42/40 + pTau217 + NfL + pTau217/A*β*42 | 0.926 | 108 | 0.874 | 95 |
| A*β*42/40 + pTau217 + NfL + pTau217/A*β*40 | 0.926 | 108 | 0.882 | 93 |
| A*β*42/40 + NfL + pTau217/A*β*42 | 0.926 | 108 | 0.865 | 96 |
| NfL + pTau217/A*β*40 | 0.924 | 131 | 0.733 | 165 |
| pTau217 + NfL + pTau217/A*β*40 | 0.924 | 131 | 0.736 | 163 |
| NfL + pTau217/A*β*42 | 0.923 | 130 | 0.727 | 161 |
| NfL + pTau217/A*β*40 + pTau217/A*β*42 | 0.923 | 130 | 0.734 | 158 |
| pTau217 + NfL + pTau217/A*β*42 | 0.923 | 130 | 0.731 | 160 |
| pTau217 + pTau181 | 0.923 | 155 | 0.743 | 222 |
| pTau217 + pTau181/A*β*40 | 0.923 | 156 | 0.733 | 225 |
| pTau217 + NfL + pTau217/A*β*40 + pTau217/A*β*42 | 0.923 | 130 | 0.734 | 158 |
| pTau217/A*β*42 + pTau181/A*β*40 | 0.923 | 156 | 0.731 | 223 |
| A*β*42/40 + pTau217 + pTau181 | 0.922 | 129 | 0.846 | 123 |
| A*β*42/40 + pTau217 + NfL + pTau181/A*β*40 | 0.922 | 102 | 0.874 | 87 |
| A*β*42/40 + pTau181 + NfL + pTau181/A*β*40 | 0.922 | 102 | 0.864 | 88 |
| A*β*42/40 + NfL + pTau217/A*β*42 + pTau181/A*β*40 | 0.921 | 101 | 0.874 | 87 |
| A*β*42/40 + NfL + pTau217/A*β*40 + pTau181/A*β*40 | 0.921 | 101 | 0.893 | 84 |
| A*β*42/40 + pTau217 + NfL + pTau217/A*β*40 + pTau181/A*β*40 | 0.921 | 101 | 0.893 | 84 |
| A*β*42/40 + NfL + pTau217/A*β*40 + pTau217/A*β*42 + pTau181/A*β*40 | 0.921 | 101 | 0.893 | 84 |
| A*β*42/40 + pTau217 + NfL + pTau217/A*β*42 + pTau181/A*β*40 | 0.921 | 101 | 0.884 | 86 |
|  |  |  |  | |

|  | Combination | | A+/T+ in BBM+ | | n BBM+ | | non-A+/T+ in BBM- | | n BBM- | |
| --- | --- | --- | --- | --- | --- | --- | --- | --- | --- | --- |
|  | A*β*42/40 + pTau217 + NfL + pTau217/A*β*40 + pTau217/A*β*42 + pTau181/A*β*40 | | 0.921 | | 101 | | 0.893 | | 84 | |
|  | A*β*42/40 + pTau181 + pTau181/A*β*40 | | 0.919 | | 135 | | 0.823 | | 124 | |
|  | A*β*42/40 + pTau217 + NfL | | 0.918 | | 110 | | 0.865 | | 96 | |
|  | A*β*42/40 + pTau217 + pTau181/A*β*40 | | 0.917 | | 132 | | 0.839 | | 124 | |
|  | A*β*42/40 + pTau217/A*β*40 + pTau181/A*β*40 | | 0.917 | | 132 | | 0.844 | | 122 | |
|  | A*β*42/40 + pTau217/A*β*40 + pTau217/A*β*42 + pTau181/A*β*40 | | 0.917 | | 132 | | 0.844 | | 122 | |
|  | A*β*42/40 + pTau217 + pTau217/A*β*42 + pTau181/A*β*40 | | 0.916 | | 131 | | 0.846 | | 123 | |
|  | pTau217/A*β*40 + pTau217/A*β*42 | | 0.916 | | 178 | | 0.714 | | 245 | |
|  | pTau217 + pTau217/A*β*40 | | 0.915 | | 176 | | 0.722 | | 245 | |
|  | A*β*42/40 + pTau217 + pTau217/A*β*40 + pTau181/A*β*40 | | 0.915 | | 130 | | 0.851 | | 121 | |
|  | A*β*42/40 + pTau217 + pTau217/A*β*40 + pTau217/A*β*42 + pTau181/A*β*40 | | 0.915 | | 130 | | 0.851 | | 121 | |
|  | pTau217 + pTau217/A*β*40 + pTau217/A*β*42 | | 0.914 | | 174 | | 0.721 | | 240 | |
|  | pTau217 + NfL | | 0.912 | | 136 | | 0.716 | | 169 | |
|  | pTau217/A*β*40 | | 0.912 | | 182 | | 0.714 | | 252 | |
|  | A*β*42/40 + pTau217/A*β*40 | | 0.912 | | 148 | | 0.837 | | 135 | |
|  | A*β*42/40 + pTau217/A*β*40 + pTau217/A*β*42 | | 0.912 | | 148 | | 0.837 | | 135 | |
|  | A*β*42/40 + pTau217/A*β*42 + pTau181/A*β*40 | | 0.911 | | 135 | | 0.832 | | 125 | |
|  | pTau181 + NfL + pTau181/A*β*40 | | 0.911 | | 123 | | 0.72 | | 161 | |
|  | A*β*42/40 + pTau217 + pTau217/A*β*40 | | 0.91 | | 145 | | 0.848 | | 132 | |
|  | A*β*42/40 + pTau217 + pTau217/A*β*40 + pTau217/A*β*42 | | 0.91 | | 145 | | 0.848 | | 132 | |
|  | pTau217 + pTau217/A*β*42 | | 0.909 | | 176 | | 0.719 | | 242 | |
|  | A*β*42/40 + pTau217 + pTau217/A*β*42 | | 0.905 | | 147 | | 0.843 | | 134 | |
|  | A*β*42/40 + pTau181 | | 0.904 | | 146 | | 0.802 | | 131 | |
|  | pTau217 | | 0.902 | | 183 | | 0.709 | | 251 | |
|  | A*β*42/40 + pTau181 + NfL | | 0.902 | | 112 | | 0.84 | | 94 | |
|  | A*β*42/40 + pTau217 | | 0.899 | | 149 | | 0.837 | | 135 | |
|  | A*β*42/40 + NfL + pTau181/A*β*40 | | 0.899 | | 109 | | 0.835 | | 91 | |
|  | A*β*42/40 + pTau181/A*β*40 | | 0.898 | | 147 | | 0.791 | | 134 | |
|  | NfL + pTau181/A*β*40 | | 0.893 | | 131 | | 0.703 | | 165 | |
|  | pTau217/A*β*42 | | 0.892 | | 185 | | 0.707 | | 249 | |
|  | pTau181 + pTau181/A*β*40 | | 0.89 | | 163 | | 0.71 | | 231 | |
|  | A*β*42/40 + pTau217/A*β*42 | | 0.884 | | 155 | | 0.82 | | 139 | |
|  | pTau181 + NfL | | 0.871 | | 140 | | 0.698 | | 172 | |
|  | pTau181 | | 0.864 | | 184 | | 0.684 | | 250 | |
|  | pTau181/A*β*40 | | 0.863 | | 182 | | 0.679 | | 252 | |
| Combination | | A+/T+ in BBM+ | | n BBM+ | | non-A+/T+ in BBM- | | n BBM- | |  |
| A*β*42/40 + NfL | | 0.765 | | 153 | | 0.798 | | 104 | |  |
| A*β*42/40 | | 0.698 | | 265 | | 0.686 | | 169 | |  |
| NfL | | 0.683 | | 218 | | 0.588 | | 216 | |  |

Supplementary table 3 Cutoff combinations — MiniBatch K-Means (all 127 combinations). Prevalence of A^+^/T^+^ CSF profile in BBM-positive subgroups and prevalence of non-A^+^/T^+^ CSF profile in BBM-negative subgroups.

| Combination | A+/T+ in BBM+ | n BBM+ | non-A+/T+ in BBM- | n BBM- |
| --- | --- | --- | --- | --- |
| pTau181 + NfL + pTau217/A*β*40 + pTau217/A*β*42 + pTau181/A*β*40 | 0.946 | 112 | 0.773 | 141 |
| pTau181 + NfL + pTau217/A*β*42 + pTau181/A*β*40 | 0.946 | 112 | 0.764 | 144 |
| pTau217 + pTau181 + NfL + pTau217/A*β*40 + pTau181/A*β*40 | 0.946 | 112 | 0.778 | 144 |
| pTau217 + pTau181 + NfL + pTau217/A*β*40 + pTau217/A*β*42 + pTau181/A*β*40 | 0.946 | 112 | 0.773 | 141 |
| pTau181 + NfL + pTau217/A*β*40 + pTau181/A*β*40 | 0.946 | 112 | 0.779 | 145 |
| pTau217 + pTau181 + NfL + pTau217/A*β*42 + pTau181/A*β*40 | 0.946 | 112 | 0.769 | 143 |
| pTau181 + NfL + pTau217/A*β*42 | 0.94 | 117 | 0.758 | 149 |
| pTau217 + pTau181 + NfL + pTau217/A*β*40 | 0.94 | 117 | 0.768 | 151 |
| pTau217 + pTau181 + NfL + pTau217/A*β*42 | 0.94 | 117 | 0.764 | 148 |
| pTau181 + NfL + pTau217/A*β*40 | 0.94 | 117 | 0.77 | 152 |
| pTau217 + pTau181 + NfL + pTau217/A*β*40 + pTau217/A*β*42 | 0.94 | 117 | 0.767 | 146 |
| pTau181 + NfL + pTau217/A*β*40 + pTau217/A*β*42 | 0.94 | 117 | 0.767 | 146 |
| pTau217 + pTau181 + NfL + pTau181/A*β*40 | 0.939 | 115 | 0.764 | 148 |
| pTau181 + pTau217/A*β*40 + pTau181/A*β*40 | 0.938 | 145 | 0.768 | 207 |
| pTau181 + pTau217/A*β*42 + pTau181/A*β*40 | 0.938 | 146 | 0.757 | 206 |
| A*β*42/40 + pTau181 + NfL + pTau217/A*β*42 | 0.938 | 97 | 0.876 | 89 |
| pTau217 + pTau181 + pTau217/A*β*40 + pTau181/A*β*40 | 0.938 | 144 | 0.771 | 205 |
| A*β*42/40 + pTau181 + NfL + pTau217/A*β*40 | 0.938 | 97 | 0.895 | 86 |
| pTau217 + pTau181 + pTau217/A*β*42 + pTau181/A*β*40 | 0.938 | 145 | 0.765 | 204 |
| A*β*42/40 + pTau217 + pTau181 + NfL + pTau217/A*β*40 | 0.938 | 97 | 0.895 | 86 |

| Combination | A+/T+ in BBM+ | n BBM+ | non-A+/T+ in BBM- | n BBM- |
| --- | --- | --- | --- | --- |
| A*β*42/40 + pTau217 + pTau181 + NfL + pTau217/A*β*40 + pTau217/A*β*42 | 0.938 | 97 | 0.895 | 86 |
| pTau217 + pTau181 + pTau217/A*β*40 + pTau217/A*β*42 + pTau181/A*β*40 | 0.938 | 144 | 0.767 | 202 |
| pTau181 + pTau217/A*β*40 + pTau217/A*β*42 + pTau181/A*β*40 | 0.938 | 145 | 0.764 | 203 |
| A*β*42/40 + pTau181 + NfL + pTau217/A*β*40 + pTau217/A*β*42 | 0.938 | 97 | 0.895 | 86 |
| A*β*42/40 + pTau217 + pTau181 + NfL + pTau217/A*β*42 | 0.938 | 97 | 0.886 | 88 |
| A*β*42/40 + pTau217 + pTau181 + NfL + pTau181/A*β*40 | 0.937 | 95 | 0.882 | 85 |
| A*β*42/40 + pTau217 + pTau181 + NfL + pTau217/A*β*42 + pTau181/A*β*40 | 0.936 | 94 | 0.893 | 84 |
| A*β*42/40 + pTau181 + NfL + pTau217/A*β*40 + pTau181/A*β*40 | 0.936 | 94 | 0.902 | 82 |
| A*β*42/40 + pTau181 + NfL + pTau217/A*β*40 + pTau217/A*β*42 + pTau181/A*β*40 | 0.936 | 94 | 0.902 | 82 |
| A*β*42/40 + pTau217 + pTau181 + NfL + pTau217/A*β*40 + pTau181/A*β*40 | 0.936 | 94 | 0.902 | 82 |
| A*β*42/40 + pTau181 + NfL + pTau217/A*β*42 + pTau181/A*β*40 | 0.936 | 94 | 0.882 | 85 |
| A*β*42/40 + pTau217 + pTau181 + NfL + pTau217/A*β*40 + pTau217/A*β*42 +  pTau181/A*β*40 | 0.936 | 94 | 0.902 | 82 |
| pTau181 + pTau217/A*β*42 | 0.935 | 154 | 0.755 | 212 |
| pTau181 + pTau217/A*β*40 | 0.935 | 153 | 0.763 | 215 |
| pTau217 + pTau181 + pTau217/A*β*42 | 0.935 | 153 | 0.762 | 210 |
| pTau181 + pTau217/A*β*40 + pTau217/A*β*42 | 0.935 | 153 | 0.761 | 209 |
| pTau217 + pTau181 + pTau217/A*β*40 | 0.934 | 152 | 0.765 | 213 |
| pTau217 + pTau181 + pTau217/A*β*40 + pTau217/A*β*42 | 0.934 | 152 | 0.764 | 208 |
| pTau217 + NfL + pTau217/A*β*40 + pTau217/A*β*42 + pTau181/A*β*40 | 0.932 | 118 | 0.752 | 145 |
| pTau217 + NfL + pTau217/A*β*42 + pTau181/A*β*40 | 0.932 | 118 | 0.748 | 147 |
| pTau217 + NfL + pTau217/A*β*40 + pTau181/A*β*40 | 0.932 | 118 | 0.757 | 148 |
| pTau217 + pTau181 + pTau181/A*β*40 | 0.932 | 148 | 0.761 | 209 |
| NfL + pTau217/A*β*40 + pTau181/A*β*40 | 0.932 | 118 | 0.758 | 149 |
| NfL + pTau217/A*β*42 + pTau181/A*β*40 | 0.932 | 118 | 0.743 | 148 |

| Combination | A+/T+ in BBM+ | n BBM+ | non-A+/T+ in BBM- | n BBM- |
| --- | --- | --- | --- | --- |
| NfL + pTau217/A*β*40 + pTau217/A*β*42 + pTau181/A*β*40 | 0.932 | 118 | 0.752 | 145 |
| A*β*42/40 + pTau181 + pTau217/A*β*42 | 0.931 | 130 | 0.843 | 121 |
| A*β*42/40 + pTau181 + pTau217/A*β*40 | 0.93 | 129 | 0.856 | 118 |
| A*β*42/40 + pTau181 + pTau217/A*β*40 + pTau217/A*β*42 | 0.93 | 129 | 0.856 | 118 |
| A*β*42/40 + pTau217 + pTau181 + pTau217/A*β*42 | 0.93 | 129 | 0.857 | 119 |
| A*β*42/40 + pTau217 + pTau181 + pTau217/A*β*40 | 0.93 | 128 | 0.863 | 117 |
| A*β*42/40 + pTau217 + pTau181 + pTau217/A*β*40 + pTau217/A*β*42 | 0.93 | 128 | 0.863 | 117 |
| pTau217/A*β*40 + pTau217/A*β*42 + pTau181/A*β*40 | 0.929 | 154 | 0.736 | 220 |
| A*β*42/40 + pTau217 + pTau181 + NfL | 0.929 | 99 | 0.876 | 89 |
| A*β*42/40 + pTau181 + pTau217/A*β*42 + pTau181/A*β*40 | 0.929 | 126 | 0.846 | 117 |
| pTau217/A*β*40 + pTau181/A*β*40 | 0.929 | 154 | 0.741 | 224 |
| A*β*42/40 + pTau217 + pTau181 + pTau181/A*β*40 | 0.929 | 126 | 0.853 | 116 |
| pTau217 + pTau217/A*β*42 + pTau181/A*β*40 | 0.928 | 153 | 0.743 | 218 |
| pTau217 + pTau217/A*β*40 + pTau181/A*β*40 | 0.928 | 152 | 0.749 | 219 |
| A*β*42/40 + pTau181 + pTau217/A*β*40 + pTau181/A*β*40 | 0.928 | 125 | 0.86 | 114 |
| A*β*42/40 + pTau217 + pTau181 + pTau217/A*β*42 + pTau181/A*β*40 | 0.928 | 125 | 0.861 | 115 |
| A*β*42/40 + pTau181 + pTau217/A*β*40 + pTau217/A*β*42 + pTau181/A*β*40 | 0.928 | 125 | 0.86 | 114 |
| pTau217 + pTau217/A*β*40 + pTau217/A*β*42 + pTau181/A*β*40 | 0.928 | 152 | 0.745 | 216 |
| pTau217 + pTau181 + NfL | 0.927 | 123 | 0.75 | 156 |
| A*β*42/40 + pTau217 + pTau181 + pTau217/A*β*40 + pTau181/A*β*40 | 0.927 | 124 | 0.867 | 113 |
| A*β*42/40 + pTau217 + pTau181 + pTau217/A*β*40 + pTau217/A*β*42 +  pTau181/A*β*40 | 0.927 | 124 | 0.867 | 113 |
| pTau217 + NfL + pTau181/A*β*40 | 0.926 | 121 | 0.743 | 152 |
| A*β*42/40 + NfL + pTau217/A*β*40 | 0.926 | 108 | 0.88 | 92 |
| A*β*42/40 + pTau217 + NfL + pTau217/A*β*40 + pTau217/A*β*42 | 0.926 | 108 | 0.88 | 92 |
| A*β*42/40 + NfL + pTau217/A*β*40 + pTau217/A*β*42 | 0.926 | 108 | 0.88 | 92 |
|  |  |  |  | |

| Combination | A+/T+ in BBM+ | n BBM+ | non-A+/T+ in BBM- | n BBM- |
| --- | --- | --- | --- | --- |
| A*β*42/40 + pTau217 + NfL + pTau217/A*β*42 | 0.926 | 108 | 0.872 | 94 |
| A*β*42/40 + pTau217 + NfL + pTau217/A*β*40 | 0.926 | 108 | 0.88 | 92 |
| A*β*42/40 + NfL + pTau217/A*β*42 | 0.926 | 108 | 0.863 | 95 |
| pTau217 + pTau181 | 0.925 | 159 | 0.752 | 218 |
| pTau217/A*β*42 + pTau181/A*β*40 | 0.924 | 157 | 0.731 | 223 |
| A*β*42/40 + pTau217 + pTau181 | 0.924 | 131 | 0.85 | 120 |
| NfL + pTau217/A*β*40 + pTau217/A*β*42 | 0.923 | 130 | 0.734 | 158 |
| NfL + pTau217/A*β*40 | 0.923 | 130 | 0.733 | 165 |
| pTau217 + pTau181/A*β*40 | 0.923 | 156 | 0.74 | 223 |
| pTau217 + NfL + pTau217/A*β*40 | 0.923 | 130 | 0.736 | 163 |
| NfL + pTau217/A*β*42 | 0.923 | 130 | 0.727 | 161 |
| pTau217 + NfL + pTau217/A*β*42 | 0.923 | 130 | 0.731 | 160 |
| pTau217 + NfL + pTau217/A*β*40 + pTau217/A*β*42 | 0.923 | 130 | 0.734 | 158 |
| A*β*42/40 + pTau217 + NfL + pTau181/A*β*40 | 0.921 | 101 | 0.872 | 86 |
| A*β*42/40 + pTau181 + NfL + pTau181/A*β*40 | 0.921 | 101 | 0.862 | 87 |
| A*β*42/40 + NfL + pTau217/A*β*42 + pTau181/A*β*40 | 0.92 | 100 | 0.872 | 86 |
| A*β*42/40 + NfL + pTau217/A*β*40 + pTau181/A*β*40 | 0.92 | 100 | 0.892 | 83 |
| A*β*42/40 + pTau217 + NfL + pTau217/A*β*40 + pTau181/A*β*40 | 0.92 | 100 | 0.892 | 83 |
| A*β*42/40 + NfL + pTau217/A*β*40 + pTau217/A*β*42 + pTau181/A*β*40 | 0.92 | 100 | 0.892 | 83 |
| A*β*42/40 + pTau217 + NfL + pTau217/A*β*42 + pTau181/A*β*40 | 0.92 | 100 | 0.882 | 85 |
| A*β*42/40 + pTau217 + NfL + pTau217/A*β*40 + pTau217/A*β*42 + pTau181/A*β*40 | 0.92 | 100 | 0.892 | 83 |
| A*β*42/40 + pTau181 + pTau181/A*β*40 | 0.919 | 136 | 0.826 | 121 |
| A*β*42/40 + pTau217 + NfL | 0.918 | 110 | 0.863 | 95 |
| A*β*42/40 + pTau217 + pTau181/A*β*40 | 0.917 | 132 | 0.851 | 121 |
| A*β*42/40 + pTau217/A*β*40 + pTau181/A*β*40 | 0.917 | 132 | 0.843 | 121 |
| A*β*42/40 + pTau217/A*β*40 + pTau217/A*β*42 + pTau181/A*β*40 | 0.917 | 132 | 0.843 | 121 |
| A*β*42/40 + pTau217 + pTau217/A*β*42 + pTau181/A*β*40 | 0.916 | 131 | 0.858 | 120 |
| pTau217/A*β*40 + pTau217/A*β*42 | 0.916 | 179 | 0.714 | 245 |
| pTau217 + pTau217/A*β*40 | 0.915 | 176 | 0.728 | 243 |
| A*β*42/40 + pTau217 + pTau217/A*β*40 + pTau181/A*β*40 | 0.915 | 130 | 0.864 | 118 |
| A*β*42/40 + pTau217 + pTau217/A*β*40 + pTau217/A*β*42 + pTau181/A*β*40 | 0.915 | 130 | 0.864 | 118 |
|  |  |  |  | |

| Combination | A+/T+ in BBM+ | n BBM+ | non-A+/T+ in BBM- | n BBM- |
| --- | --- | --- | --- | --- |
| pTau217 + pTau217/A*β*40 + pTau217/A*β*42 | 0.914 | 175 | 0.727 | 238 |
| A*β*42/40 + pTau217/A*β*40 | 0.913 | 149 | 0.836 | 134 |
| A*β*42/40 + pTau217/A*β*40 + pTau217/A*β*42 | 0.913 | 149 | 0.836 | 134 |
| pTau217 + NfL | 0.912 | 137 | 0.716 | 169 |
| pTau217/A*β*40 | 0.912 | 182 | 0.714 | 252 |
| A*β*42/40 + pTau217/A*β*42 + pTau181/A*β*40 | 0.911 | 135 | 0.831 | 124 |
| pTau181 + NfL + pTau181/A*β*40 | 0.911 | 123 | 0.734 | 158 |
| A*β*42/40 + pTau217 + pTau217/A*β*40 | 0.911 | 146 | 0.86 | 129 |
| A*β*42/40 + pTau217 + pTau217/A*β*40 + pTau217/A*β*42 | 0.911 | 146 | 0.86 | 129 |
| pTau217 + pTau217/A*β*42 | 0.91 | 177 | 0.725 | 240 |
| A*β*42/40 + pTau181 | 0.907 | 150 | 0.811 | 127 |
| A*β*42/40 + pTau217 + pTau217/A*β*42 | 0.905 | 148 | 0.855 | 131 |
| pTau217 | 0.903 | 185 | 0.715 | 249 |
| A*β*42/40 + pTau181 + NfL | 0.901 | 111 | 0.839 | 93 |
| A*β*42/40 + pTau217 | 0.9 | 150 | 0.848 | 132 |
| A*β*42/40 + NfL + pTau181/A*β*40 | 0.898 | 108 | 0.833 | 90 |
| A*β*42/40 + pTau181/A*β*40 | 0.898 | 147 | 0.795 | 132 |
| pTau217/A*β*42 | 0.892 | 186 | 0.71 | 248 |
| NfL + pTau181/A*β*40 | 0.892 | 130 | 0.703 | 165 |
| pTau181 + pTau181/A*β*40 | 0.891 | 165 | 0.718 | 227 |
| A*β*42/40 + pTau217/A*β*42 | 0.885 | 156 | 0.825 | 137 |
| pTau181 + NfL | 0.865 | 141 | 0.714 | 168 |
| pTau181/A*β*40 | 0.863 | 182 | 0.679 | 252 |
| pTau181 | 0.863 | 190 | 0.697 | 244 |
| A*β*42/40 + NfL | 0.765 | 153 | 0.796 | 103 |
| A*β*42/40 | 0.697 | 267 | 0.689 | 167 |
| NfL | 0.682 | 217 | 0.585 | 217 |

Supplementary table 4 Cutoff combinations — Agglomerative (Ward) (all 127 combinations). Prevalence of A^+^/T^+^ CSF profile in BBM-positive subgroups and prevalence of non-A^+^/T^+^ CSF profile in BBM-negative subgroups.

| Combination | A+/T+ in BBM+ | n BBM+ non-A+/T+ in BBM- n BBM- |
| --- | --- | --- |
| pTau217 + pTau181 + NfL + pTau217/A*β*40 + pTau181/A*β*40 | 0.939 | 131 0.812 128 |
| pTau217 + pTau181 + NfL + pTau217/A*β*42 | 0.939 | 131 0.801 136 |
| pTau217 + pTau181 + NfL + pTau217/A*β*40 | 0.939 | 131 0.799 139 |
| pTau217 + pTau181 + NfL + pTau217/A*β*42 + pTau181/A*β*40 | 0.938 | 130 0.817 126 |

| Combination | A+/T+ in BBM+ | n BBM+ | non-A+/T+ in BBM- | n BBM- |
| --- | --- | --- | --- | --- |
| A*β*42/40 + pTau217 + pTau181 + NfL + pTau181/A*β*40 | 0.938 | 113 | 0.921 | 76 |
| pTau217 + pTau181 + NfL + pTau217/A*β*40 + pTau217/A*β*42 | 0.938 | 129 | 0.8 | 135 |
| A*β*42/40 + pTau217 + pTau181 + NfL + pTau217/A*β*42 | 0.938 | 112 | 0.901 | 81 |
| pTau217 + pTau181 + NfL + pTau217/A*β*40 + pTau217/A*β*42 + pTau181/A*β*40 | 0.938 | 129 | 0.816 | 125 |
| A*β*42/40 + pTau217 + pTau181 + NfL + pTau217/A*β*42 + pTau181/A*β*40 | 0.937 | 111 | 0.921 | 76 |
| A*β*42/40 + pTau217 + pTau181 + NfL + pTau217/A*β*40 | 0.936 | 110 | 0.9 | 80 |
| A*β*42/40 + pTau217 + pTau181 + NfL + pTau217/A*β*40 + pTau217/A*β*42 | 0.936 | 110 | 0.9 | 80 |
| A*β*42/40 + pTau217 + pTau181 + NfL + pTau217/A*β*40 + pTau217/A*β*42 +  pTau181/A*β*40 | 0.936 | 110 | 0.92 | 75 |
| A*β*42/40 + pTau217 + pTau181 + NfL + pTau217/A*β*40 + pTau181/A*β*40 | 0.936 | 110 | 0.92 | 75 |
| pTau217 + pTau181 + NfL + pTau181/A*β*40 | 0.934 | 136 | 0.814 | 129 |
| pTau217 + pTau181 + pTau217/A*β*42 | 0.934 | 166 | 0.803 | 188 |
| pTau217 + pTau181 + pTau217/A*β*40 + pTau217/A*β*42 | 0.933 | 163 | 0.802 | 187 |
| pTau217 + pTau181 + pTau217/A*β*40 + pTau181/A*β*40 | 0.933 | 165 | 0.81 | 179 |
| pTau217 + pTau181 + pTau217/A*β*40 | 0.933 | 165 | 0.801 | 191 |
| pTau217 + pTau181 + pTau217/A*β*40 + pTau217/A*β*42 + pTau181/A*β*40 | 0.933 | 163 | 0.812 | 176 |
| pTau217 + pTau181 + pTau217/A*β*42 + pTau181/A*β*40 | 0.933 | 164 | 0.814 | 177 |
| pTau217 + NfL + pTau217/A*β*40 + pTau181/A*β*40 | 0.932 | 133 | 0.789 | 133 |
| pTau217 + NfL + pTau217/A*β*42 + pTau181/A*β*40 | 0.932 | 132 | 0.794 | 131 |
| pTau181 + NfL + pTau217/A*β*40 | 0.932 | 132 | 0.8 | 140 |
| pTau181 + NfL + pTau217/A*β*40 + pTau181/A*β*40 | 0.932 | 132 | 0.814 | 129 |
| pTau217 + NfL + pTau217/A*β*40 + pTau217/A*β*42 + pTau181/A*β*40 | 0.931 | 131 | 0.792 | 130 |
| A*β*42/40 + pTau217 + pTau181 + NfL | 0.931 | 116 | 0.901 | 81 |
| pTau181 + NfL + pTau217/A*β*40 + pTau217/A*β*42 + pTau181/A*β*40 | 0.931 | 130 | 0.816 | 125 |
|  |  |  |  | |

| Combination | A+/T+ in BBM+ | n BBM+ | non-A+/T+ in BBM- | n BBM- |
| --- | --- | --- | --- | --- |
| pTau217 + pTau217/A*β*40 + pTau181/A*β*40 | 0.931 | 173 | 0.789 | 199 |
| A*β*42/40 + pTau217 + pTau181 + pTau181/A*β*40 | 0.931 | 144 | 0.903 | 103 |
| A*β*42/40 + pTau217 + pTau181 + pTau217/A*β*42 | 0.931 | 144 | 0.889 | 108 |
| pTau181 + NfL + pTau217/A*β*40 + pTau217/A*β*42 | 0.931 | 130 | 0.8 | 135 |
| A*β*42/40 + pTau217 + pTau181 + pTau217/A*β*42 + pTau181/A*β*40 | 0.93 | 142 | 0.903 | 103 |
| pTau217 + pTau181 + pTau181/A*β*40 | 0.93 | 171 | 0.811 | 180 |
| pTau217 + pTau217/A*β*40 + pTau217/A*β*42 + pTau181/A*β*40 | 0.93 | 171 | 0.791 | 196 |
| A*β*42/40 + pTau217 + NfL + pTau181/A*β*40 | 0.93 | 115 | 0.922 | 77 |
| pTau217 + pTau217/A*β*42 + pTau181/A*β*40 | 0.93 | 172 | 0.792 | 197 |
| A*β*42/40 + pTau217 + pTau181 + pTau217/A*β*40 + pTau181/A*β*40 | 0.929 | 141 | 0.902 | 102 |
| A*β*42/40 + pTau217 + pTau181 + pTau217/A*β*40 | 0.929 | 141 | 0.888 | 107 |
| pTau181 + pTau217/A*β*40 | 0.929 | 168 | 0.802 | 192 |
| pTau217 + pTau181 + NfL | 0.929 | 140 | 0.8 | 140 |
| pTau181 + pTau217/A*β*40 + pTau181/A*β*40 | 0.929 | 168 | 0.811 | 180 |
| A*β*42/40 + pTau217 + NfL + pTau217/A*β*40 + pTau181/A*β*40 | 0.929 | 112 | 0.921 | 76 |
| A*β*42/40 + pTau217 + NfL + pTau217/A*β*40 + pTau217/A*β*42 + pTau181/A*β*40 | 0.929 | 112 | 0.921 | 76 |
| A*β*42/40 + pTau217 + pTau181 + pTau217/A*β*40 + pTau217/A*β*42 +  pTau181/A*β*40 | 0.929 | 141 | 0.902 | 102 |
| A*β*42/40 + pTau217 + NfL + pTau217/A*β*42 + pTau181/A*β*40 | 0.929 | 113 | 0.922 | 77 |
| A*β*42/40 + pTau217 + pTau181 + pTau217/A*β*40 + pTau217/A*β*42 | 0.929 | 141 | 0.888 | 107 |
| pTau217 + NfL + pTau181/A*β*40 | 0.928 | 138 | 0.791 | 134 |
| A*β*42/40 + pTau181 + NfL + pTau217/A*β*40 + pTau181/A*β*40 | 0.928 | 111 | 0.92 | 75 |
| A*β*42/40 + pTau181 + NfL + pTau217/A*β*40 + pTau217/A*β*42 | 0.928 | 111 | 0.9 | 80 |
| A*β*42/40 + pTau181 + NfL + pTau217/A*β*40 + pTau217/A*β*42 + pTau181/A*β*40 | 0.928 | 111 | 0.92 | 75 |
| A*β*42/40 + pTau181 + NfL + pTau217/A*β*40 | 0.928 | 111 | 0.9 | 80 |
| A*β*42/40 + pTau217 + pTau181/A*β*40 | 0.927 | 150 | 0.899 | 109 |

| Combination | A+/T+ in BBM+ | n BBM+ | non-A+/T+ in BBM- | n BBM- |
| --- | --- | --- | --- | --- |
| pTau181 + pTau217/A*β*40 + pTau217/A*β*42 | 0.927 | 165 | 0.802 | 187 |
| pTau181 + pTau217/A*β*40 + pTau217/A*β*42 + pTau181/A*β*40 | 0.927 | 165 | 0.812 | 176 |
| pTau217 + pTau181/A*β*40 | 0.927 | 179 | 0.79 | 200 |
| A*β*42/40 + pTau217 + pTau181 | 0.926 | 148 | 0.889 | 108 |
| A*β*42/40 + pTau217 + pTau217/A*β*42 + pTau181/A*β*40 | 0.926 | 148 | 0.899 | 109 |
| pTau217/A*β*40 + pTau181/A*β*40 | 0.926 | 176 | 0.776 | 205 |
| pTau217 + pTau181 | 0.926 | 176 | 0.802 | 192 |
| pTau181 + NfL + pTau217/A*β*42 + pTau181/A*β*40 | 0.925 | 133 | 0.811 | 127 |
| pTau181 + NfL + pTau217/A*β*42 | 0.925 | 134 | 0.796 | 137 |
| NfL + pTau217/A*β*40 + pTau181/A*β*40 | 0.925 | 134 | 0.785 | 135 |
| pTau217/A*β*40 + pTau217/A*β*42 + pTau181/A*β*40 | 0.925 | 173 | 0.784 | 199 |
| A*β*42/40 + pTau217 + pTau217/A*β*40 + pTau217/A*β*42 + pTau181/A*β*40 | 0.925 | 147 | 0.898 | 108 |
| A*β*42/40 + pTau217 + pTau217/A*β*40 + pTau181/A*β*40 | 0.925 | 147 | 0.898 | 108 |
| NfL + pTau217/A*β*40 + pTau217/A*β*42 + pTau181/A*β*40 | 0.924 | 132 | 0.792 | 130 |
| pTau181 + pTau217/A*β*42 | 0.924 | 170 | 0.795 | 190 |
| A*β*42/40 + pTau181 + pTau217/A*β*40 + pTau217/A*β*42 + pTau181/A*β*40 | 0.923 | 143 | 0.902 | 102 |
| A*β*42/40 + pTau181 + pTau217/A*β*40 + pTau217/A*β*42 | 0.923 | 143 | 0.888 | 107 |
| pTau181 + pTau217/A*β*42 + pTau181/A*β*40 | 0.923 | 168 | 0.804 | 179 |
| A*β*42/40 + pTau181 + pTau217/A*β*40 + pTau181/A*β*40 | 0.923 | 143 | 0.902 | 102 |
| A*β*42/40 + pTau181 + pTau217/A*β*40 | 0.923 | 143 | 0.888 | 107 |
| A*β*42/40 + pTau181 + NfL + pTau217/A*β*42 | 0.922 | 115 | 0.89 | 82 |
| pTau217 + NfL + pTau217/A*β*40 | 0.921 | 139 | 0.758 | 153 |
| A*β*42/40 + pTau181 + NfL + pTau217/A*β*42 + pTau181/A*β*40 | 0.921 | 114 | 0.909 | 77 |
| A*β*42/40 + NfL + pTau217/A*β*40 + pTau217/A*β*42 + pTau181/A*β*40 | 0.92 | 113 | 0.921 | 76 |
| A*β*42/40 + NfL + pTau217/A*β*40 + pTau181/A*β*40 | 0.92 | 113 | 0.921 | 76 |
| pTau217 + NfL + pTau217/A*β*42 | 0.92 | 138 | 0.755 | 151 |
| A*β*42/40 + pTau217/A*β*40 + pTau181/A*β*40 | 0.919 | 149 | 0.89 | 109 |
| NfL + pTau217/A*β*42 + pTau181/A*β*40 | 0.919 | 135 | 0.788 | 132 |

| Combination | A+/T+ in BBM+ | n BBM+ | non-A+/T+ in BBM- | n BBM- |
| --- | --- | --- | --- | --- |
| A*β*42/40 + pTau181 + pTau217/A*β*42 | 0.919 | 148 | 0.873 | 110 |
| A*β*42/40 + pTau217/A*β*40 + pTau217/A*β*42 + pTau181/A*β*40 | 0.919 | 149 | 0.89 | 109 |
| pTau217 + NfL + pTau217/A*β*40 + pTau217/A*β*42 | 0.919 | 136 | 0.758 | 149 |
| A*β*42/40 + pTau181 + pTau217/A*β*42 + pTau181/A*β*40 | 0.918 | 146 | 0.886 | 105 |
| A*β*42/40 + pTau181 + NfL + pTau181/A*β*40 | 0.916 | 119 | 0.888 | 80 |
| A*β*42/40 + pTau181 + pTau181/A*β*40 | 0.916 | 155 | 0.862 | 109 |
| pTau217/A*β*42 + pTau181/A*β*40 | 0.915 | 177 | 0.777 | 202 |
| A*β*42/40 + pTau217 + NfL + pTau217/A*β*42 | 0.915 | 118 | 0.886 | 88 |
| NfL + pTau217/A*β*40 | 0.914 | 140 | 0.75 | 156 |
| A*β*42/40 + pTau217 + NfL + pTau217/A*β*40 | 0.914 | 116 | 0.895 | 86 |
| A*β*42/40 + NfL + pTau217/A*β*42 + pTau181/A*β*40 | 0.914 | 116 | 0.91 | 78 |
| A*β*42/40 + pTau217 + NfL + pTau217/A*β*40 + pTau217/A*β*42 | 0.914 | 116 | 0.895 | 86 |
| pTau217 + NfL | 0.912 | 148 | 0.75 | 156 |
| NfL + pTau217/A*β*40 + pTau217/A*β*42 | 0.912 | 137 | 0.753 | 150 |
| pTau217 + pTau217/A*β*40 | 0.91 | 189 | 0.76 | 229 |
| A*β*42/40 + pTau217 + NfL | 0.91 | 122 | 0.886 | 88 |
| NfL + pTau217/A*β*42 | 0.908 | 141 | 0.745 | 153 |
| A*β*42/40 + pTau217/A*β*42 + pTau181/A*β*40 | 0.908 | 153 | 0.875 | 112 |
| pTau217 + pTau217/A*β*40 + pTau217/A*β*42 | 0.908 | 185 | 0.762 | 223 |
| A*β*42/40 + NfL + pTau217/A*β*40 + pTau217/A*β*42 | 0.906 | 117 | 0.885 | 87 |
| A*β*42/40 + NfL + pTau217/A*β*40 | 0.906 | 117 | 0.885 | 87 |
| pTau217 + pTau217/A*β*42 | 0.905 | 189 | 0.76 | 225 |
| A*β*42/40 + pTau217 + pTau217/A*β*40 | 0.904 | 157 | 0.877 | 122 |
| pTau217/A*β*40 + pTau217/A*β*42 | 0.904 | 187 | 0.745 | 231 |
| A*β*42/40 + pTau217 + pTau217/A*β*40 + pTau217/A*β*42 | 0.904 | 157 | 0.877 | 122 |
| pTau217/A*β*40 | 0.902 | 193 | 0.734 | 241 |
| A*β*42/40 + NfL + pTau181/A*β*40 | 0.902 | 122 | 0.889 | 81 |
| A*β*42/40 + pTau217 + pTau217/A*β*42 | 0.901 | 161 | 0.871 | 124 |
| A*β*42/40 + NfL + pTau217/A*β*42 | 0.901 | 121 | 0.867 | 90 |
| pTau217 | 0.9 | 201 | 0.755 | 233 |
| A*β*42/40 + pTau217/A*β*40 | 0.899 | 159 | 0.857 | 126 |
| A*β*42/40 + pTau217/A*β*40 + pTau217/A*β*42 | 0.899 | 159 | 0.857 | 126 |
| A*β*42/40 + pTau217 | 0.897 | 165 | 0.871 | 124 |
| pTau181 + NfL + pTau181/A*β*40 | 0.897 | 146 | 0.779 | 140 |

| Combination | A+/T+ in BBM+ | n BBM+ | non-A+/T+ in BBM- | n BBM- |
| --- | --- | --- | --- | --- |
| A*β*42/40 + pTau181/A*β*40 | 0.887 | 168 | 0.853 | 116 |
| NfL + pTau181/A*β*40 | 0.886 | 149 | 0.753 | 146 |
| pTau181 + pTau181/A*β*40 | 0.885 | 192 | 0.772 | 197 |
| pTau217/A*β*42 | 0.883 | 197 | 0.73 | 237 |
| A*β*42/40 + pTau181 | 0.879 | 173 | 0.836 | 116 |
| A*β*42/40 + pTau217/A*β*42 | 0.876 | 169 | 0.826 | 132 |
| A*β*42/40 + pTau181 + NfL | 0.864 | 132 | 0.851 | 87 |
| pTau181/A*β*40 | 0.854 | 212 | 0.743 | 222 |
| pTau181 | 0.839 | 217 | 0.742 | 217 |
| pTau181 + NfL | 0.836 | 165 | 0.739 | 157 |
| A*β*42/40 + NfL | 0.755 | 163 | 0.806 | 98 |
| A*β*42/40 | 0.69 | 274 | 0.694 | 160 |
| NfL | 0.68 | 225 | 0.593 | 209 |

Supplementary table 5 Cutoff combinations — Agglomerative (Complete) (all 127 combinations). Prevalence of A^+^/T^+^ CSF profile in BBM-positive subgroups and prevalence of non-A^+^/T^+^ CSF profile in BBM-negative subgroups.

| Combination | A+/T+ in BBM+ | n BBM+ | non-A+/T+ in BBM- | n BBM- |
| --- | --- | --- | --- | --- |
| pTau181 + NfL + pTau217/A*β*40 + pTau217/A*β*42 + pTau181/A*β*40 | 0.942 | 120 | 0.754 | 138 |
| pTau181 + NfL + pTau217/A*β*42 + pTau181/A*β*40 | 0.942 | 120 | 0.75 | 140 |
| pTau217 + pTau181 + NfL + pTau217/A*β*40 + pTau181/A*β*40 | 0.942 | 120 | 0.759 | 141 |
| pTau217 + pTau181 + NfL + pTau217/A*β*40 + pTau217/A*β*42 + pTau181/A*β*40 | 0.942 | 120 | 0.754 | 138 |
| pTau181 + NfL + pTau217/A*β*40 + pTau181/A*β*40 | 0.942 | 120 | 0.761 | 142 |
| pTau217 + pTau181 + NfL + pTau217/A*β*42 + pTau181/A*β*40 | 0.942 | 120 | 0.755 | 139 |
| pTau181 + pTau217/A*β*42 + pTau181/A*β*40 | 0.938 | 144 | 0.755 | 208 |
| pTau217 + pTau181 + pTau217/A*β*40 + pTau181/A*β*40 | 0.937 | 142 | 0.764 | 208 |
| pTau217 + pTau181 + pTau217/A*β*42 + pTau181/A*β*40 | 0.937 | 143 | 0.762 | 206 |
| pTau181 + pTau217/A*β*40 + pTau181/A*β*40 | 0.937 | 143 | 0.762 | 210 |
| pTau181 + pTau217/A*β*40 + pTau217/A*β*42 + pTau181/A*β*40 | 0.937 | 143 | 0.757 | 206 |
| pTau217 + pTau181 + pTau217/A*β*40 + pTau217/A*β*42 + pTau181/A*β*40 | 0.937 | 142 | 0.761 | 205 |

| Combination | A+/T+ in BBM+ | n BBM+ | non-A+/T+ in BBM- | n BBM- |
| --- | --- | --- | --- | --- |
| pTau217 + pTau181 + NfL + pTau217/A*β*40 + pTau217/A*β*42 | 0.936 | 125 | 0.748 | 143 |
| pTau181 + NfL + pTau217/A*β*40 | 0.936 | 125 | 0.752 | 149 |
| pTau181 + NfL + pTau217/A*β*42 | 0.936 | 125 | 0.745 | 145 |
| pTau217 + pTau181 + NfL + pTau217/A*β*42 | 0.936 | 125 | 0.75 | 144 |
| pTau217 + pTau181 + NfL + pTau217/A*β*40 | 0.936 | 125 | 0.75 | 148 |
| pTau181 + NfL + pTau217/A*β*40 + pTau217/A*β*42 | 0.936 | 125 | 0.748 | 143 |
| pTau217 + pTau181 + NfL + pTau181/A*β*40 | 0.935 | 123 | 0.745 | 145 |
| pTau181 + pTau217/A*β*42 | 0.934 | 151 | 0.752 | 214 |
| A*β*42/40 + pTau181 + NfL + pTau217/A*β*40 | 0.933 | 105 | 0.89 | 82 |
| pTau181 + pTau217/A*β*40 | 0.933 | 150 | 0.757 | 218 |
| pTau217 + pTau181 + pTau217/A*β*40 | 0.933 | 149 | 0.759 | 216 |
| pTau217 + pTau181 + pTau217/A*β*42 | 0.933 | 150 | 0.759 | 212 |
| pTau181 + pTau217/A*β*40 + pTau217/A*β*42 | 0.933 | 150 | 0.755 | 212 |
| A*β*42/40 + pTau217 + pTau181 + NfL + pTau217/A*β*40 | 0.933 | 105 | 0.89 | 82 |
| A*β*42/40 + pTau217 + pTau181 + NfL + pTau217/A*β*42 | 0.933 | 105 | 0.892 | 83 |
| A*β*42/40 + pTau181 + NfL + pTau217/A*β*40 + pTau217/A*β*42 | 0.933 | 105 | 0.89 | 82 |
| A*β*42/40 + pTau217 + pTau181 + NfL + pTau217/A*β*40 + pTau217/A*β*42 | 0.933 | 105 | 0.89 | 82 |
| A*β*42/40 + pTau181 + NfL + pTau217/A*β*42 | 0.933 | 105 | 0.881 | 84 |
| pTau217 + pTau181 + pTau217/A*β*40 + pTau217/A*β*42 | 0.933 | 149 | 0.758 | 211 |
| pTau217 + pTau181 + pTau181/A*β*40 | 0.932 | 146 | 0.755 | 212 |
| A*β*42/40 + pTau217 + pTau181 + NfL + pTau181/A*β*40 | 0.932 | 103 | 0.877 | 81 |
| A*β*42/40 + pTau217 + pTau181 + NfL + pTau217/A*β*42 + pTau181/A*β*40 | 0.931 | 102 | 0.899 | 79 |
| A*β*42/40 + pTau217 + pTau181 + NfL + pTau217/A*β*40 + pTau181/A*β*40 | 0.931 | 102 | 0.897 | 78 |
| A*β*42/40 + pTau217 + pTau181 + NfL + pTau217/A*β*40 + pTau217/A*β*42 +  pTau181/A*β*40 | 0.931 | 102 | 0.897 | 78 |
| A*β*42/40 + pTau181 + NfL + pTau217/A*β*40 + pTau217/A*β*42 + pTau181/A*β*40 | 0.931 | 102 | 0.897 | 78 |
| A*β*42/40 + pTau181 + NfL + pTau217/A*β*40 + pTau181/A*β*40 | 0.931 | 102 | 0.897 | 78 |
| A*β*42/40 + pTau181 + NfL + pTau217/A*β*42 + pTau181/A*β*40 | 0.931 | 102 | 0.888 | 80 |
|  |  |  |  | |

| Combination | | A+/T+ in BBM+ | | n BBM+ | | non-A+/T+ in BBM- | | n BBM- | |
| --- | --- | --- | --- | --- | --- | --- | --- | --- | --- |
| A*β*42/40 + pTau181 + pTau217/A*β*42 | | 0.93 | | 128 | | 0.851 | | 121 | |
| A*β*42/40 + pTau217 + pTau181 + pTau217/A*β*42 | | 0.929 | | 127 | | 0.866 | | 119 | |
| A*β*42/40 + pTau181 + pTau217/A*β*40 + pTau217/A*β*42 | | 0.929 | | 127 | | 0.857 | | 119 | |
| A*β*42/40 + pTau217 + pTau181 + pTau217/A*β*40 | | 0.929 | | 126 | | 0.864 | | 118 | |
| NfL + pTau217/A*β*42 + pTau181/A*β*40 | | 0.929 | | 127 | | 0.745 | | 141 | |
| NfL + pTau217/A*β*40 + pTau181/A*β*40 | | 0.929 | | 127 | | 0.755 | | 143 | |
| pTau217/A*β*40 + pTau217/A*β*42 + pTau181/A*β*40 | | 0.929 | | 154 | | 0.736 | | 220 | |
| pTau217/A*β*40 + pTau181/A*β*40 | | 0.929 | | 154 | | 0.741 | | 224 | |
| A*β*42/40 + pTau181 + pTau217/A*β*40 | | 0.929 | | 127 | | 0.857 | | 119 | |
| pTau217 + NfL + pTau217/A*β*42 + pTau181/A*β*40 | | 0.929 | | 127 | | 0.75 | | 140 | |
| pTau217 + NfL + pTau217/A*β*40 + pTau181/A*β*40 | | 0.929 | | 127 | | 0.754 | | 142 | |
| NfL + pTau217/A*β*40 + pTau217/A*β*42 + pTau181/A*β*40 | | 0.929 | | 127 | | 0.748 | | 139 | |
| pTau217 + NfL + pTau217/A*β*40 + pTau217/A*β*42 + pTau181/A*β*40 | | 0.929 | | 127 | | 0.748 | | 139 | |
| A*β*42/40 + pTau217 + pTau181 + pTau217/A*β*40 + pTau217/A*β*42 | | 0.929 | | 126 | | 0.864 | | 118 | |
| pTau217 + pTau217/A*β*40 + pTau181/A*β*40 | | 0.928 | | 152 | | 0.749 | | 219 | |
| pTau217 + pTau217/A*β*42 + pTau181/A*β*40 | | 0.928 | | 153 | | 0.747 | | 217 | |
| pTau217 + pTau217/A*β*40 + pTau217/A*β*42 + pTau181/A*β*40 | | 0.928 | | 152 | | 0.745 | | 216 | |
| A*β*42/40 + pTau217 + pTau181 + pTau181/A*β*40 | | 0.928 | | 125 | | 0.855 | | 117 | |
| A*β*42/40 + pTau181 + pTau217/A*β*42 + pTau181/A*β*40 | | 0.928 | | 125 | | 0.855 | | 117 | |
| A*β*42/40 + pTau181 + pTau217/A*β*40 + pTau181/A*β*40 | | 0.927 | | 124 | | 0.861 | | 115 | |
| A*β*42/40 + pTau217 + pTau181 + pTau217/A*β*40 + pTau181/A*β*40 | | 0.927 | | 123 | | 0.868 | | 114 | |
| A*β*42/40 + pTau217 + pTau181 + pTau217/A*β*42 + pTau181/A*β*40 | | 0.927 | | 124 | | 0.87 | | 115 | |
| A*β*42/40 + pTau181 + pTau217/A*β*40 + pTau217/A*β*42 + pTau181/A*β*40 | | 0.927 | | 124 | | 0.861 | | 115 | |
| A*β*42/40 + pTau217 + pTau181 + pTau217/A*β*40 + pTau217/A*β*42 +  pTau181/A*β*40 | | 0.927 | | 123 | | 0.868 | | 114 | |
|  | |  | |  | |  | | | |
| Combination | A+/T+ in BBM+ | | n BBM+ | | non-A+/T+ in BBM- | | n BBM- | |  |
| A*β*42/40 + pTau217 + pTau181 + NfL | 0.925 | | 107 | | 0.871 | | 85 | |  |
| pTau217 + pTau181 + NfL | 0.924 | | 131 | | 0.732 | | 153 | |  |
| pTau217/A*β*42 + pTau181/A*β*40 | 0.924 | | 157 | | 0.734 | | 222 | |  |
| pTau217 + pTau181 | 0.923 | | 156 | | 0.747 | | 221 | |  |
| pTau217 + NfL + pTau181/A*β*40 | 0.923 | | 130 | | 0.74 | | 146 | |  |
| pTau217 + pTau181/A*β*40 | 0.923 | | 156 | | 0.74 | | 223 | |  |
| A*β*42/40 + pTau217 + pTau181 | 0.922 | | 129 | | 0.851 | | 121 | |  |
| A*β*42/40 + pTau181 + pTau181/A*β*40 | 0.919 | | 135 | | 0.828 | | 122 | |  |
| A*β*42/40 + NfL + pTau217/A*β*42 + pTau181/A*β*40 | 0.917 | | 108 | | 0.877 | | 81 | |  |
| A*β*42/40 + pTau181 + NfL + pTau181/A*β*40 | 0.917 | | 109 | | 0.855 | | 83 | |  |
| A*β*42/40 + pTau217 + NfL + pTau217/A*β*42 + pTau181/A*β*40 | 0.917 | | 108 | | 0.888 | | 80 | |  |
| A*β*42/40 + pTau217/A*β*40 + pTau181/A*β*40 | 0.917 | | 132 | | 0.843 | | 121 | |  |
| A*β*42/40 + pTau217 + pTau181/A*β*40 | 0.917 | | 132 | | 0.851 | | 121 | |  |
| pTau217/A*β*40 + pTau217/A*β*42 | 0.917 | | 180 | | 0.714 | | 245 | |  |
| A*β*42/40 + pTau217 + NfL + pTau181/A*β*40 | 0.917 | | 109 | | 0.866 | | 82 | |  |
| A*β*42/40 + pTau217/A*β*40 + pTau217/A*β*42 + pTau181/A*β*40 | 0.917 | | 132 | | 0.843 | | 121 | |  |
| A*β*42/40 + NfL + pTau217/A*β*40 + pTau217/A*β*42 + pTau181/A*β*40 | 0.917 | | 108 | | 0.886 | | 79 | |  |
| A*β*42/40 + pTau217 + NfL + pTau217/A*β*40 + pTau217/A*β*42 + pTau181/A*β*40 | 0.917 | | 108 | | 0.886 | | 79 | |  |
| A*β*42/40 + NfL + pTau217/A*β*40 + pTau181/A*β*40 | 0.917 | | 108 | | 0.886 | | 79 | |  |
| A*β*42/40 + pTau217 + NfL + pTau217/A*β*40 + pTau181/A*β*40 | 0.917 | | 108 | | 0.886 | | 79 | |  |
| A*β*42/40 + pTau217 + pTau217/A*β*42 + pTau181/A*β*40 | 0.916 | | 131 | | 0.866 | | 119 | |  |
| NfL + pTau217/A*β*42 | 0.915 | | 141 | | 0.727 | | 154 | |  |
| pTau217 + pTau217/A*β*40 | 0.915 | | 176 | | 0.728 | | 243 | |  |
| NfL + pTau217/A*β*40 | 0.915 | | 141 | | 0.73 | | 159 | |  |
| A*β*42/40 + pTau217 + NfL + pTau217/A*β*40 | 0.915 | | 118 | | 0.875 | | 88 | |  |
| pTau217 + NfL + pTau217/A*β*40 + pTau217/A*β*42 | 0.915 | | 141 | | 0.73 | | 152 | |  |
| A*β*42/40 + pTau217 + pTau217/A*β*40 + pTau181/A*β*40 | 0.915 | | 130 | | 0.864 | | 118 | |  |
| NfL + pTau217/A*β*40 + pTau217/A*β*42 | 0.915 | | 141 | | 0.73 | | 152 | |  |
| pTau217 + NfL + pTau217/A*β*40 | 0.915 | | 141 | | 0.732 | | 157 | |  |
| A*β*42/40 + NfL + pTau217/A*β*40 | 0.915 | | 118 | | 0.875 | | 88 | |  |
| A*β*42/40 + NfL + pTau217/A*β*42 | 0.915 | | 118 | | 0.867 | | 90 | |  |

| Combination | A+/T+ in BBM+ | n BBM+ | non-A+/T+ in BBM- | n BBM- |
| --- | --- | --- | --- | --- |
| pTau217 + NfL + pTau217/A*β*42 | 0.915 | 141 | 0.732 | 153 |
| A*β*42/40 + pTau217 + pTau217/A*β*40 + pTau217/A*β*42 + pTau181/A*β*40 | 0.915 | 130 | 0.864 | 118 |
| A*β*42/40 + pTau217 + NfL + pTau217/A*β*42 | 0.915 | 118 | 0.876 | 89 |
| A*β*42/40 + pTau217 + NfL + pTau217/A*β*40 + pTau217/A*β*42 | 0.915 | 118 | 0.875 | 88 |
| A*β*42/40 + NfL + pTau217/A*β*40 + pTau217/A*β*42 | 0.915 | 118 | 0.875 | 88 |
| pTau217 + pTau217/A*β*40 + pTau217/A*β*42 | 0.914 | 175 | 0.727 | 238 |
| A*β*42/40 + pTau217/A*β*40 + pTau217/A*β*42 | 0.913 | 149 | 0.836 | 134 |
| A*β*42/40 + pTau217/A*β*40 | 0.913 | 149 | 0.836 | 134 |
| pTau217/A*β*40 | 0.912 | 182 | 0.714 | 252 |
| A*β*42/40 + pTau217 + pTau217/A*β*40 | 0.911 | 146 | 0.86 | 129 |
| A*β*42/40 + pTau217/A*β*42 + pTau181/A*β*40 | 0.911 | 135 | 0.837 | 123 |
| A*β*42/40 + pTau217 + pTau217/A*β*40 + pTau217/A*β*42 | 0.911 | 146 | 0.86 | 129 |
| pTau217 + pTau217/A*β*42 | 0.91 | 177 | 0.728 | 239 |
| A*β*42/40 + pTau217 + NfL | 0.908 | 120 | 0.857 | 91 |
| pTau181 + NfL + pTau181/A*β*40 | 0.908 | 131 | 0.714 | 154 |
| A*β*42/40 + pTau217 + pTau217/A*β*42 | 0.905 | 148 | 0.862 | 130 |
| pTau217 + NfL | 0.905 | 148 | 0.712 | 163 |
| A*β*42/40 + pTau181 | 0.904 | 146 | 0.806 | 129 |
| pTau217 | 0.903 | 185 | 0.715 | 249 |
| A*β*42/40 + pTau217 | 0.9 | 150 | 0.848 | 132 |
| A*β*42/40 + pTau181 + NfL | 0.9 | 120 | 0.831 | 89 |
| A*β*42/40 + pTau181/A*β*40 | 0.898 | 147 | 0.795 | 132 |
| A*β*42/40 + NfL + pTau181/A*β*40 | 0.897 | 116 | 0.826 | 86 |
| pTau217/A*β*42 | 0.893 | 187 | 0.713 | 247 |
| NfL + pTau181/A*β*40 | 0.892 | 139 | 0.701 | 157 |
| pTau181 + pTau181/A*β*40 | 0.89 | 163 | 0.71 | 231 |
| A*β*42/40 + pTau217/A*β*42 | 0.885 | 156 | 0.831 | 136 |
| pTau181 + NfL | 0.872 | 149 | 0.691 | 165 |
| pTau181 | 0.864 | 184 | 0.684 | 250 |
| pTau181/A*β*40 | 0.863 | 182 | 0.679 | 252 |
| A*β*42/40 + NfL | 0.764 | 165 | 0.796 | 98 |
| A*β*42/40 | 0.697 | 267 | 0.689 | 167 |
| NfL | 0.675 | 234 | 0.6 | 200 |

Supplementary table 6 Cutoff combinations — Spectral (all 127 combinations). Prevalence of A^+^/T^+^ CSF profile in BBM-positive subgroups and prevalence of non-A^+^/T^+^ CSF profile in BBM-negative subgroups.

| Combination | A+/T+ in BBM+ | n BBM+ | non-A+/T+ in BBM- | n BBM- |
| --- | --- | --- | --- | --- |
| pTau217 + pTau181 + NfL + pTau217/A*β*40 + pTau181/A*β*40 | 0.952 | 104 | 0.742 | 159 |
| pTau181 + NfL + pTau217/A*β*42 + pTau181/A*β*40 | 0.952 | 104 | 0.736 | 159 |
| pTau181 + NfL + pTau217/A*β*40 + pTau181/A*β*40 | 0.952 | 104 | 0.744 | 160 |
| pTau217 + pTau181 + NfL + pTau217/A*β*42 + pTau181/A*β*40 | 0.951 | 103 | 0.739 | 157 |
| pTau181 + NfL + pTau217/A*β*40 + pTau217/A*β*42 + pTau181/A*β*40 | 0.951 | 102 | 0.745 | 157 |
| pTau217 + pTau181 + NfL + pTau217/A*β*40 + pTau217/A*β*42 + pTau181/A*β*40 | 0.951 | 102 | 0.744 | 156 |
| pTau217 + NfL + pTau217/A*β*40 + pTau181/A*β*40 | 0.945 | 109 | 0.738 | 160 |
| pTau217 + pTau181 + NfL + pTau181/A*β*40 | 0.944 | 108 | 0.735 | 162 |
| pTau181 + NfL + pTau217/A*β*42 | 0.944 | 108 | 0.733 | 165 |
| pTau181 + NfL + pTau217/A*β*40 | 0.944 | 108 | 0.738 | 168 |
| pTau217 + NfL + pTau217/A*β*42 + pTau181/A*β*40 | 0.944 | 108 | 0.734 | 158 |
| pTau217 + pTau181 + NfL + pTau217/A*β*42 | 0.944 | 107 | 0.736 | 163 |
| pTau217 + NfL + pTau217/A*β*40 + pTau217/A*β*42 + pTau181/A*β*40 | 0.944 | 107 | 0.739 | 157 |
| pTau217 + pTau181 + NfL + pTau217/A*β*40 | 0.944 | 108 | 0.737 | 167 |
| pTau181 + NfL + pTau217/A*β*40 + pTau217/A*β*42 | 0.943 | 106 | 0.742 | 163 |
| pTau217 + pTau181 + NfL + pTau217/A*β*40 + pTau217/A*β*42 | 0.943 | 106 | 0.741 | 162 |
| pTau217 + NfL + pTau217/A*β*40 | 0.941 | 119 | 0.716 | 176 |
| pTau217 + NfL + pTau217/A*β*42 | 0.94 | 117 | 0.719 | 171 |
| pTau217 + NfL + pTau217/A*β*40 + pTau217/A*β*42 | 0.94 | 116 | 0.724 | 170 |
| A*β*42/40 + pTau217 + NfL | 0.939 | 82 | 0.822 | 118 |
| A*β*42/40 + pTau217 + NfL + pTau217/A*β*42 | 0.938 | 81 | 0.829 | 117 |
| pTau181 + pTau217/A*β*40 + pTau181/A*β*40 | 0.938 | 128 | 0.733 | 225 |
| pTau217 + pTau181 + NfL | 0.938 | 113 | 0.725 | 171 |
| pTau217 + NfL + pTau181/A*β*40 | 0.938 | 113 | 0.73 | 163 |
| pTau181 + pTau217/A*β*42 + pTau181/A*β*40 | 0.938 | 130 | 0.724 | 225 |
| A*β*42/40 + pTau217 + NfL + pTau217/A*β*40 | 0.938 | 80 | 0.836 | 116 |
| A*β*42/40 + pTau217 + NfL + pTau217/A*β*40 + pTau217/A*β*42 | 0.938 | 80 | 0.836 | 116 |

| Combination | A+/T+ in BBM+ | n BBM+ | non-A+/T+ in BBM- | n BBM- |
| --- | --- | --- | --- | --- |
| pTau217 + pTau181 + pTau217/A*β*40 + pTau181/A*β*40 | 0.937 | 127 | 0.732 | 224 |
| pTau217 + pTau181 + pTau217/A*β*42 + pTau181/A*β*40 | 0.937 | 127 | 0.73 | 222 |
| NfL + pTau217/A*β*40 + pTau181/A*β*40 | 0.937 | 111 | 0.739 | 161 |
| NfL + pTau217/A*β*42 + pTau181/A*β*40 | 0.937 | 111 | 0.727 | 161 |
| pTau181 + pTau217/A*β*40 + pTau217/A*β*42 + pTau181/A*β*40 | 0.937 | 126 | 0.734 | 222 |
| pTau217 + pTau217/A*β*40 | 0.936 | 156 | 0.695 | 262 |
| pTau217 + pTau181 + pTau217/A*β*40 + pTau217/A*β*42 + pTau181/A*β*40 | 0.936 | 125 | 0.733 | 221 |
| NfL + pTau217/A*β*40 + pTau217/A*β*42 + pTau181/A*β*40 | 0.936 | 109 | 0.741 | 158 |
| A*β*42/40 + pTau217 + NfL + pTau217/A*β*42 + pTau181/A*β*40 | 0.935 | 77 | 0.833 | 108 |
| A*β*42/40 + pTau217 + pTau181 + NfL | 0.935 | 77 | 0.832 | 113 |
| A*β*42/40 + pTau217 + NfL + pTau181/A*β*40 | 0.935 | 77 | 0.833 | 108 |
| A*β*42/40 + pTau181 + NfL + pTau217/A*β*42 | 0.935 | 77 | 0.833 | 114 |
| pTau217 + pTau217/A*β*42 | 0.935 | 153 | 0.699 | 256 |
| pTau181 + pTau217/A*β*42 | 0.935 | 138 | 0.72 | 232 |
| pTau217 + NfL | 0.935 | 124 | 0.702 | 181 |
| NfL + pTau217/A*β*42 | 0.934 | 121 | 0.709 | 175 |
| A*β*42/40 + pTau217 + NfL + pTau217/A*β*40 + pTau217/A*β*42 + pTau181/A*β*40 | 0.934 | 76 | 0.841 | 107 |
| A*β*42/40 + pTau217 + NfL + pTau217/A*β*40 + pTau181/A*β*40 | 0.934 | 76 | 0.841 | 107 |
| A*β*42/40 + pTau181 + NfL + pTau217/A*β*42 + pTau181/A*β*40 | 0.934 | 76 | 0.835 | 109 |
| A*β*42/40 + pTau217 + pTau181 + NfL + pTau217/A*β*42 | 0.934 | 76 | 0.839 | 112 |
| pTau217 + pTau181 + pTau217/A*β*40 | 0.934 | 136 | 0.725 | 233 |
| pTau217 + pTau217/A*β*40 + pTau217/A*β*42 | 0.934 | 151 | 0.702 | 255 |
| pTau181 + pTau217/A*β*40 | 0.934 | 137 | 0.726 | 234 |
| NfL + pTau217/A*β*40 | 0.934 | 122 | 0.709 | 179 |
| A*β*42/40 + pTau181 + NfL + pTau217/A*β*40 | 0.933 | 75 | 0.848 | 112 |
| pTau217 + pTau181 + pTau217/A*β*42 | 0.933 | 135 | 0.725 | 229 |
| pTau217 + pTau217/A*β*40 + pTau181/A*β*40 | 0.933 | 135 | 0.719 | 235 |
| pTau217 + pTau217/A*β*42 + pTau181/A*β*40 | 0.933 | 135 | 0.717 | 233 |
| NfL + pTau217/A*β*40 + pTau217/A*β*42 | 0.933 | 119 | 0.721 | 172 |
| pTau181 + pTau217/A*β*40 + pTau217/A*β*42 | 0.933 | 134 | 0.729 | 229 |
| A*β*42/40 + pTau217 + pTau181 + NfL + pTau217/A*β*42 + pTau181/A*β*40 | 0.933 | 75 | 0.841 | 107 |
|  |  |  |  | |

| Combination | | A+/T+ in BBM+ | | n BBM+ | | non-A+/T+ in BBM- | | n BBM- | |  |
| --- | --- | --- | --- | --- | --- | --- | --- | --- | --- | --- |
| A*β*42/40 + pTau217 + pTau181 + NfL + pTau181/A*β*40 | | 0.933 | | 75 | | 0.841 | | 107 | |  |
| A*β*42/40 + pTau181 + NfL + pTau217/A*β*40 + pTau217/A*β*42 | | 0.933 | | 75 | | 0.848 | | 112 | |  |
| A*β*42/40 + pTau217 + pTau181 + NfL + pTau217/A*β*40 | | 0.933 | | 75 | | 0.847 | | 111 | |  |
| A*β*42/40 + pTau217 + pTau181 + NfL + pTau217/A*β*40 + pTau217/A*β*42 | | 0.933 | | 75 | | 0.847 | | 111 | |  |
| pTau217 + pTau181 + pTau181/A*β*40 | | 0.932 | | 133 | | 0.724 | | 228 | |  |
| A*β*42/40 + pTau217 + pTau181 + NfL + pTau217/A*β*40 + pTau217/A*β*42 +  pTau181/A*β*40 | | 0.932 | | 74 | | 0.849 | | 106 | |  |
| pTau217 + pTau181 + pTau217/A*β*40 + pTau217/A*β*42 | | 0.932 | | 133 | | 0.728 | | 228 | |  |
| pTau217 + pTau217/A*β*40 + pTau217/A*β*42 + pTau181/A*β*40 | | 0.932 | | 133 | | 0.72 | | 232 | |  |
| A*β*42/40 + pTau217 + pTau181 + NfL + pTau217/A*β*40 + pTau181/A*β*40 | | 0.932 | | 74 | | 0.849 | | 106 | |  |
| A*β*42/40 + pTau181 + NfL + pTau217/A*β*40 + pTau217/A*β*42 + pTau181/A*β*40 | | 0.932 | | 74 | | 0.85 | | 107 | |  |
| A*β*42/40 + pTau181 + NfL + pTau217/A*β*40 + pTau181/A*β*40 | | 0.932 | | 74 | | 0.85 | | 107 | |  |
| pTau217 + pTau181 | | 0.93 | | 143 | | 0.712 | | 240 | |  |
| pTau217 + pTau181/A*β*40 | | 0.929 | | 141 | | 0.711 | | 239 | |  |
| A*β*42/40 + NfL + pTau217/A*β*42 | | 0.929 | | 85 | | 0.817 | | 120 | |  |
| A*β*42/40 + NfL + pTau217/A*β*40 | | 0.928 | | 83 | | 0.838 | | 117 | |  |
| pTau217/A*β*40 + pTau181/A*β*40 | | 0.928 | | 139 | | 0.717 | | 237 | |  |
| A*β*42/40 + NfL + pTau217/A*β*40 + pTau217/A*β*42 | | 0.928 | | 83 | | 0.838 | | 117 | |  |
| pTau217/A*β*40 | | 0.927 | | 164 | | 0.681 | | 270 | |  |
| A*β*42/40 + pTau217 | | 0.927 | | 110 | | 0.801 | | 166 | |  |
| pTau217 | | 0.927 | | 164 | | 0.681 | | 270 | |  |
| pTau217/A*β*40 + pTau217/A*β*42 + pTau181/A*β*40 | | 0.927 | | 137 | | 0.718 | | 234 | |  |
| A*β*42/40 + pTau217 + pTau217/A*β*42 | | 0.926 | | 108 | | 0.806 | | 165 | |  |
| A*β*42/40 + pTau217 + pTau217/A*β*40 + pTau217/A*β*42 | | 0.925 | | 106 | | 0.811 | | 164 | |  |
| A*β*42/40 + pTau217 + pTau217/A*β*40 | | 0.925 | | 106 | | 0.811 | | 164 | |  |
| A*β*42/40 + NfL + pTau217/A*β*42 + pTau181/A*β*40 | | 0.925 | | 80 | | 0.82 | | 111 | |  |
| pTau217/A*β*40 + pTau217/A*β*42 | | 0.924 | | 158 | | 0.693 | | 261 | |  |
|  | Combination | | A+/T+ in BBM+ | | n BBM+ | | non-A+/T+ in BBM- | | n BBM- | |
|  | pTau217/A*β*42 + pTau181/A*β*40 | | 0.923 | | 143 | | 0.706 | | 238 | |
|  | A*β*42/40 + NfL + pTau217/A*β*40 + pTau181/A*β*40 | | 0.923 | | 78 | | 0.843 | | 108 | |
|  | A*β*42/40 + NfL + pTau217/A*β*40 + pTau217/A*β*42 + pTau181/A*β*40 | | 0.923 | | 78 | | 0.843 | | 108 | |
|  | A*β*42/40 + pTau181 + pTau217/A*β*42 | | 0.922 | | 102 | | 0.813 | | 155 | |
|  | A*β*42/40 + pTau217 + pTau181 | | 0.921 | | 101 | | 0.817 | | 153 | |
|  | A*β*42/40 + pTau217 + pTau181 + pTau217/A*β*42 | | 0.919 | | 99 | | 0.822 | | 152 | |
|  | A*β*42/40 + pTau217 + pTau181/A*β*40 | | 0.919 | | 99 | | 0.81 | | 153 | |
|  | A*β*42/40 + pTau181 + pTau217/A*β*40 | | 0.918 | | 98 | | 0.829 | | 152 | |
|  | A*β*42/40 + pTau181 + pTau217/A*β*42 + pTau181/A*β*40 | | 0.918 | | 98 | | 0.813 | | 150 | |
|  | A*β*42/40 + pTau217 + pTau181 + pTau217/A*β*40 + pTau217/A*β*42 | | 0.918 | | 97 | | 0.828 | | 151 | |
|  | A*β*42/40 + pTau181 + pTau217/A*β*40 + pTau217/A*β*42 | | 0.918 | | 98 | | 0.829 | | 152 | |
|  | A*β*42/40 + pTau217 + pTau217/A*β*42 + pTau181/A*β*40 | | 0.918 | | 98 | | 0.81 | | 153 | |
|  | A*β*42/40 + pTau217 + pTau181 + pTau217/A*β*40 | | 0.918 | | 97 | | 0.828 | | 151 | |
|  | A*β*42/40 + pTau217 + pTau181 + pTau181/A*β*40 | | 0.917 | | 96 | | 0.823 | | 147 | |
|  | A*β*42/40 + pTau217 + pTau217/A*β*40 + pTau181/A*β*40 | | 0.917 | | 96 | | 0.816 | | 152 | |
|  | A*β*42/40 + pTau217 + pTau217/A*β*40 + pTau217/A*β*42 + pTau181/A*β*40 | | 0.917 | | 96 | | 0.816 | | 152 | |
|  | A*β*42/40 + pTau217 + pTau181 + pTau217/A*β*42 + pTau181/A*β*40 | | 0.916 | | 95 | | 0.823 | | 147 | |
|  | A*β*42/40 + pTau181 + pTau217/A*β*40 + pTau181/A*β*40 | | 0.915 | | 94 | | 0.83 | | 147 | |
|  | A*β*42/40 + pTau181 + pTau217/A*β*40 + pTau217/A*β*42 + pTau181/A*β*40 | | 0.915 | | 94 | | 0.83 | | 147 | |
|  | A*β*42/40 + pTau217 + pTau181 + pTau217/A*β*40 + pTau181/A*β*40 | | 0.914 | | 93 | | 0.829 | | 146 | |
|  | A*β*42/40 + pTau217 + pTau181 + pTau217/A*β*40 + pTau217/A*β*42 +  pTau181/A*β*40 | | 0.914 | | 93 | | 0.829 | | 146 | |
|  | A*β*42/40 + pTau217/A*β*40 + pTau217/A*β*42 | | 0.911 | | 112 | | 0.804 | | 168 | |
|  | A*β*42/40 + pTau217/A*β*40 | | 0.911 | | 112 | | 0.804 | | 168 | |
|  | pTau217/A*β*42 | | 0.91 | | 167 | | 0.678 | | 267 | |

| Combination | A+/T+ in BBM+ | n BBM+ | non-A+/T+ in BBM- | n BBM- |
| --- | --- | --- | --- | --- |
| A*β*42/40 + pTau217/A*β*40 + pTau181/A*β*40 | 0.909 | 99 | 0.817 | 153 |
| A*β*42/40 + pTau217/A*β*40 + pTau217/A*β*42 + pTau181/A*β*40 | 0.909 | 99 | 0.817 | 153 |
| pTau181 + NfL + pTau181/A*β*40 | 0.907 | 118 | 0.71 | 169 |
| A*β*42/40 + pTau217/A*β*42 + pTau181/A*β*40 | 0.905 | 105 | 0.796 | 157 |
| A*β*42/40 + pTau181 + NfL + pTau181/A*β*40 | 0.902 | 82 | 0.82 | 111 |
| A*β*42/40 + pTau181 + pTau181/A*β*40 | 0.898 | 108 | 0.792 | 154 |
| NfL + pTau181/A*β*40 | 0.897 | 126 | 0.686 | 175 |
| A*β*42/40 + NfL + pTau181/A*β*40 | 0.897 | 87 | 0.798 | 114 |
| A*β*42/40 + pTau217/A*β*42 | 0.893 | 121 | 0.776 | 174 |
| A*β*42/40 + pTau181 | 0.89 | 118 | 0.77 | 165 |
| A*β*42/40 + pTau181/A*β*40 | 0.889 | 117 | 0.764 | 165 |
| A*β*42/40 + pTau181 + NfL | 0.888 | 89 | 0.798 | 119 |
| pTau181 + pTau181/A*β*40 | 0.888 | 152 | 0.689 | 241 |
| pTau181 + NfL | 0.872 | 133 | 0.691 | 181 |
| pTau181 | 0.867 | 173 | 0.663 | 261 |
| pTau181/A*β*40 | 0.86 | 172 | 0.656 | 262 |
| A*β*42/40 + NfL | 0.789 | 123 | 0.754 | 130 |
| A*β*42/40 | 0.724 | 214 | 0.623 | 220 |
| NfL | 0.695 | 213 | 0.593 | 221 |

Supplementary table 7 Cutoff combinations — Birch (all 127 combinations). Prevalence of A^+^/T^+^ CSF profile in BBM-positive subgroups and prevalence of non-A^+^/T^+^ CSF profile in BBM-negative subgroups.

| Combination | A+/T+ in BBM+ | n BBM+ | non-A+/T+ in BBM- | n BBM- |
| --- | --- | --- | --- | --- |
| pTau181 + pTau217/A*β*42 + pTau181/A*β*40 | 0.949 | 118 | 0.725 | 229 |
| pTau181 + pTau217/A*β*40 + pTau217/A*β*42 + pTau181/A*β*40 | 0.949 | 117 | 0.731 | 227 |
| pTau217 + pTau181 + pTau217/A*β*42 + pTau181/A*β*40 | 0.948 | 115 | 0.73 | 226 |
| pTau181 + NfL + pTau217/A*β*40 + pTau181/A*β*40 | 0.948 | 96 | 0.735 | 166 |
| pTau217 + pTau181 + NfL + pTau217/A*β*40 + pTau181/A*β*40 | 0.948 | 96 | 0.733 | 165 |
| pTau217 + pTau181 + pTau217/A*β*40 + pTau217/A*β*42 + pTau181/A*β*40 | 0.948 | 115 | 0.733 | 225 |
| pTau181 + NfL + pTau217/A*β*42 + pTau181/A*β*40 | 0.946 | 93 | 0.727 | 165 |
| pTau181 + NfL + pTau217/A*β*40 + pTau217/A*β*42 + pTau181/A*β*40 | 0.946 | 93 | 0.736 | 163 |

| Combination | A+/T+ in BBM+ | n BBM+ | non-A+/T+ in BBM- | n BBM- |
| --- | --- | --- | --- | --- |
| pTau217 + pTau181 + NfL + pTau217/A*β*42 + pTau181/A*β*40 | 0.946 | 93 | 0.73 | 163 |
| pTau217 + pTau181 + NfL + pTau217/A*β*40 + pTau217/A*β*42 + pTau181/A*β*40 | 0.946 | 93 | 0.735 | 162 |
| pTau181 + pTau217/A*β*40 + pTau217/A*β*42 | 0.945 | 127 | 0.725 | 233 |
| pTau181 + pTau217/A*β*42 | 0.945 | 128 | 0.719 | 235 |
| pTau217 + pTau181 + pTau217/A*β*42 | 0.944 | 125 | 0.724 | 232 |
| pTau217 + pTau181 + pTau217/A*β*40 + pTau217/A*β*42 | 0.944 | 125 | 0.727 | 231 |
| pTau181 + NfL + pTau217/A*β*40 | 0.942 | 103 | 0.728 | 173 |
| pTau181 + pTau217/A*β*40 + pTau181/A*β*40 | 0.942 | 121 | 0.727 | 231 |
| pTau217 + pTau181 + NfL + pTau217/A*β*40 | 0.942 | 103 | 0.727 | 172 |
| pTau217 + pTau181 + pTau217/A*β*40 + pTau181/A*β*40 | 0.941 | 119 | 0.729 | 229 |
| NfL + pTau217/A*β*40 + pTau181/A*β*40 | 0.941 | 101 | 0.731 | 167 |
| pTau217 + pTau181 + NfL + pTau181/A*β*40 | 0.94 | 100 | 0.726 | 168 |
| pTau217 + NfL + pTau217/A*β*40 + pTau181/A*β*40 | 0.94 | 100 | 0.729 | 166 |
| pTau217 + pTau181 + NfL + pTau217/A*β*42 | 0.94 | 100 | 0.726 | 168 |
| pTau181 + NfL + pTau217/A*β*40 + pTau217/A*β*42 | 0.94 | 100 | 0.732 | 168 |
| pTau181 + NfL + pTau217/A*β*42 | 0.94 | 100 | 0.724 | 170 |
| pTau217 + pTau181 + NfL + pTau217/A*β*40 + pTau217/A*β*42 | 0.94 | 100 | 0.731 | 167 |
| pTau181 + pTau217/A*β*40 | 0.939 | 132 | 0.72 | 239 |
| NfL + pTau217/A*β*42 + pTau181/A*β*40 | 0.939 | 98 | 0.719 | 167 |
| NfL + pTau217/A*β*40 + pTau217/A*β*42 + pTau181/A*β*40 | 0.939 | 98 | 0.732 | 164 |
| pTau217/A*β*40 + pTau217/A*β*42 + pTau181/A*β*40 | 0.938 | 128 | 0.712 | 240 |
| pTau217 + pTau181 + pTau217/A*β*40 | 0.938 | 130 | 0.722 | 237 |
| pTau217 + NfL + pTau217/A*β*40 | 0.938 | 112 | 0.703 | 182 |
| pTau217 + NfL + pTau217/A*β*42 + pTau181/A*β*40 | 0.938 | 97 | 0.726 | 164 |
| pTau217 + NfL + pTau217/A*β*40 + pTau217/A*β*42 + pTau181/A*β*40 | 0.938 | 97 | 0.73 | 163 |
| pTau217 + pTau217/A*β*40 + pTau217/A*β*42 | 0.937 | 143 | 0.688 | 260 |
| pTau217 + pTau217/A*β*42 | 0.937 | 143 | 0.686 | 261 |
| pTau217 + NfL + pTau217/A*β*42 | 0.936 | 109 | 0.706 | 177 |
| A*β*42/40 + pTau181 + pTau217/A*β*42 | 0.936 | 94 | 0.808 | 156 |
| pTau217 + NfL + pTau217/A*β*40 + pTau217/A*β*42 | 0.936 | 109 | 0.71 | 176 |
|  |  |  |  | |

| Combination | A+/T+ in BBM+ | n BBM+ | non-A+/T+ in BBM- | n BBM- |
| --- | --- | --- | --- | --- |
| pTau217 + pTau181 + pTau181/A*β*40 | 0.935 | 123 | 0.722 | 234 |
| A*β*42/40 + pTau217 + NfL | 0.935 | 77 | 0.81 | 121 |
| pTau217 + pTau181 + NfL | 0.935 | 108 | 0.708 | 178 |
| A*β*42/40 + pTau181 + pTau217/A*β*40 + pTau217/A*β*42 | 0.935 | 93 | 0.818 | 154 |
| pTau217 + pTau217/A*β*40 + pTau217/A*β*42 + pTau181/A*β*40 | 0.934 | 122 | 0.717 | 237 |
| A*β*42/40 + pTau217 + pTau181 + pTau217/A*β*40 + pTau217/A*β*42 | 0.934 | 91 | 0.822 | 152 |
| pTau217 + pTau217/A*β*42 + pTau181/A*β*40 | 0.934 | 122 | 0.714 | 238 |
| A*β*42/40 + pTau217 + pTau181 + pTau217/A*β*42 | 0.934 | 91 | 0.817 | 153 |
| A*β*42/40 + pTau217 + NfL + pTau217/A*β*40 + pTau217/A*β*42 | 0.933 | 75 | 0.831 | 118 |
| pTau217 + NfL + pTau181/A*β*40 | 0.933 | 104 | 0.722 | 169 |
| A*β*42/40 + pTau217 + NfL + pTau217/A*β*42 | 0.933 | 75 | 0.824 | 119 |
| pTau217 + pTau217/A*β*40 | 0.933 | 149 | 0.682 | 267 |
| pTau217 + pTau181 | 0.933 | 135 | 0.703 | 246 |
| A*β*42/40 + pTau217 + NfL + pTau217/A*β*40 | 0.933 | 75 | 0.831 | 118 |
| pTau217 + NfL | 0.932 | 118 | 0.679 | 190 |
| A*β*42/40 + pTau181 + pTau217/A*β*42 + pTau181/A*β*40 | 0.932 | 88 | 0.809 | 152 |
| A*β*42/40 + pTau217 + pTau181 + NfL | 0.932 | 73 | 0.812 | 117 |
| pTau217/A*β*40 + pTau181/A*β*40 | 0.932 | 132 | 0.709 | 244 |
| A*β*42/40 + pTau217 + pTau217/A*β*40 + pTau217/A*β*42 | 0.931 | 101 | 0.804 | 163 |
| A*β*42/40 + pTau181 + pTau217/A*β*40 + pTau217/A*β*42 + pTau181/A*β*40 | 0.931 | 87 | 0.82 | 150 |
| pTau217/A*β*42 + pTau181/A*β*40 | 0.931 | 131 | 0.704 | 243 |
| A*β*42/40 + pTau217 + pTau217/A*β*42 | 0.931 | 101 | 0.799 | 164 |
| NfL + pTau217/A*β*40 | 0.93 | 115 | 0.705 | 183 |
| A*β*42/40 + pTau217 + pTau181 + NfL + pTau217/A*β*40 | 0.93 | 71 | 0.833 | 114 |
| A*β*42/40 + pTau217 + pTau181 + NfL + pTau217/A*β*40 + pTau217/A*β*42 | 0.93 | 71 | 0.833 | 114 |
| A*β*42/40 + pTau181 + NfL + pTau217/A*β*42 | 0.93 | 71 | 0.821 | 117 |
| A*β*42/40 + pTau217 + pTau181 + NfL + pTau217/A*β*42 | 0.93 | 71 | 0.826 | 115 |
| A*β*42/40 + pTau181 + NfL + pTau217/A*β*40 + pTau217/A*β*42 | 0.93 | 71 | 0.835 | 115 |
| A*β*42/40 + pTau181 + NfL + pTau217/A*β*40 | 0.93 | 71 | 0.835 | 115 |

| Combination | A+/T+ in BBM+ | n BBM+ | non-A+/T+ in BBM- | n BBM- |
| --- | --- | --- | --- | --- |
| NfL + pTau217/A*β*42 | 0.929 | 113 | 0.702 | 181 |
| pTau217 + pTau217/A*β*40 + pTau181/A*β*40 | 0.929 | 126 | 0.714 | 241 |
| A*β*42/40 + pTau217 + pTau181 + pTau217/A*β*42 + pTau181/A*β*40 | 0.929 | 85 | 0.819 | 149 |
| A*β*42/40 + pTau217 + pTau181 + pTau217/A*β*40 + pTau217/A*β*42 +  pTau181/A*β*40 | 0.929 | 85 | 0.824 | 148 |
| A*β*42/40 + pTau217 + NfL + pTau181/A*β*40 | 0.929 | 70 | 0.829 | 111 |
| NfL + pTau217/A*β*40 + pTau217/A*β*42 | 0.929 | 112 | 0.712 | 177 |
| A*β*42/40 + NfL + pTau217/A*β*42 + pTau181/A*β*40 | 0.928 | 69 | 0.816 | 114 |
| A*β*42/40 + NfL + pTau217/A*β*40 + pTau181/A*β*40 | 0.928 | 69 | 0.838 | 111 |
| A*β*42/40 + pTau217 + pTau181 + NfL + pTau181/A*β*40 | 0.928 | 69 | 0.829 | 111 |
| A*β*42/40 + NfL + pTau217/A*β*40 + pTau217/A*β*42 + pTau181/A*β*40 | 0.928 | 69 | 0.838 | 111 |
| pTau217/A*β*40 + pTau217/A*β*42 | 0.928 | 153 | 0.678 | 267 |
| A*β*42/40 + pTau217 + NfL + pTau217/A*β*42 + pTau181/A*β*40 | 0.926 | 68 | 0.829 | 111 |
| A*β*42/40 + pTau181 + pTau217/A*β*40 | 0.926 | 94 | 0.818 | 154 |
| A*β*42/40 + pTau217 + pTau181 | 0.926 | 94 | 0.806 | 155 |
| A*β*42/40 + pTau217 + NfL + pTau217/A*β*40 + pTau181/A*β*40 | 0.926 | 68 | 0.836 | 110 |
| A*β*42/40 + pTau217 + NfL + pTau217/A*β*40 + pTau217/A*β*42 + pTau181/A*β*40 | 0.926 | 68 | 0.836 | 110 |
| A*β*42/40 + pTau217 + pTau181 + NfL + pTau217/A*β*42 + pTau181/A*β*40 | 0.925 | 67 | 0.829 | 111 |
| A*β*42/40 + pTau217 + pTau181 + NfL + pTau217/A*β*40 + pTau181/A*β*40 | 0.925 | 67 | 0.836 | 110 |
| A*β*42/40 + pTau181 + NfL + pTau217/A*β*40 + pTau181/A*β*40 | 0.925 | 67 | 0.838 | 111 |
| A*β*42/40 + pTau181 + NfL + pTau217/A*β*42 + pTau181/A*β*40 | 0.925 | 67 | 0.823 | 113 |
| A*β*42/40 + pTau181 + NfL + pTau217/A*β*40 + pTau217/A*β*42 + pTau181/A*β*40 | 0.925 | 67 | 0.838 | 111 |
| A*β*42/40 + pTau217 + pTau181 + NfL + pTau217/A*β*40 + pTau217/A*β*42 +  pTau181/A*β*40 | 0.925 | 67 | 0.836 | 110 |
| pTau217/A*β*40 | 0.925 | 160 | 0.672 | 274 |
| A*β*42/40 + NfL + pTau217/A*β*42 | 0.924 | 79 | 0.811 | 122 |

| Combination | | A+/T+ in BBM+ | | n BBM+ | | non-A+/T+ in BBM- | | n BBM- | |  |
| --- | --- | --- | --- | --- | --- | --- | --- | --- | --- | --- |
| A*β*42/40 + pTau217 + pTau181 + pTau217/A*β*40 | | 0.924 | | 92 | | 0.822 | | 152 | |  |
| A*β*42/40 + pTau217/A*β*40 + pTau217/A*β*42 + pTau181/A*β*40 | | 0.924 | | 92 | | 0.805 | | 154 | |  |
| A*β*42/40 + NfL + pTau217/A*β*40 | | 0.923 | | 78 | | 0.832 | | 119 | |  |
| pTau217 | | 0.923 | | 156 | | 0.662 | | 278 | |  |
| A*β*42/40 + NfL + pTau217/A*β*40 + pTau217/A*β*42 | | 0.923 | | 78 | | 0.832 | | 119 | |  |
| pTau217 + pTau181/A*β*40 | | 0.923 | | 130 | | 0.707 | | 246 | |  |
| A*β*42/40 + pTau217 | | 0.923 | | 104 | | 0.789 | | 166 | |  |
| A*β*42/40 + NfL + pTau181/A*β*40 | | 0.922 | | 77 | | 0.795 | | 117 | |  |
| A*β*42/40 + pTau217 + pTau217/A*β*40 | | 0.922 | | 102 | | 0.804 | | 163 | |  |
| A*β*42/40 + pTau217 + pTau181 + pTau181/A*β*40 | | 0.92 | | 88 | | 0.819 | | 149 | |  |
| A*β*42/40 + pTau181 + pTau217/A*β*40 + pTau181/A*β*40 | | 0.92 | | 88 | | 0.82 | | 150 | |  |
| A*β*42/40 + pTau181 + pTau181/A*β*40 | | 0.92 | | 100 | | 0.788 | | 156 | |  |
| A*β*42/40 + pTau217 + pTau217/A*β*40 + pTau217/A*β*42 + pTau181/A*β*40 | | 0.92 | | 88 | | 0.815 | | 151 | |  |
| A*β*42/40 + pTau217 + pTau217/A*β*42 + pTau181/A*β*40 | | 0.92 | | 88 | | 0.809 | | 152 | |  |
| A*β*42/40 + pTau217 + pTau181 + pTau217/A*β*40 + pTau181/A*β*40 | | 0.919 | | 86 | | 0.824 | | 148 | |  |
| A*β*42/40 + pTau181 + NfL + pTau181/A*β*40 | | 0.919 | | 74 | | 0.809 | | 115 | |  |
| A*β*42/40 + pTau217/A*β*40 + pTau217/A*β*42 | | 0.917 | | 109 | | 0.792 | | 168 | |  |
| pTau181 + NfL + pTau181/A*β*40 | | 0.917 | | 109 | | 0.699 | | 176 | |  |
| A*β*42/40 + pTau217/A*β*42 + pTau181/A*β*40 | | 0.916 | | 95 | | 0.79 | | 157 | |  |
| A*β*42/40 + pTau217/A*β*40 + pTau181/A*β*40 | | 0.914 | | 93 | | 0.805 | | 154 | |  |
| NfL + pTau181/A*β*40 | | 0.913 | | 115 | | 0.68 | | 181 | |  |
| A*β*42/40 + pTau217 + pTau181/A*β*40 | | 0.912 | | 91 | | 0.809 | | 152 | |  |
| pTau217/A*β*42 | | 0.912 | | 160 | | 0.664 | | 274 | |  |
| A*β*42/40 + pTau217 + pTau217/A*β*40 + pTau181/A*β*40 | | 0.91 | | 89 | | 0.815 | | 151 | |  |
| A*β*42/40 + pTau217/A*β*40 | | 0.909 | | 110 | | 0.792 | | 168 | |  |
| A*β*42/40 + pTau181/A*β*40 | | 0.908 | | 109 | | 0.758 | | 165 | |  |
| pTau181 + pTau181/A*β*40 | | 0.902 | | 143 | | 0.685 | | 248 | |  |
| A*β*42/40 + pTau217/A*β*42 | | 0.897 | | 116 | | 0.764 | | 174 | |  |
| A*β*42/40 + pTau181 | | 0.896 | | 115 | | 0.765 | | 166 | |  |
| A*β*42/40 + pTau181 + NfL | | 0.884 | | 86 | | 0.787 | | 122 | |  |
| pTau181 + NfL | | 0.883 | | 128 | | 0.677 | | 189 | |  |
| pTau181 | | 0.88 | | 167 | | 0.659 | | 267 | |  |
|  | Combination | | A+/T+ in BBM+ | | n BBM+ | | non-A+/T+ in BBM- | | n BBM- | |
|  | pTau181/A*β*40 | | 0.877 | | 162 | | 0.647 | | 272 | |
|  | A*β*42/40 + NfL | | 0.775 | | 120 | | 0.75 | | 132 | |
|  | A*β*42/40 | | 0.718 | | 216 | | 0.619 | | 218 | |
|  | NfL | | 0.694 | | 206 | | 0.583 | | 228 | |

# Supplementary Material 2

A supplementary analysis was performed by adding the plasma pTau181/A*β*42 ratio as an eighth biomarker, yielding 2^8^−1=255 possible combinations per algorithm. The resulting combination performances were 376 largely consistent with the seven-biomarker analysis, confirming that the inclusion of pTau181/A*β*42 did not substantially improve or alter the diagnostic accuracy of the biomarker combinations.

Supplementary table 8	Cutoff combinations — GMM (all 255 combinations, eight biomarkers). Prevalence of A^+^/T^+^ CSF profile in BBM-positive subgroups and prevalence of non-A^+^/T^+^ CSF profile in BBM-negative subgroups.

| Combination | A+/T+ in BBM+ | n BBM+ | non-A+/T+ in BBM- | n BBM- |
| --- | --- | --- | --- | --- |
| pTau181 + pTau217/A*β*40 + pTau181/A*β*40 + pTau181/A*β*42 | 0.926 | 175 | 0.865 | 155 |
| pTau181 + pTau217/A*β*40 + pTau217/A*β*42 + pTau181/A*β*40 + pTau181/A*β*42 | 0.925 | 174 | 0.87 | 154 |
| pTau217 + pTau181 + pTau217/A*β*40 + pTau181/A*β*40 + pTau181/A*β*42 | 0.925 | 174 | 0.87 | 154 |
| pTau217 + pTau181 + pTau217/A*β*42 + pTau181/A*β*40 + pTau181/A*β*42 | 0.925 | 174 | 0.876 | 153 |
| pTau217 + pTau181 + pTau217/A*β*40 + pTau217/A*β*42 + pTau181/A*β*40 +  pTau181/A*β*42 | 0.925 | 174 | 0.876 | 153 |
| pTau217 + pTau181 + NfL + pTau217/A*β*40 + pTau181/A*β*40 + pTau181/A*β*42 | 0.924 | 144 | 0.857 | 105 |
| pTau181 + pTau217/A*β*40 + pTau181/A*β*40 | 0.924 | 184 | 0.869 | 160 |
| pTau181 + NfL + pTau217/A*β*40 + pTau217/A*β*42 + pTau181/A*β*40 +  pTau181/A*β*42 | 0.924 | 144 | 0.857 | 105 |
| pTau217 + pTau181 + NfL + pTau217/A*β*42 + pTau181/A*β*40 + pTau181/A*β*42 | 0.924 | 144 | 0.857 | 105 |
| pTau217 + pTau181 + NfL + pTau217/A*β*40 + pTau217/A*β*42 + pTau181/A*β*40 +  pTau181/A*β*42 | 0.924 | 144 | 0.857 | 105 |
| pTau181 + NfL + pTau217/A*β*40 + pTau181/A*β*40 + pTau181/A*β*42 | 0.924 | 144 | 0.857 | 105 |

| Combination | A+/T+ in BBM+ | n BBM+ | non-A+/T+ in BBM- | n BBM- |
| --- | --- | --- | --- | --- |
| pTau181 + pTau217/A*β*40 + pTau217/A*β*42 | 0.923 | 194 | 0.876 | 161 |
| pTau181 + pTau217/A*β*40 + pTau181/A*β*42 | 0.923 | 182 | 0.865 | 156 |
| pTau217 + pTau181 + pTau217/A*β*42 | 0.923 | 194 | 0.877 | 162 |
| pTau181 + pTau217/A*β*40 | 0.923 | 196 | 0.873 | 166 |
| pTau217 + pTau181 + pTau217/A*β*40 + pTau181/A*β*42 | 0.923 | 181 | 0.871 | 155 |
| pTau181 + pTau217/A*β*40 + pTau217/A*β*42 + pTau181/A*β*42 | 0.923 | 181 | 0.871 | 155 |
| pTau181 + pTau217/A*β*40 + pTau217/A*β*42 + pTau181/A*β*40 | 0.923 | 183 | 0.872 | 156 |
| pTau217 + pTau181 + pTau217/A*β*42 + pTau181/A*β*42 | 0.923 | 181 | 0.871 | 155 |
| pTau217 + pTau181 + pTau217/A*β*42 + pTau181/A*β*40 | 0.923 | 183 | 0.877 | 155 |
| pTau217 + pTau181 + pTau217/A*β*40 + pTau181/A*β*40 | 0.923 | 183 | 0.874 | 159 |
| pTau217 + pTau181 + pTau217/A*β*40 | 0.923 | 195 | 0.879 | 165 |
| pTau217/A*β*40 + pTau217/A*β*42 + pTau181/A*β*40 + pTau181/A*β*42 | 0.923 | 181 | 0.867 | 166 |
| pTau217 + pTau181 + pTau217/A*β*40 + pTau217/A*β*42 | 0.923 | 194 | 0.881 | 160 |
| pTau217 + pTau181 + pTau217/A*β*40 + pTau217/A*β*42 + pTau181/A*β*42 | 0.923 | 181 | 0.877 | 154 |
| pTau217 + pTau181 + pTau217/A*β*40 + pTau217/A*β*42 + pTau181/A*β*40 | 0.923 | 183 | 0.877 | 155 |
| A*β*42/40 + pTau217 + pTau181 + pTau217/A*β*40 + pTau217/A*β*42 +  pTau181/A*β*40 | 0.922 | 153 | 0.948 | 97 |
| A*β*42/40 + pTau181 + pTau217/A*β*40 + pTau181/A*β*40 | 0.922 | 153 | 0.948 | 97 |
| A*β*42/40 + pTau217 + pTau181 + pTau217/A*β*42 + pTau181/A*β*40 +  pTau181/A*β*42 | 0.922 | 153 | 0.948 | 97 |
| pTau217 + pTau217/A*β*40 + pTau217/A*β*42 + pTau181/A*β*40 + pTau181/A*β*42 | 0.922 | 180 | 0.872 | 164 |
| A*β*42/40 + pTau217 + pTau181 + pTau181/A*β*40 | 0.922 | 153 | 0.939 | 98 |
| A*β*42/40 + pTau181 + pTau217/A*β*40 + pTau181/A*β*40 + pTau181/A*β*42 | 0.922 | 153 | 0.948 | 97 |
| A*β*42/40 + pTau217 + pTau181 + pTau181/A*β*40 + pTau181/A*β*42 | 0.922 | 153 | 0.939 | 98 |

| Combination | A+/T+ in BBM+ | n BBM+ | non-A+/T+ in BBM- | n BBM- |
| --- | --- | --- | --- | --- |
| A*β*42/40 + pTau217 + pTau181 + pTau217/A*β*42 + pTau181/A*β*40 | 0.922 | 153 | 0.948 | 97 |
| A*β*42/40 + pTau217 + pTau181 + pTau217/A*β*40 + pTau181/A*β*40 | 0.922 | 153 | 0.948 | 97 |
| pTau217 + pTau217/A*β*40 + pTau181/A*β*40 + pTau181/A*β*42 | 0.922 | 180 | 0.867 | 165 |
| A*β*42/40 + pTau181 + pTau217/A*β*40 + pTau217/A*β*42 + pTau181/A*β*40 | 0.922 | 153 | 0.948 | 97 |
| pTau217 + pTau217/A*β*42 + pTau181/A*β*40 + pTau181/A*β*42 | 0.922 | 180 | 0.872 | 164 |
| A*β*42/40 + pTau217 + pTau181 + pTau217/A*β*40 + pTau217/A*β*42 + pTau181/A*β*40 + pTau181/A*β*42 | 0.922 | 153 | 0.948 | 97 |
| A*β*42/40 + pTau217 + pTau181 + pTau217/A*β*40 + pTau181/A*β*40 +  pTau181/A*β*42 | 0.922 | 153 | 0.948 | 97 |
| A*β*42/40 + pTau181 + pTau217/A*β*40 + pTau217/A*β*42 + pTau181/A*β*40 +  pTau181/A*β*42 | 0.922 | 153 | 0.948 | 97 |
| pTau217/A*β*40 + pTau217/A*β*42 + pTau181/A*β*40 | 0.921 | 191 | 0.866 | 172 |
| pTau217 + pTau217/A*β*42 + pTau181/A*β*40 | 0.921 | 190 | 0.871 | 170 |
| pTau217 + pTau217/A*β*40 + pTau217/A*β*42 + pTau181/A*β*40 | 0.921 | 190 | 0.871 | 170 |
| pTau217 + pTau217/A*β*40 + pTau181/A*β*40 | 0.921 | 190 | 0.868 | 174 |
| A*β*42/40 + pTau181 + pTau217/A*β*40 | 0.92 | 162 | 0.949 | 99 |
| A*β*42/40 + pTau217 + pTau181 | 0.92 | 162 | 0.931 | 102 |
| A*β*42/40 + pTau217 + pTau181 + pTau217/A*β*40 | 0.92 | 162 | 0.949 | 99 |
| A*β*42/40 + pTau217 + pTau181 + NfL + pTau181/A*β*40 + pTau181/A*β*42 | 0.92 | 125 | 0.929 | 70 |
| A*β*42/40 + pTau217 + pTau181 + NfL + pTau217/A*β*40 + pTau217/A*β*42 + pTau181/A*β*40 + pTau181/A*β*42 | 0.92 | 125 | 0.942 | 69 |
| A*β*42/40 + pTau181 + NfL + pTau217/A*β*40 + pTau217/A*β*42 + pTau181/A*β*40 | 0.92 | 125 | 0.942 | 69 |
| A*β*42/40 + pTau217 + pTau181 + NfL + pTau217/A*β*40 + pTau217/A*β*42 +  pTau181/A*β*40 | 0.92 | 125 | 0.942 | 69 |
| pTau217 + pTau181 + NfL + pTau217/A*β*40 + pTau217/A*β*42 + pTau181/A*β*40 | 0.92 | 150 | 0.858 | 106 |

| Combination | A+/T+ in BBM+ | n BBM+ | non-A+/T+ in BBM- | n BBM- |
| --- | --- | --- | --- | --- |
| A*β*42/40 + pTau181 + NfL + pTau217/A*β*40 + pTau181/A*β*40 + pTau181/A*β*42 | 0.92 | 125 | 0.942 | 69 |
| pTau217 + pTau181 + NfL + pTau217/A*β*40 + pTau181/A*β*40 | 0.92 | 150 | 0.861 | 108 |
| A*β*42/40 + pTau181 + NfL + pTau217/A*β*40 + pTau217/A*β*42 + pTau181/A*β*40 +  pTau181/A*β*42 | 0.92 | 125 | 0.942 | 69 |
| pTau217 + pTau181 + NfL + pTau217/A*β*42 + pTau181/A*β*40 | 0.92 | 150 | 0.858 | 106 |
| A*β*42/40 + pTau217 + pTau181 + NfL + pTau217/A*β*42 + pTau181/A*β*40 | 0.92 | 125 | 0.942 | 69 |
| A*β*42/40 + pTau217 + pTau181 + NfL + pTau217/A*β*40 + pTau181/A*β*40 | 0.92 | 125 | 0.942 | 69 |
| A*β*42/40 + pTau217 + pTau181 + NfL + pTau217/A*β*42 + pTau181/A*β*40 +  pTau181/A*β*42 | 0.92 | 125 | 0.942 | 69 |
| A*β*42/40 + pTau217 + pTau181 + NfL + pTau217/A*β*40 + pTau181/A*β*40 +  pTau181/A*β*42 | 0.92 | 125 | 0.942 | 69 |
| A*β*42/40 + pTau181 + pTau217/A*β*40 + pTau217/A*β*42 | 0.92 | 162 | 0.949 | 99 |
| A*β*42/40 + pTau217 + pTau181 + pTau217/A*β*42 | 0.92 | 162 | 0.941 | 101 |
| pTau181 + NfL + pTau217/A*β*40 + pTau217/A*β*42 + pTau181/A*β*40 | 0.92 | 150 | 0.858 | 106 |
| A*β*42/40 + pTau217 + pTau181 + NfL + pTau181/A*β*40 | 0.92 | 125 | 0.929 | 70 |
| pTau217 + pTau181 + pTau181/A*β*40 + pTau181/A*β*42 | 0.92 | 175 | 0.854 | 157 |
| A*β*42/40 + pTau181 + NfL + pTau217/A*β*40 + pTau181/A*β*40 | 0.92 | 125 | 0.942 | 69 |
| pTau181 + pTau217/A*β*42 + pTau181/A*β*40 + pTau181/A*β*42 | 0.92 | 175 | 0.865 | 156 |
| pTau181 + NfL + pTau217/A*β*40 + pTau181/A*β*40 | 0.92 | 150 | 0.861 | 108 |
| A*β*42/40 + pTau217 + pTau181 + pTau217/A*β*40 + pTau217/A*β*42 | 0.92 | 162 | 0.949 | 99 |
| pTau217 + pTau181 + NfL + pTau217/A*β*42 + pTau181/A*β*42 | 0.919 | 149 | 0.85 | 107 |
| A*β*42/40 + pTau181 + pTau217/A*β*40 + pTau217/A*β*42 + pTau181/A*β*42 | 0.919 | 160 | 0.949 | 98 |

| Combination | A+/T+ in BBM+ | n BBM+ | non-A+/T+ in BBM- | n BBM- |
| --- | --- | --- | --- | --- |
| pTau217 + pTau181 + NfL + pTau217/A*β*40 + pTau181/A*β*42 | 0.919 | 149 | 0.858 | 106 |
| pTau181 + NfL + pTau217/A*β*40 + pTau217/A*β*42 + pTau181/A*β*42 | 0.919 | 149 | 0.858 | 106 |
| A*β*42/40 + pTau217 + pTau181 + pTau217/A*β*40 + pTau217/A*β*42 +  pTau181/A*β*42 | 0.919 | 160 | 0.949 | 98 |
| pTau217 + pTau181 + NfL + pTau217/A*β*40 + pTau217/A*β*42 + pTau181/A*β*42 | 0.919 | 149 | 0.858 | 106 |
| pTau181 + NfL + pTau217/A*β*40 + pTau181/A*β*42 | 0.919 | 149 | 0.858 | 106 |
| A*β*42/40 + pTau217 + pTau181 + pTau217/A*β*40 + pTau181/A*β*42 | 0.919 | 160 | 0.949 | 98 |
| A*β*42/40 + pTau217 + pTau181 + pTau217/A*β*42 + pTau181/A*β*42 | 0.919 | 160 | 0.939 | 99 |
| A*β*42/40 + pTau181 + pTau217/A*β*40 + pTau181/A*β*42 | 0.919 | 160 | 0.949 | 98 |
| A*β*42/40 + pTau217 + pTau181 + pTau181/A*β*42 | 0.919 | 160 | 0.93 | 100 |
| A*β*42/40 + pTau217 + pTau217/A*β*40 + pTau181/A*β*40 + pTau181/A*β*42 | 0.918 | 158 | 0.942 | 103 |
| pTau181 + pTau217/A*β*42 + pTau181/A*β*40 | 0.918 | 184 | 0.867 | 158 |
| pTau181 + NfL + pTau217/A*β*40 | 0.918 | 159 | 0.867 | 113 |
| pTau181 + pTau217/A*β*42 + pTau181/A*β*42 | 0.918 | 182 | 0.861 | 158 |
| pTau181 + pTau217/A*β*42 | 0.918 | 195 | 0.867 | 165 |
| pTau217 + pTau181 + pTau181/A*β*40 | 0.918 | 184 | 0.859 | 163 |
| pTau217/A*β*40 + pTau181/A*β*40 | 0.918 | 194 | 0.864 | 176 |
| A*β*42/40 + pTau217 + pTau181/A*β*40 | 0.918 | 158 | 0.933 | 104 |
| A*β*42/40 + pTau217/A*β*40 + pTau181/A*β*40 | 0.918 | 159 | 0.942 | 104 |
| pTau217 + pTau181 + pTau181/A*β*42 | 0.918 | 182 | 0.849 | 159 |
| pTau217/A*β*40 + pTau181/A*β*40 + pTau181/A*β*42 | 0.918 | 183 | 0.862 | 167 |
| A*β*42/40 + pTau217 + pTau181/A*β*40 + pTau181/A*β*42 | 0.918 | 158 | 0.933 | 104 |
| A*β*42/40 + pTau217 + pTau217/A*β*40 + pTau181/A*β*40 | 0.918 | 158 | 0.942 | 103 |
| A*β*42/40 + pTau217 + pTau217/A*β*42 + pTau181/A*β*40 | 0.918 | 158 | 0.942 | 103 |
| A*β*42/40 + pTau217/A*β*40 + pTau181/A*β*40 + pTau181/A*β*42 | 0.918 | 159 | 0.942 | 104 |
| A*β*42/40 + pTau217/A*β*40 + pTau217/A*β*42 + pTau181/A*β*40 | 0.918 | 159 | 0.942 | 104 |
|  |  |  |  | |

| Combination | A+/T+ in BBM+ | n BBM+ | non-A+/T+ in BBM- | n BBM- |
| --- | --- | --- | --- | --- |
| A*β*42/40 + pTau217 + pTau217/A*β*40 + pTau217/A*β*42 + pTau181/A*β*40 +  pTau181/A*β*42 | 0.918 | 158 | 0.942 | 103 |
| A*β*42/40 + pTau217/A*β*40 + pTau217/A*β*42 + pTau181/A*β*40 + pTau181/A*β*42 | 0.918 | 159 | 0.942 | 104 |
| pTau217 + pTau181 + NfL + pTau217/A*β*40 | 0.918 | 159 | 0.867 | 113 |
| pTau181 + NfL + pTau217/A*β*40 + pTau217/A*β*42 | 0.918 | 158 | 0.864 | 110 |
| A*β*42/40 + pTau217 + pTau217/A*β*42 + pTau181/A*β*40 + pTau181/A*β*42 | 0.918 | 158 | 0.942 | 103 |
| pTau217 + pTau181 + NfL + pTau217/A*β*42 | 0.918 | 158 | 0.857 | 112 |
| A*β*42/40 + pTau217 + pTau217/A*β*40 + pTau217/A*β*42 + pTau181/A*β*40 | 0.918 | 158 | 0.942 | 103 |
| pTau217 + pTau181 + NfL + pTau217/A*β*40 + pTau217/A*β*42 | 0.918 | 158 | 0.864 | 110 |
| pTau217 + pTau181/A*β*40 + pTau181/A*β*42 | 0.917 | 181 | 0.851 | 168 |
| pTau181 + NfL + pTau217/A*β*42 + pTau181/A*β*40 + pTau181/A*β*42 | 0.917 | 145 | 0.858 | 106 |
| pTau217 + NfL + pTau217/A*β*42 + pTau181/A*β*40 + pTau181/A*β*42 | 0.917 | 145 | 0.85 | 107 |
| pTau217 + pTau181 + NfL + pTau181/A*β*40 + pTau181/A*β*42 | 0.917 | 145 | 0.833 | 108 |
| NfL + pTau217/A*β*40 + pTau217/A*β*42 + pTau181/A*β*40 + pTau181/A*β*42 | 0.917 | 145 | 0.85 | 107 |
| pTau217 + NfL + pTau217/A*β*40 + pTau181/A*β*40 + pTau181/A*β*42 | 0.917 | 145 | 0.85 | 107 |
| pTau217 + NfL + pTau217/A*β*40 + pTau217/A*β*42 + pTau181/A*β*40 +  pTau181/A*β*42 | 0.917 | 145 | 0.85 | 107 |
| NfL + pTau217/A*β*40 + pTau181/A*β*40 + pTau181/A*β*42 | 0.917 | 145 | 0.85 | 107 |
| A*β*42/40 + pTau181 + pTau217/A*β*42 + pTau181/A*β*40 + pTau181/A*β*42 | 0.916 | 154 | 0.939 | 99 |
| A*β*42/40 + pTau181 + pTau217/A*β*42 + pTau181/A*β*40 | 0.916 | 154 | 0.939 | 99 |
| A*β*42/40 + pTau181 + NfL + pTau217/A*β*40 | 0.916 | 131 | 0.944 | 71 |
| pTau217 + pTau217/A*β*40 + pTau181/A*β*42 | 0.916 | 190 | 0.868 | 167 |
| A*β*42/40 + pTau217 + pTau181 + NfL | 0.916 | 131 | 0.919 | 74 |
| A*β*42/40 + pTau181 + pTau181/A*β*40 + pTau181/A*β*42 | 0.916 | 155 | 0.865 | 111 |
| A*β*42/40 + pTau181 + pTau181/A*β*40 | 0.916 | 155 | 0.865 | 111 |
| Combination | A+/T+ in BBM+ | n BBM+ | non-A+/T+ in BBM- | n BBM- |
| pTau217 + pTau181/A*β*40 | 0.916 | 191 | 0.854 | 178 |
| pTau217 + pTau217/A*β*42 + pTau181/A*β*42 | 0.916 | 190 | 0.868 | 167 |
| A*β*42/40 + pTau217 + pTau181 + NfL + pTau217/A*β*40 + pTau217/A*β*42 | 0.916 | 131 | 0.944 | 71 |
| A*β*42/40 + pTau181 + NfL + pTau217/A*β*40 + pTau217/A*β*42 | 0.916 | 131 | 0.944 | 71 |
| pTau217 + pTau217/A*β*40 + pTau217/A*β*42 + pTau181/A*β*42 | 0.916 | 190 | 0.873 | 166 |
| A*β*42/40 + pTau217 + pTau181 + NfL + pTau217/A*β*42 | 0.916 | 131 | 0.932 | 73 |
| A*β*42/40 + pTau217 + pTau181 + NfL + pTau217/A*β*40 | 0.916 | 131 | 0.944 | 71 |
| A*β*42/40 + pTau181 + NfL + pTau217/A*β*40 + pTau181/A*β*42 | 0.915 | 130 | 0.943 | 70 |
| A*β*42/40 + pTau217 + pTau181 + NfL + pTau181/A*β*42 | 0.915 | 130 | 0.917 | 72 |
| A*β*42/40 + pTau217 + pTau181 + NfL + pTau217/A*β*42 + pTau181/A*β*42 | 0.915 | 130 | 0.93 | 71 |
| A*β*42/40 + pTau217 + pTau181 + NfL + pTau217/A*β*40 + pTau181/A*β*42 | 0.915 | 130 | 0.943 | 70 |
| A*β*42/40 + pTau217 + pTau181 + NfL + pTau217/A*β*40 + pTau217/A*β*42 +  pTau181/A*β*42 | 0.915 | 130 | 0.943 | 70 |
| A*β*42/40 + pTau181 + NfL + pTau217/A*β*40 + pTau217/A*β*42 + pTau181/A*β*42 | 0.915 | 130 | 0.943 | 70 |
| pTau217 + NfL + pTau217/A*β*40 + pTau217/A*β*42 + pTau181/A*β*40 | 0.914 | 152 | 0.852 | 108 |
| pTau217 + NfL + pTau217/A*β*40 + pTau217/A*β*42 + pTau181/A*β*42 | 0.914 | 151 | 0.852 | 108 |
| NfL + pTau217/A*β*40 + pTau217/A*β*42 + pTau181/A*β*40 | 0.914 | 152 | 0.852 | 108 |
| NfL + pTau217/A*β*40 + pTau217/A*β*42 + pTau181/A*β*42 | 0.914 | 151 | 0.852 | 108 |
| pTau181 + NfL + pTau217/A*β*42 + pTau181/A*β*40 | 0.914 | 151 | 0.86 | 107 |
| pTau217 + NfL + pTau217/A*β*42 + pTau181/A*β*40 | 0.914 | 152 | 0.852 | 108 |
| pTau217 + NfL + pTau217/A*β*40 + pTau181/A*β*42 | 0.914 | 151 | 0.852 | 108 |
| pTau217 + NfL + pTau217/A*β*40 + pTau181/A*β*40 | 0.914 | 152 | 0.855 | 110 |

| Combination | A+/T+ in BBM+ | n BBM+ | non-A+/T+ in BBM- | n BBM- |
| --- | --- | --- | --- | --- |
| pTau217 + pTau181 + NfL + pTau181/A*β*40 | 0.914 | 151 | 0.839 | 112 |
| pTau217 + NfL + pTau217/A*β*42 + pTau181/A*β*42 | 0.914 | 151 | 0.844 | 109 |
| NfL + pTau217/A*β*40 + pTau181/A*β*40 | 0.914 | 152 | 0.855 | 110 |
| A*β*42/40 + pTau181 + pTau217/A*β*42 | 0.914 | 163 | 0.932 | 103 |
| NfL + pTau217/A*β*40 + pTau181/A*β*42 | 0.914 | 151 | 0.852 | 108 |
| pTau217 + pTau181 | 0.914 | 197 | 0.855 | 172 |
| pTau217/A*β*42 + pTau181/A*β*40 + pTau181/A*β*42 | 0.913 | 183 | 0.858 | 169 |
| A*β*42/40 + NfL + pTau217/A*β*40 + pTau217/A*β*42 + pTau181/A*β*40 +  pTau181/A*β*42 | 0.913 | 126 | 0.943 | 70 |
| A*β*42/40 + NfL + pTau217/A*β*40 + pTau181/A*β*40 + pTau181/A*β*42 | 0.913 | 126 | 0.943 | 70 |
| A*β*42/40 + NfL + pTau217/A*β*40 + pTau217/A*β*42 + pTau181/A*β*40 | 0.913 | 126 | 0.943 | 70 |
| A*β*42/40 + pTau217 + NfL + pTau217/A*β*42 + pTau181/A*β*40 + pTau181/A*β*42 | 0.913 | 126 | 0.943 | 70 |
| A*β*42/40 + pTau217 + NfL + pTau217/A*β*40 + pTau181/A*β*40 | 0.913 | 126 | 0.943 | 70 |
| A*β*42/40 + pTau181 + NfL + pTau217/A*β*42 + pTau181/A*β*40 | 0.913 | 126 | 0.943 | 70 |
| pTau217 + pTau181 + NfL + pTau181/A*β*42 | 0.913 | 150 | 0.827 | 110 |
| A*β*42/40 + NfL + pTau217/A*β*40 + pTau181/A*β*40 | 0.913 | 126 | 0.943 | 70 |
| A*β*42/40 + pTau181 + pTau217/A*β*42 + pTau181/A*β*42 | 0.913 | 161 | 0.931 | 101 |
| A*β*42/40 + pTau181 + NfL + pTau181/A*β*40 | 0.913 | 126 | 0.882 | 76 |
| A*β*42/40 + pTau217 + NfL + pTau181/A*β*40 | 0.913 | 126 | 0.93 | 71 |
| A*β*42/40 + pTau217 + NfL + pTau217/A*β*40 + pTau217/A*β*42 + pTau181/A*β*40 +  pTau181/A*β*42 | 0.913 | 126 | 0.943 | 70 |
| A*β*42/40 + pTau217 + NfL + pTau217/A*β*40 + pTau217/A*β*42 + pTau181/A*β*40 | 0.913 | 126 | 0.943 | 70 |
| A*β*42/40 + pTau217 + NfL + pTau217/A*β*40 + pTau181/A*β*40 + pTau181/A*β*42 | 0.913 | 126 | 0.943 | 70 |
| A*β*42/40 + pTau181 + NfL + pTau217/A*β*42 + pTau181/A*β*40 + pTau181/A*β*42 | 0.913 | 126 | 0.943 | 70 |
| pTau181 + NfL + pTau217/A*β*42 + pTau181/A*β*42 | 0.913 | 150 | 0.852 | 108 |
| A*β*42/40 + pTau217 + NfL + pTau181/A*β*40 + pTau181/A*β*42 | 0.913 | 126 | 0.93 | 71 |
|  |  |  |  | |

| Combination | A+/T+ in BBM+ | n BBM+ | non-A+/T+ in BBM- | n BBM- |
| --- | --- | --- | --- | --- |
| A*β*42/40 + pTau217 + NfL + pTau217/A*β*42 + pTau181/A*β*40 | 0.913 | 126 | 0.943 | 70 |
| A*β*42/40 + pTau181 + NfL + pTau181/A*β*40 + pTau181/A*β*42 | 0.913 | 126 | 0.882 | 76 |
| pTau181 + NfL + pTau217/A*β*42 | 0.912 | 159 | 0.858 | 113 |
| pTau217/A*β*42 + pTau181/A*β*40 | 0.912 | 193 | 0.857 | 175 |
| pTau217 + pTau181/A*β*42 | 0.911 | 191 | 0.848 | 171 |
| A*β*42/40 + pTau217 + pTau181/A*β*42 | 0.911 | 168 | 0.925 | 107 |
| pTau217 + NfL + pTau181/A*β*40 + pTau181/A*β*42 | 0.911 | 146 | 0.827 | 110 |
| A*β*42/40 + pTau217 + pTau217/A*β*42 + pTau181/A*β*42 | 0.911 | 168 | 0.934 | 106 |
| A*β*42/40 + pTau217 + pTau217/A*β*40 + pTau181/A*β*42 | 0.911 | 168 | 0.943 | 105 |
| pTau217/A*β*40 + pTau217/A*β*42 + pTau181/A*β*42 | 0.911 | 192 | 0.869 | 168 |
| A*β*42/40 + pTau217 + pTau217/A*β*40 + pTau217/A*β*42 + pTau181/A*β*42 | 0.911 | 168 | 0.943 | 105 |
| NfL + pTau217/A*β*42 + pTau181/A*β*40 + pTau181/A*β*42 | 0.911 | 146 | 0.852 | 108 |
| A*β*42/40 + pTau217 + NfL + pTau217/A*β*40 + pTau181/A*β*42 | 0.909 | 132 | 0.944 | 71 |
| A*β*42/40 + pTau181 + NfL + pTau217/A*β*42 | 0.909 | 132 | 0.932 | 74 |
| A*β*42/40 + pTau217 + NfL + pTau181/A*β*42 | 0.909 | 132 | 0.918 | 73 |
| A*β*42/40 + NfL + pTau217/A*β*40 + pTau217/A*β*42 + pTau181/A*β*42 | 0.909 | 132 | 0.944 | 71 |
| A*β*42/40 + NfL + pTau217/A*β*40 + pTau181/A*β*42 | 0.909 | 132 | 0.944 | 71 |
| A*β*42/40 + pTau217 + NfL + pTau217/A*β*42 + pTau181/A*β*42 | 0.909 | 132 | 0.931 | 72 |
| A*β*42/40 + pTau217 + NfL + pTau217/A*β*40 + pTau217/A*β*42 + pTau181/A*β*42 | 0.909 | 132 | 0.944 | 71 |
| pTau217 + NfL + pTau181/A*β*40 | 0.908 | 153 | 0.833 | 114 |
| NfL + pTau217/A*β*42 + pTau181/A*β*40 | 0.908 | 153 | 0.853 | 109 |
| A*β*42/40 + pTau181 + NfL + pTau217/A*β*42 + pTau181/A*β*42 | 0.908 | 131 | 0.931 | 72 |
| pTau217 + NfL + pTau181/A*β*42 | 0.908 | 152 | 0.821 | 112 |
| pTau217 + pTau181 + NfL | 0.907 | 161 | 0.833 | 120 |
| pTau217/A*β*40 + pTau181/A*β*42 | 0.907 | 194 | 0.864 | 169 |
| A*β*42/40 + pTau217/A*β*42 + pTau181/A*β*40 | 0.907 | 161 | 0.925 | 107 |
| A*β*42/40 + pTau217/A*β*42 + pTau181/A*β*40 + pTau181/A*β*42 | 0.907 | 161 | 0.925 | 107 |
|  |  |  |  | |

| Combination | A+/T+ in BBM+ | n BBM+ | non-A+/T+ in BBM- | n BBM- |
| --- | --- | --- | --- | --- |
| A*β*42/40 + NfL + pTau217/A*β*42 + pTau181/A*β*40 | 0.906 | 127 | 0.944 | 71 |
| A*β*42/40 + pTau217/A*β*40 + pTau181/A*β*42 | 0.906 | 170 | 0.943 | 106 |
| A*β*42/40 + pTau217/A*β*40 + pTau217/A*β*42 + pTau181/A*β*42 | 0.906 | 170 | 0.943 | 106 |
| A*β*42/40 + NfL + pTau217/A*β*42 + pTau181/A*β*40 + pTau181/A*β*42 | 0.906 | 127 | 0.944 | 71 |
| pTau181 + NfL + pTau181/A*β*40 + pTau181/A*β*42 | 0.905 | 147 | 0.768 | 125 |
| NfL + pTau217/A*β*42 + pTau181/A*β*42 | 0.902 | 153 | 0.845 | 110 |
| A*β*42/40 + NfL + pTau181/A*β*40 | 0.898 | 128 | 0.883 | 77 |
| A*β*42/40 + pTau181 + pTau181/A*β*42 | 0.898 | 166 | 0.851 | 114 |
| A*β*42/40 + NfL + pTau181/A*β*40 + pTau181/A*β*42 | 0.898 | 128 | 0.883 | 77 |
| A*β*42/40 + NfL + pTau217/A*β*42 + pTau181/A*β*42 | 0.896 | 134 | 0.932 | 73 |
| pTau181 + NfL + pTau181/A*β*40 | 0.896 | 154 | 0.771 | 131 |
| NfL + pTau181/A*β*40 + pTau181/A*β*42 | 0.893 | 149 | 0.758 | 128 |
| A*β*42/40 + pTau217 + NfL | 0.893 | 140 | 0.912 | 80 |
| A*β*42/40 + NfL + pTau217/A*β*40 | 0.892 | 139 | 0.947 | 76 |
| A*β*42/40 + NfL + pTau217/A*β*40 + pTau217/A*β*42 | 0.892 | 139 | 0.947 | 76 |
| A*β*42/40 + pTau217 + NfL + pTau217/A*β*40 | 0.892 | 139 | 0.947 | 76 |
| A*β*42/40 + pTau217 + NfL + pTau217/A*β*42 | 0.892 | 139 | 0.924 | 79 |
| A*β*42/40 + pTau217 + NfL + pTau217/A*β*40 + pTau217/A*β*42 | 0.892 | 139 | 0.947 | 76 |
| pTau217 + NfL + pTau217/A*β*40 | 0.891 | 175 | 0.858 | 120 |
| NfL + pTau217/A*β*40 | 0.891 | 175 | 0.858 | 120 |
| A*β*42/40 + pTau181/A*β*40 + pTau181/A*β*42 | 0.891 | 165 | 0.843 | 121 |
| A*β*42/40 + pTau181/A*β*40 | 0.891 | 165 | 0.843 | 121 |
| pTau181 + pTau181/A*β*40 + pTau181/A*β*42 | 0.891 | 183 | 0.771 | 188 |
| NfL + pTau217/A*β*40 + pTau217/A*β*42 | 0.89 | 173 | 0.855 | 117 |
| pTau217 + NfL + pTau217/A*β*42 | 0.89 | 173 | 0.842 | 120 |
| pTau217 + NfL + pTau217/A*β*40 + pTau217/A*β*42 | 0.89 | 173 | 0.855 | 117 |
| A*β*42/40 + pTau181 + NfL + pTau181/A*β*42 | 0.889 | 135 | 0.872 | 78 |
| pTau217/A*β*42 + pTau181/A*β*42 | 0.888 | 197 | 0.855 | 172 |
| pTau181 + pTau181/A*β*40 | 0.886 | 193 | 0.772 | 197 |
| pTau181 + NfL + pTau181/A*β*42 | 0.885 | 156 | 0.764 | 127 |
| NfL + pTau181/A*β*40 | 0.885 | 157 | 0.75 | 136 |
| pTau217 + pTau217/A*β*42 | 0.884 | 232 | 0.865 | 185 |

| Combination | A+/T+ in BBM+ | n BBM+ | non-A+/T+ in BBM- | n BBM- |
| --- | --- | --- | --- | --- |
| pTau217 + pTau217/A*β*40 + pTau217/A*β*42 | 0.884 | 232 | 0.874 | 182 |
| A*β*42/40 + pTau217 | 0.883 | 188 | 0.922 | 115 |
| A*β*42/40 + pTau217 + pTau217/A*β*40 + pTau217/A*β*42 | 0.882 | 187 | 0.946 | 111 |
| A*β*42/40 + pTau217 + pTau217/A*β*40 | 0.882 | 187 | 0.946 | 111 |
| A*β*42/40 + pTau217 + pTau217/A*β*42 | 0.882 | 187 | 0.93 | 114 |
| pTau217 + NfL | 0.882 | 178 | 0.82 | 128 |
| pTau217/A*β*40 + pTau217/A*β*42 | 0.882 | 237 | 0.87 | 185 |
| NfL + pTau217/A*β*42 | 0.881 | 176 | 0.843 | 121 |
| pTau217 + pTau217/A*β*40 | 0.881 | 235 | 0.872 | 188 |
| A*β*42/40 + NfL + pTau217/A*β*42 | 0.88 | 142 | 0.925 | 80 |
| A*β*42/40 + pTau217/A*β*40 + pTau217/A*β*42 | 0.88 | 191 | 0.947 | 113 |
| A*β*42/40 + pTau217/A*β*42 + pTau181/A*β*42 | 0.88 | 175 | 0.918 | 110 |
| A*β*42/40 + pTau217/A*β*40 | 0.88 | 191 | 0.947 | 113 |
| A*β*42/40 + pTau181 | 0.879 | 173 | 0.846 | 117 |
| pTau217/A*β*40 | 0.877 | 243 | 0.869 | 191 |
| pTau181 + pTau181/A*β*42 | 0.876 | 194 | 0.764 | 191 |
| pTau217 | 0.874 | 238 | 0.847 | 196 |
| pTau217/A*β*42 | 0.864 | 243 | 0.853 | 191 |
| A*β*42/40 + pTau181 + NfL | 0.864 | 140 | 0.864 | 81 |
| A*β*42/40 + NfL + pTau181/A*β*42 | 0.864 | 140 | 0.841 | 82 |
| NfL + pTau181/A*β*42 | 0.863 | 161 | 0.737 | 133 |
| pTau181/A*β*40 + pTau181/A*β*42 | 0.862 | 196 | 0.756 | 205 |
| A*β*42/40 + pTau217/A*β*42 | 0.858 | 197 | 0.916 | 119 |
| pTau181/A*β*40 | 0.856 | 209 | 0.738 | 225 |
| A*β*42/40 + pTau181/A*β*42 | 0.843 | 185 | 0.784 | 134 |
| pTau181 | 0.837 | 221 | 0.751 | 213 |
| pTau181 + NfL | 0.836 | 177 | 0.743 | 144 |
| pTau181/A*β*42 | 0.824 | 216 | 0.725 | 218 |
| A*β*42/40 + NfL | 0.751 | 173 | 0.815 | 92 |
| A*β*42/40 | 0.695 | 269 | 0.691 | 165 |
| NfL | 0.667 | 246 | 0.606 | 188 |

Supplementary table 9 Cutoff combinations — K-Means (all 255 combinations, eight biomarkers). Prevalence of A^+^/T^+^ CSF profile in BBM-positive subgroups and prevalence of non-A^+^/T^+^ CSF profile in BBM-negative subgroups.

Combination	A+/T+ in BBM+	n BBM+	non-A+/T+ in BBM-	n BBM-

pTau217 + pTau181 + NfL + pTau217/A*β*42 +	0.948	115	0.78	141	pTau181/A*β*40

| Combination | | A+/T+ in BBM+ | | n BBM+ | | non-A+/T+ in BBM- | | n BBM- | |  |
| --- | --- | --- | --- | --- | --- | --- | --- | --- | --- | --- |
| pTau181 + NfL + pTau217/A*β*40 + pTau217/A*β*42 + pTau181/A*β*40 | | 0.948 | | 115 | | 0.784 | | 139 | |  |
| pTau217 + pTau181 + NfL + pTau217/A*β*40 + pTau217/A*β*42 + pTau181/A*β*40 | | 0.948 | | 115 | | 0.784 | | 139 | |  |
| pTau217 + pTau181 + NfL + pTau217/A*β*40 + pTau181/A*β*40 | | 0.948 | | 116 | | 0.79 | | 143 | |  |
| pTau181 + NfL + pTau217/A*β*40 + pTau181/A*β*40 | | 0.948 | | 116 | | 0.79 | | 143 | |  |
| pTau181 + NfL + pTau217/A*β*42 + pTau181/A*β*40 | | 0.948 | | 115 | | 0.775 | | 142 | |  |
| pTau217 + pTau181 + NfL + pTau217/A*β*40 + pTau217/A*β*42 + pTau181/A*β*40 +  pTau181/A*β*42 | | 0.946 | | 111 | | 0.781 | | 137 | |  |
| pTau181 + NfL + pTau217/A*β*40 + pTau181/A*β*42 | | 0.946 | | 112 | | 0.775 | | 142 | |  |
| pTau181 + NfL + pTau217/A*β*42 + pTau181/A*β*42 | | 0.946 | | 112 | | 0.762 | | 143 | |  |
| pTau181 + NfL + pTau217/A*β*40 + pTau217/A*β*42 + pTau181/A*β*40 +  pTau181/A*β*42 | | 0.946 | | 111 | | 0.781 | | 137 | |  |
| pTau217 + pTau181 + NfL + pTau217/A*β*40 + pTau217/A*β*42 + pTau181/A*β*42 | | 0.946 | | 112 | | 0.771 | | 140 | |  |
| pTau181 + NfL + pTau217/A*β*42 + pTau181/A*β*40 + pTau181/A*β*42 | | 0.946 | | 111 | | 0.771 | | 140 | |  |
| pTau181 + NfL + pTau217/A*β*40 + pTau181/A*β*40 + pTau181/A*β*42 | | 0.946 | | 111 | | 0.784 | | 139 | |  |
| pTau181 + NfL + pTau217/A*β*40 + pTau217/A*β*42 + pTau181/A*β*42 | | 0.946 | | 112 | | 0.771 | | 140 | |  |
| pTau217 + pTau181 + NfL + pTau217/A*β*40 + pTau181/A*β*42 | | 0.946 | | 112 | | 0.775 | | 142 | |  |
| pTau217 + pTau181 + NfL + pTau217/A*β*40 + pTau181/A*β*40 + pTau181/A*β*42 | | 0.946 | | 111 | | 0.784 | | 139 | |  |
| pTau217 + pTau181 + NfL + pTau217/A*β*42 + pTau181/A*β*42 | | 0.946 | | 112 | | 0.768 | | 142 | |  |
| pTau217 + pTau181 + NfL + pTau217/A*β*42 + pTau181/A*β*40 + pTau181/A*β*42 | | 0.946 | | 111 | | 0.777 | | 139 | |  |
| pTau181 + NfL + pTau217/A*β*42 | | 0.941 | | 118 | | 0.758 | | 149 | |  |
| pTau217 + pTau181 + NfL + pTau217/A*β*40 + pTau217/A*β*42 | | 0.941 | | 118 | | 0.767 | | 146 | |  |
| pTau217 + pTau181 + NfL + pTau217/A*β*40 | | 0.941 | | 119 | | 0.77 | | 152 | |  |
| pTau181 + NfL + pTau217/A*β*40 | | 0.941 | | 119 | | 0.77 | | 152 | |  |
|  | Combination | | A+/T+ in BBM+ | | n BBM+ | | non-A+/T+ in BBM- | | n BBM- | |
|  | pTau217 + pTau181 + NfL + pTau181/A*β*40 | | 0.941 | | 119 | | 0.776 | | 147 | |
|  | pTau217 + pTau181 + NfL + pTau217/A*β*42 | | 0.941 | | 118 | | 0.764 | | 148 | |
|  | pTau181 + NfL + pTau217/A*β*40 + pTau217/A*β*42 | | 0.941 | | 118 | | 0.767 | | 146 | |
|  | A*β*42/40 + pTau217 + pTau181 + NfL + pTau217/A*β*40 + pTau217/A*β*42 | | 0.939 | | 98 | | 0.895 | | 86 | |
|  | A*β*42/40 + pTau181 + NfL + pTau217/A*β*42 | | 0.939 | | 98 | | 0.876 | | 89 | |
|  | pTau181 + pTau217/A*β*42 + pTau181/A*β*40 | | 0.939 | | 147 | | 0.765 | | 204 | |
|  | pTau181 + pTau217/A*β*40 + pTau181/A*β*40 | | 0.939 | | 147 | | 0.776 | | 205 | |
|  | A*β*42/40 + pTau181 + NfL + pTau217/A*β*40 | | 0.939 | | 98 | | 0.895 | | 86 | |
|  | A*β*42/40 + pTau217 + pTau181 + NfL | | 0.939 | | 99 | | 0.876 | | 89 | |
|  | A*β*42/40 + pTau217 + pTau181 + NfL + pTau217/A*β*40 | | 0.939 | | 98 | | 0.895 | | 86 | |
|  | A*β*42/40 + pTau217 + pTau181 + NfL + pTau217/A*β*42 | | 0.939 | | 98 | | 0.886 | | 88 | |
|  | A*β*42/40 + pTau217 + pTau181 + NfL + pTau181/A*β*42 | | 0.939 | | 98 | | 0.884 | | 86 | |
|  | A*β*42/40 + pTau181 + NfL + pTau217/A*β*40 + pTau217/A*β*42 | | 0.939 | | 98 | | 0.895 | | 86 | |
|  | pTau217 + pTau181 + NfL + pTau181/A*β*42 | | 0.939 | | 114 | | 0.766 | | 145 | |
|  | pTau181 + pTau217/A*β*40 + pTau217/A*β*42 + pTau181/A*β*42 | | 0.938 | | 144 | | 0.761 | | 201 | |
|  | pTau217 + pTau181 + pTau217/A*β*40 + pTau181/A*β*40 | | 0.938 | | 146 | | 0.776 | | 205 | |
|  | pTau181 + pTau217/A*β*40 + pTau217/A*β*42 + pTau181/A*β*40 | | 0.938 | | 146 | | 0.771 | | 201 | |
|  | pTau181 + pTau217/A*β*42 + pTau181/A*β*42 | | 0.938 | | 145 | | 0.755 | | 204 | |
|  | pTau181 + pTau217/A*β*40 + pTau181/A*β*42 | | 0.938 | | 144 | | 0.764 | | 203 | |
|  | pTau217 + pTau181 + pTau217/A*β*42 + pTau181/A*β*40 | | 0.938 | | 146 | | 0.768 | | 203 | |
|  | pTau217 + pTau181 + pTau217/A*β*42 + pTau181/A*β*42 | | 0.938 | | 144 | | 0.759 | | 203 | |
|  | A*β*42/40 + pTau181 + NfL + pTau217/A*β*40 + pTau181/A*β*40 | | 0.938 | | 96 | | 0.902 | | 82 | |
|  | A*β*42/40 + pTau181 + NfL + pTau217/A*β*40 + pTau181/A*β*42 | | 0.938 | | 97 | | 0.904 | | 83 | |
|  | A*β*42/40 + pTau181 + NfL + pTau217/A*β*42 + pTau181/A*β*40 | | 0.938 | | 96 | | 0.882 | | 85 | |
|  | A*β*42/40 + pTau217 + pTau181 + NfL + pTau181/A*β*40 | | 0.938 | | 97 | | 0.882 | | 85 | |

| Combination | A+/T+ in BBM+ | n BBM+ | non-A+/T+ in BBM- | n BBM- |
| --- | --- | --- | --- | --- |
| A*β*42/40 + pTau217 + pTau181 + NfL + pTau217/A*β*40 + pTau217/A*β*42 + pTau181/A*β*40 + pTau181/A*β*42 | 0.938 | 96 | 0.902 | 82 |
| A*β*42/40 + pTau181 + NfL + pTau217/A*β*40 + pTau217/A*β*42 + pTau181/A*β*40 +  pTau181/A*β*42 | 0.938 | 96 | 0.902 | 82 |
| A*β*42/40 + pTau217 + pTau181 + NfL + pTau217/A*β*42 + pTau181/A*β*40 +  pTau181/A*β*42 | 0.938 | 96 | 0.893 | 84 |
| A*β*42/40 + pTau181 + NfL + pTau217/A*β*40 + pTau181/A*β*40 + pTau181/A*β*42 | 0.938 | 96 | 0.902 | 82 |
| A*β*42/40 + pTau181 + NfL + pTau217/A*β*40 + pTau217/A*β*42 + pTau181/A*β*42 | 0.938 | 97 | 0.904 | 83 |
| A*β*42/40 + pTau181 + NfL + pTau217/A*β*40 + pTau217/A*β*42 + pTau181/A*β*40 | 0.938 | 96 | 0.902 | 82 |
| A*β*42/40 + pTau217 + pTau181 + NfL + pTau217/A*β*40 + pTau217/A*β*42 +  pTau181/A*β*42 | 0.938 | 97 | 0.904 | 83 |
| A*β*42/40 + pTau217 + pTau181 + NfL + pTau217/A*β*40 + pTau217/A*β*42 +  pTau181/A*β*40 | 0.938 | 96 | 0.902 | 82 |
| A*β*42/40 + pTau181 + NfL + pTau217/A*β*42 + pTau181/A*β*40 + pTau181/A*β*42 | 0.938 | 96 | 0.882 | 85 |
| A*β*42/40 + pTau217 + pTau181 + NfL + pTau181/A*β*40 + pTau181/A*β*42 | 0.938 | 97 | 0.882 | 85 |
| A*β*42/40 + pTau217 + pTau181 + NfL + pTau217/A*β*40 + pTau181/A*β*42 | 0.938 | 97 | 0.904 | 83 |
| A*β*42/40 + pTau217 + pTau181 + NfL + pTau217/A*β*40 + pTau181/A*β*40 | 0.938 | 96 | 0.902 | 82 |
| A*β*42/40 + pTau217 + pTau181 + NfL + pTau217/A*β*42 + pTau181/A*β*40 | 0.938 | 96 | 0.893 | 84 |
| pTau217 + pTau181 + NfL + pTau181/A*β*40 + pTau181/A*β*42 | 0.938 | 113 | 0.775 | 142 |
| pTau217 + pTau181 + pTau217/A*β*40 + pTau217/A*β*42 + pTau181/A*β*40 | 0.938 | 145 | 0.771 | 201 |
| A*β*42/40 + pTau217 + pTau181 + NfL + pTau217/A*β*42 + pTau181/A*β*42 | 0.938 | 97 | 0.894 | 85 |
| A*β*42/40 + pTau217 + pTau181 + NfL + pTau217/A*β*40 + pTau181/A*β*40 +  pTau181/A*β*42 | 0.938 | 96 | 0.902 | 82 |
| A*β*42/40 + pTau181 + NfL + pTau217/A*β*42 + pTau181/A*β*42 | 0.938 | 97 | 0.884 | 86 |
|  |  |  |  | |

| Combination | A+/T+ in BBM+ | n BBM+ | non-A+/T+ in BBM- | n BBM- |
| --- | --- | --- | --- | --- |
| pTau181 + pTau217/A*β*40 + pTau217/A*β*42 + pTau181/A*β*40 + pTau181/A*β*42 | 0.937 | 142 | 0.768 | 198 |
| pTau217 + pTau181 + pTau217/A*β*40 + pTau217/A*β*42 + pTau181/A*β*42 | 0.937 | 143 | 0.761 | 201 |
| pTau181 + pTau217/A*β*40 + pTau181/A*β*40 + pTau181/A*β*42 | 0.937 | 142 | 0.77 | 200 |
| pTau217 + pTau181 + pTau217/A*β*42 + pTau181/A*β*40 + pTau181/A*β*42 | 0.937 | 142 | 0.765 | 200 |
| pTau217 + pTau181 + pTau217/A*β*40 + pTau181/A*β*42 | 0.937 | 143 | 0.764 | 203 |
| pTau181 + pTau217/A*β*42 + pTau181/A*β*40 + pTau181/A*β*42 | 0.937 | 143 | 0.761 | 201 |
| pTau217 + pTau181 + pTau217/A*β*40 + pTau181/A*β*40 + pTau181/A*β*42 | 0.936 | 141 | 0.77 | 200 |
| pTau217 + pTau181 + pTau217/A*β*40 + pTau217/A*β*42 + pTau181/A*β*40 +  pTau181/A*β*42 | 0.936 | 141 | 0.768 | 198 |
| pTau181 + pTau217/A*β*42 | 0.935 | 153 | 0.755 | 212 |
| pTau181 + pTau217/A*β*40 | 0.935 | 153 | 0.763 | 215 |
| pTau217 + pTau181 + NfL | 0.935 | 123 | 0.752 | 157 |
| pTau217 + pTau181 + pTau217/A*β*40 | 0.934 | 152 | 0.763 | 215 |
| NfL + pTau217/A*β*40 + pTau181/A*β*40 | 0.934 | 122 | 0.769 | 147 |
| pTau217 + pTau181 + pTau217/A*β*42 | 0.934 | 152 | 0.758 | 211 |
| pTau181 + pTau217/A*β*40 + pTau217/A*β*42 | 0.934 | 152 | 0.761 | 209 |
| NfL + pTau217/A*β*42 + pTau181/A*β*40 | 0.934 | 121 | 0.753 | 146 |
| pTau217 + NfL + pTau217/A*β*40 + pTau181/A*β*40 | 0.934 | 122 | 0.769 | 147 |
| pTau217 + NfL + pTau217/A*β*40 + pTau217/A*β*42 + pTau181/A*β*40 | 0.934 | 121 | 0.762 | 143 |
| pTau217 + NfL + pTau217/A*β*42 + pTau181/A*β*40 | 0.934 | 121 | 0.759 | 145 |
| pTau217 + pTau181 + pTau217/A*β*40 + pTau217/A*β*42 | 0.934 | 151 | 0.761 | 209 |
| NfL + pTau217/A*β*40 + pTau217/A*β*42 + pTau181/A*β*40 | 0.934 | 121 | 0.762 | 143 |
| NfL + pTau217/A*β*42 + pTau181/A*β*42 | 0.933 | 120 | 0.748 | 147 |
| pTau217 + NfL + pTau217/A*β*40 + pTau217/A*β*42 + pTau181/A*β*42 | 0.933 | 120 | 0.757 | 144 |
| pTau217 + NfL + pTau217/A*β*40 + pTau181/A*β*42 | 0.933 | 120 | 0.76 | 146 |
| NfL + pTau217/A*β*40 + pTau217/A*β*42 + pTau181/A*β*42 | 0.933 | 120 | 0.757 | 144 |
|  |  |  |  | |

| Combination | A+/T+ in BBM+ | n BBM+ | non-A+/T+ in BBM- | n BBM- |
| --- | --- | --- | --- | --- |
| NfL + pTau217/A*β*40 + pTau181/A*β*42 | 0.933 | 120 | 0.76 | 146 |
| pTau217 + pTau181 + pTau181/A*β*40 | 0.933 | 150 | 0.766 | 209 |
| pTau217 + NfL + pTau217/A*β*42 + pTau181/A*β*42 | 0.933 | 120 | 0.753 | 146 |
| pTau217 + NfL + pTau217/A*β*42 + pTau181/A*β*40 + pTau181/A*β*42 | 0.932 | 117 | 0.766 | 141 |
| pTau217 + pTau181 + pTau181/A*β*42 | 0.932 | 146 | 0.757 | 206 |
| pTau217 + NfL + pTau217/A*β*40 + pTau181/A*β*40 + pTau181/A*β*42 | 0.932 | 117 | 0.773 | 141 |
| NfL + pTau217/A*β*40 + pTau217/A*β*42 + pTau181/A*β*40 + pTau181/A*β*42 | 0.932 | 117 | 0.77 | 139 |
| NfL + pTau217/A*β*42 + pTau181/A*β*40 + pTau181/A*β*42 | 0.932 | 117 | 0.761 | 142 |
| NfL + pTau217/A*β*40 + pTau181/A*β*40 + pTau181/A*β*42 | 0.932 | 117 | 0.773 | 141 |
| pTau217 + NfL + pTau217/A*β*40 + pTau217/A*β*42 + pTau181/A*β*40 +  pTau181/A*β*42 | 0.932 | 117 | 0.77 | 139 |
| pTau217 + pTau181 + pTau181/A*β*40 + pTau181/A*β*42 | 0.931 | 144 | 0.764 | 203 |
| A*β*42/40 + pTau217 + pTau181 | 0.931 | 130 | 0.843 | 121 |
| A*β*42/40 + pTau181 + pTau217/A*β*42 | 0.931 | 130 | 0.843 | 121 |
| A*β*42/40 + pTau217 + pTau181 + pTau217/A*β*42 | 0.93 | 129 | 0.85 | 120 |
| A*β*42/40 + pTau217 + pTau181 + pTau217/A*β*40 | 0.93 | 128 | 0.856 | 118 |
| A*β*42/40 + pTau181 + pTau217/A*β*40 | 0.93 | 129 | 0.856 | 118 |
| pTau217 + pTau181 | 0.93 | 157 | 0.75 | 220 |
| A*β*42/40 + pTau181 + pTau217/A*β*40 + pTau181/A*β*42 | 0.93 | 128 | 0.861 | 115 |
| A*β*42/40 + pTau217 + pTau181 + pTau217/A*β*40 + pTau217/A*β*42 | 0.93 | 128 | 0.856 | 118 |
| A*β*42/40 + pTau181 + pTau217/A*β*40 + pTau217/A*β*42 + pTau181/A*β*42 | 0.93 | 128 | 0.861 | 115 |
| A*β*42/40 + pTau217 + pTau181 + pTau217/A*β*42 + pTau181/A*β*42 | 0.93 | 128 | 0.855 | 117 |
| A*β*42/40 + pTau217 + pTau181 + pTau181/A*β*42 | 0.93 | 129 | 0.847 | 118 |
| A*β*42/40 + pTau181 + pTau217/A*β*42 + pTau181/A*β*42 | 0.93 | 129 | 0.847 | 118 |
| A*β*42/40 + pTau181 + pTau217/A*β*40 + pTau217/A*β*42 | 0.93 | 129 | 0.856 | 118 |
|  |  |  |  | |

| Combination | A+/T+ in BBM+ | n BBM+ | non-A+/T+ in BBM- | n BBM- |
| --- | --- | --- | --- | --- |
| A*β*42/40 + pTau181 + pTau217/A*β*42 + pTau181/A*β*40 | 0.929 | 127 | 0.846 | 117 |
| pTau217 + pTau217/A*β*42 + pTau181/A*β*40 | 0.929 | 154 | 0.748 | 218 |
| pTau217 + pTau217/A*β*40 + pTau181/A*β*40 | 0.929 | 154 | 0.755 | 220 |
| pTau217/A*β*40 + pTau217/A*β*42 + pTau181/A*β*40 | 0.929 | 155 | 0.747 | 217 |
| pTau217 + pTau217/A*β*42 + pTau181/A*β*42 | 0.929 | 154 | 0.742 | 217 |
| pTau217/A*β*40 + pTau181/A*β*40 | 0.929 | 156 | 0.751 | 221 |
| pTau217/A*β*40 + pTau181/A*β*42 | 0.929 | 156 | 0.74 | 219 |
| A*β*42/40 + pTau181 + pTau217/A*β*40 + pTau181/A*β*40 + pTau181/A*β*42 | 0.929 | 126 | 0.86 | 114 |
| A*β*42/40 + pTau217 + pTau181 + pTau217/A*β*40 + pTau181/A*β*42 | 0.929 | 127 | 0.861 | 115 |
| A*β*42/40 + pTau217 + pTau181 + pTau217/A*β*42 + pTau181/A*β*40 | 0.929 | 126 | 0.853 | 116 |
| pTau217/A*β*40 + pTau217/A*β*42 + pTau181/A*β*42 | 0.929 | 156 | 0.737 | 217 |
| A*β*42/40 + pTau181 + pTau217/A*β*40 + pTau181/A*β*40 | 0.929 | 126 | 0.86 | 114 |
| A*β*42/40 + pTau217 + pTau181 + pTau181/A*β*40 | 0.929 | 127 | 0.846 | 117 |
| A*β*42/40 + pTau181 + pTau217/A*β*40 + pTau217/A*β*42 + pTau181/A*β*40 +  pTau181/A*β*42 | 0.929 | 126 | 0.86 | 114 |
| A*β*42/40 + pTau217 + pTau181 + pTau217/A*β*42 + pTau181/A*β*40 +  pTau181/A*β*42 | 0.929 | 126 | 0.853 | 116 |
| A*β*42/40 + pTau181 + pTau217/A*β*42 + pTau181/A*β*40 + pTau181/A*β*42 | 0.929 | 127 | 0.846 | 117 |
| A*β*42/40 + pTau217 + pTau181 + pTau217/A*β*40 + pTau217/A*β*42 +  pTau181/A*β*42 | 0.929 | 127 | 0.861 | 115 |
| A*β*42/40 + pTau217 + pTau181 + pTau181/A*β*40 + pTau181/A*β*42 | 0.929 | 127 | 0.846 | 117 |
| A*β*42/40 + pTau181 + pTau217/A*β*40 + pTau217/A*β*42 + pTau181/A*β*40 | 0.929 | 126 | 0.86 | 114 |
| pTau217 + pTau217/A*β*40 + pTau181/A*β*42 | 0.928 | 153 | 0.747 | 217 |
| pTau217 + NfL + pTau181/A*β*40 | 0.928 | 125 | 0.755 | 151 |
| A*β*42/40 + pTau217 + pTau181 + pTau217/A*β*40 + pTau181/A*β*40 +  pTau181/A*β*42 | 0.928 | 125 | 0.86 | 114 |

| Combination | A+/T+ in BBM+ | n BBM+ | non-A+/T+ in BBM- | n BBM- |
| --- | --- | --- | --- | --- |
| A*β*42/40 + pTau217 + pTau181 + pTau217/A*β*40 + pTau217/A*β*42 +  pTau181/A*β*40 | 0.928 | 125 | 0.86 | 114 |
| pTau217 + pTau217/A*β*40 + pTau217/A*β*42 + pTau181/A*β*40 | 0.928 | 153 | 0.75 | 216 |
| pTau217 + pTau217/A*β*40 + pTau217/A*β*42 + pTau181/A*β*42 | 0.928 | 153 | 0.744 | 215 |
| A*β*42/40 + pTau217 + pTau181 + pTau217/A*β*40 + pTau217/A*β*42 + pTau181/A*β*40 + pTau181/A*β*42 | 0.928 | 125 | 0.86 | 114 |
| A*β*42/40 + pTau217 + pTau181 + pTau217/A*β*40 + pTau181/A*β*40 | 0.928 | 125 | 0.86 | 114 |
| pTau217 + pTau217/A*β*42 + pTau181/A*β*40 + pTau181/A*β*42 | 0.927 | 150 | 0.75 | 212 |
| A*β*42/40 + pTau217 + NfL + pTau217/A*β*40 + pTau217/A*β*42 | 0.927 | 109 | 0.88 | 92 |
| A*β*42/40 + NfL + pTau217/A*β*40 | 0.927 | 109 | 0.88 | 92 |
| A*β*42/40 + NfL + pTau217/A*β*42 | 0.927 | 109 | 0.863 | 95 |
| A*β*42/40 + pTau217 + NfL + pTau217/A*β*40 | 0.927 | 109 | 0.88 | 92 |
| pTau217/A*β*40 + pTau181/A*β*40 + pTau181/A*β*42 | 0.927 | 151 | 0.751 | 213 |
| pTau217/A*β*40 + pTau217/A*β*42 + pTau181/A*β*40 + pTau181/A*β*42 | 0.927 | 151 | 0.749 | 211 |
| A*β*42/40 + pTau217 + NfL + pTau217/A*β*42 | 0.927 | 109 | 0.872 | 94 |
| A*β*42/40 + NfL + pTau217/A*β*40 + pTau217/A*β*42 | 0.927 | 109 | 0.88 | 92 |
| A*β*42/40 + pTau217 + NfL | 0.927 | 110 | 0.863 | 95 |
| pTau217 + NfL + pTau181/A*β*42 | 0.926 | 122 | 0.752 | 149 |
| pTau217 + pTau217/A*β*40 + pTau181/A*β*40 + pTau181/A*β*42 | 0.926 | 149 | 0.755 | 212 |
| pTau217 + pTau217/A*β*40 + pTau217/A*β*42 + pTau181/A*β*40 + pTau181/A*β*42 | 0.926 | 149 | 0.752 | 210 |
| A*β*42/40 + pTau217 + NfL + pTau181/A*β*42 | 0.925 | 106 | 0.875 | 88 |
| pTau217/A*β*42 + pTau181/A*β*40 | 0.924 | 158 | 0.741 | 220 |
| pTau217 + NfL + pTau217/A*β*40 | 0.924 | 132 | 0.738 | 164 |
| pTau217 + pTau181/A*β*40 | 0.924 | 158 | 0.746 | 224 |
| NfL + pTau217/A*β*40 + pTau217/A*β*42 | 0.924 | 131 | 0.734 | 158 |
| pTau217 + NfL + pTau217/A*β*40 + pTau217/A*β*42 | 0.924 | 131 | 0.734 | 158 |
| A*β*42/40 + pTau217 + NfL + pTau217/A*β*40 + pTau181/A*β*42 | 0.924 | 105 | 0.894 | 85 |

| Combination | A+/T+ in BBM+ | n BBM+ | non-A+/T+ in BBM- | n BBM- |
| --- | --- | --- | --- | --- |
| NfL + pTau217/A*β*40 | 0.924 | 132 | 0.733 | 165 |
| NfL + pTau217/A*β*42 | 0.924 | 131 | 0.727 | 161 |
| pTau217 + NfL + pTau217/A*β*42 | 0.924 | 131 | 0.731 | 160 |
| A*β*42/40 + NfL + pTau217/A*β*42 + pTau181/A*β*42 | 0.924 | 105 | 0.875 | 88 |
| A*β*42/40 + NfL + pTau217/A*β*40 + pTau181/A*β*42 | 0.924 | 105 | 0.894 | 85 |
| A*β*42/40 + NfL + pTau217/A*β*40 + pTau217/A*β*42 + pTau181/A*β*42 | 0.924 | 105 | 0.894 | 85 |
| A*β*42/40 + pTau217 + NfL + pTau217/A*β*40 + pTau217/A*β*42 + pTau181/A*β*42 | 0.924 | 105 | 0.894 | 85 |
| A*β*42/40 + pTau217 + NfL + pTau217/A*β*42 + pTau181/A*β*42 | 0.924 | 105 | 0.885 | 87 |
| pTau217 + NfL + pTau181/A*β*40 + pTau181/A*β*42 | 0.924 | 119 | 0.764 | 144 |
| pTau217 + pTau181/A*β*42 | 0.923 | 156 | 0.741 | 220 |
| A*β*42/40 + pTau181 + NfL + pTau181/A*β*40 | 0.923 | 104 | 0.862 | 87 |
| A*β*42/40 + pTau181 + NfL + pTau181/A*β*40 + pTau181/A*β*42 | 0.923 | 104 | 0.862 | 87 |
| A*β*42/40 + pTau217 + NfL + pTau217/A*β*40 + pTau181/A*β*40 | 0.922 | 102 | 0.892 | 83 |
| A*β*42/40 + pTau217 + NfL + pTau217/A*β*42 + pTau181/A*β*40 | 0.922 | 102 | 0.882 | 85 |
| A*β*42/40 + pTau217 + NfL + pTau217/A*β*42 + pTau181/A*β*40 + pTau181/A*β*42 | 0.922 | 102 | 0.882 | 85 |
| A*β*42/40 + NfL + pTau217/A*β*40 + pTau217/A*β*42 + pTau181/A*β*40 | 0.922 | 102 | 0.892 | 83 |
| A*β*42/40 + pTau217 + NfL + pTau217/A*β*40 + pTau217/A*β*42 + pTau181/A*β*40 | 0.922 | 102 | 0.892 | 83 |
| A*β*42/40 + pTau217 + NfL + pTau217/A*β*40 + pTau181/A*β*40 + pTau181/A*β*42 | 0.922 | 102 | 0.892 | 83 |
| A*β*42/40 + pTau217 + NfL + pTau217/A*β*40 + pTau217/A*β*42 + pTau181/A*β*40 +  pTau181/A*β*42 | 0.922 | 102 | 0.892 | 83 |
| A*β*42/40 + pTau217 + NfL + pTau181/A*β*40 + pTau181/A*β*42 | 0.922 | 103 | 0.872 | 86 |
| A*β*42/40 + NfL + pTau217/A*β*40 + pTau181/A*β*40 + pTau181/A*β*42 | 0.922 | 102 | 0.892 | 83 |
| A*β*42/40 + NfL + pTau217/A*β*42 + pTau181/A*β*40 + pTau181/A*β*42 | 0.922 | 102 | 0.872 | 86 |

| Combination | | A+/T+ in BBM+ | | n BBM+ | | non-A+/T+ in BBM- | | n BBM- | |  |
| --- | --- | --- | --- | --- | --- | --- | --- | --- | --- | --- |
| A*β*42/40 + NfL + pTau217/A*β*40 + pTau217/A*β*42 + pTau181/A*β*40 +  pTau181/A*β*42 | | 0.922 | | 102 | | 0.892 | | 83 | |  |
| A*β*42/40 + pTau217 + NfL + pTau181/A*β*40 | | 0.922 | | 103 | | 0.872 | | 86 | |  |
| A*β*42/40 + NfL + pTau217/A*β*42 + pTau181/A*β*40 | | 0.922 | | 102 | | 0.872 | | 86 | |  |
| pTau217/A*β*42 + pTau181/A*β*40 + pTau181/A*β*42 | | 0.922 | | 154 | | 0.743 | | 214 | |  |
| A*β*42/40 + NfL + pTau217/A*β*40 + pTau181/A*β*40 | | 0.922 | | 102 | | 0.892 | | 83 | |  |
| pTau217 + pTau181/A*β*40 + pTau181/A*β*42 | | 0.921 | | 152 | | 0.749 | | 215 | |  |
| A*β*42/40 + pTau217 + pTau181/A*β*42 | | 0.92 | | 137 | | 0.84 | | 125 | |  |
| A*β*42/40 + pTau217/A*β*40 + pTau181/A*β*42 | | 0.92 | | 138 | | 0.839 | | 124 | |  |
| A*β*42/40 + pTau181 + pTau181/A*β*40 | | 0.92 | | 138 | | 0.826 | | 121 | |  |
| A*β*42/40 + pTau217/A*β*40 + pTau217/A*β*42 + pTau181/A*β*42 | | 0.92 | | 138 | | 0.839 | | 124 | |  |
| A*β*42/40 + pTau181 + pTau181/A*β*40 + pTau181/A*β*42 | | 0.92 | | 137 | | 0.826 | | 121 | |  |
| A*β*42/40 + pTau217 + pTau217/A*β*40 + pTau217/A*β*42 + pTau181/A*β*42 | | 0.919 | | 135 | | 0.852 | | 122 | |  |
| A*β*42/40 + pTau217 + pTau217/A*β*40 + pTau181/A*β*42 | | 0.919 | | 135 | | 0.852 | | 122 | |  |
| A*β*42/40 + pTau217 + pTau217/A*β*42 + pTau181/A*β*42 | | 0.919 | | 136 | | 0.847 | | 124 | |  |
| pTau217 + NfL | | 0.919 | | 136 | | 0.718 | | 170 | |  |
| A*β*42/40 + pTau217 + pTau181/A*β*40 | | 0.917 | | 133 | | 0.837 | | 123 | |  |
| A*β*42/40 + pTau217/A*β*40 + pTau181/A*β*40 | | 0.917 | | 133 | | 0.843 | | 121 | |  |
| A*β*42/40 + pTau217/A*β*40 + pTau181/A*β*40 + pTau181/A*β*42 | | 0.917 | | 133 | | 0.843 | | 121 | |  |
| A*β*42/40 + pTau217/A*β*40 + pTau217/A*β*42 + pTau181/A*β*40 + pTau181/A*β*42 | | 0.917 | | 133 | | 0.843 | | 121 | |  |
| A*β*42/40 + pTau217 + pTau217/A*β*42 + pTau181/A*β*40 + pTau181/A*β*42 | | 0.917 | | 132 | | 0.844 | | 122 | |  |
| A*β*42/40 + pTau217/A*β*40 + pTau217/A*β*42 + pTau181/A*β*40 | | 0.917 | | 133 | | 0.843 | | 121 | |  |
| A*β*42/40 + pTau217 + pTau217/A*β*42 + pTau181/A*β*40 | | 0.917 | | 132 | | 0.844 | | 122 | |  |
| A*β*42/40 + pTau217 + pTau181/A*β*40 + pTau181/A*β*42 | | 0.917 | | 133 | | 0.837 | | 123 | |  |
| pTau217/A*β*40 + pTau217/A*β*42 | | 0.916 | | 178 | | 0.714 | | 245 | |  |
| A*β*42/40 + pTau217 + pTau217/A*β*40 + pTau181/A*β*40 + pTau181/A*β*42 | | 0.916 | | 131 | | 0.85 | | 120 | |  |
|  | |  | |  | |  | | | |  |
|  | Combination | | A+/T+ in BBM+ | | n BBM+ | | non-A+/T+ in BBM- | | n BBM- | |
|  | A*β*42/40 + pTau217 + pTau217/A*β*40 + pTau217/A*β*42 + pTau181/A*β*40 | | 0.916 | | 131 | | 0.85 | | 120 | |
|  | A*β*42/40 + pTau217 + pTau217/A*β*40 + pTau217/A*β*42 + pTau181/A*β*40 +  pTau181/A*β*42 | | 0.916 | | 131 | | 0.85 | | 120 | |
|  | A*β*42/40 + pTau217 + pTau217/A*β*40 + pTau181/A*β*40 | | 0.916 | | 131 | | 0.85 | | 120 | |
|  | A*β*42/40 + pTau181 + NfL + pTau181/A*β*42 | | 0.915 | | 106 | | 0.864 | | 88 | |
|  | A*β*42/40 + pTau181 + pTau181/A*β*42 | | 0.915 | | 142 | | 0.828 | | 122 | |
|  | pTau217 + pTau217/A*β*40 | | 0.915 | | 176 | | 0.725 | | 247 | |
|  | pTau217 + pTau217/A*β*40 + pTau217/A*β*42 | | 0.914 | | 174 | | 0.722 | | 241 | |
|  | pTau181 + NfL + pTau181/A*β*40 | | 0.914 | | 128 | | 0.744 | | 156 | |
|  | pTau217 + pTau217/A*β*42 | | 0.914 | | 175 | | 0.72 | | 243 | |
|  | pTau217/A*β*42 + pTau181/A*β*42 | | 0.913 | | 161 | | 0.729 | | 221 | |
|  | A*β*42/40 + pTau217/A*β*40 | | 0.913 | | 149 | | 0.836 | | 134 | |
|  | A*β*42/40 + pTau217/A*β*40 + pTau217/A*β*42 | | 0.913 | | 149 | | 0.836 | | 134 | |
|  | pTau217/A*β*40 | | 0.912 | | 182 | | 0.714 | | 252 | |
|  | pTau217 | | 0.912 | | 181 | | 0.711 | | 253 | |
|  | A*β*42/40 + pTau217 + pTau217/A*β*42 | | 0.912 | | 147 | | 0.842 | | 133 | |
|  | A*β*42/40 + pTau217/A*β*42 + pTau181/A*β*40 | | 0.912 | | 136 | | 0.831 | | 124 | |
|  | A*β*42/40 + pTau217/A*β*42 + pTau181/A*β*40 + pTau181/A*β*42 | | 0.912 | | 136 | | 0.831 | | 124 | |
|  | A*β*42/40 + pTau217 | | 0.912 | | 148 | | 0.836 | | 134 | |
|  | A*β*42/40 + pTau217 + pTau217/A*β*40 | | 0.911 | | 146 | | 0.847 | | 131 | |
|  | A*β*42/40 + pTau217 + pTau217/A*β*40 + pTau217/A*β*42 | | 0.911 | | 146 | | 0.847 | | 131 | |
|  | pTau181 + NfL + pTau181/A*β*40 + pTau181/A*β*42 | | 0.91 | | 122 | | 0.742 | | 151 | |
|  | A*β*42/40 + pTau181 | | 0.907 | | 150 | | 0.811 | | 127 | |
|  | pTau181 + NfL + pTau181/A*β*42 | | 0.903 | | 124 | | 0.734 | | 154 | |
|  | A*β*42/40 + pTau181 + NfL | | 0.902 | | 112 | | 0.839 | | 93 | |
|  | A*β*42/40 + pTau217/A*β*42 + pTau181/A*β*42 | | 0.902 | | 143 | | 0.82 | | 128 | |
|  | A*β*42/40 + pTau181/A*β*40 | | 0.901 | | 151 | | 0.802 | | 131 | |
|  | A*β*42/40 + NfL + pTau181/A*β*40 | | 0.901 | | 111 | | 0.833 | | 90 | |
|  | A*β*42/40 + NfL + pTau181/A*β*40 + pTau181/A*β*42 | | 0.901 | | 111 | | 0.833 | | 90 | |
|  | A*β*42/40 + pTau181/A*β*40 + pTau181/A*β*42 | | 0.899 | | 148 | | 0.802 | | 131 | |
|  | NfL + pTau181/A*β*40 | | 0.896 | | 135 | | 0.712 | | 163 | |
|  | pTau181 + pTau181/A*β*40 + pTau181/A*β*42 | | 0.894 | | 160 | | 0.728 | | 217 | |
|  | pTau181 + pTau181/A*β*40 | | 0.893 | | 168 | | 0.724 | | 225 | |
|  | pTau217/A*β*42 | | 0.892 | | 185 | | 0.707 | | 249 | |

| Combination | A+/T+ in BBM+ | n BBM+ | non-A+/T+ in BBM- | n BBM- |
| --- | --- | --- | --- | --- |
| NfL + pTau181/A*β*40 + pTau181/A*β*42 | 0.891 | 129 | 0.723 | 155 |
| pTau181 + pTau181/A*β*42 | 0.891 | 165 | 0.723 | 220 |
| A*β*42/40 + NfL + pTau181/A*β*42 | 0.888 | 116 | 0.837 | 92 |
| A*β*42/40 + pTau217/A*β*42 | 0.885 | 156 | 0.819 | 138 |
| NfL + pTau181/A*β*42 | 0.881 | 134 | 0.712 | 160 |
| A*β*42/40 + pTau181/A*β*42 | 0.87 | 161 | 0.77 | 139 |
| pTau181/A*β*40 + pTau181/A*β*42 | 0.869 | 176 | 0.705 | 234 |
| pTau181 + NfL | 0.867 | 143 | 0.714 | 168 |
| pTau181/A*β*40 | 0.866 | 187 | 0.692 | 247 |
| pTau181 | 0.863 | 190 | 0.697 | 244 |
| pTau181/A*β*42 | 0.847 | 189 | 0.682 | 245 |
| A*β*42/40 + NfL | 0.766 | 154 | 0.804 | 102 |
| A*β*42/40 | 0.697 | 267 | 0.689 | 167 |
| NfL | 0.685 | 219 | 0.591 | 215 |

Supplementary table 10	Cutoff combinations — MiniBatch K-Means (all 255 combinations, eight biomarkers). Prevalence of A^+^/T^+^ CSF profile in BBM-positive subgroups and prevalence of non-A^+^/T^+^ CSF profile in BBM-negative subgroups.

| Combination | A+/T+ in BBM+ | n BBM+ | non-A+/T+ in BBM- | n BBM- |
| --- | --- | --- | --- | --- |
| pTau217 + pTau181 + NfL + pTau217/A*β*42 + pTau181/A*β*40 | 0.948 | 115 | 0.78 | 141 |
| pTau181 + NfL + pTau217/A*β*40 + pTau217/A*β*42 + pTau181/A*β*40 | 0.948 | 115 | 0.784 | 139 |
| pTau217 + pTau181 + NfL + pTau217/A*β*40 + pTau217/A*β*42 + pTau181/A*β*40 | 0.948 | 115 | 0.784 | 139 |
| pTau217 + pTau181 + NfL + pTau217/A*β*40 + pTau181/A*β*40 | 0.948 | 115 | 0.789 | 142 |
| pTau181 + NfL + pTau217/A*β*40 + pTau181/A*β*40 | 0.948 | 115 | 0.79 | 143 |
| pTau181 + NfL + pTau217/A*β*42 + pTau181/A*β*40 | 0.948 | 115 | 0.775 | 142 |
| pTau217 + pTau181 + NfL + pTau217/A*β*40 + pTau217/A*β*42 + pTau181/A*β*40 +  pTau181/A*β*42 | 0.946 | 111 | 0.781 | 137 |
| pTau181 + NfL + pTau217/A*β*40 + pTau181/A*β*42 | 0.946 | 112 | 0.78 | 141 |
| pTau181 + NfL + pTau217/A*β*42 + pTau181/A*β*42 | 0.946 | 112 | 0.768 | 142 |

| Combination | | A+/T+ in BBM+ | | n BBM+ | | non-A+/T+ in BBM- | | n BBM- | |
| --- | --- | --- | --- | --- | --- | --- | --- | --- | --- |
| pTau181 + NfL + pTau217/A*β*40 + pTau217/A*β*42 + pTau181/A*β*40 +  pTau181/A*β*42 | | 0.946 | | 111 | | 0.781 | | 137 | |
| pTau217 + pTau181 + NfL + pTau217/A*β*40 + pTau217/A*β*42 + pTau181/A*β*42 | | 0.946 | | 112 | | 0.777 | | 139 | |
| pTau181 + NfL + pTau217/A*β*42 + pTau181/A*β*40 + pTau181/A*β*42 | | 0.946 | | 111 | | 0.771 | | 140 | |
| pTau181 + NfL + pTau217/A*β*40 + pTau181/A*β*40 + pTau181/A*β*42 | | 0.946 | | 111 | | 0.784 | | 139 | |
| pTau181 + NfL + pTau217/A*β*40 + pTau217/A*β*42 + pTau181/A*β*42 | | 0.946 | | 112 | | 0.777 | | 139 | |
| pTau217 + pTau181 + NfL + pTau217/A*β*40 + pTau181/A*β*42 | | 0.946 | | 112 | | 0.779 | | 140 | |
| pTau217 + pTau181 + NfL + pTau217/A*β*40 + pTau181/A*β*40 + pTau181/A*β*42 | | 0.946 | | 111 | | 0.783 | | 138 | |
| pTau217 + pTau181 + NfL + pTau217/A*β*42 + pTau181/A*β*42 | | 0.946 | | 112 | | 0.773 | | 141 | |
| pTau217 + pTau181 + NfL + pTau217/A*β*42 + pTau181/A*β*40 + pTau181/A*β*42 | | 0.946 | | 111 | | 0.777 | | 139 | |
| pTau181 + NfL + pTau217/A*β*42 | | 0.941 | | 118 | | 0.758 | | 149 | |
| pTau217 + pTau181 + NfL + pTau217/A*β*40 + pTau217/A*β*42 | | 0.941 | | 118 | | 0.767 | | 146 | |
| pTau217 + pTau181 + NfL + pTau217/A*β*40 | | 0.941 | | 118 | | 0.768 | | 151 | |
| pTau181 + NfL + pTau217/A*β*40 | | 0.941 | | 118 | | 0.77 | | 152 | |
| pTau217 + pTau181 + NfL + pTau181/A*β*40 | | 0.941 | | 118 | | 0.774 | | 146 | |
| pTau217 + pTau181 + NfL + pTau217/A*β*42 | | 0.941 | | 118 | | 0.764 | | 148 | |
| pTau181 + NfL + pTau217/A*β*40 + pTau217/A*β*42 | | 0.941 | | 118 | | 0.767 | | 146 | |
| A*β*42/40 + pTau217 + pTau181 + NfL + pTau217/A*β*40 + pTau217/A*β*42 | | 0.939 | | 98 | | 0.895 | | 86 | |
| pTau181 + pTau217/A*β*40 + pTau181/A*β*40 | | 0.939 | | 147 | | 0.778 | | 203 | |
| pTau181 + pTau217/A*β*42 + pTau181/A*β*40 | | 0.939 | | 148 | | 0.767 | | 202 | |
| A*β*42/40 + pTau181 + NfL + pTau217/A*β*42 | | 0.939 | | 98 | | 0.876 | | 89 | |
| A*β*42/40 + pTau181 + NfL + pTau217/A*β*40 | | 0.939 | | 98 | | 0.895 | | 86 | |
| pTau181 + pTau217/A*β*40 + pTau217/A*β*42 + pTau181/A*β*40 | | 0.939 | | 147 | | 0.774 | | 199 | |
| A*β*42/40 + pTau217 + pTau181 + NfL + pTau217/A*β*40 | | 0.939 | | 98 | | 0.895 | | 86 | |
| A*β*42/40 + pTau217 + pTau181 + NfL + pTau217/A*β*42 | | 0.939 | | 98 | | 0.886 | | 88 | |
| pTau217 + pTau181 + pTau217/A*β*42 + pTau181/A*β*40 | | 0.939 | | 147 | | 0.771 | | 201 | |
|  | |  | |  | |  | | | |
| Combination | A+/T+ in BBM+ | | n BBM+ | | non-A+/T+ in BBM- | | n BBM- | |  |
| A*β*42/40 + pTau181 + NfL + pTau217/A*β*40 + pTau217/A*β*42 | 0.939 | | 98 | | 0.895 | | 86 | |  |
| pTau181 + pTau217/A*β*42 + pTau181/A*β*40 + pTau181/A*β*42 | 0.938 | | 144 | | 0.768 | | 198 | |  |
| pTau181 + pTau217/A*β*40 + pTau217/A*β*42 + pTau181/A*β*42 | 0.938 | | 145 | | 0.772 | | 197 | |  |
| pTau217 + pTau181 + pTau217/A*β*40 + pTau181/A*β*42 | 0.938 | | 144 | | 0.773 | | 198 | |  |
| pTau181 + pTau217/A*β*42 + pTau181/A*β*42 | 0.938 | | 146 | | 0.765 | | 200 | |  |
| pTau181 + pTau217/A*β*40 + pTau181/A*β*42 | 0.938 | | 145 | | 0.774 | | 199 | |  |
| pTau217 + pTau181 + pTau217/A*β*42 + pTau181/A*β*42 | 0.938 | | 145 | | 0.769 | | 199 | |  |
| pTau217 + pTau181 + pTau217/A*β*40 + pTau181/A*β*40 | 0.938 | | 146 | | 0.777 | | 202 | |  |
| A*β*42/40 + pTau217 + pTau181 + NfL + pTau181/A*β*40 | 0.938 | | 97 | | 0.882 | | 85 | |  |
| A*β*42/40 + pTau181 + NfL + pTau217/A*β*40 + pTau181/A*β*40 | 0.938 | | 96 | | 0.902 | | 82 | |  |
| A*β*42/40 + pTau181 + NfL + pTau217/A*β*40 + pTau181/A*β*42 | 0.938 | | 97 | | 0.904 | | 83 | |  |
| A*β*42/40 + pTau181 + NfL + pTau217/A*β*42 + pTau181/A*β*40 | 0.938 | | 96 | | 0.882 | | 85 | |  |
| A*β*42/40 + pTau181 + NfL + pTau217/A*β*42 + pTau181/A*β*42 | 0.938 | | 97 | | 0.884 | | 86 | |  |
| A*β*42/40 + pTau217 + pTau181 + NfL + pTau217/A*β*42 + pTau181/A*β*42 | 0.938 | | 97 | | 0.894 | | 85 | |  |
| A*β*42/40 + pTau181 + NfL + pTau217/A*β*40 + pTau217/A*β*42 + pTau181/A*β*40 +  pTau181/A*β*42 | 0.938 | | 96 | | 0.902 | | 82 | |  |
| A*β*42/40 + pTau217 + pTau181 + NfL + pTau217/A*β*42 + pTau181/A*β*40 +  pTau181/A*β*42 | 0.938 | | 96 | | 0.893 | | 84 | |  |
| A*β*42/40 + pTau181 + NfL + pTau217/A*β*40 + pTau181/A*β*40 + pTau181/A*β*42 | 0.938 | | 96 | | 0.902 | | 82 | |  |
| A*β*42/40 + pTau181 + NfL + pTau217/A*β*40 + pTau217/A*β*42 + pTau181/A*β*42 | 0.938 | | 97 | | 0.904 | | 83 | |  |
| A*β*42/40 + pTau181 + NfL + pTau217/A*β*40 + pTau217/A*β*42 + pTau181/A*β*40 | 0.938 | | 96 | | 0.902 | | 82 | |  |
| A*β*42/40 + pTau217 + pTau181 + NfL + pTau217/A*β*40 + pTau217/A*β*42 +  pTau181/A*β*42 | 0.938 | | 97 | | 0.904 | | 83 | |  |

| Combination | A+/T+ in BBM+ | n BBM+ | non-A+/T+ in BBM- | n BBM- |
| --- | --- | --- | --- | --- |
| A*β*42/40 + pTau217 + pTau181 + NfL + pTau217/A*β*40 + pTau217/A*β*42 +  pTau181/A*β*40 | 0.938 | 96 | 0.902 | 82 |
| A*β*42/40 + pTau181 + NfL + pTau217/A*β*42 + pTau181/A*β*40 + pTau181/A*β*42 | 0.938 | 96 | 0.882 | 85 |
| A*β*42/40 + pTau217 + pTau181 + NfL + pTau181/A*β*40 + pTau181/A*β*42 | 0.938 | 97 | 0.882 | 85 |
| A*β*42/40 + pTau217 + pTau181 + NfL + pTau217/A*β*40 + pTau181/A*β*42 | 0.938 | 97 | 0.904 | 83 |
| A*β*42/40 + pTau217 + pTau181 + NfL + pTau217/A*β*40 + pTau181/A*β*40 | 0.938 | 96 | 0.902 | 82 |
| A*β*42/40 + pTau217 + pTau181 + NfL + pTau217/A*β*42 + pTau181/A*β*40 | 0.938 | 96 | 0.893 | 84 |
| pTau217 + pTau181 + NfL + pTau181/A*β*40 + pTau181/A*β*42 | 0.938 | 113 | 0.773 | 141 |
| pTau217 + pTau181 + pTau217/A*β*40 + pTau217/A*β*42 + pTau181/A*β*42 | 0.938 | 144 | 0.772 | 197 |
| pTau217 + pTau181 + pTau217/A*β*40 + pTau217/A*β*42 + pTau181/A*β*40 | 0.938 | 146 | 0.774 | 199 |
| A*β*42/40 + pTau217 + pTau181 + NfL + pTau217/A*β*40 + pTau181/A*β*40 +  pTau181/A*β*42 | 0.938 | 96 | 0.902 | 82 |
| A*β*42/40 + pTau217 + pTau181 + NfL + pTau217/A*β*40 + pTau217/A*β*42 + pTau181/A*β*40 + pTau181/A*β*42 | 0.938 | 96 | 0.902 | 82 |
| pTau181 + pTau217/A*β*40 + pTau181/A*β*40 + pTau181/A*β*42 | 0.937 | 143 | 0.777 | 197 |
| pTau217 + pTau181 + pTau217/A*β*40 + pTau217/A*β*42 + pTau181/A*β*40 +  pTau181/A*β*42 | 0.937 | 142 | 0.774 | 195 |
| pTau217 + pTau181 + pTau217/A*β*40 + pTau181/A*β*40 + pTau181/A*β*42 | 0.937 | 142 | 0.776 | 196 |
| pTau217 + pTau181 + pTau217/A*β*42 + pTau181/A*β*40 + pTau181/A*β*42 | 0.937 | 143 | 0.772 | 197 |
| pTau181 + pTau217/A*β*40 + pTau217/A*β*42 + pTau181/A*β*40 + pTau181/A*β*42 | 0.937 | 143 | 0.774 | 195 |
| pTau181 + pTau217/A*β*40 | 0.935 | 153 | 0.765 | 213 |
| pTau181 + pTau217/A*β*40 + pTau217/A*β*42 | 0.935 | 153 | 0.763 | 207 |
| pTau217 + pTau181 + pTau217/A*β*42 | 0.935 | 153 | 0.761 | 209 |
| pTau181 + pTau217/A*β*42 | 0.935 | 154 | 0.757 | 210 |
| pTau217 + pTau181 + pTau217/A*β*40 + pTau217/A*β*42 | 0.934 | 152 | 0.763 | 207 |
|  |  |  |  | |

| Combination | A+/T+ in BBM+ | n BBM+ | non-A+/T+ in BBM- | n BBM- |
| --- | --- | --- | --- | --- |
| NfL + pTau217/A*β*40 + pTau217/A*β*42 + pTau181/A*β*40 | 0.934 | 121 | 0.762 | 143 |
| pTau217 + pTau181 + pTau217/A*β*40 | 0.934 | 152 | 0.764 | 212 |
| NfL + pTau217/A*β*42 + pTau181/A*β*40 | 0.934 | 121 | 0.753 | 146 |
| NfL + pTau217/A*β*40 + pTau181/A*β*40 | 0.934 | 121 | 0.769 | 147 |
| pTau217 + NfL + pTau217/A*β*40 + pTau181/A*β*40 | 0.934 | 121 | 0.767 | 146 |
| pTau217 + NfL + pTau217/A*β*40 + pTau217/A*β*42 + pTau181/A*β*40 | 0.934 | 121 | 0.762 | 143 |
| pTau217 + NfL + pTau217/A*β*42 + pTau181/A*β*40 | 0.934 | 121 | 0.759 | 145 |
| NfL + pTau217/A*β*40 + pTau181/A*β*42 | 0.933 | 120 | 0.766 | 145 |
| pTau217 + NfL + pTau217/A*β*40 + pTau181/A*β*42 | 0.933 | 120 | 0.764 | 144 |
| pTau217 + pTau181 + pTau181/A*β*40 | 0.933 | 150 | 0.767 | 206 |
| NfL + pTau217/A*β*42 + pTau181/A*β*42 | 0.933 | 120 | 0.753 | 146 |
| pTau217 + NfL + pTau217/A*β*42 + pTau181/A*β*42 | 0.933 | 120 | 0.759 | 145 |
| NfL + pTau217/A*β*40 + pTau217/A*β*42 + pTau181/A*β*42 | 0.933 | 120 | 0.762 | 143 |
| pTau217 + NfL + pTau217/A*β*40 + pTau217/A*β*42 + pTau181/A*β*42 | 0.933 | 120 | 0.762 | 143 |
| NfL + pTau217/A*β*42 + pTau181/A*β*40 + pTau181/A*β*42 | 0.932 | 117 | 0.761 | 142 |
| NfL + pTau217/A*β*40 + pTau181/A*β*40 + pTau181/A*β*42 | 0.932 | 117 | 0.773 | 141 |
| NfL + pTau217/A*β*40 + pTau217/A*β*42 + pTau181/A*β*40 + pTau181/A*β*42 | 0.932 | 117 | 0.77 | 139 |
| pTau217 + NfL + pTau217/A*β*40 + pTau217/A*β*42 + pTau181/A*β*40 +  pTau181/A*β*42 | 0.932 | 117 | 0.77 | 139 |
| pTau217 + NfL + pTau217/A*β*40 + pTau181/A*β*40 + pTau181/A*β*42 | 0.932 | 117 | 0.771 | 140 |
| pTau217 + NfL + pTau217/A*β*42 + pTau181/A*β*40 + pTau181/A*β*42 | 0.932 | 117 | 0.766 | 141 |
| pTau217 + pTau181 + pTau181/A*β*40 + pTau181/A*β*42 | 0.931 | 145 | 0.769 | 199 |
| A*β*42/40 + pTau181 + pTau217/A*β*42 | 0.931 | 130 | 0.85 | 120 |
| A*β*42/40 + pTau181 + pTau217/A*β*40 + pTau217/A*β*42 + pTau181/A*β*42 | 0.93 | 128 | 0.868 | 114 |
| A*β*42/40 + pTau217 + pTau181 + pTau217/A*β*40 + pTau217/A*β*42 | 0.93 | 128 | 0.863 | 117 |
|  |  |  |  | |

| Combination | A+/T+ in BBM+ | n BBM+ | non-A+/T+ in BBM- | n BBM- |
| --- | --- | --- | --- | --- |
| A*β*42/40 + pTau217 + pTau181 + pTau217/A*β*42 | 0.93 | 129 | 0.857 | 119 |
| pTau217/A*β*40 + pTau217/A*β*42 + pTau181/A*β*42 | 0.93 | 157 | 0.744 | 215 |
| A*β*42/40 + pTau217 + pTau181 + pTau217/A*β*40 | 0.93 | 128 | 0.863 | 117 |
| A*β*42/40 + pTau181 + pTau217/A*β*40 | 0.93 | 129 | 0.863 | 117 |
| pTau217/A*β*40 + pTau181/A*β*42 | 0.93 | 157 | 0.747 | 217 |
| A*β*42/40 + pTau217 + pTau181 + NfL | 0.93 | 100 | 0.876 | 89 |
| A*β*42/40 + pTau181 + pTau217/A*β*40 + pTau181/A*β*42 | 0.93 | 128 | 0.868 | 114 |
| A*β*42/40 + pTau181 + pTau217/A*β*40 + pTau217/A*β*42 | 0.93 | 129 | 0.863 | 117 |
| A*β*42/40 + pTau181 + pTau217/A*β*42 + pTau181/A*β*42 | 0.93 | 129 | 0.855 | 117 |
| pTau217 + pTau181 + NfL + pTau181/A*β*42 | 0.93 | 115 | 0.769 | 143 |
| A*β*42/40 + pTau217 + pTau181 + pTau217/A*β*42 + pTau181/A*β*42 | 0.93 | 128 | 0.862 | 116 |
| pTau217 + pTau217/A*β*40 + pTau217/A*β*42 + pTau181/A*β*40 | 0.929 | 154 | 0.756 | 213 |
| pTau217 + pTau217/A*β*42 + pTau181/A*β*42 | 0.929 | 155 | 0.756 | 213 |
| pTau217/A*β*40 + pTau217/A*β*42 + pTau181/A*β*40 | 0.929 | 156 | 0.747 | 217 |
| A*β*42/40 + pTau217 + pTau181 + pTau181/A*β*40 | 0.929 | 127 | 0.853 | 116 |
| A*β*42/40 + pTau181 + pTau217/A*β*42 + pTau181/A*β*40 | 0.929 | 127 | 0.853 | 116 |
| pTau217 + pTau217/A*β*40 + pTau181/A*β*40 | 0.929 | 154 | 0.759 | 216 |
| A*β*42/40 + pTau181 + pTau217/A*β*40 + pTau181/A*β*40 | 0.929 | 126 | 0.867 | 113 |
| A*β*42/40 + pTau217 + pTau181 + pTau217/A*β*42 + pTau181/A*β*40 +  pTau181/A*β*42 | 0.929 | 126 | 0.861 | 115 |
| A*β*42/40 + pTau181 + pTau217/A*β*40 + pTau217/A*β*42 + pTau181/A*β*40 +  pTau181/A*β*42 | 0.929 | 126 | 0.867 | 113 |
| A*β*42/40 + pTau181 + pTau217/A*β*40 + pTau217/A*β*42 + pTau181/A*β*40 | 0.929 | 126 | 0.867 | 113 |
| A*β*42/40 + pTau217 + pTau181 + pTau181/A*β*40 + pTau181/A*β*42 | 0.929 | 127 | 0.853 | 116 |
| A*β*42/40 + pTau181 + pTau217/A*β*40 + pTau181/A*β*40 + pTau181/A*β*42 | 0.929 | 126 | 0.867 | 113 |
|  |  |  |  | |

| Combination | A+/T+ in BBM+ | n BBM+ | non-A+/T+ in BBM- | n BBM- |
| --- | --- | --- | --- | --- |
| A*β*42/40 + pTau217 + pTau181 + NfL + pTau181/A*β*42 | 0.929 | 99 | 0.884 | 86 |
| pTau217 + pTau217/A*β*40 + pTau217/A*β*42 + pTau181/A*β*42 | 0.929 | 154 | 0.758 | 211 |
| pTau217/A*β*40 + pTau181/A*β*40 | 0.929 | 156 | 0.751 | 221 |
| pTau217 + pTau217/A*β*40 + pTau181/A*β*42 | 0.929 | 154 | 0.759 | 212 |
| pTau217 + pTau217/A*β*42 + pTau181/A*β*40 | 0.929 | 155 | 0.753 | 215 |
| A*β*42/40 + pTau181 + pTau217/A*β*42 + pTau181/A*β*40 + pTau181/A*β*42 | 0.929 | 127 | 0.853 | 116 |
| A*β*42/40 + pTau217 + pTau181 + pTau217/A*β*40 + pTau217/A*β*42 +  pTau181/A*β*42 | 0.929 | 127 | 0.868 | 114 |
| A*β*42/40 + pTau217 + pTau181 + pTau217/A*β*40 + pTau181/A*β*42 | 0.929 | 127 | 0.868 | 114 |
| A*β*42/40 + pTau217 + pTau181 + pTau217/A*β*42 + pTau181/A*β*40 | 0.929 | 126 | 0.861 | 115 |
| A*β*42/40 + pTau217 + pTau181 + pTau217/A*β*40 + pTau217/A*β*42 + pTau181/A*β*40 + pTau181/A*β*42 | 0.928 | 125 | 0.867 | 113 |
| A*β*42/40 + pTau217 + pTau181 + pTau217/A*β*40 + pTau217/A*β*42 +  pTau181/A*β*40 | 0.928 | 125 | 0.867 | 113 |
| pTau217/A*β*40 + pTau217/A*β*42 + pTau181/A*β*40 + pTau181/A*β*42 | 0.928 | 152 | 0.752 | 210 |
| A*β*42/40 + pTau217 + pTau181 + pTau217/A*β*40 + pTau181/A*β*40 | 0.928 | 125 | 0.867 | 113 |
| pTau217/A*β*40 + pTau181/A*β*40 + pTau181/A*β*42 | 0.928 | 152 | 0.755 | 212 |
| pTau217 + pTau181 + NfL | 0.928 | 125 | 0.75 | 156 |
| A*β*42/40 + pTau217 + pTau181 + pTau217/A*β*40 + pTau181/A*β*40 +  pTau181/A*β*42 | 0.928 | 125 | 0.867 | 113 |
| pTau217 + pTau217/A*β*40 + pTau217/A*β*42 + pTau181/A*β*40 + pTau181/A*β*42 | 0.927 | 150 | 0.763 | 207 |
| pTau217 + pTau217/A*β*40 + pTau181/A*β*40 + pTau181/A*β*42 | 0.927 | 150 | 0.764 | 208 |
| pTau217 + pTau217/A*β*42 + pTau181/A*β*40 + pTau181/A*β*42 | 0.927 | 151 | 0.761 | 209 |
| pTau217 + NfL + pTau181/A*β*40 | 0.927 | 124 | 0.753 | 150 |
| A*β*42/40 + pTau217 + NfL + pTau217/A*β*40 + pTau217/A*β*42 | 0.926 | 108 | 0.88 | 92 |

| Combination | | A+/T+ in BBM+ | | n BBM+ | | non-A+/T+ in BBM- | | n BBM- | |
| --- | --- | --- | --- | --- | --- | --- | --- | --- | --- |
| A*β*42/40 + NfL + pTau217/A*β*42 | | 0.926 | | 108 | | 0.863 | | 95 | |
| A*β*42/40 + NfL + pTau217/A*β*40 + pTau217/A*β*42 | | 0.926 | | 108 | | 0.88 | | 92 | |
| A*β*42/40 + NfL + pTau217/A*β*40 | | 0.926 | | 108 | | 0.88 | | 92 | |
| pTau217 + pTau181 + pTau181/A*β*42 | | 0.926 | | 148 | | 0.766 | | 201 | |
| A*β*42/40 + pTau217 + NfL + pTau217/A*β*40 | | 0.926 | | 108 | | 0.88 | | 92 | |
| A*β*42/40 + pTau217 + NfL + pTau217/A*β*42 | | 0.926 | | 108 | | 0.872 | | 94 | |
| pTau217/A*β*42 + pTau181/A*β*40 | | 0.925 | | 159 | | 0.741 | | 220 | |
| pTau217 + pTau181 | | 0.925 | | 160 | | 0.751 | | 217 | |
| pTau217 + NfL + pTau217/A*β*40 | | 0.924 | | 131 | | 0.736 | | 163 | |
| NfL + pTau217/A*β*40 + pTau217/A*β*42 | | 0.924 | | 131 | | 0.734 | | 158 | |
| pTau217 + NfL + pTau217/A*β*42 | | 0.924 | | 131 | | 0.731 | | 160 | |
| NfL + pTau217/A*β*42 | | 0.924 | | 131 | | 0.727 | | 161 | |
| NfL + pTau217/A*β*40 | | 0.924 | | 131 | | 0.733 | | 165 | |
| A*β*42/40 + pTau217 + pTau181 | | 0.924 | | 131 | | 0.85 | | 120 | |
| pTau217 + pTau181/A*β*40 | | 0.924 | | 158 | | 0.75 | | 220 | |
| A*β*42/40 + NfL + pTau217/A*β*42 + pTau181/A*β*42 | | 0.924 | | 105 | | 0.875 | | 88 | |
| pTau217 + NfL + pTau217/A*β*40 + pTau217/A*β*42 | | 0.924 | | 131 | | 0.734 | | 158 | |
| A*β*42/40 + pTau217 + NfL + pTau217/A*β*40 + pTau181/A*β*42 | | 0.924 | | 105 | | 0.894 | | 85 | |
| pTau217 + NfL + pTau181/A*β*40 + pTau181/A*β*42 | | 0.924 | | 119 | | 0.762 | | 143 | |
| A*β*42/40 + pTau217 + NfL + pTau217/A*β*42 + pTau181/A*β*42 | | 0.924 | | 105 | | 0.885 | | 87 | |
| A*β*42/40 + pTau217 + NfL + pTau217/A*β*40 + pTau217/A*β*42 + pTau181/A*β*42 | | 0.924 | | 105 | | 0.894 | | 85 | |
| A*β*42/40 + NfL + pTau217/A*β*40 + pTau217/A*β*42 + pTau181/A*β*42 | | 0.924 | | 105 | | 0.894 | | 85 | |
| A*β*42/40 + NfL + pTau217/A*β*40 + pTau181/A*β*42 | | 0.924 | | 105 | | 0.894 | | 85 | |
| A*β*42/40 + pTau181 + NfL + pTau181/A*β*40 | | 0.923 | | 104 | | 0.862 | | 87 | |
| A*β*42/40 + pTau217 + pTau181 + pTau181/A*β*42 | | 0.923 | | 130 | | 0.855 | | 117 | |
| A*β*42/40 + pTau181 + NfL + pTau181/A*β*40 + pTau181/A*β*42 | | 0.923 | | 104 | | 0.862 | | 87 | |
| pTau217/A*β*42 + pTau181/A*β*40 + pTau181/A*β*42 | | 0.923 | | 155 | | 0.746 | | 213 | |
| pTau217 + pTau181/A*β*40 + pTau181/A*β*42 | | 0.922 | | 153 | | 0.758 | | 211 | |
| A*β*42/40 + NfL + pTau217/A*β*42 + pTau181/A*β*40 | | 0.922 | | 102 | | 0.872 | | 86 | |
|  | |  | |  | |  | | | |
| Combination | A+/T+ in BBM+ | | n BBM+ | | non-A+/T+ in BBM- | | n BBM- | |  |
| A*β*42/40 + NfL + pTau217/A*β*40 + pTau181/A*β*40 | 0.922 | | 102 | | 0.892 | | 83 | |  |
| A*β*42/40 + pTau217 + NfL + pTau217/A*β*40 + pTau181/A*β*40 | 0.922 | | 102 | | 0.892 | | 83 | |  |
| A*β*42/40 + pTau217 + NfL + pTau217/A*β*42 + pTau181/A*β*40 | 0.922 | | 102 | | 0.882 | | 85 | |  |
| A*β*42/40 + pTau217 + NfL + pTau217/A*β*42 + pTau181/A*β*40 + pTau181/A*β*42 | 0.922 | | 102 | | 0.882 | | 85 | |  |
| A*β*42/40 + NfL + pTau217/A*β*40 + pTau217/A*β*42 + pTau181/A*β*40 | 0.922 | | 102 | | 0.892 | | 83 | |  |
| A*β*42/40 + pTau217 + NfL + pTau217/A*β*40 + pTau217/A*β*42 + pTau181/A*β*40 | 0.922 | | 102 | | 0.892 | | 83 | |  |
| A*β*42/40 + pTau217 + NfL + pTau217/A*β*40 + pTau181/A*β*40 + pTau181/A*β*42 | 0.922 | | 102 | | 0.892 | | 83 | |  |
| A*β*42/40 + pTau217 + NfL + pTau217/A*β*40 + pTau217/A*β*42 + pTau181/A*β*40 +  pTau181/A*β*42 | 0.922 | | 102 | | 0.892 | | 83 | |  |
| A*β*42/40 + pTau217 + NfL + pTau181/A*β*40 + pTau181/A*β*42 | 0.922 | | 103 | | 0.872 | | 86 | |  |
| A*β*42/40 + NfL + pTau217/A*β*40 + pTau181/A*β*40 + pTau181/A*β*42 | 0.922 | | 102 | | 0.892 | | 83 | |  |
| A*β*42/40 + NfL + pTau217/A*β*42 + pTau181/A*β*40 + pTau181/A*β*42 | 0.922 | | 102 | | 0.872 | | 86 | |  |
| A*β*42/40 + NfL + pTau217/A*β*40 + pTau217/A*β*42 + pTau181/A*β*40 +  pTau181/A*β*42 | 0.922 | | 102 | | 0.892 | | 83 | |  |
| A*β*42/40 + pTau217 + NfL + pTau181/A*β*40 | 0.922 | | 103 | | 0.872 | | 86 | |  |
| A*β*42/40 + pTau181 + pTau181/A*β*40 + pTau181/A*β*42 | 0.92 | | 137 | | 0.826 | | 121 | |  |
| A*β*42/40 + pTau181 + pTau181/A*β*40 | 0.92 | | 138 | | 0.826 | | 121 | |  |
| A*β*42/40 + pTau217/A*β*40 + pTau181/A*β*42 | 0.92 | | 138 | | 0.839 | | 124 | |  |
| A*β*42/40 + pTau217/A*β*40 + pTau217/A*β*42 + pTau181/A*β*42 | 0.92 | | 138 | | 0.839 | | 124 | |  |
| A*β*42/40 + pTau217 + pTau217/A*β*42 + pTau181/A*β*42 | 0.919 | | 136 | | 0.861 | | 122 | |  |
| A*β*42/40 + pTau217 + pTau217/A*β*40 + pTau181/A*β*42 | 0.919 | | 135 | | 0.867 | | 120 | |  |
| pTau217 + NfL + pTau181/A*β*42 | 0.919 | | 123 | | 0.755 | | 147 | |  |
| A*β*42/40 + pTau217 + pTau217/A*β*40 + pTau217/A*β*42 + pTau181/A*β*42 | 0.919 | | 135 | | 0.867 | | 120 | |  |
| A*β*42/40 + pTau217 + NfL | 0.918 | | 110 | | 0.863 | | 95 | |  |

| Combination | A+/T+ in BBM+ | n BBM+ | non-A+/T+ in BBM- | n BBM- |
| --- | --- | --- | --- | --- |
| pTau217 + pTau181/A*β*42 | 0.918 | 158 | 0.753 | 215 |
| A*β*42/40 + pTau217/A*β*40 + pTau181/A*β*40 + pTau181/A*β*42 | 0.917 | 133 | 0.843 | 121 |
| A*β*42/40 + pTau217 + pTau217/A*β*42 + pTau181/A*β*40 | 0.917 | 132 | 0.858 | 120 |
| A*β*42/40 + pTau217 + pTau181/A*β*40 | 0.917 | 133 | 0.851 | 121 |
| A*β*42/40 + pTau217/A*β*40 + pTau217/A*β*42 + pTau181/A*β*40 | 0.917 | 133 | 0.843 | 121 |
| A*β*42/40 + pTau217 + pTau181/A*β*40 + pTau181/A*β*42 | 0.917 | 133 | 0.851 | 121 |
| A*β*42/40 + pTau217/A*β*40 + pTau181/A*β*40 | 0.917 | 133 | 0.843 | 121 |
| A*β*42/40 + pTau217/A*β*40 + pTau217/A*β*42 + pTau181/A*β*40 + pTau181/A*β*42 | 0.917 | 133 | 0.843 | 121 |
| A*β*42/40 + pTau217 + pTau217/A*β*42 + pTau181/A*β*40 + pTau181/A*β*42 | 0.917 | 132 | 0.858 | 120 |
| A*β*42/40 + pTau217 + pTau217/A*β*40 + pTau181/A*β*40 + pTau181/A*β*42 | 0.916 | 131 | 0.864 | 118 |
| A*β*42/40 + pTau217 + NfL + pTau181/A*β*42 | 0.916 | 107 | 0.875 | 88 |
| A*β*42/40 + pTau217 + pTau217/A*β*40 + pTau181/A*β*40 | 0.916 | 131 | 0.864 | 118 |
| pTau217/A*β*40 + pTau217/A*β*42 | 0.916 | 179 | 0.714 | 245 |
| A*β*42/40 + pTau217 + pTau217/A*β*40 + pTau217/A*β*42 + pTau181/A*β*40 +  pTau181/A*β*42 | 0.916 | 131 | 0.864 | 118 |
| A*β*42/40 + pTau217 + pTau217/A*β*40 + pTau217/A*β*42 + pTau181/A*β*40 | 0.916 | 131 | 0.864 | 118 |
| A*β*42/40 + pTau181 + pTau181/A*β*42 | 0.915 | 142 | 0.828 | 122 |
| pTau217 + pTau217/A*β*40 | 0.915 | 176 | 0.728 | 243 |
| A*β*42/40 + pTau181 + NfL + pTau181/A*β*42 | 0.915 | 106 | 0.864 | 88 |
| pTau217 + pTau217/A*β*40 + pTau217/A*β*42 | 0.914 | 175 | 0.727 | 238 |
| pTau217/A*β*42 + pTau181/A*β*42 | 0.914 | 162 | 0.739 | 218 |
| pTau217 + NfL | 0.913 | 138 | 0.716 | 169 |
| pTau181 + NfL + pTau181/A*β*40 | 0.913 | 127 | 0.744 | 156 |
| A*β*42/40 + pTau217 + pTau181/A*β*42 | 0.913 | 138 | 0.854 | 123 |
| A*β*42/40 + pTau217/A*β*40 + pTau217/A*β*42 | 0.912 | 148 | 0.836 | 134 |
| A*β*42/40 + pTau217/A*β*40 | 0.912 | 148 | 0.836 | 134 |
| A*β*42/40 + pTau217/A*β*42 + pTau181/A*β*40 + pTau181/A*β*42 | 0.912 | 136 | 0.831 | 124 |
| pTau217/A*β*40 | 0.912 | 182 | 0.714 | 252 |
| A*β*42/40 + pTau217/A*β*42 + pTau181/A*β*40 | 0.912 | 136 | 0.831 | 124 |
| A*β*42/40 + pTau217 + pTau217/A*β*40 | 0.91 | 145 | 0.86 | 129 |

| Combination | A+/T+ in BBM+ | n BBM+ | non-A+/T+ in BBM- | n BBM- |
| --- | --- | --- | --- | --- |
| pTau217 + pTau217/A*β*42 | 0.91 | 177 | 0.725 | 240 |
| A*β*42/40 + pTau217 + pTau217/A*β*40 + pTau217/A*β*42 | 0.91 | 145 | 0.86 | 129 |
| pTau181 + NfL + pTau181/A*β*40 + pTau181/A*β*42 | 0.91 | 122 | 0.742 | 151 |
| A*β*42/40 + pTau217 + pTau217/A*β*42 | 0.905 | 147 | 0.855 | 131 |
| pTau217 | 0.903 | 185 | 0.715 | 249 |
| pTau181 + NfL + pTau181/A*β*42 | 0.903 | 124 | 0.739 | 153 |
| A*β*42/40 + pTau217/A*β*42 + pTau181/A*β*42 | 0.902 | 143 | 0.827 | 127 |
| A*β*42/40 + pTau181 | 0.901 | 151 | 0.811 | 127 |
| A*β*42/40 + NfL + pTau181/A*β*40 + pTau181/A*β*42 | 0.901 | 111 | 0.833 | 90 |
| A*β*42/40 + pTau181/A*β*40 | 0.901 | 151 | 0.795 | 132 |
| A*β*42/40 + NfL + pTau181/A*β*40 | 0.901 | 111 | 0.833 | 90 |
| A*β*42/40 + pTau181/A*β*40 + pTau181/A*β*42 | 0.899 | 149 | 0.795 | 132 |
| A*β*42/40 + pTau217 | 0.899 | 149 | 0.848 | 132 |
| NfL + pTau181/A*β*40 | 0.896 | 134 | 0.712 | 163 |
| A*β*42/40 + pTau181 + NfL | 0.894 | 113 | 0.839 | 93 |
| pTau181 + pTau181/A*β*40 + pTau181/A*β*42 | 0.894 | 161 | 0.734 | 214 |
| pTau181 + pTau181/A*β*40 | 0.893 | 168 | 0.726 | 223 |
| pTau181 + pTau181/A*β*42 | 0.892 | 166 | 0.731 | 216 |
| pTau217/A*β*42 | 0.892 | 186 | 0.71 | 248 |
| NfL + pTau181/A*β*40 + pTau181/A*β*42 | 0.891 | 129 | 0.723 | 155 |
| A*β*42/40 + NfL + pTau181/A*β*42 | 0.889 | 117 | 0.837 | 92 |
| A*β*42/40 + pTau217/A*β*42 | 0.884 | 155 | 0.825 | 137 |
| NfL + pTau181/A*β*42 | 0.881 | 135 | 0.717 | 159 |
| A*β*42/40 + pTau181/A*β*42 | 0.871 | 163 | 0.77 | 139 |
| pTau181/A*β*40 + pTau181/A*β*42 | 0.871 | 178 | 0.708 | 233 |
| pTau181/A*β*40 | 0.866 | 187 | 0.692 | 247 |
| pTau181 + NfL | 0.861 | 144 | 0.714 | 168 |
| pTau181 | 0.859 | 192 | 0.698 | 242 |
| pTau181/A*β*42 | 0.849 | 192 | 0.69 | 242 |
| A*β*42/40 + NfL | 0.765 | 153 | 0.796 | 103 |
| A*β*42/40 | 0.695 | 266 | 0.685 | 168 |
| NfL | 0.683 | 218 | 0.588 | 216 |

Supplementary table 11 Cutoff combinations — Agglomerative (Ward) (all 255 combinations, eight biomarkers). Prevalence of A^+^/T^+^ CSF profile in BBM-positive subgroups and prevalence of non-A^+^/T^+^ CSF profile in BBM-negative subgroups.

| Combination | A+/T+ in BBM+ | n BBM+ | non-A+/T+ in BBM- | n BBM- |
| --- | --- | --- | --- | --- |
| pTau181 + NfL + pTau217/A*β*40 + pTau181/A*β*40 | 0.956 | 113 | 0.747 | 150 |
| pTau217 + pTau181 + NfL + pTau217/A*β*40 + pTau181/A*β*40 | 0.956 | 113 | 0.752 | 149 |
| pTau181 + NfL + pTau217/A*β*40 + pTau217/A*β*42 + pTau181/A*β*40 | 0.955 | 111 | 0.752 | 145 |
| pTau217 + pTau181 + NfL + pTau217/A*β*40 + pTau217/A*β*42 + pTau181/A*β*40 | 0.955 | 111 | 0.752 | 145 |
| pTau217 + pTau181 + NfL + pTau217/A*β*40 + pTau181/A*β*40 + pTau181/A*β*42 | 0.954 | 108 | 0.753 | 146 |
| pTau181 + NfL + pTau217/A*β*40 + pTau181/A*β*42 | 0.954 | 109 | 0.757 | 148 |
| pTau181 + NfL + pTau217/A*β*40 + pTau217/A*β*42 + pTau181/A*β*42 | 0.954 | 108 | 0.76 | 146 |
| pTau181 + NfL + pTau217/A*β*40 + pTau181/A*β*40 + pTau181/A*β*42 | 0.954 | 108 | 0.753 | 146 |
| pTau217 + pTau181 + NfL + pTau217/A*β*40 + pTau181/A*β*42 | 0.954 | 109 | 0.757 | 148 |
| pTau217 + pTau181 + NfL + pTau217/A*β*40 + pTau217/A*β*42 + pTau181/A*β*42 | 0.954 | 108 | 0.76 | 146 |
| pTau217 + pTau181 + NfL + pTau217/A*β*40 + pTau217/A*β*42 + pTau181/A*β*40 +  pTau181/A*β*42 | 0.953 | 107 | 0.757 | 144 |
| pTau181 + NfL + pTau217/A*β*40 + pTau217/A*β*42 + pTau181/A*β*40 +  pTau181/A*β*42 | 0.953 | 107 | 0.757 | 144 |
| pTau181 + NfL + pTau217/A*β*40 | 0.949 | 117 | 0.745 | 157 |
| pTau217 + pTau181 + NfL + pTau217/A*β*40 | 0.949 | 117 | 0.75 | 156 |
| A*β*42/40 + pTau181 + NfL + pTau217/A*β*40 | 0.948 | 97 | 0.895 | 86 |
| pTau181 + NfL + pTau217/A*β*40 + pTau217/A*β*42 | 0.948 | 115 | 0.748 | 151 |
| A*β*42/40 + pTau181 + NfL + pTau217/A*β*40 + pTau217/A*β*42 | 0.948 | 96 | 0.895 | 86 |
| A*β*42/40 + pTau181 + NfL + pTau217/A*β*40 + pTau181/A*β*42 | 0.948 | 96 | 0.904 | 83 |
| A*β*42/40 + pTau217 + pTau181 + NfL + pTau217/A*β*40 + pTau217/A*β*42 | 0.948 | 96 | 0.895 | 86 |
| A*β*42/40 + pTau217 + pTau181 + NfL + pTau217/A*β*40 + pTau181/A*β*42 | 0.948 | 96 | 0.904 | 83 |

| Combination | A+/T+ in BBM+ | n BBM+ | non-A+/T+ in BBM- | n BBM- |
| --- | --- | --- | --- | --- |
| A*β*42/40 + pTau217 + pTau181 + NfL + pTau217/A*β*40 | 0.948 | 97 | 0.895 | 86 |
| pTau217 + pTau181 + NfL + pTau217/A*β*40 + pTau217/A*β*42 | 0.948 | 115 | 0.748 | 151 |
| A*β*42/40 + pTau217 + pTau181 + NfL + pTau217/A*β*40 + pTau181/A*β*40 +  pTau181/A*β*42 | 0.947 | 95 | 0.901 | 81 |
| pTau181 + NfL + pTau217/A*β*42 + pTau181/A*β*40 | 0.947 | 113 | 0.738 | 149 |
| A*β*42/40 + pTau217 + pTau181 + NfL + pTau217/A*β*40 + pTau181/A*β*40 | 0.947 | 95 | 0.901 | 81 |
| A*β*42/40 + pTau181 + NfL + pTau217/A*β*40 + pTau217/A*β*42 + pTau181/A*β*40 | 0.947 | 94 | 0.901 | 81 |
| pTau217 + pTau181 + NfL + pTau217/A*β*42 + pTau181/A*β*40 | 0.947 | 113 | 0.743 | 148 |
| A*β*42/40 + pTau217 + pTau181 + NfL + pTau217/A*β*40 + pTau217/A*β*42 + pTau181/A*β*40 + pTau181/A*β*42 | 0.947 | 94 | 0.901 | 81 |
| A*β*42/40 + pTau217 + pTau181 + NfL + pTau217/A*β*40 + pTau217/A*β*42 +  pTau181/A*β*40 | 0.947 | 94 | 0.901 | 81 |
| A*β*42/40 + pTau181 + NfL + pTau217/A*β*40 + pTau217/A*β*42 + pTau181/A*β*40 +  pTau181/A*β*42 | 0.947 | 94 | 0.901 | 81 |
| A*β*42/40 + pTau181 + NfL + pTau217/A*β*40 + pTau181/A*β*40 | 0.947 | 95 | 0.901 | 81 |
| A*β*42/40 + pTau181 + NfL + pTau217/A*β*40 + pTau181/A*β*40 + pTau181/A*β*42 | 0.947 | 95 | 0.901 | 81 |
| A*β*42/40 + pTau181 + NfL + pTau217/A*β*40 + pTau217/A*β*42 + pTau181/A*β*42 | 0.947 | 95 | 0.904 | 83 |
| A*β*42/40 + pTau217 + pTau181 + NfL + pTau217/A*β*40 + pTau217/A*β*42 +  pTau181/A*β*42 | 0.947 | 95 | 0.904 | 83 |
| pTau217 + pTau181 + NfL + pTau217/A*β*42 + pTau181/A*β*40 + pTau181/A*β*42 | 0.945 | 109 | 0.748 | 147 |
| pTau217 + pTau181 + NfL + pTau217/A*β*42 + pTau181/A*β*42 | 0.945 | 110 | 0.752 | 149 |
| pTau181 + NfL + pTau217/A*β*42 + pTau181/A*β*40 + pTau181/A*β*42 | 0.945 | 109 | 0.743 | 148 |
| pTau181 + NfL + pTau217/A*β*42 + pTau181/A*β*42 | 0.945 | 110 | 0.747 | 150 |

| Combination | A+/T+ in BBM+ | n BBM+ | non-A+/T+ in BBM- | n BBM- |
| --- | --- | --- | --- | --- |
| pTau181 + pTau217/A*β*40 + pTau181/A*β*40 | 0.942 | 139 | 0.742 | 217 |
| pTau217 + pTau181 + pTau217/A*β*40 + pTau181/A*β*40 | 0.942 | 138 | 0.749 | 215 |
| pTau181 + pTau217/A*β*40 + pTau217/A*β*42 + pTau181/A*β*40 | 0.942 | 137 | 0.749 | 211 |
| pTau217 + pTau181 + pTau217/A*β*40 + pTau217/A*β*42 + pTau181/A*β*40 | 0.941 | 136 | 0.749 | 211 |
| pTau217 + pTau181 + NfL + pTau217/A*β*42 | 0.941 | 118 | 0.74 | 154 |
| pTau181 + NfL + pTau217/A*β*42 | 0.941 | 118 | 0.735 | 155 |
| pTau181 + pTau217/A*β*40 + pTau181/A*β*42 | 0.941 | 135 | 0.746 | 213 |
| NfL + pTau217/A*β*40 + pTau181/A*β*40 | 0.941 | 118 | 0.737 | 152 |
| A*β*42/40 + pTau217 + pTau181 + NfL | 0.941 | 101 | 0.876 | 89 |
| pTau217 + pTau181 + NfL + pTau181/A*β*40 | 0.94 | 117 | 0.739 | 153 |
| pTau181 + pTau217/A*β*40 + pTau217/A*β*42 + pTau181/A*β*42 | 0.94 | 134 | 0.752 | 210 |
| pTau181 + pTau217/A*β*40 + pTau181/A*β*40 + pTau181/A*β*42 | 0.94 | 134 | 0.744 | 211 |
| pTau181 + pTau217/A*β*40 + pTau217/A*β*42 + pTau181/A*β*40 + pTau181/A*β*42 | 0.94 | 133 | 0.75 | 208 |
| pTau217 + pTau181 + pTau217/A*β*40 + pTau217/A*β*42 + pTau181/A*β*42 | 0.94 | 133 | 0.752 | 210 |
| pTau217 + pTau181 + pTau217/A*β*40 + pTau181/A*β*40 + pTau181/A*β*42 | 0.94 | 133 | 0.748 | 210 |
| pTau217 + NfL + pTau217/A*β*40 + pTau181/A*β*40 | 0.94 | 117 | 0.742 | 151 |
| pTau217 + pTau181 + pTau217/A*β*40 + pTau181/A*β*42 | 0.94 | 134 | 0.75 | 212 |
| NfL + pTau217/A*β*40 + pTau181/A*β*42 | 0.94 | 116 | 0.747 | 150 |
| NfL + pTau217/A*β*40 + pTau217/A*β*42 + pTau181/A*β*40 | 0.94 | 116 | 0.741 | 147 |
| A*β*42/40 + pTau217 + pTau181 + NfL + pTau217/A*β*42 | 0.939 | 99 | 0.876 | 89 |
| A*β*42/40 + pTau181 + NfL + pTau217/A*β*42 | 0.939 | 99 | 0.867 | 90 |
| A*β*42/40 + pTau217 + pTau181 + NfL + pTau181/A*β*40 | 0.939 | 98 | 0.881 | 84 |
| pTau217 + NfL + pTau217/A*β*40 + pTau217/A*β*42 + pTau181/A*β*42 | 0.939 | 114 | 0.75 | 148 |
| pTau217 + NfL + pTau217/A*β*40 + pTau217/A*β*42 + pTau181/A*β*40 | 0.939 | 115 | 0.741 | 147 |
| NfL + pTau217/A*β*40 + pTau217/A*β*42 + pTau181/A*β*42 | 0.939 | 115 | 0.75 | 148 |

| Combination | A+/T+ in BBM+ | n BBM+ | non-A+/T+ in BBM- | n BBM- |
| --- | --- | --- | --- | --- |
| pTau217 + NfL + pTau217/A*β*40 + pTau181/A*β*42 | 0.939 | 115 | 0.747 | 150 |
| A*β*42/40 + pTau217 + pTau181 + NfL + pTau181/A*β*42 | 0.939 | 99 | 0.884 | 86 |
| pTau217 + pTau181 + pTau217/A*β*40 + pTau217/A*β*42 + pTau181/A*β*40 +  pTau181/A*β*42 | 0.939 | 132 | 0.75 | 208 |
| A*β*42/40 + pTau217 + pTau181 + NfL + pTau181/A*β*40 + pTau181/A*β*42 | 0.939 | 98 | 0.881 | 84 |
| pTau217 + pTau181 + NfL + pTau181/A*β*42 | 0.938 | 113 | 0.743 | 152 |
| NfL + pTau217/A*β*40 + pTau181/A*β*40 + pTau181/A*β*42 | 0.938 | 113 | 0.743 | 148 |
| A*β*42/40 + pTau217 + pTau181 + NfL + pTau217/A*β*42 + pTau181/A*β*40 +  pTau181/A*β*42 | 0.938 | 96 | 0.881 | 84 |
| A*β*42/40 + pTau181 + NfL + pTau217/A*β*42 + pTau181/A*β*40 + pTau181/A*β*42 | 0.938 | 96 | 0.871 | 85 |
| A*β*42/40 + pTau181 + NfL + pTau217/A*β*42 + pTau181/A*β*40 | 0.938 | 96 | 0.871 | 85 |
| A*β*42/40 + pTau181 + NfL + pTau217/A*β*42 + pTau181/A*β*42 | 0.938 | 97 | 0.874 | 87 |
| pTau217 + pTau181 + pTau217/A*β*40 | 0.938 | 144 | 0.748 | 222 |
| pTau181 + pTau217/A*β*40 | 0.938 | 145 | 0.741 | 224 |
| A*β*42/40 + pTau217 + pTau181 + NfL + pTau217/A*β*42 + pTau181/A*β*40 | 0.938 | 96 | 0.881 | 84 |
| A*β*42/40 + pTau217 + pTau181 + NfL + pTau217/A*β*42 + pTau181/A*β*42 | 0.938 | 97 | 0.884 | 86 |
| pTau217 + NfL + pTau217/A*β*40 + pTau181/A*β*40 + pTau181/A*β*42 | 0.938 | 112 | 0.743 | 148 |
| pTau217 + pTau181 + NfL + pTau181/A*β*40 + pTau181/A*β*42 | 0.938 | 112 | 0.74 | 150 |
| NfL + pTau217/A*β*40 + pTau217/A*β*42 + pTau181/A*β*40 + pTau181/A*β*42 | 0.938 | 112 | 0.747 | 146 |
| pTau181 + pTau217/A*β*40 + pTau217/A*β*42 | 0.937 | 143 | 0.747 | 217 |
| pTau217 + NfL + pTau217/A*β*40 + pTau217/A*β*42 + pTau181/A*β*40 +  pTau181/A*β*42 | 0.937 | 111 | 0.747 | 146 |
| pTau217 + pTau181 + pTau217/A*β*40 + pTau217/A*β*42 | 0.937 | 142 | 0.747 | 217 |
| pTau181 + pTau217/A*β*42 + pTau181/A*β*40 | 0.936 | 141 | 0.737 | 217 |
| pTau217 + pTau181 + pTau217/A*β*42 + pTau181/A*β*40 | 0.935 | 139 | 0.743 | 214 |
|  |  |  |  | |

| Combination | A+/T+ in BBM+ | n BBM+ | non-A+/T+ in BBM- | n BBM- |
| --- | --- | --- | --- | --- |
| pTau181 + pTau217/A*β*42 + pTau181/A*β*42 | 0.935 | 138 | 0.741 | 216 |
| pTau217 + pTau181 + NfL | 0.935 | 123 | 0.733 | 161 |
| A*β*42/40 + pTau181 + pTau217/A*β*40 | 0.934 | 122 | 0.858 | 120 |
| A*β*42/40 + pTau217 + pTau181 + pTau217/A*β*40 | 0.934 | 121 | 0.858 | 120 |
| A*β*42/40 + pTau181 + pTau217/A*β*40 + pTau181/A*β*42 | 0.934 | 121 | 0.863 | 117 |
| A*β*42/40 + pTau181 + pTau217/A*β*40 + pTau217/A*β*42 | 0.934 | 121 | 0.858 | 120 |
| pTau181 + pTau217/A*β*42 + pTau181/A*β*40 + pTau181/A*β*42 | 0.934 | 137 | 0.738 | 214 |
| pTau217 + pTau181 + pTau217/A*β*42 + pTau181/A*β*42 | 0.934 | 136 | 0.746 | 213 |
| A*β*42/40 + pTau181 + pTau217/A*β*40 + pTau181/A*β*40 | 0.933 | 120 | 0.861 | 115 |
| pTau217/A*β*40 + pTau181/A*β*40 | 0.933 | 149 | 0.723 | 231 |
| A*β*42/40 + pTau181 + pTau217/A*β*40 + pTau217/A*β*42 + pTau181/A*β*40 | 0.933 | 119 | 0.861 | 115 |
| A*β*42/40 + pTau217 + pTau181 + pTau217/A*β*40 + pTau217/A*β*42 | 0.933 | 120 | 0.858 | 120 |
| A*β*42/40 + pTau217 + pTau181 + pTau217/A*β*40 + pTau181/A*β*40 +  pTau181/A*β*42 | 0.933 | 119 | 0.861 | 115 |
| A*β*42/40 + pTau181 + pTau217/A*β*40 + pTau217/A*β*42 + pTau181/A*β*40 +  pTau181/A*β*42 | 0.933 | 119 | 0.861 | 115 |
| A*β*42/40 + pTau217 + pTau181 + pTau217/A*β*40 + pTau217/A*β*42 +  pTau181/A*β*42 | 0.933 | 119 | 0.863 | 117 |
| pTau217 + pTau181 + pTau217/A*β*42 + pTau181/A*β*40 + pTau181/A*β*42 | 0.933 | 135 | 0.744 | 211 |
| A*β*42/40 + pTau181 + pTau217/A*β*40 + pTau181/A*β*40 + pTau181/A*β*42 | 0.933 | 120 | 0.861 | 115 |
| A*β*42/40 + pTau181 + pTau217/A*β*40 + pTau217/A*β*42 + pTau181/A*β*42 | 0.933 | 120 | 0.863 | 117 |
| A*β*42/40 + pTau217 + pTau181 + pTau217/A*β*40 + pTau181/A*β*42 | 0.933 | 120 | 0.863 | 117 |
| A*β*42/40 + pTau217 + pTau181 + pTau217/A*β*40 + pTau181/A*β*40 | 0.933 | 119 | 0.861 | 115 |
| pTau217 + NfL + pTau217/A*β*42 + pTau181/A*β*40 | 0.932 | 117 | 0.733 | 150 |

| Combination | A+/T+ in BBM+ | n BBM+ | non-A+/T+ in BBM- | n BBM- |
| --- | --- | --- | --- | --- |
| A*β*42/40 + pTau217 + pTau181 + pTau217/A*β*40 + pTau217/A*β*42 + pTau181/A*β*40 + pTau181/A*β*42 | 0.932 | 118 | 0.861 | 115 |
| pTau217 + pTau181 + pTau217/A*β*42 | 0.932 | 146 | 0.741 | 220 |
| A*β*42/40 + pTau217 + pTau181 + pTau217/A*β*40 + pTau217/A*β*42 +  pTau181/A*β*40 | 0.932 | 118 | 0.861 | 115 |
| pTau181 + pTau217/A*β*42 | 0.932 | 148 | 0.735 | 223 |
| pTau217/A*β*40 + pTau181/A*β*42 | 0.932 | 147 | 0.726 | 226 |
| NfL + pTau217/A*β*42 + pTau181/A*β*40 | 0.932 | 118 | 0.728 | 151 |
| A*β*42/40 + NfL + pTau217/A*β*40 + pTau181/A*β*42 | 0.932 | 103 | 0.893 | 84 |
| pTau217/A*β*40 + pTau217/A*β*42 + pTau181/A*β*40 | 0.932 | 147 | 0.732 | 224 |
| pTau217/A*β*40 + pTau217/A*β*42 + pTau181/A*β*42 | 0.932 | 146 | 0.734 | 222 |
| pTau217 + pTau217/A*β*40 + pTau181/A*β*40 | 0.932 | 146 | 0.736 | 227 |
| NfL + pTau217/A*β*42 + pTau181/A*β*42 | 0.932 | 117 | 0.737 | 152 |
| pTau217 + pTau217/A*β*40 + pTau181/A*β*42 | 0.931 | 144 | 0.735 | 223 |
| A*β*42/40 + pTau217 + NfL + pTau217/A*β*40 + pTau181/A*β*42 | 0.931 | 102 | 0.893 | 84 |
| A*β*42/40 + pTau217 + NfL + pTau217/A*β*40 + pTau217/A*β*42 + pTau181/A*β*42 | 0.931 | 101 | 0.893 | 84 |
| pTau217 + pTau217/A*β*40 + pTau217/A*β*42 + pTau181/A*β*40 | 0.931 | 144 | 0.735 | 223 |
| A*β*42/40 + NfL + pTau217/A*β*40 + pTau217/A*β*42 + pTau181/A*β*42 | 0.931 | 102 | 0.893 | 84 |
| pTau217/A*β*40 + pTau181/A*β*40 + pTau181/A*β*42 | 0.931 | 144 | 0.726 | 223 |
| pTau217 + NfL + pTau217/A*β*42 + pTau181/A*β*42 | 0.931 | 116 | 0.742 | 151 |
| pTau217 + pTau181 + pTau181/A*β*40 | 0.93 | 143 | 0.74 | 219 |
| A*β*42/40 + NfL + pTau217/A*β*40 + pTau181/A*β*40 | 0.93 | 100 | 0.89 | 82 |
| pTau217/A*β*40 + pTau217/A*β*42 + pTau181/A*β*40 + pTau181/A*β*42 | 0.93 | 143 | 0.735 | 219 |
| A*β*42/40 + NfL + pTau217/A*β*40 + pTau181/A*β*40 + pTau181/A*β*42 | 0.93 | 100 | 0.89 | 82 |
| NfL + pTau217/A*β*42 + pTau181/A*β*40 + pTau181/A*β*42 | 0.93 | 114 | 0.733 | 150 |
| pTau217 + pTau217/A*β*40 + pTau217/A*β*42 + pTau181/A*β*42 | 0.93 | 143 | 0.738 | 221 |
|  |  |  |  | |

| Combination | A+/T+ in BBM+ | n BBM+ | non-A+/T+ in BBM- | n BBM- |
| --- | --- | --- | --- | --- |
| A*β*42/40 + pTau217 + NfL + pTau217/A*β*40 + pTau181/A*β*40 | 0.929 | 99 | 0.89 | 82 |
| A*β*42/40 + NfL + pTau217/A*β*40 + pTau217/A*β*42 + pTau181/A*β*40 | 0.929 | 99 | 0.89 | 82 |
| pTau217 + pTau217/A*β*40 + pTau181/A*β*40 + pTau181/A*β*42 | 0.929 | 141 | 0.736 | 220 |
| pTau217 + NfL + pTau217/A*β*40 + pTau217/A*β*42 | 0.929 | 126 | 0.73 | 159 |
| A*β*42/40 + pTau217 + NfL + pTau217/A*β*40 + pTau217/A*β*42 + pTau181/A*β*40 | 0.929 | 98 | 0.89 | 82 |
| A*β*42/40 + pTau217 + NfL + pTau217/A*β*40 + pTau217/A*β*42 + pTau181/A*β*40 +  pTau181/A*β*42 | 0.929 | 98 | 0.89 | 82 |
| A*β*42/40 + NfL + pTau217/A*β*40 + pTau217/A*β*42 + pTau181/A*β*40 +  pTau181/A*β*42 | 0.929 | 99 | 0.89 | 82 |
| A*β*42/40 + pTau181 + pTau217/A*β*42 | 0.929 | 126 | 0.833 | 126 |
| NfL + pTau217/A*β*40 + pTau217/A*β*42 | 0.929 | 127 | 0.73 | 159 |
| A*β*42/40 + pTau217 + pTau181 | 0.929 | 126 | 0.846 | 123 |
| pTau217 + pTau217/A*β*40 + pTau217/A*β*42 + pTau181/A*β*40 + pTau181/A*β*42 | 0.929 | 140 | 0.739 | 218 |
| A*β*42/40 + pTau217 + NfL + pTau217/A*β*40 + pTau181/A*β*40 + pTau181/A*β*42 | 0.929 | 99 | 0.89 | 82 |
| pTau217 + NfL + pTau217/A*β*42 + pTau181/A*β*40 + pTau181/A*β*42 | 0.929 | 113 | 0.738 | 149 |
| pTau217 + pTau181 + pTau181/A*β*40 + pTau181/A*β*42 | 0.928 | 138 | 0.738 | 214 |
| pTau217 + pTau181 + pTau181/A*β*42 | 0.928 | 139 | 0.741 | 216 |
| A*β*42/40 + pTau217 + pTau181 + pTau181/A*β*40 | 0.927 | 123 | 0.847 | 118 |
| pTau217 + pTau181 | 0.927 | 151 | 0.732 | 228 |
| A*β*42/40 + pTau181 + pTau217/A*β*42 + pTau181/A*β*42 | 0.927 | 124 | 0.837 | 123 |
| A*β*42/40 + pTau217 + pTau181 + pTau181/A*β*42 | 0.927 | 124 | 0.85 | 120 |
| A*β*42/40 + pTau181 + pTau217/A*β*42 + pTau181/A*β*40 | 0.927 | 123 | 0.835 | 121 |
| A*β*42/40 + pTau217 + pTau181 + pTau217/A*β*42 | 0.927 | 124 | 0.846 | 123 |
| A*β*42/40 + pTau217 + pTau181 + pTau181/A*β*40 + pTau181/A*β*42 | 0.927 | 123 | 0.847 | 118 |

| Combination | A+/T+ in BBM+ | n BBM+ | non-A+/T+ in BBM- | n BBM- |
| --- | --- | --- | --- | --- |
| A*β*42/40 + pTau181 + pTau217/A*β*42 + pTau181/A*β*40 + pTau181/A*β*42 | 0.927 | 123 | 0.835 | 121 |
| pTau217 + NfL + pTau181/A*β*40 | 0.926 | 121 | 0.729 | 155 |
| A*β*42/40 + pTau217 + pTau181 + pTau217/A*β*42 + pTau181/A*β*42 | 0.926 | 122 | 0.85 | 120 |
| A*β*42/40 + pTau217 + pTau181 + pTau217/A*β*42 + pTau181/A*β*40 | 0.926 | 121 | 0.847 | 118 |
| A*β*42/40 + pTau217 + pTau181 + pTau217/A*β*42 + pTau181/A*β*40 +  pTau181/A*β*42 | 0.926 | 121 | 0.847 | 118 |
| A*β*42/40 + pTau217 + NfL + pTau217/A*β*40 | 0.925 | 106 | 0.879 | 91 |
| A*β*42/40 + NfL + pTau217/A*β*40 | 0.925 | 107 | 0.879 | 91 |
| pTau217 + pTau217/A*β*42 + pTau181/A*β*40 | 0.925 | 147 | 0.73 | 226 |
| pTau217 + pTau217/A*β*42 + pTau181/A*β*42 | 0.925 | 147 | 0.732 | 224 |
| A*β*42/40 + NfL + pTau217/A*β*40 + pTau217/A*β*42 | 0.925 | 106 | 0.879 | 91 |
| A*β*42/40 + pTau217 + NfL + pTau217/A*β*40 + pTau217/A*β*42 | 0.924 | 105 | 0.879 | 91 |
| pTau217 + NfL + pTau181/A*β*42 | 0.924 | 119 | 0.734 | 154 |
| A*β*42/40 + pTau217 + NfL + pTau181/A*β*42 | 0.924 | 105 | 0.874 | 87 |
| NfL + pTau217/A*β*42 | 0.924 | 131 | 0.718 | 163 |
| A*β*42/40 + pTau217/A*β*40 + pTau181/A*β*42 | 0.924 | 131 | 0.839 | 124 |
| NfL + pTau217/A*β*40 | 0.924 | 131 | 0.719 | 167 |
| A*β*42/40 + pTau181 + NfL + pTau181/A*β*40 + pTau181/A*β*42 | 0.923 | 104 | 0.86 | 86 |
| pTau217 + pTau217/A*β*42 + pTau181/A*β*40 + pTau181/A*β*42 | 0.923 | 143 | 0.733 | 221 |
| A*β*42/40 + pTau217/A*β*40 + pTau217/A*β*42 + pTau181/A*β*42 | 0.923 | 130 | 0.839 | 124 |
| A*β*42/40 + NfL + pTau217/A*β*42 + pTau181/A*β*42 | 0.923 | 104 | 0.864 | 88 |
| pTau217 + NfL + pTau217/A*β*40 | 0.923 | 130 | 0.727 | 165 |
| A*β*42/40 + pTau181 + NfL + pTau181/A*β*40 | 0.923 | 104 | 0.86 | 86 |
| pTau217 + NfL + pTau217/A*β*42 | 0.923 | 130 | 0.722 | 162 |
| A*β*42/40 + pTau217/A*β*40 + pTau181/A*β*40 | 0.922 | 128 | 0.843 | 121 |
| pTau217/A*β*40 + pTau217/A*β*42 | 0.922 | 167 | 0.713 | 247 |
| A*β*42/40 + pTau217/A*β*40 + pTau181/A*β*40 + pTau181/A*β*42 | 0.922 | 128 | 0.843 | 121 |
| pTau217 + NfL + pTau181/A*β*40 + pTau181/A*β*42 | 0.922 | 116 | 0.73 | 152 |
| A*β*42/40 + pTau217 + NfL + pTau217/A*β*42 + pTau181/A*β*42 | 0.922 | 103 | 0.874 | 87 |
|  |  |  |  | |

| Combination | A+/T+ in BBM+ | n BBM+ | non-A+/T+ in BBM- | n BBM- |
| --- | --- | --- | --- | --- |
| A*β*42/40 + pTau217 + NfL + pTau181/A*β*40 + pTau181/A*β*42 | 0.922 | 102 | 0.871 | 85 |
| A*β*42/40 + pTau217 + NfL + pTau181/A*β*40 | 0.922 | 102 | 0.871 | 85 |
| A*β*42/40 + pTau217 + pTau217/A*β*40 + pTau181/A*β*42 | 0.922 | 128 | 0.846 | 123 |
| A*β*42/40 + pTau217/A*β*40 + pTau217/A*β*42 + pTau181/A*β*40 + pTau181/A*β*42 | 0.921 | 127 | 0.843 | 121 |
| pTau217/A*β*42 + pTau181/A*β*40 | 0.921 | 152 | 0.722 | 230 |
| A*β*42/40 + NfL + pTau217/A*β*42 + pTau181/A*β*40 | 0.921 | 101 | 0.86 | 86 |
| A*β*42/40 + pTau217/A*β*40 + pTau217/A*β*42 + pTau181/A*β*40 | 0.921 | 127 | 0.843 | 121 |
| pTau217 + pTau181/A*β*40 | 0.921 | 151 | 0.727 | 231 |
| A*β*42/40 + pTau217 + pTau217/A*β*40 + pTau217/A*β*42 + pTau181/A*β*42 | 0.921 | 127 | 0.846 | 123 |
| A*β*42/40 + NfL + pTau217/A*β*42 + pTau181/A*β*40 + pTau181/A*β*42 | 0.921 | 101 | 0.86 | 86 |
| A*β*42/40 + pTau217 + pTau217/A*β*40 + pTau181/A*β*40 | 0.92 | 125 | 0.85 | 120 |
| pTau217 + pTau217/A*β*40 + pTau217/A*β*42 | 0.92 | 163 | 0.717 | 244 |
| A*β*42/40 + pTau217 + NfL + pTau217/A*β*42 + pTau181/A*β*40 | 0.92 | 100 | 0.871 | 85 |
| A*β*42/40 + pTau217 + NfL + pTau217/A*β*42 + pTau181/A*β*40 + pTau181/A*β*42 | 0.92 | 100 | 0.871 | 85 |
| A*β*42/40 + pTau217 + pTau217/A*β*40 + pTau181/A*β*40 + pTau181/A*β*42 | 0.92 | 125 | 0.85 | 120 |
| pTau217 + pTau181/A*β*42 | 0.92 | 150 | 0.727 | 227 |
| A*β*42/40 + pTau217 + pTau217/A*β*40 + pTau217/A*β*42 + pTau181/A*β*40 | 0.919 | 124 | 0.85 | 120 |
| pTau217/A*β*42 + pTau181/A*β*40 + pTau181/A*β*42 | 0.919 | 148 | 0.724 | 225 |
| A*β*42/40 + pTau217 + NfL | 0.919 | 111 | 0.862 | 94 |
| A*β*42/40 + pTau217 + pTau217/A*β*40 + pTau217/A*β*42 + pTau181/A*β*40 +  pTau181/A*β*42 | 0.919 | 124 | 0.85 | 120 |
| pTau217 + pTau181/A*β*40 + pTau181/A*β*42 | 0.918 | 146 | 0.728 | 224 |
| A*β*42/40 + NfL + pTau217/A*β*42 | 0.918 | 110 | 0.853 | 95 |
| pTau217 + pTau217/A*β*40 | 0.917 | 168 | 0.716 | 250 |
| A*β*42/40 + pTau217 + pTau181/A*β*42 | 0.917 | 133 | 0.833 | 126 |
| pTau217 + pTau217/A*β*42 | 0.917 | 169 | 0.713 | 247 |
| A*β*42/40 + pTau217 + NfL + pTau217/A*β*42 | 0.917 | 109 | 0.862 | 94 |

| Combination | A+/T+ in BBM+ | n BBM+ | non-A+/T+ in BBM- | n BBM- |
| --- | --- | --- | --- | --- |
| A*β*42/40 + pTau181 + pTau181/A*β*40 + pTau181/A*β*42 | 0.917 | 133 | 0.815 | 124 |
| A*β*42/40 + pTau181 + pTau181/A*β*40 | 0.917 | 133 | 0.815 | 124 |
| A*β*42/40 + pTau217 + pTau217/A*β*42 + pTau181/A*β*42 | 0.916 | 131 | 0.833 | 126 |
| A*β*42/40 + pTau181 + NfL + pTau181/A*β*42 | 0.916 | 107 | 0.864 | 88 |
| A*β*42/40 + pTau217 + pTau181/A*β*40 | 0.915 | 129 | 0.837 | 123 |
| A*β*42/40 + pTau217 + pTau181/A*β*40 + pTau181/A*β*42 | 0.915 | 129 | 0.837 | 123 |
| A*β*42/40 + pTau217/A*β*40 | 0.915 | 141 | 0.836 | 134 |
| A*β*42/40 + pTau217/A*β*40 + pTau217/A*β*42 | 0.914 | 140 | 0.836 | 134 |
| pTau217/A*β*40 | 0.914 | 175 | 0.699 | 259 |
| pTau181 + NfL + pTau181/A*β*40 | 0.913 | 126 | 0.708 | 161 |
| A*β*42/40 + pTau217 + pTau217/A*β*42 + pTau181/A*β*40 | 0.913 | 127 | 0.837 | 123 |
| A*β*42/40 + pTau217 + pTau217/A*β*42 + pTau181/A*β*40 + pTau181/A*β*42 | 0.913 | 127 | 0.837 | 123 |
| A*β*42/40 + pTau217 + pTau217/A*β*40 | 0.912 | 137 | 0.841 | 132 |
| A*β*42/40 + pTau181 + pTau181/A*β*42 | 0.912 | 136 | 0.817 | 126 |
| A*β*42/40 + pTau217 + pTau217/A*β*40 + pTau217/A*β*42 | 0.912 | 136 | 0.841 | 132 |
| pTau217 + NfL | 0.912 | 137 | 0.708 | 171 |
| A*β*42/40 + pTau217 | 0.91 | 144 | 0.83 | 135 |
| pTau217 | 0.91 | 177 | 0.7 | 257 |
| pTau217/A*β*42 + pTau181/A*β*42 | 0.909 | 154 | 0.721 | 229 |
| A*β*42/40 + pTau217/A*β*42 + pTau181/A*β*40 + pTau181/A*β*42 | 0.909 | 132 | 0.819 | 127 |
| A*β*42/40 + pTau217/A*β*42 + pTau181/A*β*40 | 0.909 | 132 | 0.819 | 127 |
| A*β*42/40 + pTau217 + pTau217/A*β*42 | 0.908 | 142 | 0.83 | 135 |
| A*β*42/40 + NfL + pTau181/A*β*40 | 0.908 | 109 | 0.831 | 89 |
| A*β*42/40 + NfL + pTau181/A*β*40 + pTau181/A*β*42 | 0.908 | 109 | 0.831 | 89 |
| pTau181 + NfL + pTau181/A*β*40 + pTau181/A*β*42 | 0.908 | 120 | 0.713 | 157 |
| A*β*42/40 + pTau181 + NfL | 0.903 | 113 | 0.839 | 93 |
| A*β*42/40 + pTau181/A*β*40 + pTau181/A*β*42 | 0.902 | 143 | 0.784 | 134 |
| A*β*42/40 + pTau181 | 0.902 | 143 | 0.802 | 131 |
| A*β*42/40 + pTau181/A*β*40 | 0.902 | 143 | 0.784 | 134 |

| Combination | A+/T+ in BBM+ | n BBM+ | non-A+/T+ in BBM- | n BBM- |
| --- | --- | --- | --- | --- |
| pTau181 + NfL + pTau181/A*β*42 | 0.902 | 123 | 0.717 | 159 |
| NfL + pTau181/A*β*40 | 0.901 | 131 | 0.691 | 165 |
| pTau217/A*β*42 | 0.899 | 179 | 0.698 | 255 |
| A*β*42/40 + pTau217/A*β*42 + pTau181/A*β*42 | 0.899 | 138 | 0.809 | 131 |
| NfL + pTau181/A*β*40 + pTau181/A*β*42 | 0.896 | 125 | 0.696 | 161 |
| pTau181 + pTau181/A*β*40 | 0.894 | 160 | 0.696 | 237 |
| pTau181 + pTau181/A*β*40 + pTau181/A*β*42 | 0.89 | 154 | 0.703 | 229 |
| A*β*42/40 + pTau217/A*β*42 | 0.888 | 152 | 0.809 | 141 |
| A*β*42/40 + NfL + pTau181/A*β*42 | 0.888 | 116 | 0.835 | 91 |
| pTau181 + pTau181/A*β*42 | 0.885 | 157 | 0.706 | 231 |
| NfL + pTau181/A*β*42 | 0.879 | 132 | 0.699 | 163 |
| A*β*42/40 + pTau181/A*β*42 | 0.877 | 154 | 0.754 | 142 |
| pTau181 + NfL | 0.873 | 142 | 0.688 | 173 |
| pTau181/A*β*40 + pTau181/A*β*42 | 0.87 | 169 | 0.679 | 246 |
| pTau181 | 0.867 | 180 | 0.677 | 254 |
| pTau181/A*β*40 | 0.864 | 177 | 0.669 | 257 |
| pTau181/A*β*42 | 0.85 | 180 | 0.665 | 254 |
| A*β*42/40 + NfL | 0.763 | 156 | 0.802 | 101 |
| A*β*42/40 | 0.695 | 266 | 0.685 | 168 |
| NfL | 0.682 | 223 | 0.592 | 211 |

Supplementary table 12	Cutoff combinations — Agglomerative (Complete) (all 255 combinations, eight biomarkers). Prevalence of A^+^/T^+^ CSF profile in BBM-positive subgroups and prevalence of non-A^+^/T^+^ CSF profile in BBM-negative subgroups.

| Combination | A+/T+ in BBM+ | n BBM+ | non-A+/T+ in BBM- | n BBM- |
| --- | --- | --- | --- | --- |
| pTau181 + pTau217/A*β*42 | 0.978 | 90 | 0.643 | 269 |
| pTau217 + pTau181 + pTau217/A*β*42 | 0.977 | 88 | 0.655 | 264 |
| pTau181 + pTau217/A*β*40 + pTau217/A*β*42 | 0.977 | 87 | 0.646 | 268 |
| pTau217 + pTau181 + pTau217/A*β*40 + pTau217/A*β*42 | 0.977 | 86 | 0.655 | 264 |
| pTau181 + pTau217/A*β*40 + pTau181/A*β*40 | 0.976 | 85 | 0.658 | 263 |
| pTau217 + pTau181 + pTau181/A*β*40 | 0.976 | 85 | 0.665 | 260 |
| pTau181 + pTau217/A*β*40 + pTau181/A*β*42 | 0.976 | 82 | 0.663 | 255 |
| pTau181 + pTau217/A*β*42 + pTau181/A*β*40 | 0.975 | 81 | 0.653 | 262 |
| pTau181 + pTau217/A*β*42 + pTau181/A*β*42 | 0.975 | 80 | 0.66 | 256 |
| pTau181 + pTau217/A*β*40 + pTau217/A*β*42 + pTau181/A*β*40 | 0.975 | 79 | 0.655 | 261 |
| pTau181 + pTau217/A*β*42 + pTau181/A*β*40 + pTau181/A*β*42 | 0.975 | 79 | 0.663 | 255 |
| pTau217 + pTau181 + pTau181/A*β*40 + pTau181/A*β*42 | 0.975 | 79 | 0.673 | 251 |
| pTau217 + pTau181 + pTau217/A*β*42 + pTau181/A*β*40 | 0.975 | 79 | 0.665 | 257 |

| Combination | A+/T+ in BBM+ | n BBM+ | non-A+/T+ in BBM- | n BBM- |
| --- | --- | --- | --- | --- |
| pTau217 + pTau181 + pTau217/A*β*40 + pTau181/A*β*40 | 0.975 | 81 | 0.668 | 259 |
| pTau217 + pTau181 + pTau181/A*β*42 | 0.975 | 80 | 0.668 | 253 |
| pTau181 + pTau217/A*β*40 + pTau181/A*β*40 + pTau181/A*β*42 | 0.975 | 81 | 0.665 | 254 |
| pTau217 + pTau181 + pTau217/A*β*40 + pTau217/A*β*42 + pTau181/A*β*40 | 0.974 | 78 | 0.665 | 257 |
| pTau217 + pTau181 + pTau217/A*β*40 + pTau181/A*β*40 + pTau181/A*β*42 | 0.974 | 77 | 0.676 | 250 |
| pTau217 + pTau181 + pTau217/A*β*42 + pTau181/A*β*40 + pTau181/A*β*42 | 0.974 | 77 | 0.676 | 250 |
| pTau181 + pTau217/A*β*40 + pTau217/A*β*42 + pTau181/A*β*40 + pTau181/A*β*42 | 0.974 | 77 | 0.665 | 254 |
| pTau217 + pTau181 + pTau217/A*β*40 + pTau217/A*β*42 + pTau181/A*β*40 +  pTau181/A*β*42 | 0.974 | 76 | 0.676 | 250 |
| pTau217 + pTau181 + pTau217/A*β*40 + pTau181/A*β*42 | 0.974 | 78 | 0.673 | 251 |
| pTau181 + pTau217/A*β*40 + pTau217/A*β*42 + pTau181/A*β*42 | 0.974 | 78 | 0.663 | 255 |
| pTau217 + pTau181 + pTau217/A*β*42 + pTau181/A*β*42 | 0.974 | 78 | 0.673 | 251 |
| pTau217 + pTau181 + pTau217/A*β*40 + pTau217/A*β*42 + pTau181/A*β*42 | 0.974 | 77 | 0.673 | 251 |
| A*β*42/40 + pTau217 + pTau181 + pTau217/A*β*42 | 0.973 | 73 | 0.789 | 152 |
| A*β*42/40 + pTau181 + pTau217/A*β*40 | 0.973 | 74 | 0.784 | 153 |
| A*β*42/40 + pTau181 + pTau217/A*β*42 | 0.973 | 75 | 0.784 | 153 |
| A*β*42/40 + pTau217 + pTau181 + pTau217/A*β*40 | 0.972 | 71 | 0.789 | 152 |
| pTau181 + NfL + pTau217/A*β*42 | 0.972 | 72 | 0.667 | 198 |
| A*β*42/40 + pTau181 + pTau217/A*β*40 + pTau217/A*β*42 | 0.972 | 72 | 0.784 | 153 |
| A*β*42/40 + pTau217 + pTau181 + pTau217/A*β*40 + pTau217/A*β*42 | 0.972 | 71 | 0.789 | 152 |
| pTau217 + pTau181 + NfL + pTau217/A*β*42 | 0.972 | 71 | 0.677 | 195 |
| NfL + pTau217/A*β*40 + pTau181/A*β*40 | 0.971 | 69 | 0.686 | 194 |
| A*β*42/40 + pTau181 + pTau217/A*β*42 + pTau181/A*β*42 | 0.971 | 68 | 0.787 | 150 |
| pTau217 + NfL + pTau181/A*β*40 | 0.971 | 69 | 0.691 | 191 |
| A*β*42/40 + pTau181 + pTau217/A*β*42 + pTau181/A*β*40 | 0.971 | 68 | 0.792 | 149 |
|  |  |  |  | |

| Combination | | A+/T+ in BBM+ | | n BBM+ | | non-A+/T+ in BBM- | | n BBM- | |
| --- | --- | --- | --- | --- | --- | --- | --- | --- | --- |
| A*β*42/40 + pTau181 + pTau217/A*β*40 + pTau181/A*β*40 | | 0.971 | | 68 | | 0.792 | | 149 | |
| A*β*42/40 + pTau181 + pTau217/A*β*40 + pTau181/A*β*42 | | 0.971 | | 68 | | 0.787 | | 150 | |
| pTau217 + pTau181 + NfL + pTau217/A*β*40 + pTau217/A*β*42 | | 0.971 | | 69 | | 0.677 | | 195 | |
| pTau181 + NfL + pTau217/A*β*40 + pTau217/A*β*42 | | 0.971 | | 70 | | 0.67 | | 197 | |
| pTau217 + NfL + pTau217/A*β*40 + pTau181/A*β*40 | | 0.97 | | 66 | | 0.691 | | 191 | |
| A*β*42/40 + pTau181 + pTau217/A*β*40 + pTau181/A*β*40 + pTau181/A*β*42 | | 0.97 | | 67 | | 0.792 | | 149 | |
| pTau217 + pTau181 + NfL + pTau181/A*β*40 | | 0.97 | | 67 | | 0.691 | | 191 | |
| pTau181 + NfL + pTau217/A*β*40 + pTau181/A*β*40 | | 0.97 | | 67 | | 0.684 | | 193 | |
| A*β*42/40 + pTau217 + pTau181 + pTau217/A*β*42 + pTau181/A*β*40 | | 0.97 | | 66 | | 0.797 | | 148 | |
| A*β*42/40 + pTau181 + pTau217/A*β*42 + pTau181/A*β*40 + pTau181/A*β*42 | | 0.97 | | 67 | | 0.792 | | 149 | |
| A*β*42/40 + pTau217 + pTau181 + pTau181/A*β*42 | | 0.97 | | 66 | | 0.787 | | 150 | |
| A*β*42/40 + pTau181 + pTau217/A*β*40 + pTau217/A*β*42 + pTau181/A*β*42 | | 0.97 | | 66 | | 0.787 | | 150 | |
| NfL + pTau217/A*β*42 + pTau181/A*β*40 | | 0.97 | | 66 | | 0.679 | | 193 | |
| A*β*42/40 + pTau217 + pTau181 + pTau217/A*β*42 + pTau181/A*β*42 | | 0.97 | | 66 | | 0.792 | | 149 | |
| A*β*42/40 + pTau181 + pTau217/A*β*40 + pTau217/A*β*42 + pTau181/A*β*40 | | 0.97 | | 66 | | 0.792 | | 149 | |
| A*β*42/40 + pTau217 + pTau181 + pTau181/A*β*40 | | 0.97 | | 67 | | 0.797 | | 148 | |
| pTau181 + NfL + pTau217/A*β*40 + pTau181/A*β*42 | | 0.969 | | 64 | | 0.694 | | 186 | |
| NfL + pTau217/A*β*40 + pTau217/A*β*42 + pTau181/A*β*40 | | 0.969 | | 65 | | 0.682 | | 192 | |
| A*β*42/40 + pTau217 + pTau181 + pTau181/A*β*40 + pTau181/A*β*42 | | 0.969 | | 65 | | 0.797 | | 148 | |
| NfL + pTau217/A*β*42 + pTau181/A*β*42 | | 0.969 | | 64 | | 0.684 | | 190 | |
| NfL + pTau217/A*β*40 + pTau181/A*β*42 | | 0.969 | | 65 | | 0.688 | | 189 | |
| pTau217 + NfL + pTau181/A*β*42 | | 0.969 | | 64 | | 0.694 | | 186 | |
| A*β*42/40 + pTau217 + pTau181 + pTau217/A*β*40 + pTau217/A*β*42 +  pTau181/A*β*42 | | 0.969 | | 65 | | 0.792 | | 149 | |
|  | |  | |  | |  | | | |
| Combination | A+/T+ in BBM+ | | n BBM+ | | non-A+/T+ in BBM- | | n BBM- | |  |
| A*β*42/40 + pTau217 + pTau181 + pTau217/A*β*40 + pTau181/A*β*40 +  pTau181/A*β*42 | 0.969 | | 64 | | 0.797 | | 148 | |  |
| A*β*42/40 + pTau217 + pTau181 + pTau217/A*β*40 + pTau217/A*β*42 +  pTau181/A*β*40 | 0.969 | | 65 | | 0.797 | | 148 | |  |
| pTau217 + pTau181 + NfL + pTau217/A*β*40 + pTau181/A*β*40 | 0.969 | | 64 | | 0.691 | | 191 | |  |
| pTau217 + NfL + pTau217/A*β*40 + pTau217/A*β*42 + pTau181/A*β*40 | 0.969 | | 64 | | 0.688 | | 189 | |  |
| A*β*42/40 + pTau217 + pTau181 + pTau217/A*β*40 + pTau217/A*β*42 + pTau181/A*β*40 + pTau181/A*β*42 | 0.969 | | 64 | | 0.797 | | 148 | |  |
| A*β*42/40 + pTau217 + pTau181 + pTau217/A*β*42 + pTau181/A*β*40 +  pTau181/A*β*42 | 0.969 | | 65 | | 0.797 | | 148 | |  |
| A*β*42/40 + pTau217 + pTau181 + pTau217/A*β*40 + pTau181/A*β*42 | 0.969 | | 65 | | 0.792 | | 149 | |  |
| pTau217 + NfL + pTau217/A*β*42 + pTau181/A*β*40 | 0.969 | | 65 | | 0.688 | | 189 | |  |
| pTau181 + NfL + pTau217/A*β*42 + pTau181/A*β*40 | 0.969 | | 64 | | 0.677 | | 192 | |  |
| A*β*42/40 + pTau217 + pTau181 + pTau217/A*β*40 + pTau181/A*β*40 | 0.969 | | 65 | | 0.797 | | 148 | |  |
| NfL + pTau217/A*β*40 + pTau181/A*β*40 + pTau181/A*β*42 | 0.969 | | 64 | | 0.699 | | 186 | |  |
| A*β*42/40 + pTau181 + pTau217/A*β*40 + pTau217/A*β*42 + pTau181/A*β*40 +  pTau181/A*β*42 | 0.969 | | 65 | | 0.792 | | 149 | |  |
| NfL + pTau217/A*β*40 + pTau217/A*β*42 + pTau181/A*β*40 + pTau181/A*β*42 | 0.968 | | 62 | | 0.699 | | 186 | |  |
| pTau217 + NfL + pTau217/A*β*42 + pTau181/A*β*42 | 0.968 | | 63 | | 0.697 | | 185 | |  |
| pTau181 + NfL + pTau217/A*β*42 + pTau181/A*β*42 | 0.968 | | 63 | | 0.69 | | 187 | |  |
| NfL + pTau217/A*β*40 + pTau217/A*β*42 + pTau181/A*β*42 | 0.968 | | 63 | | 0.688 | | 189 | |  |
| NfL + pTau217/A*β*42 + pTau181/A*β*40 + pTau181/A*β*42 | 0.968 | | 63 | | 0.695 | | 187 | |  |
| pTau217 + NfL + pTau217/A*β*40 + pTau181/A*β*42 | 0.968 | | 62 | | 0.697 | | 185 | |  |

| Combination | A+/T+ in BBM+ | n BBM+ | non-A+/T+ in BBM- | n BBM- |
| --- | --- | --- | --- | --- |
| pTau217 + NfL + pTau181/A*β*40 + pTau181/A*β*42 | 0.968 | 63 | 0.705 | 183 |
| pTau217 + pTau181 + NfL + pTau217/A*β*40 + pTau217/A*β*42 + pTau181/A*β*40 | 0.968 | 62 | 0.688 | 189 |
| pTau181 + NfL + pTau217/A*β*40 + pTau217/A*β*42 + pTau181/A*β*40 | 0.968 | 63 | 0.681 | 191 |
| pTau181 + NfL + pTau217/A*β*42 + pTau181/A*β*40 + pTau181/A*β*42 | 0.968 | 62 | 0.694 | 186 |
| pTau217 + pTau181 + NfL + pTau181/A*β*42 | 0.968 | 63 | 0.697 | 185 |
| pTau217 + NfL + pTau217/A*β*40 + pTau217/A*β*42 + pTau181/A*β*42 | 0.968 | 62 | 0.697 | 185 |
| pTau217 + NfL + pTau217/A*β*42 + pTau181/A*β*40 + pTau181/A*β*42 | 0.968 | 62 | 0.705 | 183 |
| pTau181 + NfL + pTau217/A*β*40 + pTau217/A*β*42 + pTau181/A*β*42 | 0.968 | 62 | 0.694 | 186 |
| pTau217 + pTau181 + NfL + pTau217/A*β*42 + pTau181/A*β*42 | 0.968 | 62 | 0.701 | 184 |
| pTau217/A*β*40 + pTau181/A*β*40 | 0.968 | 95 | 0.647 | 278 |
| pTau217 + pTau181 + NfL + pTau181/A*β*40 + pTau181/A*β*42 | 0.968 | 62 | 0.705 | 183 |
| pTau217 + pTau181 + NfL + pTau217/A*β*42 + pTau181/A*β*40 | 0.968 | 63 | 0.688 | 189 |
| pTau181 + NfL + pTau217/A*β*40 + pTau181/A*β*40 + pTau181/A*β*42 | 0.968 | 63 | 0.697 | 185 |
| A*β*42/40 + pTau217 + pTau181 + NfL | 0.968 | 63 | 0.812 | 117 |
| pTau217 + pTau181/A*β*40 | 0.968 | 94 | 0.665 | 269 |
| pTau181 + pTau217/A*β*40 | 0.968 | 95 | 0.648 | 270 |
| pTau217 + NfL + pTau217/A*β*40 + pTau181/A*β*40 + pTau181/A*β*42 | 0.967 | 61 | 0.705 | 183 |
| pTau217/A*β*42 + pTau181/A*β*40 | 0.967 | 91 | 0.644 | 278 |
| pTau217 + pTau217/A*β*40 + pTau181/A*β*40 | 0.967 | 90 | 0.668 | 268 |
| pTau217 + pTau181 + pTau217/A*β*40 | 0.967 | 91 | 0.658 | 266 |
| pTau217 + pTau181 + NfL + pTau217/A*β*40 + pTau181/A*β*42 | 0.967 | 61 | 0.701 | 184 |
| pTau217 + pTau181 + NfL + pTau217/A*β*40 + pTau217/A*β*42 + pTau181/A*β*42 | 0.967 | 61 | 0.701 | 184 |
| pTau217 + pTau181 + NfL + pTau217/A*β*42 + pTau181/A*β*40 + pTau181/A*β*42 | 0.967 | 61 | 0.705 | 183 |
| pTau217 + pTau181 + NfL + pTau217/A*β*40 + pTau217/A*β*42 + pTau181/A*β*40 +  pTau181/A*β*42 | 0.967 | 60 | 0.705 | 183 |

| Combination | A+/T+ in BBM+ | n BBM+ | non-A+/T+ in BBM- | n BBM- |
| --- | --- | --- | --- | --- |
| pTau217 + pTau181 + NfL + pTau217/A*β*40 + pTau181/A*β*40 + pTau181/A*β*42 | 0.967 | 60 | 0.705 | 183 |
| pTau217 + NfL + pTau217/A*β*40 + pTau217/A*β*42 + pTau181/A*β*40 +  pTau181/A*β*42 | 0.967 | 61 | 0.705 | 183 |
| pTau181 + NfL + pTau217/A*β*40 + pTau217/A*β*42 + pTau181/A*β*40 +  pTau181/A*β*42 | 0.967 | 61 | 0.697 | 185 |
| pTau217 + pTau181/A*β*42 | 0.966 | 87 | 0.667 | 264 |
| pTau217 + pTau217/A*β*42 + pTau181/A*β*40 | 0.966 | 88 | 0.665 | 266 |
| A*β*42/40 + pTau181 + NfL + pTau217/A*β*42 | 0.966 | 59 | 0.812 | 117 |
| pTau217/A*β*40 + pTau181/A*β*40 + pTau181/A*β*42 | 0.966 | 88 | 0.654 | 269 |
| pTau217/A*β*40 + pTau217/A*β*42 + pTau181/A*β*40 | 0.966 | 89 | 0.647 | 275 |
| A*β*42/40 + pTau181 + NfL + pTau217/A*β*40 | 0.966 | 58 | 0.812 | 117 |
| pTau217 + pTau217/A*β*40 + pTau217/A*β*42 + pTau181/A*β*40 | 0.966 | 87 | 0.665 | 266 |
| pTau217/A*β*40 + pTau217/A*β*42 | 0.966 | 116 | 0.622 | 304 |
| pTau217/A*β*40 + pTau181/A*β*42 | 0.966 | 89 | 0.646 | 274 |
| A*β*42/40 + pTau217 + pTau181 + NfL + pTau217/A*β*42 | 0.966 | 58 | 0.819 | 116 |
| pTau217/A*β*40 + pTau217/A*β*42 + pTau181/A*β*42 | 0.965 | 85 | 0.648 | 273 |
| pTau217 + pTau217/A*β*42 + pTau181/A*β*42 | 0.965 | 85 | 0.672 | 262 |
| A*β*42/40 + pTau181 + NfL + pTau217/A*β*40 + pTau217/A*β*42 | 0.965 | 57 | 0.812 | 117 |
| pTau217 + pTau217/A*β*40 + pTau181/A*β*42 | 0.965 | 85 | 0.672 | 262 |
| pTau217/A*β*42 + pTau181/A*β*40 + pTau181/A*β*42 | 0.965 | 86 | 0.653 | 271 |
| pTau217 + pTau181/A*β*40 + pTau181/A*β*42 | 0.965 | 86 | 0.673 | 260 |
| pTau217 + pTau217/A*β*40 + pTau217/A*β*42 | 0.965 | 113 | 0.644 | 292 |
| pTau217 + pTau217/A*β*42 | 0.965 | 115 | 0.637 | 295 |
| pTau217 + pTau217/A*β*40 + pTau217/A*β*42 + pTau181/A*β*40 + pTau181/A*β*42 | 0.964 | 83 | 0.676 | 259 |
| pTau217 + pTau217/A*β*40 + pTau181/A*β*40 + pTau181/A*β*42 | 0.964 | 84 | 0.676 | 259 |
| NfL + pTau217/A*β*42 | 0.964 | 84 | 0.649 | 211 |
| A*β*42/40 + pTau217 + pTau181 + NfL + pTau217/A*β*40 + pTau217/A*β*42 | 0.964 | 56 | 0.819 | 116 |
| pTau217 + pTau217/A*β*42 + pTau181/A*β*40 + pTau181/A*β*42 | 0.964 | 84 | 0.676 | 259 |
|  |  |  |  | |

| Combination | A+/T+ in BBM+ | n BBM+ | non-A+/T+ in BBM- | n BBM- |
| --- | --- | --- | --- | --- |
| pTau217/A*β*40 + pTau217/A*β*42 + pTau181/A*β*40 + pTau181/A*β*42 | 0.964 | 84 | 0.657 | 268 |
| A*β*42/40 + pTau217 + pTau181 + NfL + pTau217/A*β*40 | 0.964 | 56 | 0.819 | 116 |
| pTau217 + pTau217/A*β*40 + pTau217/A*β*42 + pTau181/A*β*42 | 0.964 | 84 | 0.672 | 262 |
| pTau217 + pTau181 + NfL | 0.963 | 81 | 0.677 | 198 |
| pTau217 + NfL + pTau217/A*β*42 | 0.963 | 82 | 0.663 | 205 |
| A*β*42/40 + NfL + pTau217/A*β*40 + pTau181/A*β*40 | 0.963 | 54 | 0.823 | 113 |
| A*β*42/40 + NfL + pTau217/A*β*40 + pTau181/A*β*42 | 0.963 | 54 | 0.802 | 116 |
| A*β*42/40 + pTau217 + NfL + pTau181/A*β*40 | 0.963 | 54 | 0.83 | 112 |
| A*β*42/40 + NfL + pTau217/A*β*42 + pTau181/A*β*42 | 0.963 | 54 | 0.802 | 116 |
| NfL + pTau217/A*β*40 + pTau217/A*β*42 | 0.963 | 82 | 0.659 | 208 |
| A*β*42/40 + NfL + pTau217/A*β*42 + pTau181/A*β*40 | 0.963 | 54 | 0.823 | 113 |
| A*β*42/40 + pTau217 + pTau181 | 0.962 | 79 | 0.784 | 153 |
| A*β*42/40 + pTau181 + NfL + pTau217/A*β*42 + pTau181/A*β*40 + pTau181/A*β*42 | 0.962 | 52 | 0.823 | 113 |
| A*β*42/40 + NfL + pTau217/A*β*40 + pTau217/A*β*42 + pTau181/A*β*40 +  pTau181/A*β*42 | 0.962 | 52 | 0.823 | 113 |
| A*β*42/40 + pTau217 + NfL + pTau181/A*β*42 | 0.962 | 53 | 0.809 | 115 |
| A*β*42/40 + pTau181 + NfL + pTau217/A*β*40 + pTau217/A*β*42 + pTau181/A*β*40 | 0.962 | 52 | 0.823 | 113 |
| pTau217 + NfL + pTau217/A*β*40 + pTau217/A*β*42 | 0.962 | 80 | 0.667 | 204 |
| A*β*42/40 + pTau217 + NfL + pTau217/A*β*40 + pTau181/A*β*42 | 0.962 | 52 | 0.816 | 114 |
| A*β*42/40 + pTau181 + NfL + pTau217/A*β*42 + pTau181/A*β*42 | 0.962 | 53 | 0.816 | 114 |
| A*β*42/40 + pTau217 + pTau181 + NfL + pTau181/A*β*40 | 0.962 | 53 | 0.83 | 112 |
| A*β*42/40 + pTau217 + NfL + pTau217/A*β*40 + pTau217/A*β*42 + pTau181/A*β*40 | 0.962 | 52 | 0.83 | 112 |
| A*β*42/40 + NfL + pTau217/A*β*40 + pTau181/A*β*40 + pTau181/A*β*42 | 0.962 | 53 | 0.823 | 113 |
| A*β*42/40 + pTau217 + pTau181 + NfL + pTau217/A*β*42 + pTau181/A*β*42 | 0.962 | 52 | 0.823 | 113 |

| Combination | A+/T+ in BBM+ | n BBM+ | non-A+/T+ in BBM- | n BBM- |
| --- | --- | --- | --- | --- |
| A*β*42/40 + pTau217 + NfL + pTau217/A*β*42 + pTau181/A*β*42 | 0.962 | 53 | 0.816 | 114 |
| A*β*42/40 + pTau217 + NfL + pTau181/A*β*40 + pTau181/A*β*42 | 0.962 | 52 | 0.83 | 112 |
| A*β*42/40 + pTau217 + NfL + pTau217/A*β*42 + pTau181/A*β*40 | 0.962 | 53 | 0.83 | 112 |
| A*β*42/40 + pTau181 + NfL + pTau217/A*β*40 + pTau181/A*β*40 | 0.962 | 53 | 0.823 | 113 |
| A*β*42/40 + pTau217 + pTau181 + NfL + pTau181/A*β*42 | 0.962 | 52 | 0.816 | 114 |
| A*β*42/40 + pTau217 + NfL + pTau217/A*β*40 + pTau181/A*β*40 | 0.962 | 52 | 0.83 | 112 |
| A*β*42/40 + pTau181 + NfL + pTau217/A*β*42 + pTau181/A*β*40 | 0.962 | 53 | 0.823 | 113 |
| A*β*42/40 + pTau181 + NfL + pTau217/A*β*40 + pTau181/A*β*42 | 0.962 | 53 | 0.816 | 114 |
| A*β*42/40 + NfL + pTau217/A*β*40 + pTau217/A*β*42 + pTau181/A*β*40 | 0.962 | 53 | 0.823 | 113 |
| A*β*42/40 + pTau181 + NfL + pTau217/A*β*40 + pTau217/A*β*42 + pTau181/A*β*42 | 0.962 | 52 | 0.816 | 114 |
| A*β*42/40 + pTau181 + NfL + pTau217/A*β*40 + pTau181/A*β*40 + pTau181/A*β*42 | 0.962 | 52 | 0.823 | 113 |
| A*β*42/40 + pTau217 + pTau181 + NfL + pTau217/A*β*42 + pTau181/A*β*40 | 0.962 | 52 | 0.83 | 112 |
| A*β*42/40 + NfL + pTau217/A*β*40 + pTau217/A*β*42 + pTau181/A*β*42 | 0.962 | 53 | 0.802 | 116 |
| A*β*42/40 + NfL + pTau217/A*β*42 + pTau181/A*β*40 + pTau181/A*β*42 | 0.962 | 53 | 0.823 | 113 |
| A*β*42/40 + pTau217 + NfL + pTau217/A*β*42 + pTau181/A*β*40 + pTau181/A*β*42 | 0.962 | 52 | 0.83 | 112 |
| A*β*42/40 + pTau217 + NfL + pTau217/A*β*40 + pTau217/A*β*42 + pTau181/A*β*42 | 0.962 | 52 | 0.816 | 114 |
| A*β*42/40 + pTau217 + NfL + pTau217/A*β*40 + pTau217/A*β*42 + pTau181/A*β*40 +  pTau181/A*β*42 | 0.961 | 51 | 0.83 | 112 |
| A*β*42/40 + pTau217 + pTau181 + NfL + pTau217/A*β*40 + pTau217/A*β*42 +  pTau181/A*β*40 | 0.961 | 51 | 0.83 | 112 |
| pTau181 + NfL + pTau217/A*β*40 | 0.961 | 76 | 0.673 | 199 |
| A*β*42/40 + pTau217 + pTau181 + NfL + pTau217/A*β*40 + pTau181/A*β*42 | 0.961 | 51 | 0.823 | 113 |

| Combination | A+/T+ in BBM+ | n BBM+ | non-A+/T+ in BBM- | n BBM- |
| --- | --- | --- | --- | --- |
| A*β*42/40 + pTau181 + NfL + pTau217/A*β*40 + pTau217/A*β*42 + pTau181/A*β*40 +  pTau181/A*β*42 | 0.961 | 51 | 0.823 | 113 |
| A*β*42/40 + pTau217 + pTau181 + NfL + pTau217/A*β*40 + pTau181/A*β*40 | 0.961 | 51 | 0.83 | 112 |
| A*β*42/40 + pTau217 + pTau181 + NfL + pTau181/A*β*40 + pTau181/A*β*42 | 0.961 | 51 | 0.83 | 112 |
| A*β*42/40 + pTau217 + NfL + pTau217/A*β*40 + pTau181/A*β*40 + pTau181/A*β*42 | 0.961 | 51 | 0.83 | 112 |
| A*β*42/40 + pTau217 + pTau181 + NfL + pTau217/A*β*42 + pTau181/A*β*40 +  pTau181/A*β*42 | 0.961 | 51 | 0.83 | 112 |
| A*β*42/40 + pTau217 + pTau181 + NfL + pTau217/A*β*40 + pTau217/A*β*42 +  pTau181/A*β*42 | 0.961 | 51 | 0.823 | 113 |
| pTau217 + pTau181 | 0.96 | 101 | 0.653 | 268 |
| A*β*42/40 + pTau217/A*β*40 + pTau181/A*β*42 | 0.96 | 75 | 0.766 | 158 |
| A*β*42/40 + pTau217 + pTau181 + NfL + pTau217/A*β*40 + pTau217/A*β*42 + pTau181/A*β*40 + pTau181/A*β*42 | 0.96 | 50 | 0.83 | 112 |
| A*β*42/40 + pTau217 + pTau181 + NfL + pTau217/A*β*40 + pTau181/A*β*40 +  pTau181/A*β*42 | 0.96 | 50 | 0.83 | 112 |
| A*β*42/40 + pTau217/A*β*40 + pTau181/A*β*40 | 0.96 | 75 | 0.779 | 154 |
| A*β*42/40 + pTau217/A*β*42 + pTau181/A*β*40 | 0.96 | 75 | 0.776 | 156 |
| pTau217/A*β*40 | 0.96 | 125 | 0.618 | 309 |
| A*β*42/40 + pTau217 + pTau181/A*β*40 | 0.959 | 74 | 0.784 | 153 |
| A*β*42/40 + pTau217/A*β*40 + pTau217/A*β*42 + pTau181/A*β*42 | 0.959 | 73 | 0.766 | 158 |
| A*β*42/40 + pTau217 + pTau217/A*β*42 + pTau181/A*β*40 | 0.959 | 73 | 0.784 | 153 |
| A*β*42/40 + pTau217/A*β*40 + pTau217/A*β*42 + pTau181/A*β*40 | 0.959 | 73 | 0.779 | 154 |
| A*β*42/40 + pTau217 + pTau181/A*β*42 | 0.959 | 73 | 0.771 | 157 |
| A*β*42/40 + pTau217 + pTau217/A*β*42 + pTau181/A*β*42 | 0.959 | 73 | 0.776 | 156 |
| A*β*42/40 + pTau217/A*β*42 + pTau181/A*β*40 + pTau181/A*β*42 | 0.959 | 74 | 0.776 | 156 |
| pTau217 + pTau181 + NfL + pTau217/A*β*40 | 0.959 | 73 | 0.68 | 197 |
| A*β*42/40 + pTau217/A*β*40 + pTau181/A*β*40 + pTau181/A*β*42 | 0.959 | 74 | 0.779 | 154 |

| Combination | A+/T+ in BBM+ | n BBM+ | non-A+/T+ in BBM- | n BBM- |
| --- | --- | --- | --- | --- |
| A*β*42/40 + pTau217 + pTau217/A*β*40 + pTau217/A*β*42 + pTau181/A*β*40 | 0.958 | 72 | 0.784 | 153 |
| A*β*42/40 + pTau217 + pTau217/A*β*40 + pTau181/A*β*42 | 0.958 | 72 | 0.776 | 156 |
| A*β*42/40 + pTau217 + pTau217/A*β*40 + pTau181/A*β*40 | 0.958 | 72 | 0.784 | 153 |
| A*β*42/40 + pTau217 + pTau181/A*β*40 + pTau181/A*β*42 | 0.958 | 72 | 0.784 | 153 |
| pTau217 + pTau217/A*β*40 | 0.958 | 119 | 0.644 | 295 |
| A*β*42/40 + pTau217 + pTau217/A*β*40 + pTau217/A*β*42 + pTau181/A*β*42 | 0.958 | 72 | 0.776 | 156 |
| A*β*42/40 + pTau217 + pTau217/A*β*40 + pTau217/A*β*42 + pTau181/A*β*40 +  pTau181/A*β*42 | 0.958 | 71 | 0.784 | 153 |
| A*β*42/40 + pTau217 + pTau217/A*β*42 + pTau181/A*β*40 + pTau181/A*β*42 | 0.958 | 72 | 0.784 | 153 |
| A*β*42/40 + pTau217/A*β*40 + pTau217/A*β*42 + pTau181/A*β*40 + pTau181/A*β*42 | 0.958 | 72 | 0.779 | 154 |
| A*β*42/40 + pTau217 + pTau217/A*β*40 + pTau181/A*β*40 + pTau181/A*β*42 | 0.958 | 71 | 0.784 | 153 |
| A*β*42/40 + pTau217/A*β*40 | 0.957 | 93 | 0.765 | 166 |
| A*β*42/40 + pTau217 + NfL | 0.957 | 70 | 0.81 | 121 |
| pTau217 + NfL | 0.957 | 94 | 0.66 | 209 |
| A*β*42/40 + pTau217 + pTau217/A*β*42 | 0.956 | 90 | 0.774 | 164 |
| A*β*42/40 + pTau217/A*β*40 + pTau217/A*β*42 | 0.956 | 90 | 0.765 | 166 |
| pTau217 | 0.955 | 133 | 0.631 | 301 |
| A*β*42/40 + pTau217 + pTau217/A*β*40 | 0.955 | 89 | 0.779 | 163 |
| A*β*42/40 + pTau217 + pTau217/A*β*40 + pTau217/A*β*42 | 0.955 | 88 | 0.779 | 163 |
| NfL + pTau217/A*β*40 | 0.955 | 88 | 0.659 | 211 |
| A*β*42/40 + NfL + pTau217/A*β*42 | 0.954 | 65 | 0.797 | 123 |
| A*β*42/40 + NfL + pTau217/A*β*40 | 0.953 | 64 | 0.803 | 122 |
| pTau217 + NfL + pTau217/A*β*40 | 0.952 | 84 | 0.667 | 207 |
| A*β*42/40 + NfL + pTau217/A*β*40 + pTau217/A*β*42 | 0.952 | 63 | 0.803 | 122 |
| A*β*42/40 + pTau217 + NfL + pTau217/A*β*42 | 0.952 | 63 | 0.817 | 120 |
| A*β*42/40 + pTau217 + NfL + pTau217/A*β*40 | 0.951 | 61 | 0.817 | 120 |
| A*β*42/40 + pTau217 + NfL + pTau217/A*β*40 + pTau217/A*β*42 | 0.951 | 61 | 0.817 | 120 |
| A*β*42/40 + pTau217 | 0.95 | 100 | 0.77 | 165 |
| pTau217/A*β*42 | 0.95 | 121 | 0.607 | 313 |

| Combination | A+/T+ in BBM+ | n BBM+ | non-A+/T+ in BBM- | n BBM- |
| --- | --- | --- | --- | --- |
| pTau217/A*β*42 + pTau181/A*β*42 | 0.944 | 89 | 0.64 | 278 |
| A*β*42/40 + pTau217/A*β*42 | 0.937 | 95 | 0.744 | 172 |
| A*β*42/40 + pTau217/A*β*42 + pTau181/A*β*42 | 0.935 | 77 | 0.753 | 162 |
| A*β*42/40 + pTau181 + pTau181/A*β*40 | 0.929 | 84 | 0.752 | 157 |
| A*β*42/40 + pTau181 + pTau181/A*β*42 | 0.928 | 83 | 0.733 | 161 |
| A*β*42/40 + pTau181 + pTau181/A*β*40 + pTau181/A*β*42 | 0.927 | 82 | 0.752 | 157 |
| A*β*42/40 + pTau181/A*β*40 | 0.926 | 94 | 0.729 | 166 |
| A*β*42/40 + pTau181/A*β*40 + pTau181/A*β*42 | 0.924 | 92 | 0.729 | 166 |
| A*β*42/40 + NfL + pTau181/A*β*40 | 0.923 | 65 | 0.802 | 116 |
| A*β*42/40 + pTau181 + NfL + pTau181/A*β*40 | 0.921 | 63 | 0.802 | 116 |
| NfL + pTau181/A*β*40 + pTau181/A*β*42 | 0.921 | 76 | 0.66 | 197 |
| A*β*42/40 + NfL + pTau181/A*β*40 + pTau181/A*β*42 | 0.921 | 63 | 0.802 | 116 |
| pTau181 + NfL + pTau181/A*β*42 | 0.92 | 75 | 0.652 | 198 |
| A*β*42/40 + pTau181 + NfL + pTau181/A*β*42 | 0.919 | 62 | 0.782 | 119 |
| pTau181 + NfL + pTau181/A*β*40 + pTau181/A*β*42 | 0.919 | 74 | 0.662 | 195 |
| A*β*42/40 + pTau181 + NfL + pTau181/A*β*40 + pTau181/A*β*42 | 0.918 | 61 | 0.802 | 116 |
| NfL + pTau181/A*β*40 | 0.916 | 83 | 0.649 | 205 |
| pTau181 + NfL + pTau181/A*β*40 | 0.912 | 80 | 0.65 | 203 |
| NfL + pTau181/A*β*42 | 0.91 | 78 | 0.644 | 202 |
| A*β*42/40 + NfL + pTau181/A*β*42 | 0.908 | 65 | 0.769 | 121 |
| pTau181 + pTau181/A*β*42 | 0.902 | 102 | 0.612 | 278 |
| pTau181 + pTau181/A*β*40 + pTau181/A*β*42 | 0.901 | 101 | 0.62 | 274 |
| pTau181 + pTau181/A*β*40 | 0.899 | 109 | 0.615 | 283 |
| A*β*42/40 + pTau181/A*β*42 | 0.892 | 102 | 0.693 | 176 |
| A*β*42/40 + pTau181 | 0.89 | 100 | 0.732 | 164 |
| pTau181/A*β*40 | 0.889 | 126 | 0.591 | 308 |
| pTau181/A*β*40 + pTau181/A*β*42 | 0.886 | 114 | 0.597 | 298 |
| A*β*42/40 + pTau181 + NfL | 0.883 | 77 | 0.779 | 122 |
| pTau181 + NfL | 0.864 | 103 | 0.622 | 217 |
| pTau181/A*β*42 | 0.863 | 124 | 0.577 | 310 |
| pTau181 | 0.858 | 134 | 0.59 | 300 |
| A*β*42/40 + NfL | 0.78 | 118 | 0.754 | 130 |
| A*β*42/40 | 0.712 | 236 | 0.646 | 198 |
| NfL | 0.699 | 186 | 0.565 | 248 |

Supplementary table 13 Cutoff combinations — Spectral (all 255 combinations, eight biomarkers). Prevalence of A^+^/T^+^ CSF profile in BBM-positive subgroups and prevalence of non-A^+^/T^+^ CSF profile in BBM-negative subgroups.

| Combination | A+/T+ in BBM+ | n BBM+ | non-A+/T+ in BBM- | n BBM- |
| --- | --- | --- | --- | --- |
| pTau181 + NfL + pTau217/A*β*40 + pTau181/A*β*40 | 0.954 | 109 | 0.762 | 151 |
| pTau217 + pTau181 + NfL + pTau217/A*β*40 + pTau181/A*β*40 | 0.954 | 109 | 0.762 | 151 |
| pTau217 + pTau181 + NfL + pTau217/A*β*40 + pTau181/A*β*42 | 0.953 | 106 | 0.767 | 150 |
| pTau181 + NfL + pTau217/A*β*40 + pTau181/A*β*42 | 0.953 | 106 | 0.767 | 150 |
| pTau217 + pTau181 + NfL + pTau217/A*β*40 + pTau217/A*β*42 + pTau181/A*β*40 | 0.953 | 107 | 0.767 | 146 |
| pTau181 + NfL + pTau217/A*β*40 + pTau217/A*β*42 + pTau181/A*β*40 | 0.953 | 107 | 0.767 | 146 |
| pTau217 + pTau181 + NfL + pTau217/A*β*40 + pTau217/A*β*42 + pTau181/A*β*42 | 0.952 | 105 | 0.77 | 148 |
| pTau181 + NfL + pTau217/A*β*40 + pTau181/A*β*40 + pTau181/A*β*42 | 0.952 | 105 | 0.774 | 146 |
| pTau217 + pTau181 + NfL + pTau217/A*β*40 + pTau217/A*β*42 + pTau181/A*β*40 +  pTau181/A*β*42 | 0.952 | 104 | 0.778 | 144 |
| pTau181 + NfL + pTau217/A*β*40 + pTau217/A*β*42 + pTau181/A*β*40 +  pTau181/A*β*42 | 0.952 | 104 | 0.778 | 144 |
| pTau181 + NfL + pTau217/A*β*40 + pTau217/A*β*42 + pTau181/A*β*42 | 0.952 | 105 | 0.77 | 148 |
| pTau217 + pTau181 + NfL + pTau217/A*β*40 + pTau181/A*β*40 + pTau181/A*β*42 | 0.952 | 105 | 0.774 | 146 |
| pTau181 + NfL + pTau217/A*β*40 | 0.947 | 113 | 0.75 | 160 |
| pTau217 + pTau181 + NfL + pTau217/A*β*40 | 0.947 | 113 | 0.75 | 160 |
| pTau217 + pTau181 + NfL + pTau217/A*β*40 + pTau217/A*β*42 | 0.946 | 111 | 0.753 | 154 |
| pTau181 + NfL + pTau217/A*β*40 + pTau217/A*β*42 | 0.946 | 111 | 0.753 | 154 |
| pTau181 + NfL + pTau217/A*β*42 + pTau181/A*β*40 | 0.945 | 109 | 0.755 | 151 |
| pTau217 + pTau181 + NfL + pTau217/A*β*42 + pTau181/A*β*40 | 0.945 | 109 | 0.758 | 149 |
| pTau181 + NfL + pTau217/A*β*42 + pTau181/A*β*42 | 0.944 | 107 | 0.758 | 153 |

| Combination | A+/T+ in BBM+ | n BBM+ | non-A+/T+ in BBM- | n BBM- |
| --- | --- | --- | --- | --- |
| pTau217 + pTau181 + NfL + pTau217/A*β*42 + pTau181/A*β*42 | 0.944 | 107 | 0.762 | 151 |
| pTau181 + NfL + pTau217/A*β*42 + pTau181/A*β*40 + pTau181/A*β*42 | 0.943 | 106 | 0.765 | 149 |
| pTau217 + pTau181 + NfL + pTau217/A*β*42 + pTau181/A*β*40 + pTau181/A*β*42 | 0.943 | 106 | 0.769 | 147 |
| pTau181 + pTau217/A*β*40 + pTau181/A*β*40 | 0.942 | 138 | 0.748 | 214 |
| pTau217 + pTau181 + pTau217/A*β*40 + pTau181/A*β*40 | 0.942 | 138 | 0.748 | 214 |
| pTau217 + pTau181 + pTau217/A*β*40 + pTau217/A*β*42 + pTau181/A*β*40 | 0.941 | 136 | 0.755 | 208 |
| pTau181 + pTau217/A*β*40 + pTau217/A*β*42 + pTau181/A*β*40 | 0.941 | 136 | 0.755 | 208 |
| pTau181 + pTau217/A*β*40 + pTau181/A*β*42 | 0.94 | 134 | 0.75 | 212 |
| NfL + pTau217/A*β*40 + pTau181/A*β*40 | 0.94 | 116 | 0.752 | 153 |
| pTau217 + pTau181 + pTau217/A*β*40 + pTau181/A*β*42 | 0.94 | 134 | 0.75 | 212 |
| pTau181 + pTau217/A*β*40 + pTau181/A*β*40 + pTau181/A*β*42 | 0.94 | 133 | 0.755 | 208 |
| pTau181 + pTau217/A*β*40 + pTau217/A*β*42 + pTau181/A*β*42 | 0.94 | 133 | 0.756 | 209 |
| pTau217 + pTau181 + pTau217/A*β*40 + pTau217/A*β*42 + pTau181/A*β*42 | 0.94 | 133 | 0.756 | 209 |
| pTau217 + pTau181 + pTau217/A*β*40 + pTau181/A*β*40 + pTau181/A*β*42 | 0.94 | 133 | 0.755 | 208 |
| pTau181 + pTau217/A*β*40 + pTau217/A*β*42 + pTau181/A*β*40 + pTau181/A*β*42 | 0.939 | 132 | 0.761 | 205 |
| NfL + pTau217/A*β*40 + pTau217/A*β*42 + pTau181/A*β*40 | 0.939 | 114 | 0.757 | 148 |
| pTau217 + NfL + pTau217/A*β*40 + pTau181/A*β*40 | 0.939 | 115 | 0.752 | 153 |
| pTau217 + pTau181 + pTau217/A*β*40 + pTau217/A*β*42 + pTau181/A*β*40 +  pTau181/A*β*42 | 0.939 | 132 | 0.761 | 205 |
| pTau217 + pTau181 + pTau217/A*β*40 | 0.938 | 144 | 0.741 | 224 |
| pTau181 + pTau217/A*β*40 | 0.938 | 144 | 0.741 | 224 |
| pTau181 + NfL + pTau217/A*β*42 | 0.938 | 113 | 0.742 | 159 |
| pTau217 + NfL + pTau217/A*β*40 + pTau181/A*β*42 | 0.938 | 112 | 0.757 | 152 |
| pTau217 + pTau181 + NfL + pTau217/A*β*42 | 0.938 | 113 | 0.745 | 157 |
| NfL + pTau217/A*β*40 + pTau217/A*β*42 + pTau181/A*β*42 | 0.938 | 112 | 0.76 | 150 |
|  |  |  |  | |

| Combination | A+/T+ in BBM+ | n BBM+ | non-A+/T+ in BBM- | n BBM- |
| --- | --- | --- | --- | --- |
| pTau217 + NfL + pTau217/A*β*40 + pTau217/A*β*42 + pTau181/A*β*40 | 0.938 | 113 | 0.757 | 148 |
| NfL + pTau217/A*β*40 + pTau181/A*β*42 | 0.938 | 113 | 0.757 | 152 |
| pTau217 + pTau181 + NfL + pTau181/A*β*40 | 0.938 | 113 | 0.748 | 155 |
| pTau181 + pTau217/A*β*40 + pTau217/A*β*42 | 0.937 | 142 | 0.747 | 217 |
| pTau217 + pTau181 + pTau217/A*β*40 + pTau217/A*β*42 | 0.937 | 142 | 0.747 | 217 |
| NfL + pTau217/A*β*40 + pTau181/A*β*40 + pTau181/A*β*42 | 0.937 | 111 | 0.764 | 148 |
| pTau217 + NfL + pTau217/A*β*40 + pTau217/A*β*42 + pTau181/A*β*42 | 0.937 | 111 | 0.76 | 150 |
| pTau217 + NfL + pTau217/A*β*40 + pTau217/A*β*42 + pTau181/A*β*40 +  pTau181/A*β*42 | 0.936 | 109 | 0.767 | 146 |
| pTau217 + pTau181 + NfL + pTau181/A*β*42 | 0.936 | 110 | 0.753 | 154 |
| pTau217 + pTau181 + NfL + pTau181/A*β*40 + pTau181/A*β*42 | 0.936 | 109 | 0.76 | 150 |
| NfL + pTau217/A*β*40 + pTau217/A*β*42 + pTau181/A*β*40 + pTau181/A*β*42 | 0.936 | 110 | 0.767 | 146 |
| pTau217 + NfL + pTau217/A*β*40 + pTau181/A*β*40 + pTau181/A*β*42 | 0.936 | 110 | 0.764 | 148 |
| pTau181 + pTau217/A*β*42 + pTau181/A*β*40 | 0.936 | 140 | 0.744 | 215 |
| A*β*42/40 + pTau217 + pTau181 + NfL + pTau217/A*β*40 + pTau217/A*β*42 | 0.935 | 77 | 0.85 | 107 |
| A*β*42/40 + pTau217 + pTau181 + NfL + pTau217/A*β*40 + pTau181/A*β*42 | 0.935 | 77 | 0.856 | 104 |
| A*β*42/40 + pTau181 + NfL + pTau217/A*β*40 | 0.935 | 77 | 0.85 | 107 |
| A*β*42/40 + pTau217 + pTau181 + NfL + pTau217/A*β*40 + pTau217/A*β*42 +  pTau181/A*β*42 | 0.935 | 77 | 0.856 | 104 |
| A*β*42/40 + pTau181 + NfL + pTau217/A*β*40 + pTau217/A*β*42 + pTau181/A*β*42 | 0.935 | 77 | 0.856 | 104 |
| A*β*42/40 + pTau181 + NfL + pTau217/A*β*40 + pTau181/A*β*42 | 0.935 | 77 | 0.856 | 104 |
| A*β*42/40 + pTau181 + NfL + pTau217/A*β*40 + pTau217/A*β*42 | 0.935 | 77 | 0.85 | 107 |
| pTau217 + pTau181 + pTau217/A*β*42 + pTau181/A*β*40 | 0.935 | 139 | 0.749 | 211 |
| A*β*42/40 + pTau217 + pTau181 + NfL + pTau217/A*β*40 | 0.935 | 77 | 0.85 | 107 |

| Combination | A+/T+ in BBM+ | n BBM+ | non-A+/T+ in BBM- | n BBM- |
| --- | --- | --- | --- | --- |
| A*β*42/40 + pTau217 + pTau181 + NfL + pTau217/A*β*40 + pTau181/A*β*40 +  pTau181/A*β*42 | 0.934 | 76 | 0.87 | 100 |
| A*β*42/40 + pTau181 + NfL + pTau217/A*β*40 + pTau217/A*β*42 + pTau181/A*β*40 +  pTau181/A*β*42 | 0.934 | 76 | 0.87 | 100 |
| A*β*42/40 + pTau181 + NfL + pTau217/A*β*40 + pTau181/A*β*40 | 0.934 | 76 | 0.87 | 100 |
| pTau181 + pTau217/A*β*42 + pTau181/A*β*40 + pTau181/A*β*42 | 0.934 | 136 | 0.75 | 212 |
| pTau217 + NfL + pTau217/A*β*40 + pTau217/A*β*42 | 0.934 | 121 | 0.735 | 162 |
| pTau217 + pTau181 + pTau217/A*β*42 + pTau181/A*β*42 | 0.934 | 136 | 0.75 | 212 |
| A*β*42/40 + pTau217 + pTau181 + NfL + pTau217/A*β*40 + pTau181/A*β*40 | 0.934 | 76 | 0.87 | 100 |
| A*β*42/40 + pTau181 + NfL + pTau217/A*β*40 + pTau181/A*β*40 + pTau181/A*β*42 | 0.934 | 76 | 0.87 | 100 |
| A*β*42/40 + pTau217 + pTau181 + NfL + pTau217/A*β*40 + pTau217/A*β*42 +  pTau181/A*β*40 | 0.934 | 76 | 0.87 | 100 |
| A*β*42/40 + pTau181 + NfL + pTau217/A*β*40 + pTau217/A*β*42 + pTau181/A*β*40 | 0.934 | 76 | 0.87 | 100 |
| pTau181 + pTau217/A*β*42 + pTau181/A*β*42 | 0.934 | 137 | 0.745 | 216 |
| NfL + pTau217/A*β*40 + pTau217/A*β*42 | 0.934 | 122 | 0.735 | 162 |
| pTau217/A*β*40 + pTau181/A*β*40 | 0.934 | 151 | 0.728 | 228 |
| A*β*42/40 + pTau217 + pTau181 + NfL + pTau217/A*β*40 + pTau217/A*β*42 + pTau181/A*β*40 + pTau181/A*β*42 | 0.934 | 76 | 0.87 | 100 |
| pTau217 + pTau181 + pTau217/A*β*42 + pTau181/A*β*40 + pTau181/A*β*42 | 0.933 | 135 | 0.755 | 208 |
| pTau217/A*β*40 + pTau217/A*β*42 + pTau181/A*β*40 | 0.933 | 149 | 0.734 | 222 |
| pTau217/A*β*40 + pTau181/A*β*42 | 0.932 | 147 | 0.729 | 225 |
| pTau217 + pTau181 + NfL | 0.932 | 118 | 0.729 | 166 |
| pTau181 + pTau217/A*β*42 | 0.932 | 146 | 0.737 | 224 |
| pTau217 + pTau217/A*β*40 + pTau217/A*β*42 + pTau181/A*β*40 | 0.932 | 146 | 0.738 | 221 |
| pTau217/A*β*40 + pTau217/A*β*42 + pTau181/A*β*42 | 0.932 | 146 | 0.734 | 222 |
| pTau217 + pTau217/A*β*40 + pTau181/A*β*40 | 0.932 | 148 | 0.731 | 227 |

| Combination | | A+/T+ in BBM+ | | n BBM+ | | non-A+/T+ in BBM- | | n BBM- | |
| --- | --- | --- | --- | --- | --- | --- | --- | --- | --- |
| pTau217/A*β*40 + pTau181/A*β*40 + pTau181/A*β*42 | | 0.931 | | 145 | | 0.736 | | 220 | |
| pTau217 + pTau217/A*β*40 + pTau181/A*β*42 | | 0.931 | | 144 | | 0.732 | | 224 | |
| NfL + pTau217/A*β*42 + pTau181/A*β*40 | | 0.931 | | 116 | | 0.745 | | 153 | |
| pTau217 + pTau181 + pTau217/A*β*42 | | 0.931 | | 145 | | 0.741 | | 220 | |
| pTau217/A*β*40 + pTau217/A*β*42 + pTau181/A*β*40 + pTau181/A*β*42 | | 0.931 | | 144 | | 0.742 | | 217 | |
| pTau217 + pTau181 + pTau181/A*β*40 | | 0.93 | | 143 | | 0.739 | | 218 | |
| NfL + pTau217/A*β*42 + pTau181/A*β*42 | | 0.93 | | 114 | | 0.748 | | 155 | |
| pTau217 + pTau217/A*β*40 + pTau181/A*β*40 + pTau181/A*β*42 | | 0.93 | | 142 | | 0.74 | | 219 | |
| pTau217 + pTau217/A*β*40 + pTau217/A*β*42 + pTau181/A*β*42 | | 0.93 | | 143 | | 0.738 | | 221 | |
| pTau217 + NfL + pTau217/A*β*42 + pTau181/A*β*40 | | 0.93 | | 115 | | 0.748 | | 151 | |
| pTau217 + pTau217/A*β*40 + pTau217/A*β*42 + pTau181/A*β*40 + pTau181/A*β*42 | | 0.929 | | 141 | | 0.745 | | 216 | |
| A*β*42/40 + NfL + pTau217/A*β*40 | | 0.929 | | 85 | | 0.839 | | 112 | |
| A*β*42/40 + NfL + pTau217/A*β*40 + pTau217/A*β*42 | | 0.929 | | 85 | | 0.839 | | 112 | |
| pTau217 + NfL + pTau217/A*β*42 + pTau181/A*β*42 | | 0.929 | | 113 | | 0.752 | | 153 | |
| A*β*42/40 + pTau217 + NfL + pTau217/A*β*40 + pTau217/A*β*42 | | 0.929 | | 84 | | 0.839 | | 112 | |
| NfL + pTau217/A*β*42 + pTau181/A*β*40 + pTau181/A*β*42 | | 0.929 | | 112 | | 0.755 | | 151 | |
| NfL + pTau217/A*β*40 | | 0.929 | | 126 | | 0.724 | | 170 | |
| A*β*42/40 + pTau217 + NfL + pTau217/A*β*40 | | 0.929 | | 84 | | 0.839 | | 112 | |
| pTau217/A*β*40 + pTau217/A*β*42 | | 0.928 | | 166 | | 0.71 | | 248 | |
| A*β*42/40 + NfL + pTau217/A*β*40 + pTau181/A*β*42 | | 0.928 | | 83 | | 0.848 | | 105 | |
| pTau217 + pTau181 + pTau181/A*β*40 + pTau181/A*β*42 | | 0.928 | | 138 | | 0.745 | | 212 | |
| NfL + pTau217/A*β*42 | | 0.928 | | 125 | | 0.725 | | 167 | |
| pTau217 + NfL + pTau217/A*β*40 | | 0.928 | | 125 | | 0.728 | | 169 | |
| pTau217 + pTau181 + pTau181/A*β*42 | | 0.928 | | 139 | | 0.741 | | 216 | |
| A*β*42/40 + NfL + pTau217/A*β*40 + pTau217/A*β*42 + pTau181/A*β*42 | | 0.928 | | 83 | | 0.848 | | 105 | |
| pTau217 + NfL + pTau217/A*β*42 + pTau181/A*β*40 + pTau181/A*β*42 | | 0.928 | | 111 | | 0.758 | | 149 | |
| A*β*42/40 + pTau217 + NfL + pTau217/A*β*40 + pTau217/A*β*42 + pTau181/A*β*42 | | 0.927 | | 82 | | 0.848 | | 105 | |
|  | |  | |  | |  | | | |
| Combination | A+/T+ in BBM+ | | n BBM+ | | non-A+/T+ in BBM- | | n BBM- | |  |
| A*β*42/40 + pTau217 + NfL + pTau217/A*β*40 + pTau181/A*β*42 | 0.927 | | 82 | | 0.848 | | 105 | |  |
| pTau217 + NfL + pTau217/A*β*42 | 0.927 | | 123 | | 0.727 | | 165 | |  |
| pTau217 + pTau181 | 0.927 | | 150 | | 0.723 | | 231 | |  |
| pTau217 + pTau217/A*β*40 + pTau217/A*β*42 | 0.926 | | 162 | | 0.714 | | 245 | |  |
| pTau217 + pTau217/A*β*42 + pTau181/A*β*40 | 0.926 | | 149 | | 0.732 | | 224 | |  |
| A*β*42/40 + NfL + pTau217/A*β*40 + pTau181/A*β*40 | 0.926 | | 81 | | 0.861 | | 101 | |  |
| A*β*42/40 + NfL + pTau217/A*β*40 + pTau217/A*β*42 + pTau181/A*β*40 | 0.926 | | 81 | | 0.861 | | 101 | |  |
| A*β*42/40 + NfL + pTau217/A*β*40 + pTau217/A*β*42 + pTau181/A*β*40 +  pTau181/A*β*42 | 0.926 | | 81 | | 0.861 | | 101 | |  |
| A*β*42/40 + NfL + pTau217/A*β*40 + pTau181/A*β*40 + pTau181/A*β*42 | 0.926 | | 81 | | 0.861 | | 101 | |  |
| A*β*42/40 + pTau217 + NfL + pTau217/A*β*40 + pTau181/A*β*40 | 0.925 | | 80 | | 0.861 | | 101 | |  |
| A*β*42/40 + pTau217 + pTau181 + NfL | 0.925 | | 80 | | 0.829 | | 111 | |  |
| pTau217 + pTau217/A*β*42 + pTau181/A*β*42 | 0.925 | | 146 | | 0.732 | | 224 | |  |
| A*β*42/40 + pTau217 + NfL + pTau217/A*β*40 + pTau217/A*β*42 + pTau181/A*β*40 | 0.925 | | 80 | | 0.861 | | 101 | |  |
| A*β*42/40 + pTau217 + NfL + pTau217/A*β*40 + pTau181/A*β*40 + pTau181/A*β*42 | 0.925 | | 80 | | 0.861 | | 101 | |  |
| A*β*42/40 + pTau217 + NfL + pTau217/A*β*40 + pTau217/A*β*42 + pTau181/A*β*40 +  pTau181/A*β*42 | 0.925 | | 80 | | 0.861 | | 101 | |  |
| pTau217 + pTau217/A*β*42 + pTau181/A*β*40 + pTau181/A*β*42 | 0.924 | | 144 | | 0.74 | | 219 | |  |
| pTau217 + NfL + pTau181/A*β*40 | 0.924 | | 119 | | 0.739 | | 157 | |  |
| A*β*42/40 + pTau181 + NfL + pTau217/A*β*42 | 0.924 | | 79 | | 0.829 | | 111 | |  |
| A*β*42/40 + pTau217 + pTau181 + NfL + pTau181/A*β*42 | 0.924 | | 79 | | 0.841 | | 107 | |  |
| A*β*42/40 + pTau217 + pTau181 + NfL + pTau217/A*β*42 | 0.924 | | 79 | | 0.836 | | 110 | |  |
| A*β*42/40 + pTau217 + pTau181 + NfL + pTau217/A*β*42 + pTau181/A*β*42 | 0.924 | | 79 | | 0.841 | | 107 | |  |
| A*β*42/40 + pTau181 + NfL + pTau217/A*β*42 + pTau181/A*β*42 | 0.924 | | 79 | | 0.833 | | 108 | |  |
| A*β*42/40 + pTau181 + NfL + pTau217/A*β*42 + pTau181/A*β*40 + pTau181/A*β*42 | 0.923 | | 78 | | 0.846 | | 104 | |  |

| Combination | A+/T+ in BBM+ | n BBM+ | non-A+/T+ in BBM- | n BBM- |
| --- | --- | --- | --- | --- |
| A*β*42/40 + pTau217 + pTau181 + NfL + pTau217/A*β*42 + pTau181/A*β*40 +  pTau181/A*β*42 | 0.923 | 78 | 0.854 | 103 |
| A*β*42/40 + pTau217 + pTau181 + NfL + pTau217/A*β*42 + pTau181/A*β*40 | 0.923 | 78 | 0.854 | 103 |
| A*β*42/40 + pTau217 + pTau181 + NfL + pTau181/A*β*40 | 0.923 | 78 | 0.854 | 103 |
| A*β*42/40 + pTau217 + pTau181 + NfL + pTau181/A*β*40 + pTau181/A*β*42 | 0.923 | 78 | 0.854 | 103 |
| A*β*42/40 + pTau181 + NfL + pTau217/A*β*42 + pTau181/A*β*40 | 0.923 | 78 | 0.846 | 104 |
| A*β*42/40 + pTau181 + pTau217/A*β*40 + pTau217/A*β*42 + pTau181/A*β*42 | 0.922 | 102 | 0.833 | 144 |
| A*β*42/40 + pTau217 + pTau181 + pTau217/A*β*40 | 0.922 | 102 | 0.83 | 147 |
| A*β*42/40 + pTau181 + pTau217/A*β*40 + pTau217/A*β*42 | 0.922 | 102 | 0.83 | 147 |
| pTau217 + NfL + pTau181/A*β*42 | 0.922 | 116 | 0.744 | 156 |
| A*β*42/40 + pTau217 + pTau181 + pTau217/A*β*40 + pTau181/A*β*42 | 0.922 | 102 | 0.833 | 144 |
| A*β*42/40 + pTau217 + pTau181 + pTau217/A*β*40 + pTau217/A*β*42 | 0.922 | 102 | 0.83 | 147 |
| A*β*42/40 + pTau217 + pTau181 + pTau217/A*β*40 + pTau217/A*β*42 +  pTau181/A*β*42 | 0.922 | 102 | 0.833 | 144 |
| A*β*42/40 + pTau181 + pTau217/A*β*40 | 0.922 | 102 | 0.83 | 147 |
| pTau217/A*β*42 + pTau181/A*β*40 | 0.922 | 154 | 0.725 | 229 |
| A*β*42/40 + pTau181 + pTau217/A*β*40 + pTau181/A*β*42 | 0.922 | 102 | 0.833 | 144 |
| pTau217 + pTau181/A*β*40 | 0.922 | 153 | 0.723 | 231 |
| pTau217 + pTau217/A*β*42 | 0.921 | 165 | 0.71 | 248 |
| A*β*42/40 + pTau181 + pTau217/A*β*40 + pTau181/A*β*40 | 0.921 | 101 | 0.843 | 140 |
| A*β*42/40 + pTau217 + pTau181 + pTau217/A*β*40 + pTau217/A*β*42 + pTau181/A*β*40 + pTau181/A*β*42 | 0.921 | 101 | 0.843 | 140 |
| A*β*42/40 + pTau181 + pTau217/A*β*40 + pTau217/A*β*42 + pTau181/A*β*40 +  pTau181/A*β*42 | 0.921 | 101 | 0.843 | 140 |
| A*β*42/40 + pTau217 + pTau181 + pTau217/A*β*40 + pTau181/A*β*40 +  pTau181/A*β*42 | 0.921 | 101 | 0.843 | 140 |

| Combination | A+/T+ in BBM+ | n BBM+ | non-A+/T+ in BBM- | n BBM- |
| --- | --- | --- | --- | --- |
| A*β*42/40 + pTau217 + pTau181 + pTau217/A*β*40 + pTau217/A*β*42 +  pTau181/A*β*40 | 0.921 | 101 | 0.843 | 140 |
| A*β*42/40 + pTau217 + pTau181 + pTau217/A*β*40 + pTau181/A*β*40 | 0.921 | 101 | 0.843 | 140 |
| A*β*42/40 + pTau181 + pTau217/A*β*40 + pTau217/A*β*42 + pTau181/A*β*40 | 0.921 | 101 | 0.843 | 140 |
| pTau217 + NfL + pTau181/A*β*40 + pTau181/A*β*42 | 0.921 | 114 | 0.75 | 152 |
| A*β*42/40 + pTau181 + pTau217/A*β*40 + pTau181/A*β*40 + pTau181/A*β*42 | 0.921 | 101 | 0.843 | 140 |
| A*β*42/40 + pTau217 + NfL | 0.92 | 87 | 0.819 | 116 |
| A*β*42/40 + NfL + pTau217/A*β*42 | 0.92 | 88 | 0.819 | 116 |
| A*β*42/40 + pTau217 + NfL + pTau217/A*β*42 | 0.919 | 86 | 0.826 | 115 |
| A*β*42/40 + pTau217/A*β*40 + pTau217/A*β*42 + pTau181/A*β*42 | 0.919 | 111 | 0.815 | 151 |
| pTau217/A*β*42 + pTau181/A*β*40 + pTau181/A*β*42 | 0.919 | 149 | 0.732 | 224 |
| A*β*42/40 + pTau217/A*β*40 + pTau181/A*β*42 | 0.919 | 111 | 0.815 | 151 |
| pTau217 + pTau181/A*β*42 | 0.919 | 149 | 0.724 | 228 |
| pTau217 + pTau181/A*β*40 + pTau181/A*β*42 | 0.918 | 147 | 0.731 | 223 |
| A*β*42/40 + NfL + pTau217/A*β*42 + pTau181/A*β*42 | 0.918 | 85 | 0.826 | 109 |
| pTau217 + pTau217/A*β*40 | 0.917 | 168 | 0.708 | 253 |
| A*β*42/40 + pTau217 + NfL + pTau217/A*β*42 + pTau181/A*β*42 | 0.917 | 84 | 0.833 | 108 |
| A*β*42/40 + pTau217 + pTau217/A*β*40 + pTau217/A*β*42 + pTau181/A*β*42 | 0.917 | 109 | 0.815 | 151 |
| A*β*42/40 + pTau217/A*β*40 + pTau217/A*β*42 + pTau181/A*β*40 + pTau181/A*β*42 | 0.917 | 109 | 0.829 | 146 |
| A*β*42/40 + pTau217/A*β*40 + pTau181/A*β*40 + pTau181/A*β*42 | 0.917 | 109 | 0.829 | 146 |
| A*β*42/40 + pTau217 + pTau217/A*β*40 + pTau181/A*β*42 | 0.917 | 109 | 0.815 | 151 |
| A*β*42/40 + pTau217/A*β*40 + pTau181/A*β*40 | 0.917 | 109 | 0.829 | 146 |
| A*β*42/40 + pTau217 + NfL + pTau181/A*β*42 | 0.917 | 84 | 0.833 | 108 |
| A*β*42/40 + pTau217/A*β*40 + pTau217/A*β*42 + pTau181/A*β*40 | 0.917 | 109 | 0.829 | 146 |
| A*β*42/40 + pTau217 + pTau217/A*β*40 + pTau217/A*β*42 + pTau181/A*β*40 | 0.916 | 107 | 0.829 | 146 |
| A*β*42/40 + pTau217 + pTau217/A*β*40 + pTau181/A*β*40 + pTau181/A*β*42 | 0.916 | 107 | 0.829 | 146 |
|  |  |  |  | |

| Combination | A+/T+ in BBM+ | n BBM+ | non-A+/T+ in BBM- | n BBM- |
| --- | --- | --- | --- | --- |
| A*β*42/40 + pTau217 + pTau217/A*β*40 + pTau217/A*β*42 + pTau181/A*β*40 +  pTau181/A*β*42 | 0.916 | 107 | 0.829 | 146 |
| A*β*42/40 + pTau217 + pTau217/A*β*40 + pTau181/A*β*40 | 0.916 | 107 | 0.829 | 146 |
| A*β*42/40 + NfL + pTau217/A*β*42 + pTau181/A*β*40 | 0.916 | 83 | 0.838 | 105 |
| A*β*42/40 + NfL + pTau217/A*β*42 + pTau181/A*β*40 + pTau181/A*β*42 | 0.916 | 83 | 0.838 | 105 |
| A*β*42/40 + pTau217 + pTau181 | 0.915 | 106 | 0.815 | 151 |
| A*β*42/40 + pTau217/A*β*40 + pTau217/A*β*42 | 0.915 | 118 | 0.809 | 162 |
| A*β*42/40 + pTau217 + NfL + pTau181/A*β*40 | 0.915 | 82 | 0.846 | 104 |
| pTau217 + NfL | 0.915 | 130 | 0.709 | 175 |
| A*β*42/40 + pTau181 + pTau217/A*β*42 | 0.915 | 106 | 0.81 | 153 |
| A*β*42/40 + pTau217 + NfL + pTau217/A*β*42 + pTau181/A*β*40 + pTau181/A*β*42 | 0.915 | 82 | 0.846 | 104 |
| A*β*42/40 + pTau217 + NfL + pTau217/A*β*42 + pTau181/A*β*40 | 0.915 | 82 | 0.846 | 104 |
| A*β*42/40 + pTau181 + pTau217/A*β*42 + pTau181/A*β*42 | 0.915 | 106 | 0.813 | 150 |
| A*β*42/40 + pTau217 + NfL + pTau181/A*β*40 + pTau181/A*β*42 | 0.915 | 82 | 0.846 | 104 |
| A*β*42/40 + pTau217 + pTau181 + pTau217/A*β*42 | 0.914 | 105 | 0.82 | 150 |
| pTau217/A*β*40 | 0.914 | 175 | 0.699 | 259 |
| A*β*42/40 + pTau181 + pTau217/A*β*42 + pTau181/A*β*40 | 0.914 | 105 | 0.822 | 146 |
| A*β*42/40 + pTau181 + pTau217/A*β*42 + pTau181/A*β*40 + pTau181/A*β*42 | 0.914 | 105 | 0.822 | 146 |
| A*β*42/40 + pTau217 + pTau181 + pTau217/A*β*42 + pTau181/A*β*42 | 0.914 | 105 | 0.823 | 147 |
| A*β*42/40 + pTau217 + pTau181 + pTau181/A*β*42 | 0.914 | 105 | 0.823 | 147 |
| A*β*42/40 + pTau217 + pTau181 + pTau181/A*β*40 + pTau181/A*β*42 | 0.913 | 104 | 0.832 | 143 |
| A*β*42/40 + pTau217 + pTau181 + pTau217/A*β*42 + pTau181/A*β*40 +  pTau181/A*β*42 | 0.913 | 104 | 0.832 | 143 |
| A*β*42/40 + pTau217 + pTau217/A*β*40 + pTau217/A*β*42 | 0.913 | 115 | 0.812 | 160 |
| A*β*42/40 + pTau217 + pTau181 + pTau181/A*β*40 | 0.913 | 104 | 0.832 | 143 |
|  |  |  |  | |

|  | Combination | | A+/T+ in BBM+ | | n BBM+ | | non-A+/T+ in BBM- | | n BBM- | |
| --- | --- | --- | --- | --- | --- | --- | --- | --- | --- | --- |
|  | A*β*42/40 + pTau217 + pTau181 + pTau217/A*β*42 + pTau181/A*β*40 | | 0.913 | | 104 | | 0.832 | | 143 | |
|  | A*β*42/40 + pTau217 + pTau181/A*β*42 | | 0.911 | | 112 | | 0.805 | | 154 | |
|  | A*β*42/40 + pTau217 + pTau217/A*β*42 + pTau181/A*β*42 | | 0.911 | | 112 | | 0.805 | | 154 | |
|  | pTau181 + NfL + pTau181/A*β*40 | | 0.91 | | 122 | | 0.724 | | 163 | |
|  | A*β*42/40 + pTau217 + pTau217/A*β*42 + pTau181/A*β*40 | | 0.909 | | 110 | | 0.819 | | 149 | |
|  | A*β*42/40 + pTau217 + pTau181/A*β*40 + pTau181/A*β*42 | | 0.909 | | 110 | | 0.819 | | 149 | |
|  | A*β*42/40 + pTau217 + pTau217/A*β*42 + pTau181/A*β*40 + pTau181/A*β*42 | | 0.909 | | 110 | | 0.819 | | 149 | |
|  | A*β*42/40 + pTau217 + pTau181/A*β*40 | | 0.909 | | 110 | | 0.819 | | 149 | |
|  | pTau217/A*β*42 + pTau181/A*β*42 | | 0.909 | | 154 | | 0.722 | | 230 | |
|  | pTau217 | | 0.908 | | 174 | | 0.692 | | 260 | |
|  | A*β*42/40 + pTau217/A*β*40 | | 0.908 | | 119 | | 0.809 | | 162 | |
|  | A*β*42/40 + pTau217 + pTau217/A*β*42 | | 0.907 | | 118 | | 0.804 | | 163 | |
|  | pTau181 + NfL + pTau181/A*β*40 + pTau181/A*β*42 | | 0.906 | | 117 | | 0.734 | | 158 | |
|  | A*β*42/40 + pTau181 + NfL + pTau181/A*β*40 | | 0.905 | | 84 | | 0.83 | | 106 | |
|  | A*β*42/40 + pTau217 + pTau217/A*β*40 | | 0.905 | | 116 | | 0.812 | | 160 | |
|  | A*β*42/40 + pTau181 + NfL + pTau181/A*β*40 + pTau181/A*β*42 | | 0.905 | | 84 | | 0.83 | | 106 | |
|  | pTau217/A*β*42 | | 0.904 | | 177 | | 0.696 | | 257 | |
|  | A*β*42/40 + pTau217/A*β*42 + pTau181/A*β*40 + pTau181/A*β*42 | | 0.904 | | 114 | | 0.809 | | 152 | |
|  | A*β*42/40 + pTau217/A*β*42 + pTau181/A*β*40 | | 0.904 | | 114 | | 0.809 | | 152 | |
|  | A*β*42/40 + pTau181 + pTau181/A*β*40 | | 0.903 | | 113 | | 0.805 | | 149 | |
|  | A*β*42/40 + pTau181 + pTau181/A*β*40 + pTau181/A*β*42 | | 0.903 | | 113 | | 0.805 | | 149 | |
|  | pTau181 + NfL + pTau181/A*β*42 | | 0.9 | | 120 | | 0.728 | | 162 | |
|  | A*β*42/40 + pTau217 | | 0.9 | | 120 | | 0.799 | | 164 | |
|  | A*β*42/40 + pTau181 + pTau181/A*β*42 | | 0.897 | | 116 | | 0.792 | | 154 | |
|  | A*β*42/40 + pTau181 + NfL + pTau181/A*β*42 | | 0.897 | | 87 | | 0.818 | | 110 | |
|  | pTau181 + pTau181/A*β*40 | | 0.893 | | 159 | | 0.707 | | 232 | |
|  | NfL + pTau181/A*β*40 | | 0.892 | | 130 | | 0.707 | | 167 | |
|  | A*β*42/40 + pTau217/A*β*42 + pTau181/A*β*42 | | 0.891 | | 119 | | 0.791 | | 158 | |
|  | A*β*42/40 + NfL + pTau181/A*β*40 | | 0.889 | | 90 | | 0.807 | | 109 | |
|  | pTau181 + pTau181/A*β*40 + pTau181/A*β*42 | | 0.889 | | 153 | | 0.716 | | 225 | |
|  | A*β*42/40 + NfL + pTau181/A*β*40 + pTau181/A*β*42 | | 0.889 | | 90 | | 0.807 | | 109 | |
| Combination | | A+/T+ in BBM+ | | n BBM+ | | non-A+/T+ in BBM- | | n BBM- | |  |
| A*β*42/40 + pTau181/A*β*40 + pTau181/A*β*42 | | 0.887 | | 124 | | 0.775 | | 160 | |  |
| A*β*42/40 + pTau181/A*β*40 | | 0.887 | | 124 | | 0.775 | | 160 | |  |
| NfL + pTau181/A*β*40 + pTau181/A*β*42 | | 0.887 | | 124 | | 0.716 | | 162 | |  |
| A*β*42/40 + pTau181 | | 0.885 | | 122 | | 0.776 | | 161 | |  |
| pTau181 + pTau181/A*β*42 | | 0.885 | | 156 | | 0.709 | | 230 | |  |
| A*β*42/40 + pTau217/A*β*42 | | 0.884 | | 129 | | 0.782 | | 170 | |  |
| A*β*42/40 + pTau181 + NfL | | 0.88 | | 92 | | 0.793 | | 116 | |  |
| NfL + pTau181/A*β*42 | | 0.876 | | 129 | | 0.707 | | 167 | |  |
| A*β*42/40 + NfL + pTau181/A*β*42 | | 0.874 | | 95 | | 0.789 | | 114 | |  |
| pTau181 + NfL | | 0.87 | | 138 | | 0.691 | | 178 | |  |
| A*β*42/40 + pTau181/A*β*42 | | 0.866 | | 134 | | 0.737 | | 171 | |  |
| pTau181 | | 0.866 | | 179 | | 0.675 | | 255 | |  |
| pTau181/A*β*40 + pTau181/A*β*42 | | 0.865 | | 171 | | 0.69 | | 242 | |  |
| pTau181/A*β*40 | | 0.863 | | 182 | | 0.679 | | 252 | |  |
| pTau181/A*β*42 | | 0.851 | | 181 | | 0.668 | | 253 | |  |
| A*β*42/40 + NfL | | 0.782 | | 124 | | 0.748 | | 127 | |  |
| A*β*42/40 | | 0.718 | | 216 | | 0.619 | | 218 | |  |
| NfL | | 0.688 | | 215 | | 0.589 | | 219 | |  |

Supplementary table 14 Cutoff combinations — Birch (all 255 combinations, eight biomarkers). Prevalence of A^+^/T^+^ CSF profile in BBM-positive subgroups and prevalence of non-A^+^/T^+^ CSF profile in BBM-negative subgroups.

| Combination | A+/T+ in BBM+ | n BBM+ | non-A+/T+ in BBM- | n BBM- |
| --- | --- | --- | --- | --- |
| pTau217 + pTau181 + NfL + pTau217/A*β*42 + pTau181/A*β*40 | 0.943 | 123 | 0.755 | 139 |
| pTau181 + NfL + pTau217/A*β*40 + pTau217/A*β*42 + pTau181/A*β*40 | 0.943 | 123 | 0.759 | 137 |
| pTau217 + pTau181 + NfL + pTau217/A*β*40 + pTau217/A*β*42 + pTau181/A*β*40 | 0.943 | 123 | 0.759 | 137 |
| pTau217 + pTau181 + NfL + pTau217/A*β*40 + pTau181/A*β*40 | 0.943 | 123 | 0.764 | 140 |
| pTau181 + NfL + pTau217/A*β*40 + pTau181/A*β*40 | 0.943 | 123 | 0.766 | 141 |
| pTau181 + NfL + pTau217/A*β*42 + pTau181/A*β*40 | 0.943 | 123 | 0.75 | 140 |
| pTau181 + NfL + pTau217/A*β*42 + pTau181/A*β*42 | 0.942 | 120 | 0.755 | 139 |
| pTau181 + NfL + pTau217/A*β*40 + pTau181/A*β*42 | 0.942 | 120 | 0.768 | 138 |

| Combination | A+/T+ in BBM+ | n BBM+ | non-A+/T+ in BBM- | n BBM- |
| --- | --- | --- | --- | --- |
| pTau217 + pTau181 + NfL + pTau217/A*β*42 + pTau181/A*β*42 | 0.942 | 120 | 0.761 | 138 |
| pTau217 + pTau181 + NfL + pTau217/A*β*40 + pTau217/A*β*42 + pTau181/A*β*42 | 0.942 | 120 | 0.765 | 136 |
| pTau217 + pTau181 + NfL + pTau217/A*β*40 + pTau181/A*β*42 | 0.942 | 120 | 0.766 | 137 |
| pTau181 + NfL + pTau217/A*β*40 + pTau217/A*β*42 + pTau181/A*β*42 | 0.942 | 120 | 0.765 | 136 |
| pTau217 + pTau181 + NfL + pTau217/A*β*42 + pTau181/A*β*40 + pTau181/A*β*42 | 0.941 | 119 | 0.765 | 136 |
| pTau181 + NfL + pTau217/A*β*40 + pTau217/A*β*42 + pTau181/A*β*40 +  pTau181/A*β*42 | 0.941 | 119 | 0.769 | 134 |
| pTau217 + pTau181 + NfL + pTau217/A*β*40 + pTau217/A*β*42 + pTau181/A*β*40 +  pTau181/A*β*42 | 0.941 | 119 | 0.769 | 134 |
| pTau217 + pTau181 + NfL + pTau217/A*β*40 + pTau181/A*β*40 + pTau181/A*β*42 | 0.941 | 119 | 0.77 | 135 |
| pTau181 + NfL + pTau217/A*β*40 + pTau181/A*β*40 + pTau181/A*β*42 | 0.941 | 119 | 0.772 | 136 |
| pTau181 + NfL + pTau217/A*β*42 + pTau181/A*β*40 + pTau181/A*β*42 | 0.941 | 119 | 0.759 | 137 |
| pTau181 + pTau217/A*β*40 + pTau181/A*β*40 | 0.938 | 145 | 0.766 | 209 |
| pTau217 + pTau181 + pTau217/A*β*40 + pTau217/A*β*42 + pTau181/A*β*40 | 0.938 | 144 | 0.765 | 204 |
| pTau217 + pTau181 + pTau217/A*β*42 + pTau181/A*β*40 | 0.938 | 145 | 0.762 | 206 |
| pTau181 + pTau217/A*β*42 + pTau181/A*β*40 | 0.938 | 146 | 0.755 | 208 |
| pTau181 + pTau217/A*β*40 + pTau217/A*β*42 + pTau181/A*β*40 | 0.938 | 145 | 0.761 | 205 |
| pTau217 + pTau181 + pTau217/A*β*40 + pTau181/A*β*40 | 0.938 | 144 | 0.768 | 207 |
| pTau217 + pTau181 + NfL + pTau217/A*β*40 + pTau217/A*β*42 | 0.937 | 126 | 0.748 | 143 |
| pTau217 + pTau181 + NfL + pTau217/A*β*40 | 0.937 | 126 | 0.75 | 148 |
| pTau181 + NfL + pTau217/A*β*40 | 0.937 | 126 | 0.752 | 149 |
| pTau181 + NfL + pTau217/A*β*42 | 0.937 | 126 | 0.74 | 146 |
| pTau181 + pTau217/A*β*42 + pTau181/A*β*42 | 0.937 | 143 | 0.757 | 206 |
| pTau181 + pTau217/A*β*40 + pTau181/A*β*42 | 0.937 | 142 | 0.766 | 205 |
| pTau181 + NfL + pTau217/A*β*40 + pTau217/A*β*42 | 0.937 | 126 | 0.748 | 143 |

| Combination | A+/T+ in BBM+ | n BBM+ | non-A+/T+ in BBM- | n BBM- |
| --- | --- | --- | --- | --- |
| pTau217 + pTau181 + NfL + pTau217/A*β*42 | 0.937 | 126 | 0.745 | 145 |
| pTau181 + pTau217/A*β*40 + pTau217/A*β*42 + pTau181/A*β*42 | 0.937 | 142 | 0.764 | 203 |
| pTau217 + pTau181 + pTau217/A*β*42 + pTau181/A*β*42 | 0.937 | 142 | 0.765 | 204 |
| pTau217 + pTau181 + NfL + pTau181/A*β*40 | 0.937 | 126 | 0.75 | 144 |
| pTau181 + pTau217/A*β*42 + pTau181/A*β*40 + pTau181/A*β*42 | 0.937 | 142 | 0.76 | 204 |
| pTau217 + pTau181 + pTau217/A*β*40 + pTau181/A*β*42 | 0.936 | 141 | 0.768 | 203 |
| pTau181 + pTau217/A*β*40 + pTau181/A*β*40 + pTau181/A*β*42 | 0.936 | 141 | 0.768 | 203 |
| pTau181 + pTau217/A*β*40 + pTau217/A*β*42 + pTau181/A*β*40 + pTau181/A*β*42 | 0.936 | 141 | 0.766 | 201 |
| pTau217 + pTau181 + pTau217/A*β*40 + pTau181/A*β*40 + pTau181/A*β*42 | 0.936 | 140 | 0.771 | 201 |
| pTau217 + pTau181 + pTau217/A*β*40 + pTau217/A*β*42 + pTau181/A*β*40 +  pTau181/A*β*42 | 0.936 | 140 | 0.77 | 200 |
| pTau217 + pTau181 + pTau217/A*β*40 + pTau217/A*β*42 + pTau181/A*β*42 | 0.936 | 141 | 0.767 | 202 |
| pTau217 + pTau181 + pTau217/A*β*42 + pTau181/A*β*40 + pTau181/A*β*42 | 0.936 | 141 | 0.767 | 202 |
| A*β*42/40 + pTau181 + NfL + pTau217/A*β*40 | 0.934 | 106 | 0.889 | 81 |
| A*β*42/40 + pTau181 + NfL + pTau217/A*β*42 | 0.934 | 106 | 0.869 | 84 |
| A*β*42/40 + pTau217 + pTau181 + NfL + pTau217/A*β*42 | 0.934 | 106 | 0.88 | 83 |
| pTau181 + pTau217/A*β*42 | 0.934 | 151 | 0.749 | 215 |
| A*β*42/40 + pTau181 + NfL + pTau217/A*β*40 + pTau217/A*β*42 | 0.934 | 106 | 0.889 | 81 |
| A*β*42/40 + pTau217 + pTau181 + NfL + pTau217/A*β*40 | 0.934 | 106 | 0.889 | 81 |
| pTau217 + pTau181 + NfL + pTau181/A*β*40 + pTau181/A*β*42 | 0.934 | 121 | 0.761 | 138 |
| A*β*42/40 + pTau217 + pTau181 + NfL + pTau217/A*β*40 + pTau217/A*β*42 | 0.934 | 106 | 0.889 | 81 |
| A*β*42/40 + pTau181 + NfL + pTau217/A*β*40 + pTau217/A*β*42 + pTau181/A*β*42 | 0.933 | 105 | 0.897 | 78 |
| A*β*42/40 + pTau217 + pTau181 + NfL + pTau217/A*β*42 + pTau181/A*β*42 | 0.933 | 105 | 0.888 | 80 |
| A*β*42/40 + pTau217 + pTau181 + NfL + pTau217/A*β*40 + pTau181/A*β*40 | 0.933 | 104 | 0.896 | 77 |
|  |  |  |  | |

| Combination | A+/T+ in BBM+ | n BBM+ | non-A+/T+ in BBM- | n BBM- |
| --- | --- | --- | --- | --- |
| A*β*42/40 + pTau217 + pTau181 + NfL + pTau217/A*β*42 + pTau181/A*β*40 | 0.933 | 104 | 0.886 | 79 |
| A*β*42/40 + pTau217 + pTau181 + NfL + pTau181/A*β*40 + pTau181/A*β*42 | 0.933 | 105 | 0.875 | 80 |
| A*β*42/40 + pTau217 + pTau181 + NfL + pTau217/A*β*40 + pTau181/A*β*42 | 0.933 | 105 | 0.897 | 78 |
| pTau181 + pTau217/A*β*40 | 0.933 | 150 | 0.757 | 218 |
| pTau217 + pTau181 + pTau217/A*β*40 | 0.933 | 149 | 0.759 | 216 |
| pTau217 + pTau181 + pTau217/A*β*42 | 0.933 | 150 | 0.756 | 213 |
| pTau181 + pTau217/A*β*40 + pTau217/A*β*42 | 0.933 | 150 | 0.755 | 212 |
| A*β*42/40 + pTau181 + NfL + pTau217/A*β*42 + pTau181/A*β*40 + pTau181/A*β*42 | 0.933 | 104 | 0.875 | 80 |
| A*β*42/40 + pTau217 + pTau181 + NfL + pTau217/A*β*40 + pTau217/A*β*42 +  pTau181/A*β*40 | 0.933 | 104 | 0.896 | 77 |
| A*β*42/40 + pTau181 + NfL + pTau217/A*β*40 + pTau181/A*β*40 | 0.933 | 104 | 0.896 | 77 |
| A*β*42/40 + pTau217 + pTau181 + NfL + pTau217/A*β*40 + pTau181/A*β*40 +  pTau181/A*β*42 | 0.933 | 104 | 0.896 | 77 |
| A*β*42/40 + pTau181 + NfL + pTau217/A*β*40 + pTau217/A*β*42 + pTau181/A*β*40 +  pTau181/A*β*42 | 0.933 | 104 | 0.896 | 77 |
| A*β*42/40 + pTau217 + pTau181 + NfL + pTau217/A*β*40 + pTau217/A*β*42 + pTau181/A*β*40 + pTau181/A*β*42 | 0.933 | 104 | 0.896 | 77 |
| A*β*42/40 + pTau181 + NfL + pTau217/A*β*40 + pTau181/A*β*40 + pTau181/A*β*42 | 0.933 | 104 | 0.896 | 77 |
| A*β*42/40 + pTau217 + pTau181 + NfL + pTau217/A*β*42 + pTau181/A*β*40 +  pTau181/A*β*42 | 0.933 | 104 | 0.886 | 79 |
| A*β*42/40 + pTau217 + pTau181 + NfL + pTau217/A*β*40 + pTau217/A*β*42 +  pTau181/A*β*42 | 0.933 | 105 | 0.897 | 78 |
| A*β*42/40 + pTau181 + NfL + pTau217/A*β*42 + pTau181/A*β*42 | 0.933 | 105 | 0.877 | 81 |
| pTau217 + pTau181 + pTau217/A*β*40 + pTau217/A*β*42 | 0.933 | 149 | 0.758 | 211 |
| A*β*42/40 + pTau181 + NfL + pTau217/A*β*40 + pTau181/A*β*42 | 0.933 | 105 | 0.897 | 78 |
| A*β*42/40 + pTau181 + NfL + pTau217/A*β*42 + pTau181/A*β*40 | 0.933 | 104 | 0.875 | 80 |
|  |  |  |  | |

| Combination | A+/T+ in BBM+ | n BBM+ | non-A+/T+ in BBM- | n BBM- |
| --- | --- | --- | --- | --- |
| A*β*42/40 + pTau217 + pTau181 + NfL + pTau181/A*β*40 | 0.933 | 105 | 0.875 | 80 |
| A*β*42/40 + pTau181 + NfL + pTau217/A*β*40 + pTau217/A*β*42 + pTau181/A*β*40 | 0.933 | 104 | 0.896 | 77 |
| pTau217 + pTau181 + pTau181/A*β*40 | 0.932 | 148 | 0.758 | 211 |
| NfL + pTau217/A*β*40 + pTau217/A*β*42 + pTau181/A*β*40 | 0.931 | 130 | 0.754 | 138 |
| NfL + pTau217/A*β*42 + pTau181/A*β*40 | 0.931 | 130 | 0.745 | 141 |
| NfL + pTau217/A*β*40 + pTau181/A*β*40 | 0.931 | 130 | 0.761 | 142 |
| pTau217 + NfL + pTau217/A*β*40 + pTau217/A*β*42 + pTau181/A*β*40 | 0.931 | 130 | 0.754 | 138 |
| pTau217 + NfL + pTau217/A*β*40 + pTau181/A*β*40 | 0.931 | 130 | 0.759 | 141 |
| pTau217 + NfL + pTau217/A*β*42 + pTau181/A*β*40 | 0.931 | 130 | 0.75 | 140 |
| A*β*42/40 + pTau181 + pTau217/A*β*40 | 0.93 | 128 | 0.855 | 117 |
| A*β*42/40 + pTau217 + pTau181 + pTau217/A*β*42 | 0.93 | 128 | 0.856 | 118 |
| A*β*42/40 + pTau181 + pTau217/A*β*40 + pTau217/A*β*42 | 0.93 | 128 | 0.855 | 117 |
| NfL + pTau217/A*β*42 + pTau181/A*β*42 | 0.93 | 128 | 0.746 | 142 |
| pTau217 + NfL + pTau217/A*β*40 + pTau217/A*β*42 + pTau181/A*β*42 | 0.93 | 128 | 0.755 | 139 |
| pTau217 + NfL + pTau217/A*β*42 + pTau181/A*β*42 | 0.93 | 128 | 0.752 | 141 |
| NfL + pTau217/A*β*40 + pTau217/A*β*42 + pTau181/A*β*42 | 0.93 | 128 | 0.755 | 139 |
| pTau217 + NfL + pTau217/A*β*40 + pTau181/A*β*42 | 0.93 | 128 | 0.757 | 140 |
| pTau217 + pTau181 + pTau181/A*β*40 + pTau181/A*β*42 | 0.93 | 143 | 0.765 | 204 |
| NfL + pTau217/A*β*40 + pTau181/A*β*42 | 0.93 | 128 | 0.759 | 141 |
| A*β*42/40 + pTau181 + pTau217/A*β*42 | 0.93 | 129 | 0.842 | 120 |
| pTau217/A*β*40 + pTau217/A*β*42 + pTau181/A*β*40 | 0.929 | 156 | 0.743 | 218 |
| pTau217 + pTau217/A*β*42 + pTau181/A*β*42 | 0.929 | 154 | 0.75 | 216 |
| pTau217 + pTau217/A*β*40 + pTau181/A*β*40 | 0.929 | 154 | 0.756 | 217 |
| A*β*42/40 + pTau181 + pTau217/A*β*40 + pTau181/A*β*42 | 0.929 | 126 | 0.86 | 114 |
| A*β*42/40 + pTau217 + pTau181 + pTau217/A*β*40 + pTau217/A*β*42 | 0.929 | 127 | 0.862 | 116 |

| Combination | A+/T+ in BBM+ | n BBM+ | non-A+/T+ in BBM- | n BBM- |
| --- | --- | --- | --- | --- |
| pTau217 + pTau217/A*β*40 + pTau217/A*β*42 + pTau181/A*β*40 | 0.929 | 154 | 0.752 | 214 |
| A*β*42/40 + pTau217 + pTau181 + pTau217/A*β*42 + pTau181/A*β*42 | 0.929 | 126 | 0.861 | 115 |
| A*β*42/40 + pTau181 + pTau217/A*β*40 + pTau217/A*β*42 + pTau181/A*β*42 | 0.929 | 126 | 0.86 | 114 |
| A*β*42/40 + pTau181 + pTau217/A*β*42 + pTau181/A*β*40 + pTau181/A*β*42 | 0.929 | 126 | 0.845 | 116 |
| pTau217/A*β*40 + pTau181/A*β*40 | 0.929 | 156 | 0.748 | 222 |
| pTau217/A*β*40 + pTau181/A*β*42 | 0.929 | 156 | 0.741 | 220 |
| pTau217 + pTau217/A*β*42 + pTau181/A*β*40 | 0.929 | 155 | 0.75 | 216 |
| A*β*42/40 + pTau217 + pTau181 + pTau181/A*β*40 | 0.929 | 126 | 0.852 | 115 |
| A*β*42/40 + pTau181 + pTau217/A*β*42 + pTau181/A*β*42 | 0.929 | 127 | 0.846 | 117 |
| A*β*42/40 + pTau181 + pTau217/A*β*42 + pTau181/A*β*40 | 0.929 | 126 | 0.845 | 116 |
| pTau217/A*β*40 + pTau217/A*β*42 + pTau181/A*β*42 | 0.929 | 156 | 0.739 | 218 |
| A*β*42/40 + pTau217 + pTau181 + pTau217/A*β*40 | 0.929 | 127 | 0.862 | 116 |
| A*β*42/40 + pTau217 + pTau181 + pTau181/A*β*40 + pTau181/A*β*42 | 0.929 | 126 | 0.852 | 115 |
| A*β*42/40 + pTau217 + pTau181 + pTau217/A*β*40 + pTau217/A*β*42 +  pTau181/A*β*42 | 0.928 | 125 | 0.867 | 113 |
| pTau217 + NfL + pTau217/A*β*40 + pTau181/A*β*40 + pTau181/A*β*42 | 0.928 | 125 | 0.765 | 136 |
| A*β*42/40 + pTau181 + pTau217/A*β*40 + pTau181/A*β*40 | 0.928 | 125 | 0.858 | 113 |
| pTau217 + pTau217/A*β*40 + pTau181/A*β*42 | 0.928 | 153 | 0.753 | 215 |
| pTau217 + NfL + pTau217/A*β*42 + pTau181/A*β*40 + pTau181/A*β*42 | 0.928 | 125 | 0.759 | 137 |
| pTau217 + NfL + pTau217/A*β*40 + pTau217/A*β*42 + pTau181/A*β*40 +  pTau181/A*β*42 | 0.928 | 125 | 0.763 | 135 |
| A*β*42/40 + pTau181 + pTau217/A*β*40 + pTau217/A*β*42 + pTau181/A*β*40 +  pTau181/A*β*42 | 0.928 | 125 | 0.858 | 113 |
| A*β*42/40 + pTau181 + pTau217/A*β*40 + pTau217/A*β*42 + pTau181/A*β*40 | 0.928 | 125 | 0.858 | 113 |

| Combination | A+/T+ in BBM+ | n BBM+ | non-A+/T+ in BBM- | n BBM- |
| --- | --- | --- | --- | --- |
| A*β*42/40 + pTau181 + pTau217/A*β*40 + pTau181/A*β*40 + pTau181/A*β*42 | 0.928 | 125 | 0.858 | 113 |
| NfL + pTau217/A*β*40 + pTau181/A*β*40 + pTau181/A*β*42 | 0.928 | 125 | 0.766 | 137 |
| NfL + pTau217/A*β*42 + pTau181/A*β*40 + pTau181/A*β*42 | 0.928 | 125 | 0.754 | 138 |
| A*β*42/40 + pTau217 + pTau181 + pTau217/A*β*42 + pTau181/A*β*40 +  pTau181/A*β*42 | 0.928 | 125 | 0.86 | 114 |
| A*β*42/40 + pTau217 + pTau181 + pTau217/A*β*42 + pTau181/A*β*40 | 0.928 | 125 | 0.86 | 114 |
| pTau217 + pTau217/A*β*40 + pTau217/A*β*42 + pTau181/A*β*42 | 0.928 | 153 | 0.752 | 214 |
| A*β*42/40 + pTau217 + pTau181 + pTau217/A*β*40 + pTau181/A*β*42 | 0.928 | 125 | 0.867 | 113 |
| NfL + pTau217/A*β*40 + pTau217/A*β*42 + pTau181/A*β*40 + pTau181/A*β*42 | 0.928 | 125 | 0.763 | 135 |
| A*β*42/40 + pTau217 + pTau181 + pTau217/A*β*40 + pTau217/A*β*42 + pTau181/A*β*40 + pTau181/A*β*42 | 0.927 | 124 | 0.866 | 112 |
| A*β*42/40 + pTau217 + pTau181 + pTau217/A*β*40 + pTau181/A*β*40 +  pTau181/A*β*42 | 0.927 | 124 | 0.866 | 112 |
| A*β*42/40 + pTau217 + pTau181 + pTau217/A*β*40 + pTau217/A*β*42 +  pTau181/A*β*40 | 0.927 | 124 | 0.866 | 112 |
| A*β*42/40 + pTau217 + pTau181 + pTau217/A*β*40 + pTau181/A*β*40 | 0.927 | 124 | 0.866 | 112 |
| pTau217/A*β*40 + pTau217/A*β*42 + pTau181/A*β*40 + pTau181/A*β*42 | 0.927 | 151 | 0.746 | 213 |
| pTau217 + pTau181 + NfL + pTau181/A*β*42 | 0.927 | 123 | 0.757 | 140 |
| pTau217 + pTau217/A*β*42 + pTau181/A*β*40 + pTau181/A*β*42 | 0.927 | 150 | 0.755 | 212 |
| pTau217/A*β*40 + pTau181/A*β*40 + pTau181/A*β*42 | 0.927 | 151 | 0.749 | 215 |
| A*β*42/40 + pTau217 + pTau181 + NfL | 0.926 | 108 | 0.869 | 84 |
| pTau217 + pTau217/A*β*40 + pTau181/A*β*40 + pTau181/A*β*42 | 0.926 | 149 | 0.758 | 211 |
| pTau217 + pTau217/A*β*40 + pTau217/A*β*42 + pTau181/A*β*40 + pTau181/A*β*42 | 0.926 | 149 | 0.757 | 210 |
| pTau217 + NfL + pTau181/A*β*40 | 0.925 | 133 | 0.745 | 145 |

| Combination | A+/T+ in BBM+ | n BBM+ | non-A+/T+ in BBM- | n BBM- |
| --- | --- | --- | --- | --- |
| A*β*42/40 + pTau217 + pTau181 + NfL + pTau181/A*β*42 | 0.925 | 107 | 0.877 | 81 |
| pTau217/A*β*42 + pTau181/A*β*40 | 0.925 | 159 | 0.738 | 221 |
| pTau217 + pTau181 + NfL | 0.924 | 132 | 0.732 | 153 |
| pTau217 + pTau181/A*β*40 | 0.924 | 158 | 0.747 | 221 |
| pTau217 + pTau181 + pTau181/A*β*42 | 0.924 | 145 | 0.762 | 206 |
| A*β*42/40 + pTau217 + pTau181 | 0.923 | 130 | 0.849 | 119 |
| pTau217 + pTau181 | 0.923 | 156 | 0.747 | 221 |
| pTau217/A*β*42 + pTau181/A*β*40 + pTau181/A*β*42 | 0.922 | 154 | 0.741 | 216 |
| A*β*42/40 + pTau217 + pTau181 + pTau181/A*β*42 | 0.922 | 128 | 0.853 | 116 |
| pTau217 + NfL + pTau181/A*β*40 + pTau181/A*β*42 | 0.921 | 127 | 0.755 | 139 |
| pTau217 + pTau181/A*β*40 + pTau181/A*β*42 | 0.921 | 152 | 0.752 | 214 |
| A*β*42/40 + pTau217 + NfL + pTau217/A*β*42 + pTau181/A*β*42 | 0.92 | 113 | 0.878 | 82 |
| A*β*42/40 + pTau181 + NfL + pTau181/A*β*40 | 0.92 | 112 | 0.854 | 82 |
| A*β*42/40 + pTau217/A*β*40 + pTau217/A*β*42 + pTau181/A*β*42 | 0.92 | 138 | 0.836 | 122 |
| A*β*42/40 + NfL + pTau217/A*β*42 + pTau181/A*β*42 | 0.92 | 113 | 0.867 | 83 |
| A*β*42/40 + pTau181 + pTau181/A*β*40 | 0.92 | 137 | 0.825 | 120 |
| A*β*42/40 + NfL + pTau217/A*β*40 + pTau181/A*β*42 | 0.92 | 113 | 0.888 | 80 |
| A*β*42/40 + pTau217/A*β*40 + pTau181/A*β*42 | 0.92 | 138 | 0.836 | 122 |
| A*β*42/40 + NfL + pTau217/A*β*40 + pTau217/A*β*42 + pTau181/A*β*42 | 0.92 | 113 | 0.888 | 80 |
| A*β*42/40 + pTau181 + NfL + pTau181/A*β*40 + pTau181/A*β*42 | 0.92 | 112 | 0.854 | 82 |
| A*β*42/40 + pTau217 + NfL + pTau217/A*β*40 + pTau217/A*β*42 + pTau181/A*β*42 | 0.92 | 113 | 0.888 | 80 |
| A*β*42/40 + pTau217 + NfL + pTau217/A*β*40 + pTau181/A*β*42 | 0.92 | 113 | 0.888 | 80 |
| A*β*42/40 + pTau217 + pTau217/A*β*40 + pTau181/A*β*42 | 0.919 | 135 | 0.864 | 118 |
| A*β*42/40 + pTau217 + pTau217/A*β*40 + pTau217/A*β*42 + pTau181/A*β*42 | 0.919 | 135 | 0.864 | 118 |
| A*β*42/40 + pTau181 + pTau181/A*β*40 + pTau181/A*β*42 | 0.919 | 136 | 0.825 | 120 |
| A*β*42/40 + pTau217 + NfL + pTau181/A*β*40 | 0.919 | 111 | 0.864 | 81 |

| Combination | A+/T+ in BBM+ | n BBM+ | non-A+/T+ in BBM- | n BBM- |
| --- | --- | --- | --- | --- |
| A*β*42/40 + pTau217 + pTau217/A*β*42 + pTau181/A*β*42 | 0.919 | 136 | 0.858 | 120 |
| A*β*42/40 + pTau217 + NfL + pTau181/A*β*40 + pTau181/A*β*42 | 0.919 | 111 | 0.864 | 81 |
| A*β*42/40 + pTau217 + NfL + pTau217/A*β*42 + pTau181/A*β*40 + pTau181/A*β*42 | 0.918 | 110 | 0.875 | 80 |
| A*β*42/40 + pTau217 + NfL + pTau217/A*β*40 + pTau217/A*β*42 + pTau181/A*β*40 | 0.918 | 110 | 0.885 | 78 |
| A*β*42/40 + NfL + pTau217/A*β*42 + pTau181/A*β*40 | 0.918 | 110 | 0.864 | 81 |
| A*β*42/40 + NfL + pTau217/A*β*40 + pTau181/A*β*40 | 0.918 | 110 | 0.885 | 78 |
| A*β*42/40 + NfL + pTau217/A*β*40 + pTau217/A*β*42 + pTau181/A*β*40 | 0.918 | 110 | 0.885 | 78 |
| A*β*42/40 + pTau217 + NfL + pTau217/A*β*40 + pTau181/A*β*40 | 0.918 | 110 | 0.885 | 78 |
| A*β*42/40 + pTau217 + NfL + pTau217/A*β*42 + pTau181/A*β*40 | 0.918 | 110 | 0.875 | 80 |
| A*β*42/40 + pTau217 + NfL + pTau217/A*β*40 + pTau217/A*β*42 + pTau181/A*β*40 +  pTau181/A*β*42 | 0.918 | 110 | 0.885 | 78 |
| A*β*42/40 + NfL + pTau217/A*β*40 + pTau217/A*β*42 + pTau181/A*β*40 +  pTau181/A*β*42 | 0.918 | 110 | 0.885 | 78 |
| A*β*42/40 + NfL + pTau217/A*β*40 + pTau181/A*β*40 + pTau181/A*β*42 | 0.918 | 110 | 0.885 | 78 |
| A*β*42/40 + pTau217 + NfL + pTau217/A*β*40 + pTau181/A*β*40 + pTau181/A*β*42 | 0.918 | 110 | 0.885 | 78 |
| A*β*42/40 + NfL + pTau217/A*β*42 + pTau181/A*β*40 + pTau181/A*β*42 | 0.918 | 110 | 0.864 | 81 |
| A*β*42/40 + pTau217 + pTau181/A*β*40 | 0.917 | 133 | 0.849 | 119 |
| pTau217 + pTau181/A*β*42 | 0.917 | 157 | 0.748 | 218 |
| A*β*42/40 + pTau217/A*β*40 + pTau181/A*β*40 | 0.917 | 133 | 0.84 | 119 |
| A*β*42/40 + pTau217/A*β*40 + pTau217/A*β*42 + pTau181/A*β*40 + pTau181/A*β*42 | 0.917 | 133 | 0.84 | 119 |
| A*β*42/40 + pTau217 + pTau217/A*β*42 + pTau181/A*β*40 + pTau181/A*β*42 | 0.917 | 132 | 0.856 | 118 |
| A*β*42/40 + pTau217 + pTau181/A*β*40 + pTau181/A*β*42 | 0.917 | 133 | 0.849 | 119 |
| A*β*42/40 + pTau217/A*β*40 + pTau217/A*β*42 + pTau181/A*β*40 | 0.917 | 133 | 0.84 | 119 |

| Combination | A+/T+ in BBM+ | n BBM+ | non-A+/T+ in BBM- | n BBM- |
| --- | --- | --- | --- | --- |
| A*β*42/40 + pTau217/A*β*40 + pTau181/A*β*40 + pTau181/A*β*42 | 0.917 | 133 | 0.84 | 119 |
| A*β*42/40 + pTau217 + pTau217/A*β*42 + pTau181/A*β*40 | 0.917 | 132 | 0.856 | 118 |
| A*β*42/40 + NfL + pTau217/A*β*40 + pTau217/A*β*42 | 0.916 | 119 | 0.884 | 86 |
| A*β*42/40 + pTau217 + NfL + pTau217/A*β*42 | 0.916 | 119 | 0.875 | 88 |
| pTau217 + NfL + pTau181/A*β*42 | 0.916 | 131 | 0.748 | 143 |
| A*β*42/40 + pTau217 + pTau217/A*β*40 + pTau181/A*β*40 | 0.916 | 131 | 0.862 | 116 |
| A*β*42/40 + pTau217 + NfL + pTau217/A*β*40 + pTau217/A*β*42 | 0.916 | 119 | 0.884 | 86 |
| A*β*42/40 + pTau217 + pTau217/A*β*40 + pTau181/A*β*40 + pTau181/A*β*42 | 0.916 | 131 | 0.862 | 116 |
| A*β*42/40 + pTau217 + NfL + pTau217/A*β*40 | 0.916 | 119 | 0.884 | 86 |
| A*β*42/40 + NfL + pTau217/A*β*40 | 0.916 | 119 | 0.884 | 86 |
| pTau217/A*β*40 + pTau217/A*β*42 | 0.916 | 179 | 0.714 | 245 |
| A*β*42/40 + NfL + pTau217/A*β*42 | 0.916 | 119 | 0.865 | 89 |
| A*β*42/40 + pTau217 + pTau217/A*β*40 + pTau217/A*β*42 + pTau181/A*β*40 | 0.916 | 131 | 0.862 | 116 |
| A*β*42/40 + pTau217 + pTau217/A*β*40 + pTau217/A*β*42 + pTau181/A*β*40 +  pTau181/A*β*42 | 0.916 | 131 | 0.862 | 116 |
| pTau217 + NfL + pTau217/A*β*40 | 0.915 | 142 | 0.737 | 156 |
| pTau217 + pTau217/A*β*40 | 0.915 | 176 | 0.728 | 243 |
| NfL + pTau217/A*β*42 | 0.915 | 142 | 0.727 | 154 |
| NfL + pTau217/A*β*40 | 0.915 | 142 | 0.734 | 158 |
| pTau217 + NfL + pTau217/A*β*42 | 0.915 | 142 | 0.732 | 153 |
| NfL + pTau217/A*β*40 + pTau217/A*β*42 | 0.915 | 142 | 0.735 | 151 |
| pTau217 + NfL + pTau217/A*β*40 + pTau217/A*β*42 | 0.915 | 142 | 0.735 | 151 |
| pTau217 + pTau217/A*β*40 + pTau217/A*β*42 | 0.914 | 175 | 0.727 | 238 |
| A*β*42/40 + pTau217 + NfL + pTau181/A*β*42 | 0.913 | 115 | 0.867 | 83 |
| A*β*42/40 + pTau181 + pTau181/A*β*42 | 0.913 | 138 | 0.82 | 122 |
| A*β*42/40 + pTau217 + pTau181/A*β*42 | 0.913 | 138 | 0.851 | 121 |
| A*β*42/40 + pTau217/A*β*40 + pTau217/A*β*42 | 0.913 | 150 | 0.833 | 132 |
| A*β*42/40 + pTau217/A*β*40 | 0.913 | 150 | 0.833 | 132 |
| pTau217/A*β*42 + pTau181/A*β*42 | 0.913 | 161 | 0.733 | 221 |
| pTau181 + NfL + pTau181/A*β*40 | 0.912 | 136 | 0.719 | 153 |
| pTau217/A*β*40 | 0.912 | 182 | 0.714 | 252 |
| A*β*42/40 + pTau217 + pTau217/A*β*40 | 0.912 | 147 | 0.858 | 127 |

| Combination | A+/T+ in BBM+ | n BBM+ | non-A+/T+ in BBM- | n BBM- |
| --- | --- | --- | --- | --- |
| A*β*42/40 + pTau217/A*β*42 + pTau181/A*β*40 | 0.912 | 136 | 0.828 | 122 |
| A*β*42/40 + pTau217 + pTau217/A*β*40 + pTau217/A*β*42 | 0.912 | 147 | 0.858 | 127 |
| A*β*42/40 + pTau217/A*β*42 + pTau181/A*β*40 + pTau181/A*β*42 | 0.912 | 136 | 0.828 | 122 |
| A*β*42/40 + pTau181 + NfL + pTau181/A*β*42 | 0.912 | 114 | 0.855 | 83 |
| pTau217 + pTau217/A*β*42 | 0.91 | 177 | 0.725 | 240 |
| A*β*42/40 + pTau217 + NfL | 0.909 | 121 | 0.865 | 89 |
| pTau181 + NfL + pTau181/A*β*40 + pTau181/A*β*42 | 0.908 | 131 | 0.733 | 146 |
| pTau217 + NfL | 0.906 | 149 | 0.716 | 162 |
| A*β*42/40 + pTau217 + pTau217/A*β*42 | 0.906 | 149 | 0.853 | 129 |
| A*β*42/40 + pTau181 | 0.905 | 147 | 0.803 | 127 |
| pTau217 | 0.903 | 185 | 0.715 | 249 |
| A*β*42/40 + pTau217/A*β*42 + pTau181/A*β*42 | 0.902 | 143 | 0.824 | 125 |
| pTau181 + NfL + pTau181/A*β*42 | 0.902 | 133 | 0.73 | 148 |
| A*β*42/40 + pTau217 | 0.901 | 151 | 0.846 | 130 |
| A*β*42/40 + pTau181 + NfL | 0.901 | 121 | 0.83 | 88 |
| A*β*42/40 + pTau181/A*β*40 | 0.9 | 150 | 0.805 | 128 |
| A*β*42/40 + pTau181/A*β*40 + pTau181/A*β*42 | 0.899 | 148 | 0.805 | 128 |
| A*β*42/40 + NfL + pTau181/A*β*40 + pTau181/A*β*42 | 0.899 | 119 | 0.833 | 84 |
| A*β*42/40 + NfL + pTau181/A*β*40 | 0.899 | 119 | 0.833 | 84 |
| NfL + pTau181/A*β*40 | 0.896 | 144 | 0.705 | 156 |
| pTau181 + pTau181/A*β*40 + pTau181/A*β*42 | 0.893 | 159 | 0.727 | 220 |
| pTau181 + pTau181/A*β*40 | 0.892 | 166 | 0.713 | 230 |
| pTau217/A*β*42 | 0.892 | 186 | 0.71 | 248 |
| NfL + pTau181/A*β*40 + pTau181/A*β*42 | 0.891 | 138 | 0.718 | 149 |
| pTau181 + pTau181/A*β*42 | 0.888 | 161 | 0.722 | 223 |
| A*β*42/40 + NfL + pTau181/A*β*42 | 0.887 | 124 | 0.837 | 86 |
| A*β*42/40 + pTau217/A*β*42 | 0.885 | 157 | 0.822 | 135 |
| NfL + pTau181/A*β*42 | 0.881 | 143 | 0.712 | 153 |
| A*β*42/40 + pTau181/A*β*42 | 0.875 | 160 | 0.772 | 136 |
| pTau181 + NfL | 0.874 | 151 | 0.691 | 165 |
| pTau181/A*β*40 + pTau181/A*β*42 | 0.869 | 176 | 0.703 | 236 |
| pTau181/A*β*40 | 0.866 | 186 | 0.69 | 248 |
| pTau181 | 0.864 | 184 | 0.684 | 250 |
| pTau181/A*β*42 | 0.851 | 188 | 0.683 | 246 |
| A*β*42/40 + NfL | 0.76 | 167 | 0.811 | 95 |
| A*β*42/40 | 0.693 | 270 | 0.689 | 164 |
| NfL | 0.678 | 236 | 0.606 | 198 |
